# Clonal analysis of HIV-1 genotype and function associated with virologic failure in treatment-experienced persons receiving maraviroc: Results from the MOTIVATE phase 3 randomized, placebo-controlled trials

**DOI:** 10.1371/journal.pone.0204099

**Published:** 2018-12-26

**Authors:** Marilyn Lewis, Julie Mori, Jonathan Toma, Mike Mosley, Wei Huang, Paul Simpson, Roy Mansfield, Charles Craig, Elna van der Ryst, David L. Robertson, Jeannette M. Whitcomb, Mike Westby

**Affiliations:** 1 The Research Network, Sandwich, Kent, United Kingdom; 2 hVIVO, Queen Mary BioEnterprise Innovation Centre, London, United Kingdom; 3 Monogram Biosciences, South San Francisco, California, United States of America; 4 University of Oxford, Oxford, United Kingdom; 5 MedImmune Ltd, Cambridge, United Kingdom; 6 Pfizer Global Research and Development, Sandwich Labs, Sandwich, Kent, United Kingdom; 7 Evolution and Genomic Sciences, School of Biological Sciences, The University of Manchester, Manchester, United Kingdom; 8 MRC-University of Glasgow Centre for Virus Research, Glasgow, United Kingdom; 9 Centauri Therapeutics Limited, Discovery Park, Kent, United Kingdom; Rush University, UNITED STATES

## Abstract

Detailed clonal phenotypic/genotypic analyses explored viral-escape mechanisms during maraviroc-based therapy in highly treatment-experienced participants from the MOTIVATE trials. To allow real-time assessment of samples while maintaining a blind trial, the first 267 enrolled participants were selected for evaluation. At failure, plasma samples from 20/50 participants (16/20 maraviroc-treated) with CXCR4-using virus and all 38 (13 maraviroc-treated) with CCR5-tropic virus were evaluated. Of those maraviroc-treated participants with CXCR4-using virus at failure, genotypic and phenotypic clonal tropism determinations showed >90% correspondence in 14/16 at Day 1 and 14/16 at failure. Phylogenetic analysis of clonal sequences detected pre-treatment progenitor CXCR4-using virus, or on-treatment virus highly divergent from the Day 1 R5 virus, excluding possible co-receptor switch through maraviroc-mediated evolution. Re-analysis of pre-treatment samples using the enhanced-sensitivity Trofile^®^ assay detected CXCR4-using virus pre-treatment in 16/20 participants failing with CXCR4-using virus. Post-maraviroc reversion of CXCR4-use to CCR5-tropic occurred in 7/8 participants with follow-up, suggesting selective maraviroc inhibition of CCR5-tropic variants in a mixed-tropic viral population, not emergence of *de novo* mutations in CCR5-tropic virus, as the main virologic escape mechanism. Maraviroc-resistant CCR5-tropic virus was observed in plasma from 5 treated participants with virus displaying reduced maximal percent inhibition (MPI) but no evidence of IC_50_ change. Env clones with reduced MPI showed 1–5 amino acid changes specific to each V3-loop region of env relative to Day 1. However, transferring on-treatment resistance-associated changes using site-directed mutagenesis did not always establish resistance in Day 1 virus, and key ‘signature’ mutation patterns associated with reduced susceptibility to maraviroc were not identified. Evolutionary divergence of the CXCR4-using viruses is confirmed, emphasizing natural selection not influenced directly by maraviroc; maraviroc simply unmasks pre-existing lineages by inhibiting the R5 virus. For R5-viral failure, resistance development through drug selection pressure was uncommon and manifested through reduced MPI and with virus strain–specific mutational patterns.

## Introduction

HIV-1 entry into cells requires initial virus-cell contact via the CD4 receptor followed by co-receptor binding, most commonly through CCR5 (R5) [1]. CXCR4 provides an alternative co-receptor, with increased prevalence of CXCR4-using virus in later stages of disease in approximately 50% of infected individuals [2]. Emergence of CXCR4-using virus is a complex process, with lineages arising independently during the course of infection and the development of clinically relevant variants depending on both viral and host factors [3]. Dual-tropic or mixed-tropism (DM) viral populations can use both the CCR5 and CXCR4 co-receptors (dual tropic) or comprise a mixed tropism population, where viruses use either co-receptor and are co-existing [4,5]. As dual- or mixed-tropic viruses cannot be distinguished readily in phenotypic or genotypic assays, they are referred to as infections with DM tropism; however, clonal analyses can distinguish true dual-tropic virus from CXCR4-tropic (X4) virus. Tropism can also be predicted using the amino acid sequence of the 3rd variable loop of env (V3-loop) and viruses classified as R5 or non-R5 [6–9].

Unique among the current approved antiretroviral drugs, maraviroc (a CCR5 antagonist) binds with high affinity to host protein, CCR5, on the cell surface rather than to a viral protein [10]. This causes conformational changes that prevent appropriate docking of R5 HIV-1 strains and subsequent entry into the cell [10–14]. Maraviroc binds to CCR5 deep within a hydrophobic pocket formed by 6 of the 7 CCR5 transmembrane helices [15]. The binding site is remote from the HIV-1 gp120 binding site on Extracellular Loop 2 and the N-terminal of CCR5 [16–18], so it can be inferred that maraviroc acts allosterically [19]. CXCR4-using viruses, which include X4-, mixed-, and dual-tropic viruses, are inherently insensitive to maraviroc. Therefore, a sensitive tropism test needs to be performed prior to initiating treatment with a MVC-containing regimen.

There are two potential mechanisms of ‘viral escape’ from maraviroc: *de novo* mutation resulting in resistance in an R5 virus or through emergence of virus populations that use CXCR4 for entry (either through exposure of a pre-existing CXCR4-using minority virus population in the presence of maraviroc or through *de novo* mutation in an R5 variant enabling the use of CXCR4) [19–21]. Consistent with maraviroc acting through an allosteric, cell-binding mechanism, the primary phenotypic expression of resistance in R5 virus *in vitro* is a reduction in the maximal percent inhibition (MPI) rather than an increase in the 50% inhibitory concentration (IC_50_) [19,20]. The emergence of CXCR4-using virus during treatment with CCR5 antagonists was first described for two individuals undergoing short-term monotherapy with maraviroc [21]. Phylogenetic analysis in this and subsequent studies indicated that the CXCR4-using variants emerged from a pre-existing CXCR4-using reservoir rather than via a co-receptor switch of a CCR5-tropic clone under selection pressure from maraviroc [22,23]. This is consistent with *in vitro* studies demonstrating that co-receptor switching is a significant evolutionary change requiring sequential accumulation of multiple mutations in the V1/2 and/or V3 regions of gp120 [24].

The safety and efficacy of maraviroc as a component of antiretroviral regimens for treatment-experienced persons infected with R5 HIV-1 was demonstrated in the MOTIVATE 1 and MOTIVATE 2 clinical studies [25,26]. In these studies, the efficacy of maraviroc taken once or twice daily was significantly superior to placebo when given with optimized background therapy in all primary and secondary virologic and immunologic endpoints at Week 48 [25,26].

We examined the mechanisms of resistance to maraviroc in the MOTIVATE trials in detail to extend previous observations of CXCR4-using virus on-treatment and to investigate the origin of this virus (pre-existing or selected on-treatment) using clonal analysis to determine phenotype and phylogenetic relatedness. As participants were selected prior to unblinding the study, both maraviroc- and placebo-treated participants were included. In addition, R5 virus identified with reduced susceptibility to maraviroc was studied to establish whether resistance can be associated with any consistent mutational pattern in the V3-loop.

## Results

### Population

**Fig 1:**
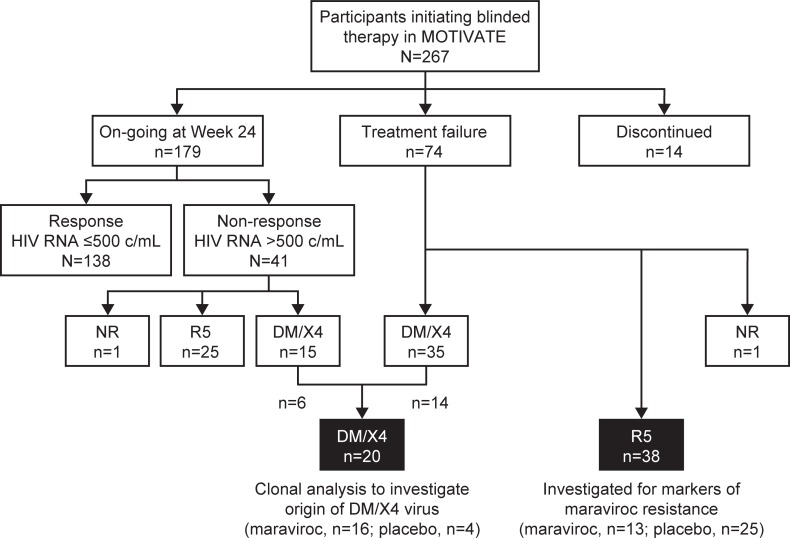
Characterization of participants from MOTIVATE 1 and 2 for analysis of mechanisms of virologic failure.

The first 267 participants enrolled in the MOTIVATE 1 and 2 clinical studies were included in this study, and the analysis was performed after the last participant had reached Week 24 and while the study was still fully blinded (S1 Fig). Individuals were categorized according to their response to treatment and their tropism testing results. A total of 73 participants with treatment failure together with 15 participants with DM/X4 virus on completing Week 24 with HIV-1 RNA >500 copies/mL were identified (N = 88; tropism: CXCR4-using, n = 50; R5-tropic, n = 38) (Fig 1). Paired Day 1 and on-treatment viruses from all 38 participants with R5 virus at treatment failure using the original Trofile assay (Monogram Biosciences, South San Francisco, CA, USA) were studied further using detailed clonal analysis of tropism, maraviroc susceptibility, and V3-loop genotype. In addition, 20 of the 50 participants with CXCR4-using virus on-treatment were selected using a random procedure (see Materials and Methods) to ensure proportionate representation by time of failure and treatment (Fig 1).

The exploratory analysis of mechanisms associated with virologic failure in the MOTIVATE 1 and 2 studies was carried out on a subset of the first 267 participants to be enrolled in the studies. Participants were categorized according to treatment status, viral load, tropism (original Trofile), and time of treatment failure. Participants excluded from the analysis comprised 138 who remained on treatment at Week 24 (Responders) with an HIV-1 RNA below the protocol-defined limit for running a tropism assay (<500 copies/mL) and 14 who discontinued treatment before Week 24 for reasons other than protocol-defined treatment failure. Filled (black) boxes indicate participants selected for analysis. DM/X4, CXCR4-using virus (ie, X4- and dual- or mixed-tropic); NR, no valid tropism result; R5, CCR5-tropic virus.

The clinically relevant demographic and randomization characteristics of all 267 participants as well as the 58 participants studied are summarized together with the total population in S1 Table. Overall, the populations showed similar demographic characteristics for sex (89%-95% were male), race (81%-87% White), and viral subtype (95% subtype B) as those of the total study population. There was an over-representation of participants with plasma HIV-1 RNA ≥100,000 copies/mL among the 267-participant sub-population and the failure participants (total population, 42%; sub-population, 51%; failure set, 59%), and also of those with CD4 cell counts <50 cells/mm^3^ (total population, 20%; sub-population, 28%; failure set, 48%). Further analysis of the sub-population indicated generally similar proportions of the 267 participants in treatment groups and with treatment failure as for the total population (S1 Table). Retrospectively, 29 of the 58 participants selected for study were found to have been assigned to the placebo treatment group, including 4 of the 20 with CXCR4-using virus and 25 of the 38 with R5 virus at failure.

### Evaluation of participants failing with CXCR4-using virus

Viral clones from pre-treatment (Day 1 pre-dose, n = 192 clones) and on-treatment (n = 48 clones) plasma HIV-1 RNA from each of the 20 selected participants with CXCR4-using viral populations underwent initial testing for co-receptor use with a simplified, single-well, pseudo-typed virus infectivity assay (see Materials and Methods). Additionally, a region of gp160 env spanning the V3-loop was sequenced for each clone.

### Analysis of the pre-treatment plasma samples

Virus from 5 of the 20 participants had a change in Trofile result between screening (R5) and Day 1 (DM; 3 were subsequently treated with maraviroc and 2 with placebo).

For plasma from all 20 participants, a median of 104 functional Day 1 clones were observed per participant (range, 73–143), providing a sensitivity to detect 2%-4% CXCR4-using clones with 95% probability. The V3-loop was successfully sequenced for the majority of Day 1 clones (success rate: median, 91% [range, 68%-97%]; sensitivity to detect CXCR4-using clones: median, 1.7% [range, 1.6%-2.3%]).

Results from the infectivity analysis of clones were generally consistent with the Trofile findings. Among all 16 maraviroc-treated participants, the majority of clones showed R5-tropic phenotype at Day 1 (median, 98%; range, 100–53%; S2 Table). In 4 of the 5 instances where a change in Trofile result between Screening and Day 1 was observed (maraviroc-treated, n = 2; placebo-treated, n = 2), the number of non-R5 clones was >15% using phenotype or genotype at Day 1. In the fifth instance (Participant Identifier [PID] Tropism [T]17: maraviroc-treated), 2% and 3% of clones showed non-R5 tropism by phenotype and genotype, respectively (S2 Table).

There was >90% concordance between genotypic and phenotypic tropism determination in clones from Day 1 virus from 14 of 16 of the maraviroc-treated participants; of the other participants, T825 had only R5 virus present by genotype but 30% dual-tropic clones by phenotype, and T246 had mostly non-R5 clones using genotype (98/104, 94%) but mostly R5-using phenotype (83/104, 80%), with overall 26% concordance. In addition, in clones from the Day 1 isolates from 2 of the 4 participants who received placebo, there was >97% concordance of genotype and phenotype tropism. Of the other 2 participants, T397 had only non-R5 clones detected using genotype but 61% R5 by phenotype, and in the Day 1 isolate from T57, the infectivity assay found R5 virus in 105 of 107 clones (98%), whereas genotype detected non-R5 virus in 105 of 107 clones (98%) (S2 Table).

#### Analysis of the on-treatment plasma samples

The infectivity analysis of clones on treatment was less comprehensive than at Day 1 because it was anticipated that the suppression of R5 virus by maraviroc would give rise to enhanced proportions of CXCR4-using virus. On treatment, a median of 23 functional clones (range, 13–32) were analyzed successfully in the infectivity assay, sufficient to detect 9%-21% CXCR4-using clones with 95% probability. The V3-loop was successfully sequenced for the majority of clones (success rate: median, 94% [range, 56%-100%]; sensitivity: median 6.5%, [range, 6.1%-10.5%]).

The on-treatment isolates from 15 of the 16 participants receiving maraviroc had a majority of non-R5 clones with both phenotypic and genotypic determinations (median total number of clones, 19; non-R5 range phenotype: 13–31, 80%-100%; genotype: 12–32, 75%-100%). On-treatment clones from the other participant (T347) showed 65% R5 tropism using both phenotypic and genotypic determinations (S2 Table).

Among the 4 placebo-treated participants, clones from the on-treatment virus had predominantly R5-tropic phenotype in 3 participants. Clones from the participant with the greatest proportion of R5 viral clones (90%, T132) included 2 of 20 (10%) X4 clones using both phenotypic and genotypic methods; this small proportion of virus with exclusively X4 tropism appears to have resulted in the phenotypic DM assignment at the viral population level, which is consistent with the previously demonstrated sensitivity to detect minor CXCR4-using variants (original Trofile, 5%-10%).

#### Confirmatory clonal analysis

A confirmatory analysis using the original Trofile assay was performed on 11 to 12 selected clones from each of the time points for all 20 participants (475 clones total; S3 Table). There were similar proportions of functional clones between the time points (Day 1: 218/237, 92%; on-treatment: 217/238, 91%). For these clones, phenotype/genotype concordance was high both at Day 1 and on treatment (Day 1: 191/218, 88%; on-treatment: 204/217, 94%). The majority of the discordance (30/40, 75%) occurred when the phenotype result indicated R5 tropism and the genotypic tropism prediction was non-R5 both before and after treatment (Day 1: 20/27, 74%; on-treatment: 10/13, 77%). Fourteen discordant clones (Day 1: 10; failure: 4) were all from one participant (T57).

### CXCR4-using virus detected during blinded treatment originated from a pre-existing CXCR4-using virus population

**Fig 2:**
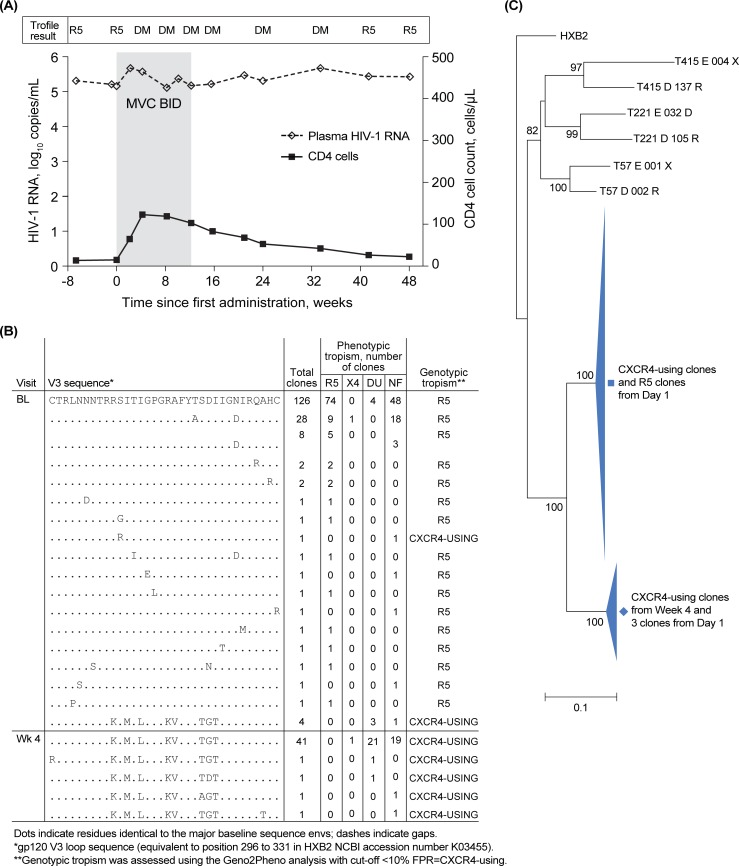
An example of detection of CXCR4-using env clones at low frequency in the Day 1 sample. (A) Change in viral load (◊ dashed line) and CD4 (▪ solid line) for participant T285, who received maraviroc together with 1 additional active antiviral agent, saquinavir/r, for 12 weeks (shaded area) and experienced protocol-defined treatment failure (PDTF) at Week 8. Viral tropism as measured using the original Trofile assay in the clinical program is shown above the plot. At Week 4 of treatment, the participant’s plasma virus was classified as DM. Clonal analyses were performed on plasma stored from Day 1 (pre-treatment; plasma HIV-1 RNA, 141,000 copies/mL) and Week 4 (on-treatment; plasma HIV-1 RNA, 381,000 copies/mL). (B) V3 sequence alignments and tropism assignments for clones tested at Day 1 (BL) and Week 4 (WK4). CXCR4-using clones from the Week 4 sample shared the same sequence as CXCR4-using clones present as a minority in the Day 1 sample. (C) Diagrammatic representation of a phylogenetic tree for the participant based on the neighbor-joining tree for Day 1 and Week 4 env cones. The tree is rooted using HXB2 and includes examples of circulating virus from participants T57, T221, and T415. The bootstrap values have been added to the nodes. The maximum likelihood tree for this participant’s clonal sequence data is provided in S3 Fig. BL, Day 1; BID, twice daily; DM, dual- or mixed-tropic virus; DU, dual tropic; MVC, maraviroc; NF, non-functional; R5, CCR5-tropic virus.

Amino acid sequences and tropisms of the V3-loop are included for all 20 participants in Fig 2 and S2 Table. The phylogenetic analysis of the nucleotide sequences of pre- and on-treatment cloned virus from each participant is included in the supplementary information in the form of preliminary neighbor-joining trees (S2 Fig) and confirmatory maximum likelihood trees (S3 Fig). The evolutionary history inferences are in agreement, showing that the sequences from each participant form a distinct monophyletic cluster. In all 20 participants, the CXCR4-using viruses observed on-treatment were phylogenetically distinct from the R5-tropic population present before treatment (S2 Fig). This CXCR4-using sub-population included detectable CXCR4-using progenitors before treatment in 14 of 20 (70%) instances (maraviroc-treated, 11/16 [69%]; placebo-treated, 3/4 [75%]). In the 6 (30%) instances (maraviroc-treated, 5/16; placebo-treated, 1/4) with no CXCR4-using progenitors observed before treatment, the emerging CXCR4-using virus was sufficiently distinct from the R5 virus present at treatment initiation for it not to have been the CXCR4 progenitor. This finding was supported by the high bootstrap replicate values indicating a high level of confidence in the analysis (minimum bootstrap value, 75%). Collectively, these evolutionary inferences indicate the presence of a pre-existing CXCR4-using sub-population that becomes detectable only after inhibition by maraviroc of the dominant R5-tropic population. Thus, emergence of CXCR4-using virus is through a passive rather than active maraviroc selection process.

A diagrammatic representation of a phylogenetic tree for clones from one participant (T285) who failed to respond to treatment, maintaining HIV-1 RNA 100,000 to 1,000,000 copies/mL and CD4 cell count <100 cells/mm^3^ throughout, is shown in Fig 2A. Visual inspection revealed clones with amino acid variants at 8 positions (10, 12, 14, 18, 19, 23, 24, 25) common between 4 clones at Day 1 (3 dual tropic and 1 non-functional by phenotype) and all 51 clones from Week 4 (Fig 2B). The phylogenetic analysis at the nucleotide level further supported the inferred pre-existence of a progenitor to the on-treatment CXCR4-using viral strain that is distinct from the R5 virus present at Day 1; the tree bifurcated with 1 branch containing all the on-treatment DM and X4 clones together with 3 Day 1 and no Day 1 R5 viral clones (Fig 2C).

**Fig 3:**
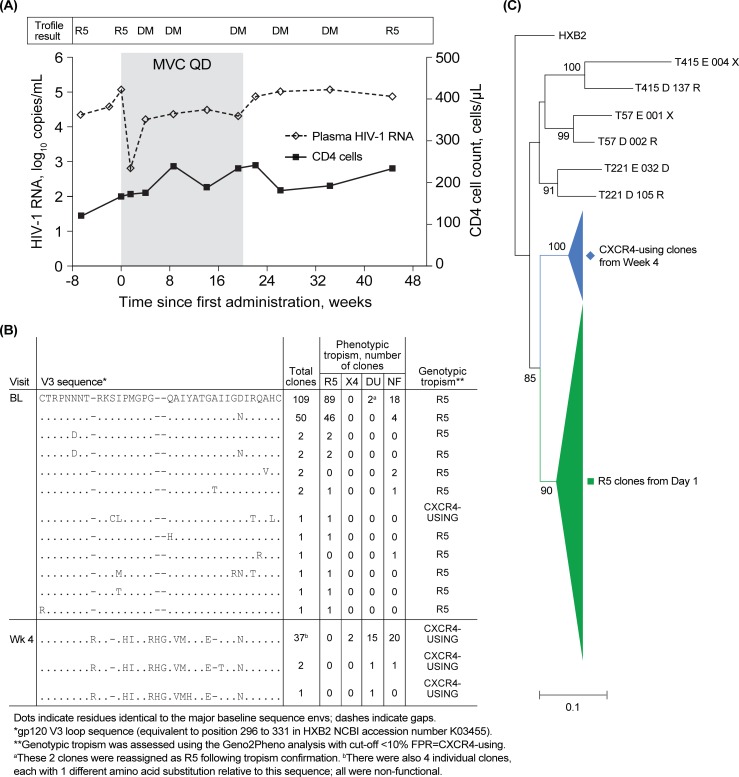
An example of a participant with no CXCR4-using env clones detected in the Day 1 sample. (A) Change in viral load (◊ dashed line) and CD4 (▪ solid line) for participant T398, who received maraviroc with no additional active drugs for 134 days (Week 20, shaded area) and failed therapy at Week 8. Viral tropism as measured by the Trofile assay in the clinical program is shown above the plot. Clonal analyses were performed on plasma stored from Day 1 (pre-treatment; plasma HIV-1 RNA, 115,000 copies/mL) and when the tropism of the plasma was assigned as DM at Week 4 (plasma HIV-1 RNA, 16,400 copies/mL). Results of the clonal tropism analysis are shown in the embedded table. (B) V3 sequence alignments and tropism assignments for clones tested at Day 1 (BL) and Week 4 (WK4). CXCR4-using clones from the Week 4 sample shared the same sequence as CXCR4-using clones present as a minority in the Day 1 sample. (C) Diagrammatic representation of a phylogenetic tree for participant T398 based on the neighbor-joining tree. The tree is rooted using HXB2 and includes examples of circulating virus from participants T57, T221, and T415. The maximum likelihood tree is provided in S3 Fig. BL, Day 1; DM, dual- or mixed-tropic virus; DU, dual tropic; MVC, maraviroc; NF, non-functional; QD, once daily; R5, CCR5-tropic virus.

Fig 3 details the results obtained for one participant in whom no potential progenitor CXCR4-using virus was detected in the Day 1 plasma sample. Using phenotype, most of the functional pre-treatment clones (145/147, 98.6%) showed R5 tropism (dual tropism, n = 2); by contrast, all of the functional on-treatment clones (n = 19) at Week 4 showed CXCR4-using characteristics (89% dual and 11% X4). Genotypically, the clones showed 99.3% R5 and 100% CXCR4-using tropism characteristics pre-treatment and on-treatment, respectively. Pre-treatment V3-loop amino acid sequences showed limited variability with 2 sequences accounting for 159 of the 173 clones (Fig 3B). All the clones at Week 4 had a distinct V3-loop with 10–11 amino acid sequence differences from the Day 1 R5 sequences and showed predictive CXCR4-using genotypic features. The phylogenetic tree bifurcated with 1 branch containing all CXCR4-using clones from Week 4 and another branch containing all the Day 1 R5 clones (Fig 3C). Collectively, these data show that the CXCR4-using clones identified on-treatment were distinct from the R5 clones detected at Day 1 and that CXCR4-using virus was present at low prevalence prior to maraviroc therapy.

### The majority of participants with CXCR4-using virus at failure also had CXCR4-using virus present at screening when tested retrospectively using a more sensitive tropism assay

A retrospective phenotypic re-analysis of tropism at Screening was performed using an enhanced sensitivity version of the Trofile assay (ESTA), which confirmed pre-existence of CXCR4-using virus at Screening in 13 of the 16 (81%) maraviroc-treated participants included in the clonal analysis, who were originally classified as having R5 virus using the Trofile assay (S2 Table). Thus, 81% of this subgroup who failed maraviroc treatment with CXCR4-using virus would not have been recruited to the study had ESTA been available for screening. Consistent with an R5-tropism result from the ESTA analysis, all 3 participants whose screening test showed R5 virus using ESTA had no functional CXCR4-using clones at Day 1 (T251, T398, and T415; S3 Table)

### Tropism change on-treatment is reversible after stopping maraviroc treatment

Eight of the 16 participants who received maraviroc and failed with CXCR4-using virus had at least one post-treatment virology follow-up sample. In 7 of these (87.5%), the virus reverted to R5 tropism after maraviroc was withdrawn, even if the exposure to maraviroc had continued for some time following first detection of CXCR4-using virus (median time from initial observation of CXCR4-using virus to last on-treatment dose: 55 days; range, 13–105 days). Both individuals with detailed clonal analysis exemplified in Figs 2 and 3 showed post-maraviroc reversion from CXCR4-using to R5 virus despite all on-treatment clones showing CXCR4-using characteristics. In the remaining participant, the only post-treatment observation showed X4 tropism 196 days after stopping maraviroc (T415); in this participant, the time from initial DM-tropism observation to last on-treatment dose was 839 days.

### Evaluation of participants failing with CCR5 virus

#### Reduced maximum percentage inhibition, rather than shifts in IC_50_, was a marker of reduced maraviroc susceptibility in participants failing maraviroc therapy with R5 virus

Maraviroc susceptibility was assessed using the PhenoSense™ HIV Entry assay in virus from paired pre-treatment (Day 1) and failure (on blinded treatment) plasma taken from all 38 participants who failed treatment with R5 virus, regardless of whether they received maraviroc or placebo. Susceptibility results were obtained for all but 2 individuals (maraviroc, n = 11/13; placebo, n = 25/25). At Day 1, the R5 virus showed full susceptibility to maraviroc by IC_50_ (geometric mean IC_50_ fold-change [FC] relative to reference virus JRCSF: maraviroc, 0.84 [range, 0.32–1.58]; placebo, 0.67 [range, 0.22–2.65]) with all viruses showing MPI ≥95% (S4 Table).

There were no apparent differences of the IC_50_ FC relative to the JRCSF reference strain of HIV-1 at the time of failure in the maraviroc or the placebo treatment groups (geometric mean IC_50_ FC maraviroc: 1.0 [range, 0.38–2.42]; placebo: 0.65 [range, 0.22–2.82]; *P*>0.05; S4 Table). However, one individual (PID 21) who received maraviroc had a modest 3.1 FC in maraviroc susceptibility between Day 1 and treatment failure time points; the MPI was 100% at both time points (S5 Table).

**Fig 4:**
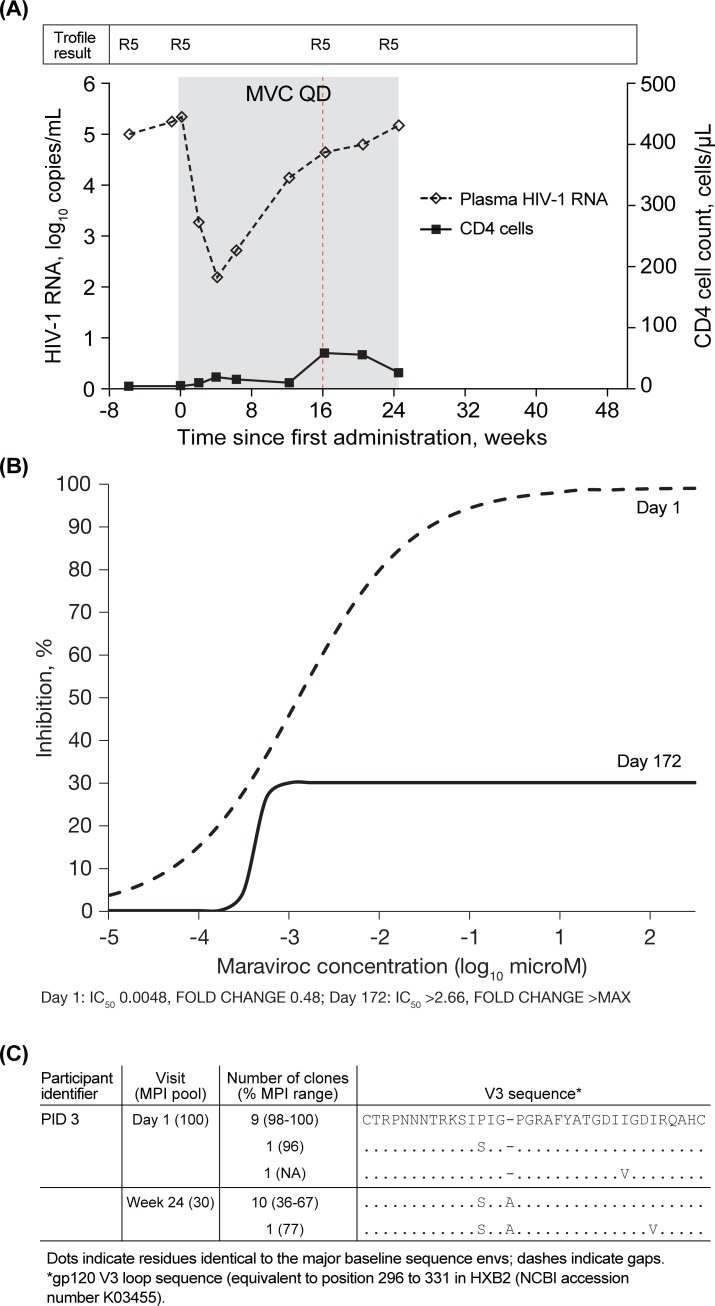
Example of failed maraviroc-containing treatment with R5 virus showing a plateau in MPI (indicates resistance). (A) Change in viral load (◊ dashed line) and CD4 (▪ solid line) for participant 3, who received maraviroc in combination with emtricitabine (FTC), atazanavir (ATV), and enfuvirtide (ENF) for 24 weeks (shaded area) and failed therapy at Week 16 (red vertical dotted line) with R5 virus. Regimen prior to Day 1: none. OBT from Day 1: FTC, ATV, ENF. Screening: FTCres, ATVres, ENFsens. Failure: FTCres, ATVres, ENFres (V38E). (B) The dose-response curves obtained in the PhenoSense Entry assay at Day 1 and at Day 172 (the last on-treatment time point). On Day 1, the dose-response curve reaches >95% inhibition and an IC_50_ can be calculated. On day 172, the dose-response curve plateaus at 30%, indicating resistance to maraviroc. (C) V3 sequence alignments for clones tested at Day 1 and Week 24. MPI, maximal percent inhibition; MVC, maraviroc; OBT, optimized background treatment; QD, once daily; R5, CCR5-tropic virus.

Reduced MPI were observed in viruses from 4 of the 11 maraviroc-treated participants and 1 placebo-treated participant who received open-label maraviroc following failure (PID16; MPI, 30%-91%; S5 Table). One example of an individual in whom reduced MPI was observed is shown in Fig 4. This participant (PID 3) received maraviroc in combination with optimized background treatment (OBT) for 24 weeks and experienced therapy failure with R5 virus at Week 16 (Fig 4A). On Day 1, the maraviroc dose-response curve reached >95% inhibition and an IC_50_ could be calculated (Fig 4B). At Week 24, maraviroc MPI 30% was observed, and IC_50_ could not be obtained (MPI <50%). Viral load, CD4, and drug-treatment data for the other 3 participants receiving maraviroc and the participant who received placebo are provided in S4 Fig. Clonal maraviroc susceptibility data and sequences are shown in S5 Table.

Twelve env clones per sample were analyzed from 6 participants with possible phenotypic markers of maraviroc resistance (maraviroc, n = 5; placebo/open-label maraviroc, n = 1). The results were consistent with the population phenotypic findings, as MPI <95% was observed in all env clones from the treatment failure time points of all 4 maraviroc-treated participants, and the participant treated with open-label maraviroc after placebo, with reduced MPI in the population sample (S5 Table). Whilst MPI was found to vary between individual env clones from a single sample, the distribution and average MPI amongst the 12 env clones analyzed was consistent with the MPI observed with the population sample from which they were derived (see result for PID 3: population MPI, 30%; cloned virus range, 36%-77%; Fig 4C). The clonal analysis of serial samples from the individual, who went on to receive open-label maraviroc after experiencing failure during blinded treatment with maraviroc BID (Week 8), shows a similar pattern (PID 12; S5 Table; S4 Fig). Although there were no env clones detected with MPI <95% in the Day 1 sample, at Week 8, all 12 env clones had reduced MPI to maraviroc. A later sample, taken 49 days after stopping maraviroc treatment, demonstrated MPI <95% in 7 of 12 clones. A Week 32 sample, taken 77 days after re-initiating treatment with open-label maraviroc, once again revealed reduced MPI in all 12 clones.

Both Day 1 and on-treatment clones from the participant with a significant IC_50_ FC to maraviroc (PID 21, 3.1 FC) did not show any reduction in MPI (MPI 100%: Day 1, 12/12; Failure, 12/12), and the susceptibility of the clonal virus was also in good agreement with the population data (geometric mean IC_50_ FC, 3.05; range, 1.46–4.4).

### Changes in the gp120 V3-loop were identified in all env clones with reduced MPI from participants failing a maraviroc-containing regimen

In participants with reduced maraviroc MPI, those viral clones with reduced MPI were associated with 1 to 5 amino acid changes in the V3-loop relative to Day 1 (S5 Table). The majority of changes in the V3-loop occurred in the stem and loop rather than the base of the loop. However, the gp120 V3-loop amino acid sequences were unique for each of the 5 participants, and any changes associated with reduced MPI were specific for that sequence. For example, an insertion of alanine between residues 15 and 16 of the V3-loop of clones from Week 24 virus from a participant (PID 3, Fig 4C) was associated with reduced MPI. This was not observed in any of the viral clones from the other participants (S5 Table). Interestingly virus from Week 24 of another individual (PID 8) included 2 clones with identical V3-loops to those with reduced MPI but showed no loss of susceptibility, suggesting that regions outside the V3-loop influence maraviroc susceptibility. In summary, no specific signature mutations were identified that could reliably predict loss of maraviroc susceptibility in R5 viruses.

Changes in the V3 loop of viral clones from 4 participants during placebo treatment were also observed. Usually, there were only 0 to 2 amino acid changes, but in one individual (PID 18), a variant with 6 different amino acids was selected on treatment (S5 Table). This demonstrates that variations in the V3-loop sequence can occur with or without maraviroc treatment.

### Changes in the gp120 V3-loop play a key role in conferring maraviroc resistance

In order to determine whether the V3-loop amino acid changes observed following treatment with maraviroc were associated with reduced susceptibility and not unrelated changes as those seen in participants receiving placebo, site-directed mutagenesis (SDM) was performed. To assess whether the changes seen in the on-treatment samples of the 4 maraviroc-treated participants with reduced MPI were both sufficient and necessary to confer reduced susceptibility to maraviroc, SDM was used to insert the observed on-treatment amino acid variants into a Day 1 derived viral clone or to remove it from the on-treatment variant clones to re-establish the Day 1 V3-loop sequence (Table 1).

**Table 1:** Maraviroc susceptibility of parental and site-directed mutagenesis Env clones.

Participant ID	Clone ID	MPI (%)	V3 Sequence
PID 3	Day 1	100	CTRPNNNTRKSIPIG-PGRAFYATGDIIGDIRQAHC
	Week 24	51	............S..A....................
	Day 1 (SDM V3 Fail)	41	............S..A....................
	Week 24 (SDM V3 Day 1)	98	....................................
PID 8	Day 1	100	CTRPGNNTRKSIHMG-PGSSIYATGAIIGDIRQAHC
	Week 24	63	....................F....DV.........
	Day 1 (SDM V3 Fail)	85	....................F....DV.........
	Week 24 (SDM V3 Day 1)	99	....................................
PID 12	Day 1	100	CTRPNNNTRKGIHIG-PGRSFYATGDIIGDIRQVHC
	Week 8	55	.I........S........... ....V... ...A..
	Day 1 (SDM V3 Fail)	100	....... ...S........... ....V.........
	Week 8 (SDM V3 Day 1^a^)	99	.I........................... ....A..
PID 11	Day 1	96	CIRPNNNTRKSINIG-PGRAWYTTGDIIGDIRQAHC
	Week 8	50	.T....... ...H.....K ...A.............
	Day 1 (SDM V3 Fail)	66	............H................... ....
	Week 8 (SDM V3 Day 1^a^)	91	.T................K ...A.............

V3 Sequence gp120 V3-loop sequence position in HXB2 (NCBI accession number K03455) env is 296 to 331. Dots indicate residues identical to the Day 1 maraviroc-sensitive envs and dashes indicate gaps.

^a^These sequences do not match exactly the V3-loop Day 1 as 2/3 additional amino acid changes are present in the Week 8 clonal sequences.

In cloned virus from 2 of the 4 participants (PID 3 and PID 8), the changes observed in the V3-loop appeared to be necessary and sufficient to establish reduced MPI (P13S/G15GA and I20F/A25D/I26V; Table 1). However, a clone from another participant (PID 12) showed reduced MPI with 4 changes from Day 1: T2I, G11S, I26V, and V34A. Removal of 2 mutations (G11S, I26V) restored full susceptibility to the on-treatment virus; however, insertion of G11S and I26V into the Day 1 clone did not give rise to reduced susceptibility. For virus from the fourth individual (PID 11), the insertion of N13H resulted in a significant reduction of MPI when inserted into the Day 1 clone (Table 1). Removal of this variant (but retention of I2T, R18K, T22A) from the clone derived from the on-treatment sample resulted in a marked increase in MPI but did not fully restore susceptibility to maraviroc. These latter 2 instances suggest that regions outside the V3-loop might contribute to maraviroc resistance.

### Phenotypic markers of maraviroc resistance were qualitatively reproduced with clonal recombinant virus in peripheral blood lymphocytes

Individual env clones with V3-loop genotypes similar to the consensus sequences were selected from maraviroc-treated participants with reduced MPI or IC_50_ FC and were tested in a peripheral blood lymphocyte (PBL)–based assay (S6 Table). There was concordance between PBL and PhenoSense HIV Entry results with Day 1 samples (MPI ≥95%; S6 Table). At failure, MPI showed some variability between tests in PBLs, although there were similarities (eg, the same clones showed MPI <50% using both methods). Of note, one participant (PID 3) had on-treatment MPI 37% using PhenoSense HIV Entry, whereas MPI using PBL was negative (–27%). There were greater differences between IC_50_ FC values, with the PBL assay consistently providing lower values. One viral clone from the participant with plasma viral IC_50_ FC 3.1 was tested using PBL, with a qualitatively similar result observed (IC_50_ FC 4.52).

### Cross-resistance was not always observed to other entry inhibitors (aplaviroc and enfuvirtide)

Cross-resistance with another CCR5 antagonist, aplaviroc (APL), was assessed in 4 of the 5 participants with reduced MPI. Only virus from PID 3 showed possible cross resistance with aplaviroc (Table 2). The aplaviroc MPI for viruses obtained from the other 3 participants’ pre- and post-maraviroc treatment ranged from 98% to 100%. These findings are consistent with the previous analysis suggesting different binding of these 2 antagonists to CCR5 [27]. Susceptibility to the fusion inhibitor, enfuvirtide, was also assessed (Table 2). All 4 participants were treated with enfuvirtide in the background regimen and resistance was found at Day 1 in 3 of the 4 participants. Thus, although increased fold-change relative to Day 1 was observed in virus from 2 of the 4 participants, a mechanism involving cross-resistance between maraviroc and enfuvirtide cannot be inferred.

**Table 2:** Viral susceptibility to aplaviroc and enfuvirtide in virus from participants with resistance to maraviroc.

PID/Time point	Maraviroc MPI	Aplaviroc MPI	Enfuvirtide IC_50_ fold-change^a^
PID 3/Day 1	100	99	0.31/—
PID 3/Failure^b^	30	90	>MAX/>MAX
PID 12/Day 1	100	98	161/—
PID 12/Failure^b^	85	99	266/1.65
PID 8/Day 1	100	100	291/—
PID 8/Failure^b^	84	99	223/0.77
PID 11/Day 1	100	99	36.0/—
PID 11/Failure^b^	80	99	31.6/0.88

^a^Fold-change relative to JRCSF reference virus/relative to Day 1.

^b^Optimized background therapy included enfuvirtide.

## Discussion

The MOTIVATE 1 and 2 studies provided a unique opportunity to examine the viral changes associated with failure in highly treatment-experienced participants receiving a maraviroc-containing regimen. Many received sub-optimal background regimens as they had limited remaining treatment options due to previous exposure to multiple regimens and development of resistance [25,26]. In many instances, maraviroc was the only new active antiretroviral drug in the regimen [28], resulting in exposure to maraviroc functional monotherapy for prolonged periods of time in the presence of replicating virus, thus creating the ideal combination of circumstances for viral escape and the development of resistance [29].

For the MOTIVATE studies and other early maraviroc studies, the original Trofile assay [30] was used to determine tropism status pre-treatment. The original Trofile assay was validated to detect minor variants of CXCR4-using HIV in a population at approximately 5%-10% [30]. This assay was later superseded by an enhanced sensitivity version of the assay (ESTA), able to detect lower levels of minority CXCR4-using variants (~0.3% minor CXCR4-using variants) [31]. In retrospect, the results of the MOTIVATE and other CCR5-antagonist trials demonstrated that a tropism assay with increased sensitivity relative to the original Trofile assay was required for optimal clinical efficacy [32,33]; indeed, the ESTA was developed in response to disappointing results from early clinical trials of CCR5 antagonists. The most common virologic observation at treatment failure was the detection of CXCR4-using virus; however, reanalysis of the screening samples using ESTA demonstrated the presence of DM virus prior to maraviroc treatment in 16/20 (80%) participants studied with CXCR4-using virus at failure. These participants would have been excluded from the study had ESTA been available and used at Screening in the MOTIVATE studies. Indeed, in more recent clinical studies where the ESTA assay was used for screening, few virologic failure individuals were found with detectable CXCR4-using virus [34–36].

In the phase 2a maraviroc monotherapy studies, A4001007 and A4001015, CXCR4-using virus was detected in 2 of 62 participants after 10 days of treatment [21]. Phylogenetic analysis of pre- and post-treatment samples indicated that low levels of CXCR4-using virus pre-existed and the inhibition of the R5 virus by maraviroc resulted in detection of the CXCR4-using variants. This preliminary observation is extended here to 20 individuals (16 receiving maraviroc) treated with combination antiretroviral therapy over 24 weeks. In the majority, there was evidence of CXCR4-using virus present pre-treatment using clonal analysis. In the remaining participants’ phylogenetic analyses, the on-treatment CXCR4-using virus was shown to be highly unlikely to have originated from an R5 precursor present upon starting treatment, regardless of having received maraviroc or placebo. In instances when there were no Day 1 CXCR4-using clones detected in plasma, the prevalence may have been very low prior to treatment. Studies of tropism using ultra-deep sequencing have also confirmed the emergence of pre-existing virus as the only source of CXCR4-using virus on failure with maraviroc [22,37,38]. In addition, X4 and R5 viruses show distinct lineages [22] and a complex scenario of multiple CXCR4-using viral emergence may occur even in the absence of treatment [3]. Altogether, these results are consistent with CXCR4-using virus detection on-treatment being due to revealing or unmasking of its presence relative to the diminished R5 virus population during maraviroc treatment [21] and not as a result of on-going maraviroc selective pressure and change within the R5 component of the infection. These findings are important, firstly, as they indicate that pre-screening with an adequately sensitive tropism assay can predict the likelihood of sustained maraviroc activity. Secondly, the mechanism for detection of CXCR4-using virus during maraviroc treatment was due in large part to the inhibition of R5 virus enhancing the proportion of CXCR4-using virus in the viral population. The evolutionary distinct lineage of exclusively R5-tropic and CXCR4-using variants also suggests that maraviroc has relatively little capacity to influence CXCR4-using viral evolution.

This is further supported by the observation of relatively poor fitness of the CXCR4-using virus, with frequent observation of reversion to an infection dominated by R5 virus after maraviroc treatment was stopped. Many of the Day 1 clones identified as CXCR4-using with genotypic assays showed poor infectivity *in vitro* and were often considered to be non-functional in the Trofile assay. Indeed, CXCR4-using virus is generally understood to be at a disadvantage relative to R5 virus in infected persons; CXCR4-using virus is rarely transmitted (reviewed in [39]), but is usually observed only late in infection and then only in a proportion of individuals [40]. Under normal conditions (ie, without maraviroc treatment), it is suggested that outgrowth of CXCR4-using virus is affected by the relative availability of susceptible cells [41,42] and differential immune control [43,44].

The evolution of CXCR4-using virus has been associated previously with increased disease progression when it occurred in untreated or sub-optimally treated individuals during the natural course of infection [45,46]. However, the emergence of CXCR4-using virus in the absence of CCR5-antagonists is generally observed at later stages of the disease, when progression is expected and target availability is changing. Natural emergence of CXCR4-using virus is thus attributed to the relative success of this virus in response to the changes in availability of host cells late in the disease course, which is a consequence of disease progression [47]. Interestingly, the dominance of CXCR4-using virus during maraviroc treatment was reversed during follow-up when maraviroc was removed from the regimen. Furthermore, there was no detrimental virologic or immunologic outcome in these participants [25]. Reversion was also observed during post-treatment follow-up in the MERIT study [48], which is consistent with the CXCR4-using virus observed during maraviroc treatment having reduced fitness relative to R5 virus and becoming dominant due to the removal of R5 virus in the presence of maraviroc.

Consistent with maraviroc being an allosteric inhibitor of CCR5 binding, among participants who failed with R5 virus, a change in IC_50_ (the conventional measure of resistance with competitive inhibitors) has been observed rarely. Reduced MPI to maraviroc was the key indicator of viral resistance, which is consistent with the virus developing the ability to enter cells through maraviroc-bound CCR5 [49]. MPI reduction has been described previously for maraviroc [19] and for other CCR5 antagonists [50–52]. In all, approximately two-thirds of the R5 failures from the MOTIVATE studies retained susceptibility to maraviroc (MPI ≥ 95%). Poor compliance or an ineffective background regimen is likely a reason for some of these failures.

The mechanism for selection of ‘true’ resistance to maraviroc in R5 virus is for the virus to adapt to be able to bind to the CCR5 molecule with inhibitor present, with greater binding through the CCR5 N-terminal region in the absence of maraviroc [53]. However, the resistant virus becomes more reliant on CCR5 expression for infection [49], thus the development of resistance seems to impart a significant replicative disadvantage [53]. Indeed, in the present study, there was one instance of negative MPI (PID 3: –27%), albeit observed only with a cloned virus examined in the PBL assay. This finding is consistent with the interpretation that resistant R5 virus might become dependent for entry on maraviroc-bound CCR5 host protein to the extent of partial dependency [54].

While the overall molecular mechanism to attain HIV envelope binding to CCR5 appears to be the same for different maraviroc-associated resistance (ie, recognition of and binding to the compound-bound receptor by the mutated gp160 envelope glycoprotein), the exact genotypic changes required seem to be highly variant. Although changes in the V3-loop were observed on all resistant viruses in the present study, there was no consistent pattern of mutations; indeed, regions outside the V3-loop might be contributory in addition to compensatory [20]. Strikingly, the removal of 2 mutations in the V3-loop of 1 failure virus restored maraviroc susceptibility, yet their inclusion in the paired Day 1 virus did not give rise to resistance (PID 12), emphasizing the importance of the context around a mutation in the HIV-1 envelope.

A separate analysis has been performed using frozen plasma samples from 8 MOTIVATE participants who experienced treatment failure with R5 virus that had reduced MPI to maraviroc [55]. Four of these participants had samples included in this report, while the other 4 failed after the data cut for the current analysis (ie, the first 267 participants enrolled through the Week 24 time point). Between 2 and 8 env clones were amplified, cloned, and characterized from Day 1 and failure samples. Phenotypic susceptibility to maraviroc was characterized for these env clones using a 293-Affinofile system as well as NP2-CD4/CCR5 cells. Their findings support the observation that resistance to maraviroc involves varied mutational pathways in the V3-loops that are not predictable. In addition, they confirm a lack of cross-resistance to other CCR5 antagonists, with only sparse and apparently random cross-resistance to TAK-779 and vicriviroc that was not associated with the degree of maraviroc resistance.

The X-ray crystal structure of maraviroc-bound CCR5 has recently been solved, confirming that the compound binds deep into the bottom of the pocket formed by the helices of CCR5, and is consistent with it being an allosteric inhibitor of viral entry [15]. Furthermore, biochemical and receptor modeling data comparing the binding properties of different small molecule CCR5 antagonists indicate that whilst all compounds bind into a similar binding pocket to maraviroc, the effect of binding on receptor function is unique [56]. Watson and colleagues speculate that the compounds induce unique receptor conformations that may reduce the likelihood of cross-resistance. These findings possibly explain why cross-resistance to aplaviroc is only found in 1 maraviroc resistance virus from 1 participant in this study and, in this case, it is only marginal. It has also been reported that cross-resistance to vicriviroc did not occur in any of the maraviroc-resistant viruses derived from these participant samples using an alternative method [57].

In conclusion, extensive data from phenotypic and genotypic analysis of viral escape from inhibition by maraviroc is presented at a clonal level. The main virologic change observed in virologic failure samples was the detection of CXCR4-using virus. This was due in large part to unmasking of pre-existing CXCR4-using virus populations caused by R5 virus inhibition with maraviroc. The effect was reversible on stopping maraviroc treatment. Reduction of maraviroc susceptibility in R5-tropic virus was less frequent and associated with various changes in the env protein V3-loop, but without a common resistance pathway.

## Materials and methods

### Participant population

MOTIVATE 1 and 2 were randomized, double-blind, placebo-controlled, phase 3 trials conducted in Canada, the United States, Australia, and Europe to assess the safety and efficacy of maraviroc in combination with other antiretroviral drugs for the treatment of HIV-1 (ClinicalTrials.gov: NCT00098306 and NCT00098722). The studies have been described in detail elsewhere, but briefly, eligible participants had an R5 HIV-1 screening tropism result using the original Trofile^®^ assay (Monogram Biosciences, South San Francisco, CA, USA) [30] screening HIV-1 RNA >5000 copies/mL and ≥6 months treatment experience of—or documented resistance to—agents from at least 3 different antiretroviral classes (at least 2 agents for protease inhibitors). Participants were randomized to receive maraviroc once daily, maraviroc twice daily, or placebo (2:2:1), in combination with an investigator-selected OBT comprised of 3 to 6 antiretroviral drugs. Randomization was stratified by the use versus nonuse of enfuvirtide and plasma HIV-1 RNA level <100,000 copies/mL versus ≥100,000 copies/mL at screening. The OBT excluded agents undergoing investigation at the time (ie, darunavir, etravirine, raltegravir). Participants remained on blinded therapy until Week 48 with a planned interim analysis at Week 24 [25,26]. The study was designed by the sponsor, Pfizer Global Research and Development, with input from the study investigators. Both studies were conducted in compliance with the Declaration of Helsinki and International Conference on Harmonisation Good Clinical Practice Guidelines, and all local regulatory requirements were followed. The protocol was approved by institutional review boards/independent ethics committees at all sites and written informed consent was provided by all participants. The studies were scrutinized by an independent Data and Safety Monitoring Board.

Treatment failure was defined as 1 of 4 virologic endpoints confirmed by a second consecutive measurement within 14 days:

1. increase in HIV-1 RNA to ≥3 times the Day 1 level at or after Week 2;
2. decrease of <0.5 log_10_ copies/mL at or after Week 8;
3. decrease of <1.0 log_10_ copies/mL at or after Week 8, after a decrease of ≥2.0 log_10_ copies/mL; and
4. increase in HIV-1 RNA to ≥5000 copies/mL after a decrease to <400 copies/mL had been recorded on 2 consecutive visits.

Treatment failure was categorized as ‘early’ failure, occurring at or before Week 8, or ‘late’ failure, occurring after Week 8. Participants who did not experience treatment failure were categorized as responders.

### Selection of participants for virologic substudy

An exploratory analysis of tropism changes and maraviroc resistance was carried out on a subset of participants selected on a blinded basis using a pre-specified ~6-month period of enrollment that would give ~250 participants for analysis through Week 24. A data cut after all completers had reached Week 24 was performed. This allowed for a detailed observational analysis of the mechanisms of virologic escape from maraviroc including phenotypic and genotypic clonal analysis in order to provide a timeous assessment of mechanisms of virologic escape to inform clinical use of maraviroc.

### Selection of participants with CXCR4-using virus on-treatment

All participants selected for analysis were tested for tropism at treatment failure or Week 24. Those with CXCR4-using virus were subjected to random selection of 20 participants on a fully blinded basis. Participants were categorized by time to failure (≤Week 8, >Week 8, Week 24 completer with HIV-1 RNA >500 copies/mL). As there were only 6 participants with non-B viral infection failing with CXCR4-using virus, these were omitted from the analysis in case their findings might bias the analysis. A third party, which could be unblinded, was provided with participant identifier numbers in groups according to time to failure (ie, ≤Week 8, >Week 8 to <Week 24, Week 24) and Day 1 tropism. A random selection from each of the 6 groups was then made according to a set of pre-defined rules to ensure proportionate representation according to the number of participants in each group.

### Phenotypic determination of virus population co-receptor tropism

Co-receptor tropism in the MOTIVATE studies was determined using the original Trofile assay, as previously described [30]. Briefly, participant-derived gp160 sequences are amplified from plasma viral RNA and cloned into an envelope-expression vector as a population (library) of sequences. HIV virions carrying a luciferase reporter gene are pseudo-typed with participant-derived envelope (gp160) and used to infect CD4+ cell lines (U87CD4) expressing either CXCR4 or CCR5. Co-receptor usage is determined by the production of luciferase following a single round of viral replication in these cell lines. Specificity of co-receptor usage is demonstrated by inhibition of luciferase production by CXCR4 or CCR5 antagonists. The Trofile assay can detect 5%-10% prevalence of X4 virus [30], and results are reported as R5 (CCR5-tropic virus only detected), X4 (CXCR4-tropic virus only detected), DM (dual/mixed tropism, ie, a signal on both CCR5-expressing and CXCR4-expressing cells), DU (dual tropic virus as determined using cloned viruses), or NR/NP (not reportable/not phenotyped, ie, the test was canceled or the sample failed to give a valid tropism result). ‘CXCR4-using’ was provided as the genotypic read-out and used in relation to collective discussion of viruses with DM, DU, or X4 tropism. The output of the Trofile assay is a reflection of the relative sizes as well as the infectivity of the CCR5-using and CXCR4-using virus populations in the participant sample and is neither quantitative nor absolute.

A retrospective analysis of the samples was performed using the enhanced sensitivity Trofile assay (ESTA). This assay follows the same procedures as the original Trofile assay described above but has undergone further optimization to increase sensitivity to detect minor X4-tropic variants in the population when present with 0.3% prevalence [31].

### Clonal analysis of co-receptor tropism (infectivity assay)

Clones from paired pre-treatment (Day 1, pre-dose) and on-treatment samples from selected participants were isolated and pre-screened for co-receptor tropism using a high through-put infectivity assay. There are several differences between the Trofile assay used for clinical testing and the infectivity assay used to screen clones in this study. A key difference is the lack of confirmation of viral entry specificity using a co-receptor inhibitor. In addition, single-well, low-volume measurements and potential cross-talk between wells limit the ability of the infectivity to mirror the performance of the Trofile assay. Published information and a simple statistical model were used to decide how many clones to pre-screen for tropism (S4 Fig). Based on these calculations, it was estimated that approximately 180 clones per participant would need to be screened in order to be able to detect minor variants present at a 5% incidence with 99% probability, assuming that 50% would be non-functional. Each clone was tested in a single well of two 96-well plates: one containing CCR5-expressing U87CD4 cells and the other containing CXCR4-expressing U87CD4 cells. For each sample, the percentage of functional and non-functional clones was calculated as well as the percentage of functional clones that were either ‘R5,’ ‘X4,’ or ‘Dual.’

A region including the V3-loop was sequenced for all clones (Monogram Biosciences, San Francisco, CA, USA) and the sequences aligned using the CLUSTAL_X program [58]. The V3-loop sequence was extracted and tropism was predicted from the V3-loop sequences using the Geno2Pheno algorithm (FPR 10%; http://coreceptor.geno2pheno.org/index.php, accessed February-April 2013). The consensus sequences were calculated using >80% identity. Less than 80% was represented by ‘X.’

A further confirmatory re-analysis of tropism was performed using the clinical version of the original Trofile assay. This analysis included 12 clones each per paired Day 1 and on-treatment samples. Samples were selected such that any clones with discordance between genotypic and phenotypic findings from the infectivity assay were included where possible.

### Phylogenetic analysis

DNA sequences of minimum length 280 nucleotides from each env clone were used to infer evolutionary trees. Reference sequences from unrelated individuals were included to root the trees. For each participant, 7 reference sequences were also included in the alignment:

- The reference HIV-1 strain, HXB2 (NCBI accession number K03455), was used as an out-group (ie, an evolutionarily divergent sequence) to root the phylogenetic trees.
- In addition, an R5 env clone and CXCR4-using env clone from 3 other participants were used to represent examples of clade B viruses currently in circulation.

The nucleotide sequences of interest and the seven reference sequences were aligned using the CLUSTAL_X program [58] and default multiple alignment parameters. The alignment was then edited manually to adjust any gapped residues if appropriate and to excise any poorly aligned regions that would potentially cause bias. Phylogenetic trees were inferred using neighbor-joining and maximum likelihood with CLUSTAL_X and PAUP version 4 [59], respectively. For the maximum likelihood trees, the HKY+gamma model of nucleotide substitution and NNI heuristic were used, with a neighbor joining tree start tree (using the HKY model). Sites including indels were excluded. The CLUSTAL_X program was used to compute the neighbor-joining trees with the following parameters: ‘correct for multiple substitutions’ and standard bootstrapping with 100 replicates. The BOOTSTRAP N-J TREE parameter uses a method for deriving confidence values for the groupings in a tree (first adapted for trees by Felsenstein, 1985) [60]. It involves making N random samples of sites from the alignment (in this case, N = 100) and counting how many times each grouping from the original tree occurs in the sample trees. Diagrammatic representations of neighbor-joining trees were generated using MEGA (http://www.megasoftware.net/home) to collapse large branches and enable visualization and annotation; bootstrap values ≥75 are shown.

### Determination of susceptibility to maraviroc

Virus susceptibility to maraviroc was determined using two different methods; the PhenoSense™ HIV Entry assay (Monogram Biosciences, San Francisco, CA, USA) and a PBL based assay, as previously described [10,19,21,30].

The PhenoSense HIV Entry assay is a modification of the Trofile assay. Maraviroc susceptibility is determined from dose-response plots of percent inhibition of viral replication versus drug concentration using the CCR5-expressing U87CD4+ cells. Pre-clinical studies have shown maraviroc resistance can be identified by plateaus in maximum percent inhibition (MPI) in the PhenoSense HIV entry assay [19]. Plateau heights measured in the assay have been found to be reproducible and stable over a wide range of virus inputs. In our exploratory analysis, the results of PhenoSense HIV entry assay were assessed using specific pre-determined criteria for the definition of maraviroc resistance: a maraviroc dose-response curve with an MPI <95%; a maraviroc dose response curve with a >1.95 FC in IC_50_ relative to the reference virus JRCSF (equivalent to the geometric mean IC_50_ FC ± 2 standard deviations for a panel of 100 antiretroviral treatment–experienced clinical isolates tested in the same assay); or a >2-FC in IC_50_ between virus taken at Day 1 and virus taken at treatment failure from the same participant.

For the PBL-based assay, participant-derived env genes from the vectors used in the PhenoSense HIV Entry assay were transferred into an NL4-3 vector that was transfected into HEK-293 cells to produce replication-competent virus able to infect PBL. Phytohemagglutinin-stimulated PBL (pooled from multiple donors) were acutely infected with the replication-competent virus and cultured in the presence or absence of maraviroc for 7 days before estimating the amount of replication in each well by measuring reverse transcriptase (RT) levels in the culture supernatant. The CCR5-tropic lab strain Ba-L was included in all PBL assays as a reference strain; maraviroc susceptibility was reported both as geometric mean IC_50_ FC relative to Ba-L and arithmetic mean percentages in MPI. A shift in IC_50_ FC was only deemed relevant if the dose-response curve showed no evidence of a plateau in MPI. The accuracy of the estimates of susceptibility to maraviroc (IC_50_ FC and MPI) in PBL assays was reported by calculating 95% confidence intervals. Thus, it was possible to distinguish between dose-response curves that reached close to 100% inhibition (but were not statistically different from 100%) from those curves that demonstrated a plateau with MPI<100%. In this way, MPI values <95% were considered to reflect a biologically significant reduction.

### Generation of molecular clones and SDM of the env gene

In order to identify key genotypic markers of maraviroc resistance, the V3-loop amino acid residues of 4 treatment failure maraviroc-resistant clones were back mutated to correspond to the genotype of their respective Day 1 env clones. Furthermore, the Day 1 clones were also mutated to the sequence of their respective maraviroc-resistant counterparts.

Plasmid DNA corresponding to the Day 1 and treatment failure env clones from the participants was provided by Monogram Biosciences, Inc. Each env gene was transferred to an appropriate shuttle vector (pSP72 or pSP73) prior to SDM using the Quick Change II XL Site Directed Mutagenesis Kit (Stratagene, Amsterdam, the Netherlands) and sequence specific oligonucleotide primers. DNA was isolated from bacterial cultures using QIAprep Spin Miniprep Kit (Qiagen) and sequencing was performed by Lark Technologies, Inc. Sequence analysis using Vector NTI software (InforMax Inc, Bethesda, MD, USA) confirmed the authenticity of the SDM sequences. In each case, the mutated sites were those that were consistently associated with the maraviroc-sensitive or maraviroc-resistant clones sequenced for each pair.

The mutated env clones were then tested for susceptibility to maraviroc, aplaviroc, and enfuvirtide by Monogram Biosciences, Inc, in the PhenoSense HIV-1 entry assay as described above.

### Statistical testing

Analysis of variance (ANOVA) was used to identify changes in maraviroc IC_50_ fold-change at failure and between treatment groups. The *P* value and mean difference with 95% confidence intervals are quoted.

### Nucleotide sequence accession numbers

The following HIV-1 envelope sequences have been submitted to GenBank:

Maraviroc-treated: KT452383-KT452406, KT452431 –KT452454, KT452263-KT452310, KT452479 –KT452502, KT452527 –KT452562, MG183941 –MG188267, MG244243-MG244246.

Placebo: KT452599-KT452622, KT452359-KT452382, KT452084-KT452107.

## Supporting information

S1 Checklist CONSORT checklist. (DOC)

S1 Fig CONSORT 2010 flow diagram. (PDF)

S2 Fig Diagrammatic representation of neighbor-joining trees from clonal analysis of the HIV-1 envelope region from 20 participants with CXCR4-using infection on treatment. Each tree is rooted using HXB2 (NCBI accession number K03455). Examples of 2 clonal sequences: R5 (Day 1) and CXCR4-using (on-treatment) from 3 unrelated individuals were included together with the patients’ Day 1 and on-treatment nucleotide sequences. CLUSTAL_X was used to infer the neighbor-joining tree and the diagrammatic representation was created using MEGA, collapsing the branches with the size of the triangle representative of the number of sequences in the branch and the bootstrap values from ‘100 trials’ added to the nodes. Only those values greater than 75 were included. Green triangles and lines represents sequences from R5 clones and blue triangles and lines those from CXCR4-using clones. If the branch contained a mix of R5 and confirmed CXCR4-using clones, then that branch and triangle are colored blue. Black lines represent non-functional clones where the tropism could not be determined phenotypically. Individual clones not clustering in the main lineages are represented by single lines. Sequence diversity is represented by horizontal distance.
(PDF)

S3 Fig Diagrammatic representation of maximum likelihood trees from clonal analysis of the HIV-1 envelope region from 20 participants with CXCR4-using infection on treatment. Each tree is rooted using HXB2 (NCBI accession number: K03455) and inferred using PAUP with the HKY + gamma model of nucleotide substitution and NNI heuristic settings. Day 1 clones are labeled D and in black font. On-treatment clones are labeled E and in blue font.
(PDF)

S4 Fig Virologic and immunologic analyses for 4 participants who received open-label maraviroc following failure. (A) Change in viral load (◊ dashed line) and CD4 (▪ solid line) for participant 8, who received maraviroc in combination with didanosine (ddI), tenofovir (TFV), lopinavir/r (LPV/r), and enfuvirtide (ENF) for 27 weeks (shaded area) and failed therapy at Week 24 (red vertical dotted line) with R5 virus. Regimen prior to Day 1: ddI, LPV/r, ENF. OBT from Day 1: ddI, TFV, LPV/r, ENF. Screening: ddIsens, TFVsens, LPV/rres, ENFres (V38A). Failure: ddIres, TFVres, LPV/rres, ENFres (V38A). (B) Change in viral load (◊ dashed line) and CD4 (▪ solid line) for participant 11, who received maraviroc in combination with emtricitabine (FTC), tenofovir (TFV), stavudine (d4T), and enfuvirtide (ENF) for 15 weeks (shaded area) and failed therapy at Week 8 (red vertical dotted line) with R5 virus. Regimen prior to Day 1: FTC, TFV, d4T, TPV/r, ENF. OBT from Day 1: FTC, TFV, d4T, RTV, ENF. Screening: FTCres, TFVsens, d4Tsens, RTVres, ENFres (N43D/N). Failure: FTCres, TFVsens, d4Tres, RTVres, ENFres (N43D/N). (C) Change in viral load (◊ dashed line) and CD4 (▪ solid line) for participant 12, who received maraviroc in combination with didanosine (ddI), tenofovir (TFV), amprenavir (AMP), and enfuvirtide (ENF) for 12 weeks (shaded area) and failed therapy at Week 8 (red vertical dotted line) with R5 virus. Open-label therapy with maraviroc commenced Week 21 and finished Week 60 (shaded area). Regimen prior to Day 1: ddI, TFV, AMP/r, ENF. OBT from Day 1: ddI, TFV, FPV/r, ENF. Screening: ddIres, TFVres, FPV/rres, ENFres (N43D). Failure: ddIres, TFVres, FPV/rres, ENFres (N34D). (D) Change in viral load (◊ dashed line) and CD4 (▪ solid line) for participant 16, who received placebo in combination with abacavir (ABC) and tenofovir (TFV) for 14 weeks and failed therapy at Week 8 (red vertical dotted line) with R5 virus. Open-label therapy with maraviroc commenced Week 24 and finished Week 60 (shaded area). Regimen prior to Day 1: 3TC, TFV, ATZ/r. OBT from Day 1: ABC, 3TC, TFV. Screening: ABCsens,* 3TCres, TFVsens.* Failure: ABCsens,* 3TCres, TFVsens.*. *Sensitive by phenotype but resistant by genotype.
(PDF)

S1 Table Demographic characteristics of 58 participants studied in this analysis together with the characteristics for all 267 participants included in the population subset and the full participant population in the MOTIVATE trials (N = 1049). (DOCX)

S2 Table Genotypic and phenotypic clonal analysis of virus from 20 participants with CXCR4-using infection on treatment: Participants whose CXCR4-using virus on-treatment was related to a component of the pre-treatment virus population and participants whose CXCR4-using virus on-treatment was not related to a component of the pre-treatment virus population. (DOCX)

S3 Table Genotypic and phenotypic analysis of 12 clones from virus pre- and post-treatment, from 20 participants with CXCR4-using infection on treatment: Participants whose CXCR4-using virus on-treatment was related to a component of the pre-treatment virus population and participants whose CXCR4-using virus on-treatment was not related to a component of the pre-treatment virus population. (DOCX)

S4 Table Summary of MVC IC_50_ FC and MPI for CCR5 tropic virus from 36 participants failing blinded therapy in the MOTIVATE trials. (DOCX)

S5 Table Genotypic and phenotypic analysis of 12 clones from virus pre- and post-treatment obtained from 5 maraviroc-treated participants failing with maraviroc-resistant R5 virus and 4 participants receiving placebo and failing with r5 virus. (DOCX)

S6 Table PhenoSense HIV entry and PBL assay results for maraviroc susceptibility (IC_50_ fold change and MPI) from individual viral clones. (DOCX)

S1 Protocol (PDF)
