# Supplementary material for: Clonal analysis of HIV-1 genotype and function associated with virologic failure in treatment-experienced persons receiving maraviroc: Results from the MOTIVATE phase 3 randomized, placebo-controlled trials
Source: PLoS One. 2018 Dec 26;13(12):e0204099. doi: 10.1371/journal.pone.0204099 (PMC6306210; doi:10.1371/journal.pone.0204099)
Supplement: S1 Fig — (PDF) [file pone.0204099.s002.pdf]

S1 Fig. Diagrammatic representation of neighbor-joining trees from clonal analysis of the HIV-1 envelope region from 20 patients with CXCR4-using infection on treatment.

Each tree is rooted using HXB2 (NCBI accession number K03455). Examples of 2 clonal sequences: R5 (Day 1) and CXCR4-using (on-treatment) from 3 unrelated individuals were included together with the patients Day 1 and on-treatment nucleotide sequences. CLUSTAL_X was used to infer the neighbor-joining tree and the diagrammatic representation was created using MEGA, collapsing the branches with the size of the triangle representative of the number of sequences in the branch and the bootstrap values from '100 trials' added to the nodes. Only those values greater than 75 were included. Green triangles and lines represents sequences from R5 clones and blue triangles and lines those from CXCR4-using clones. If the branch contained a mix of R5 and confirmed CXCR4-using clones then that branch and triangle are colored blue. Black lines represent non-functional clones where the tropism could not be determined phenotypically. Individual clones not clustering in the main lineages are represented by single lines. Sequence diversity is represented by horizontal distance.

PID T6
Neighbor-joining tree

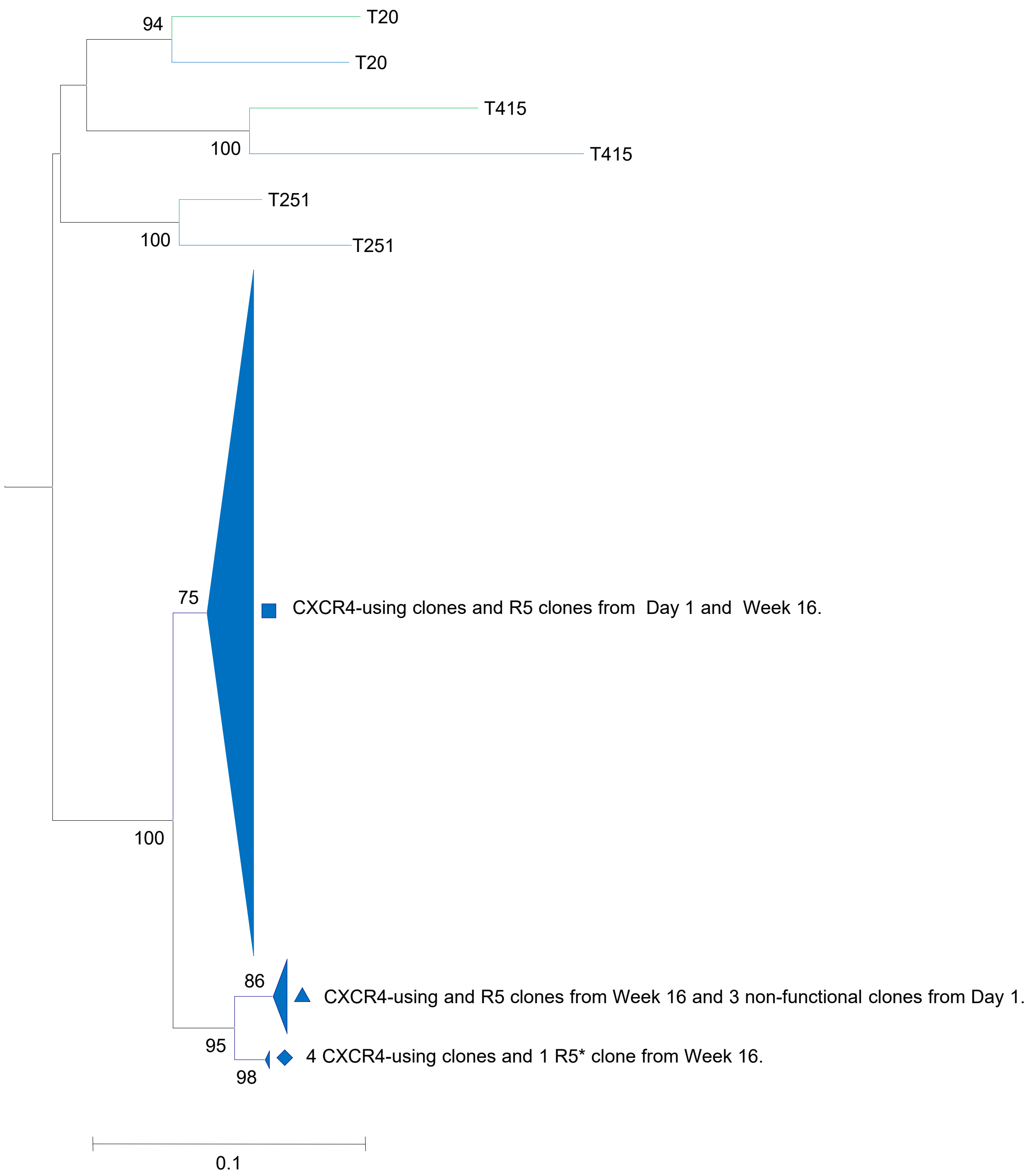

* This clone was non-functional in the Trofile assay.

PID T16
Neighbor-joining tree

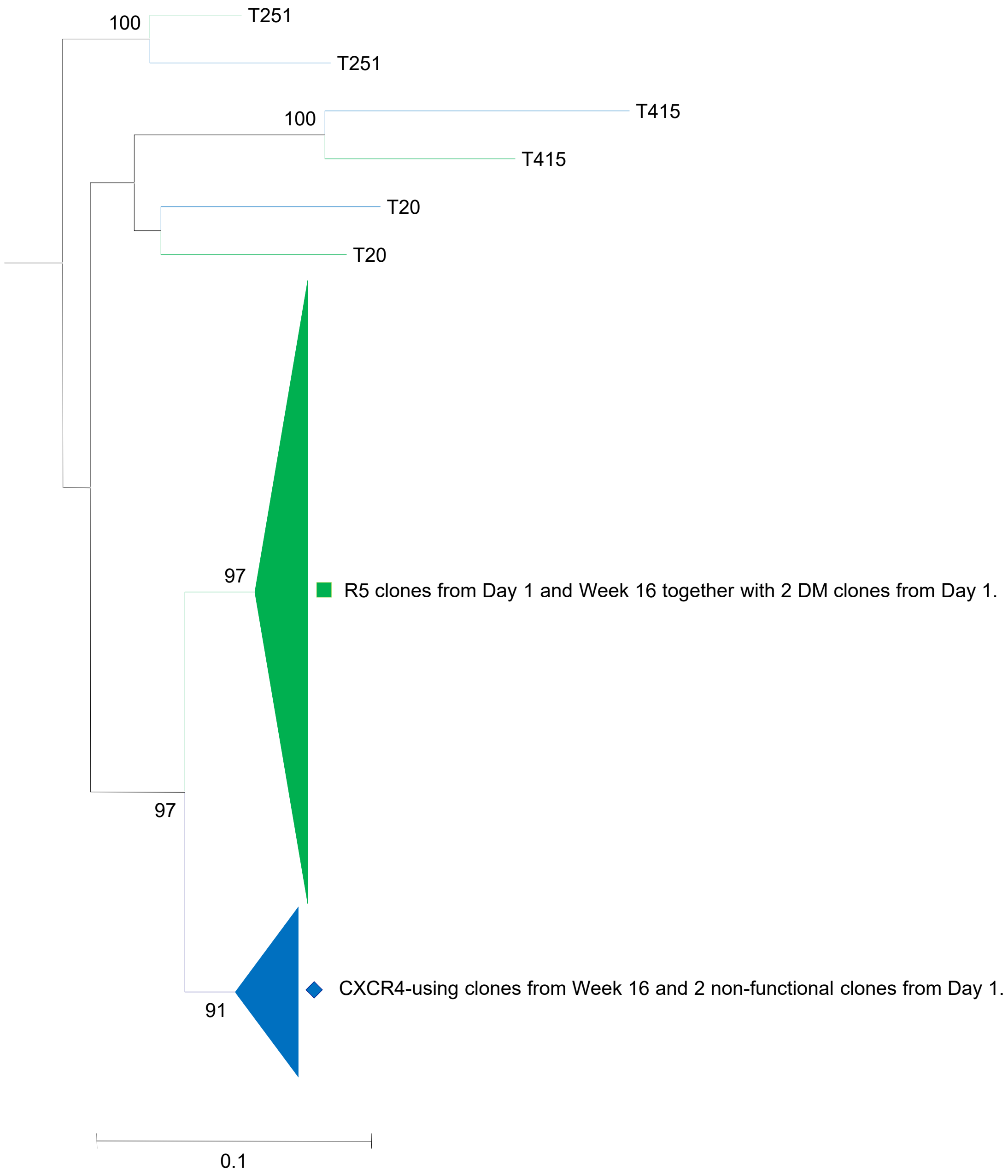

PID T17
Neighbor-joining tree

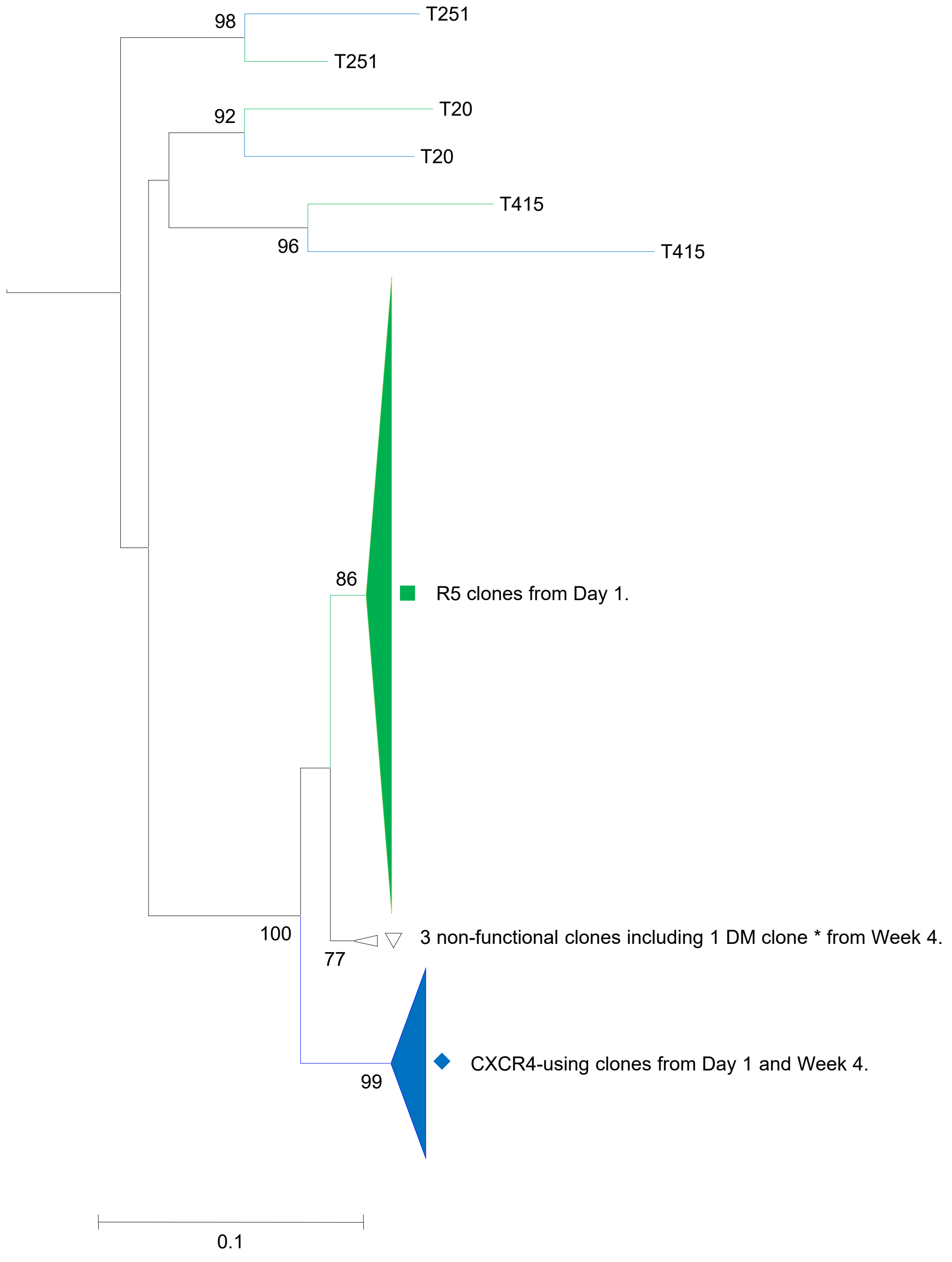

* This clone was non-functional in the Trofile assay.

PID T20
Neighbor-joining tree

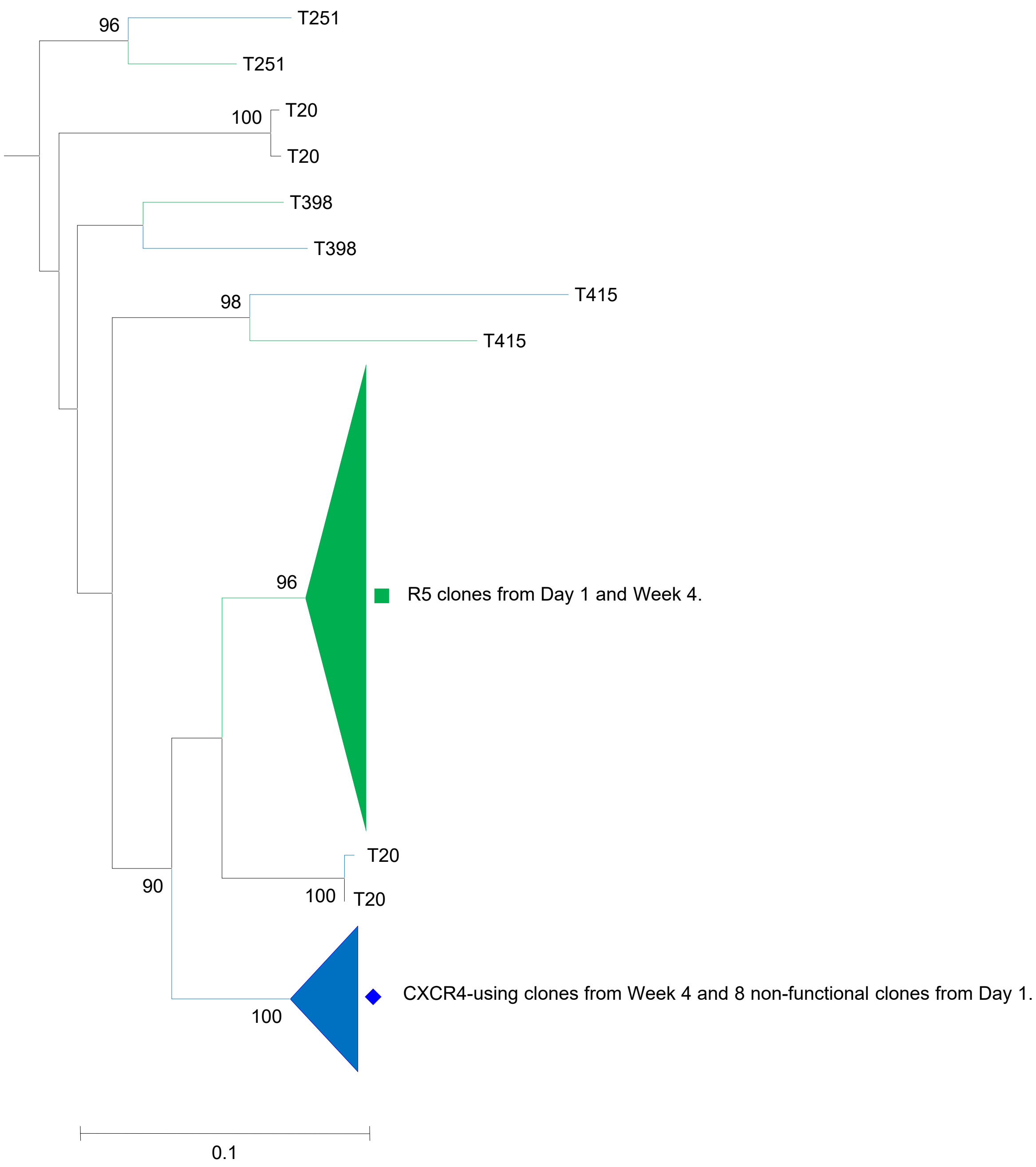

PID T69
Neighbor-joining tree

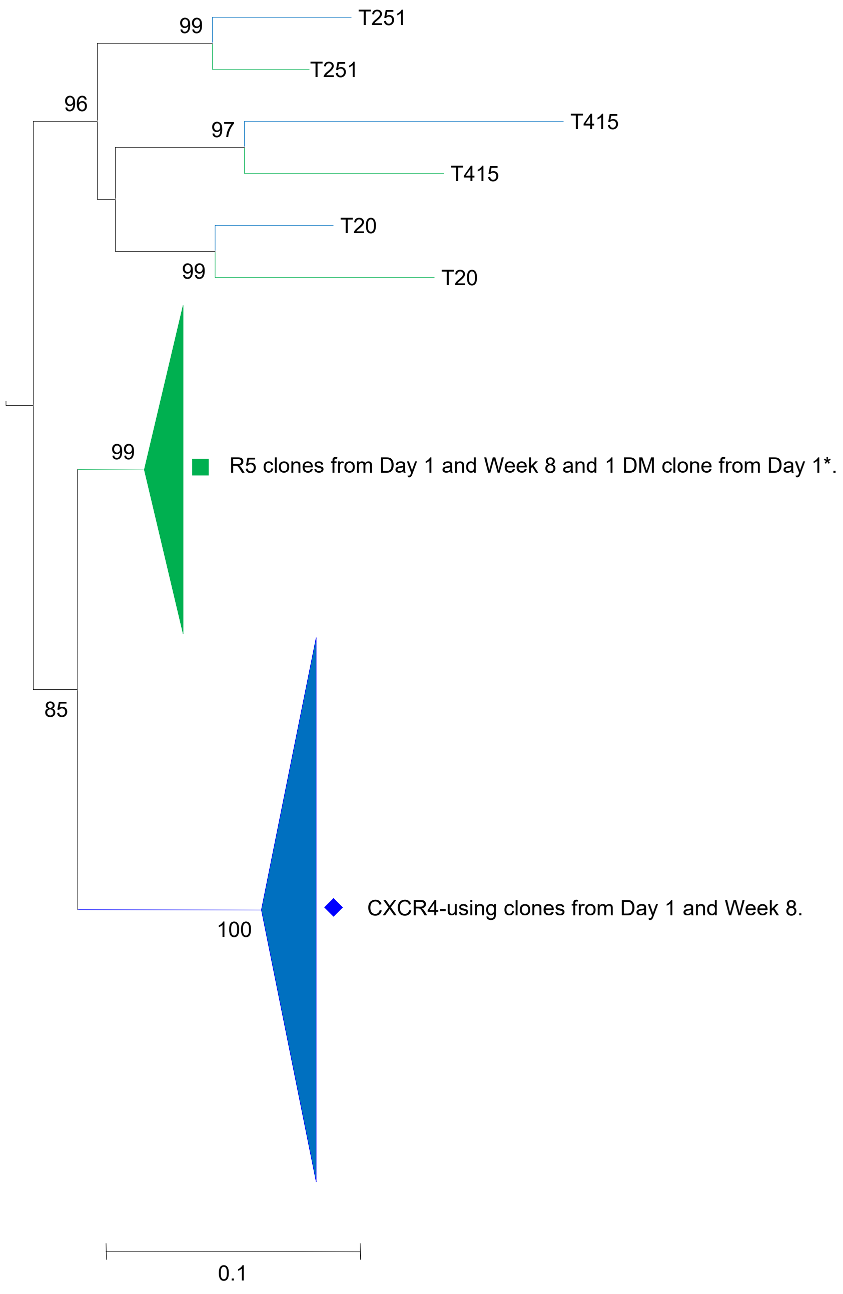

* Not tested in confirmational Trofile assay.

PID T132
Neighbor-joining tree

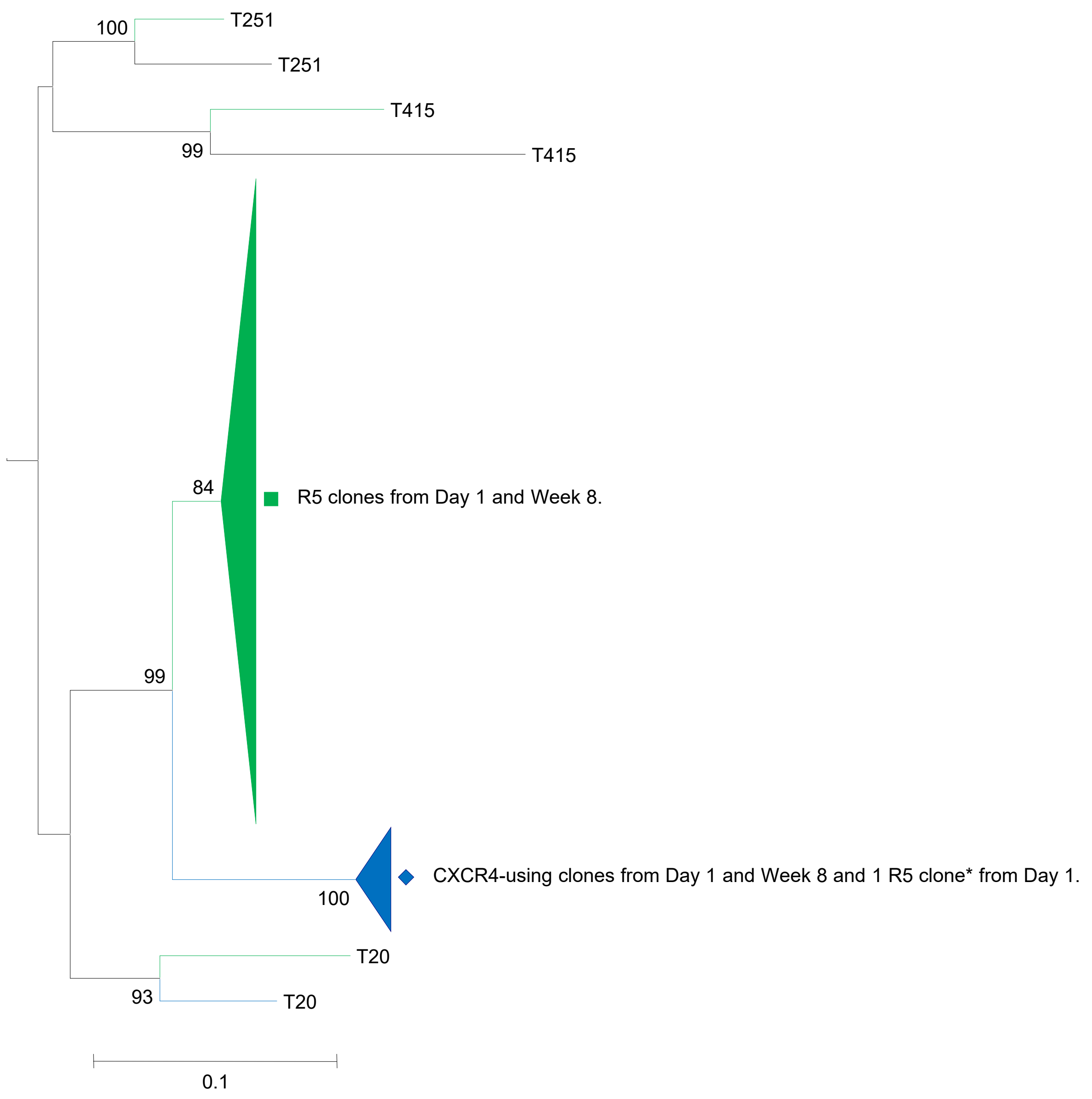

* This clone was confirmed in the Trofile assay.

PID T205
Neighbor-joining tree

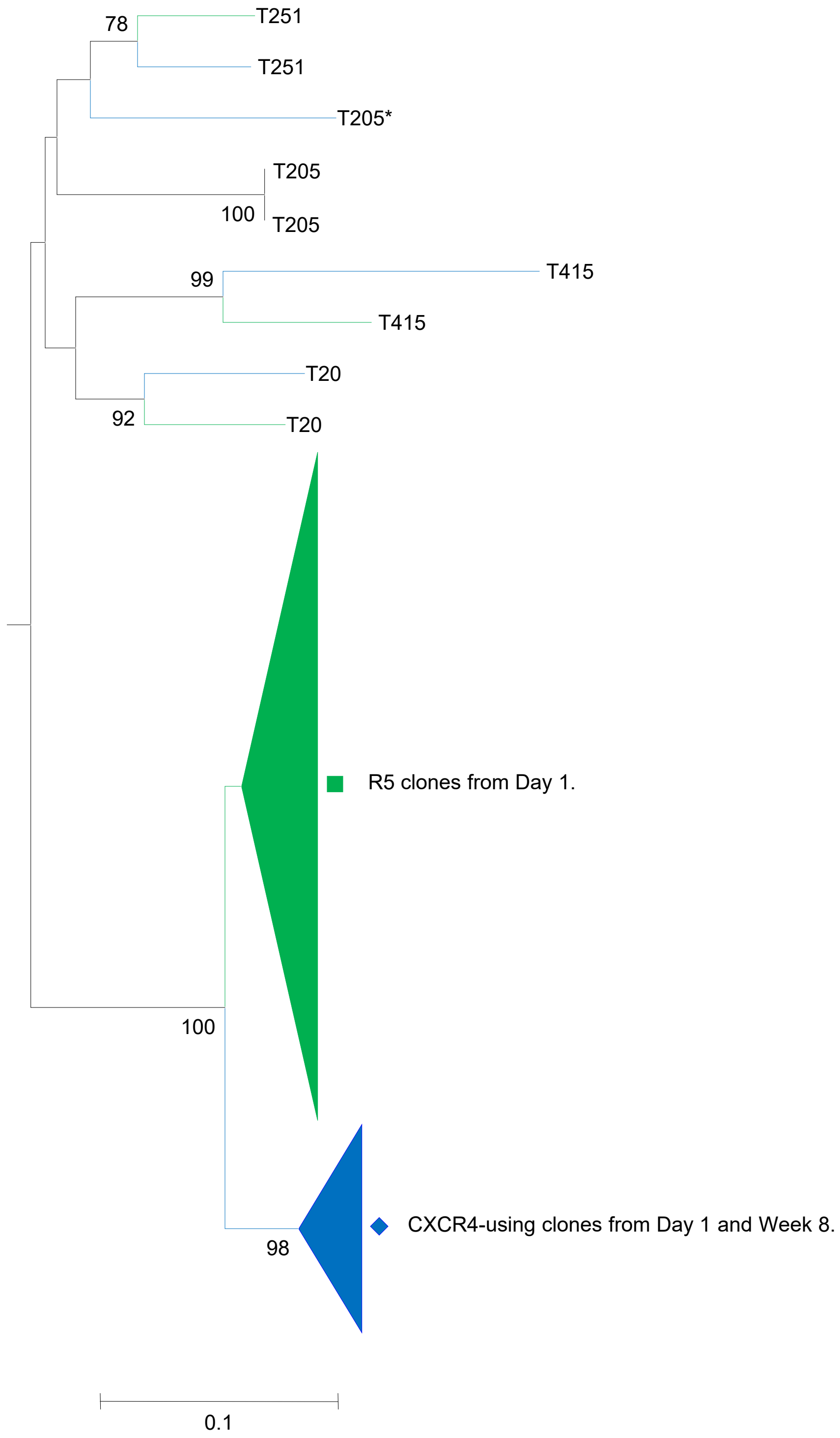

*This clone was non-functional in the Trofile assay.

PID T221
Neighbor-joining tree

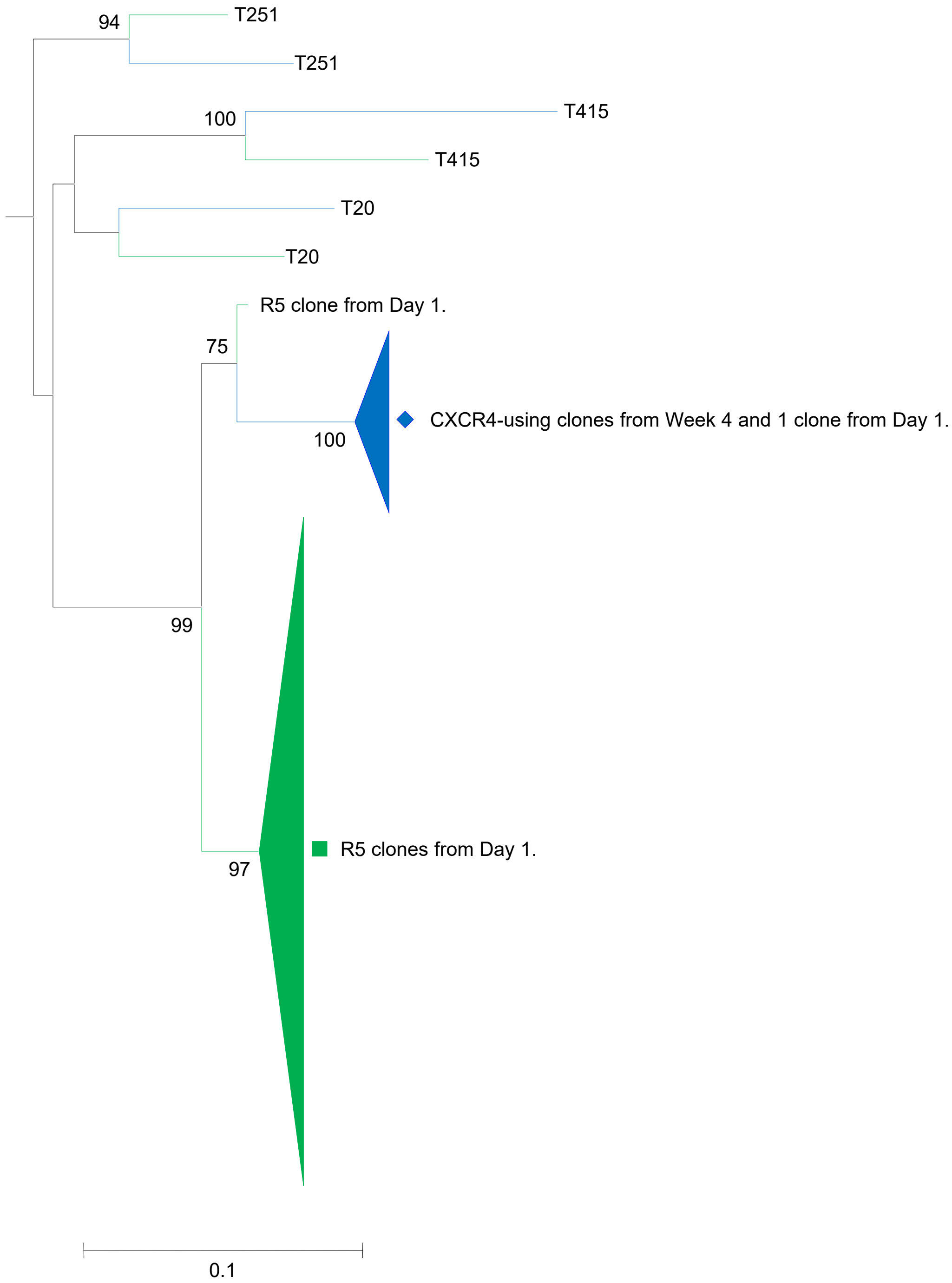

PID T246
Neighbor-joining tree

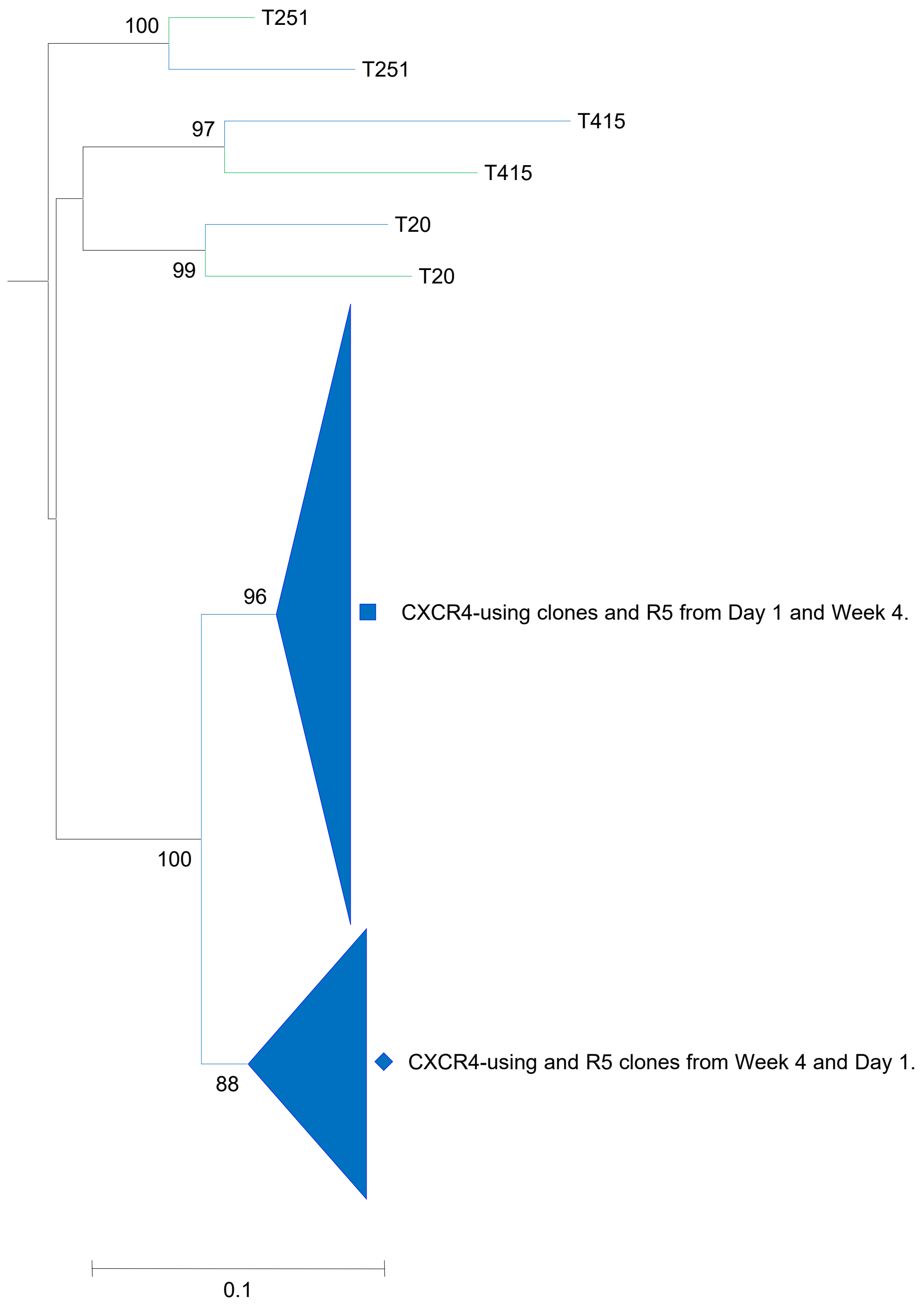

PID T251
Neighbor-joining tree

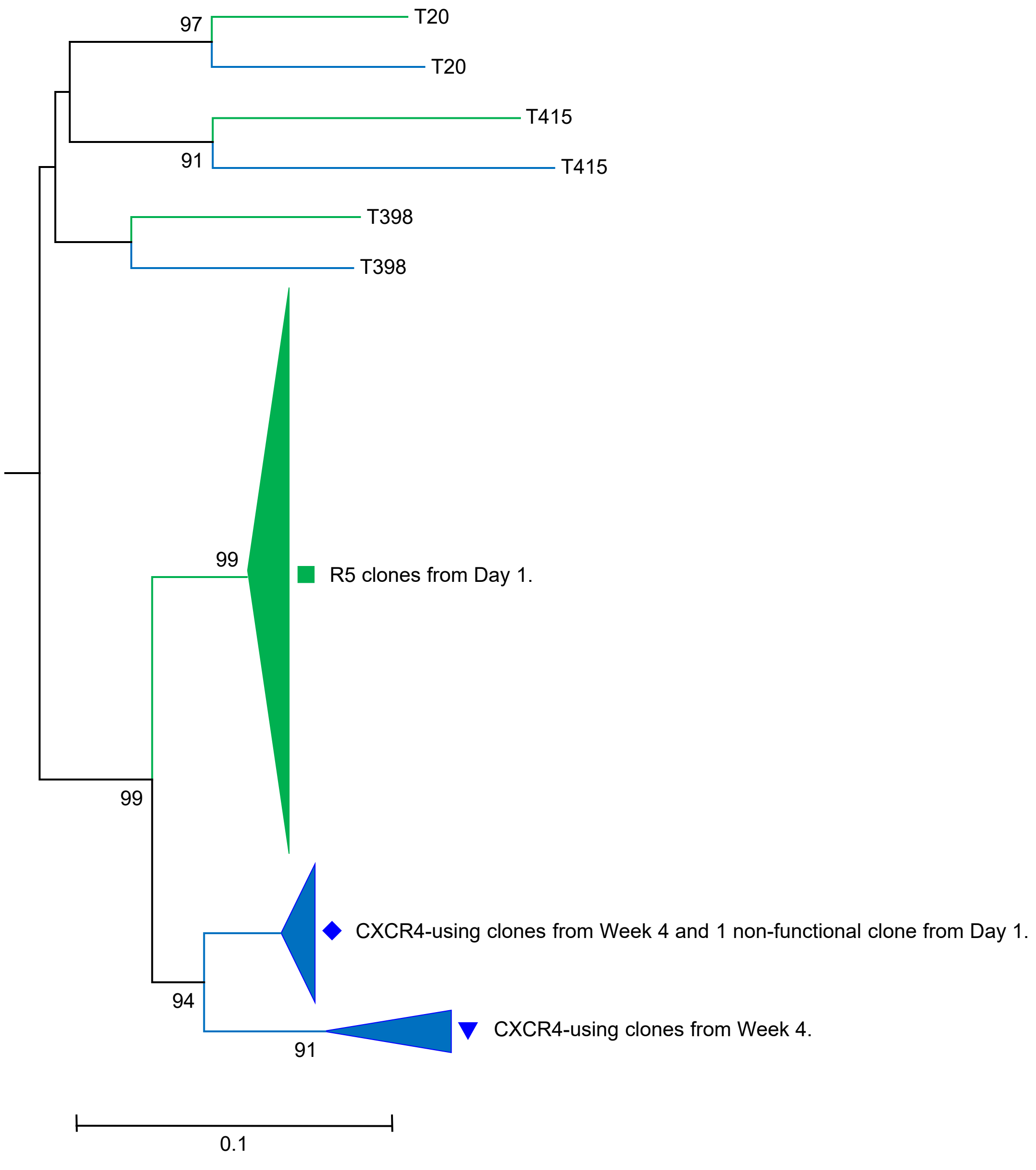

PID T285
Neighbor-joining tree

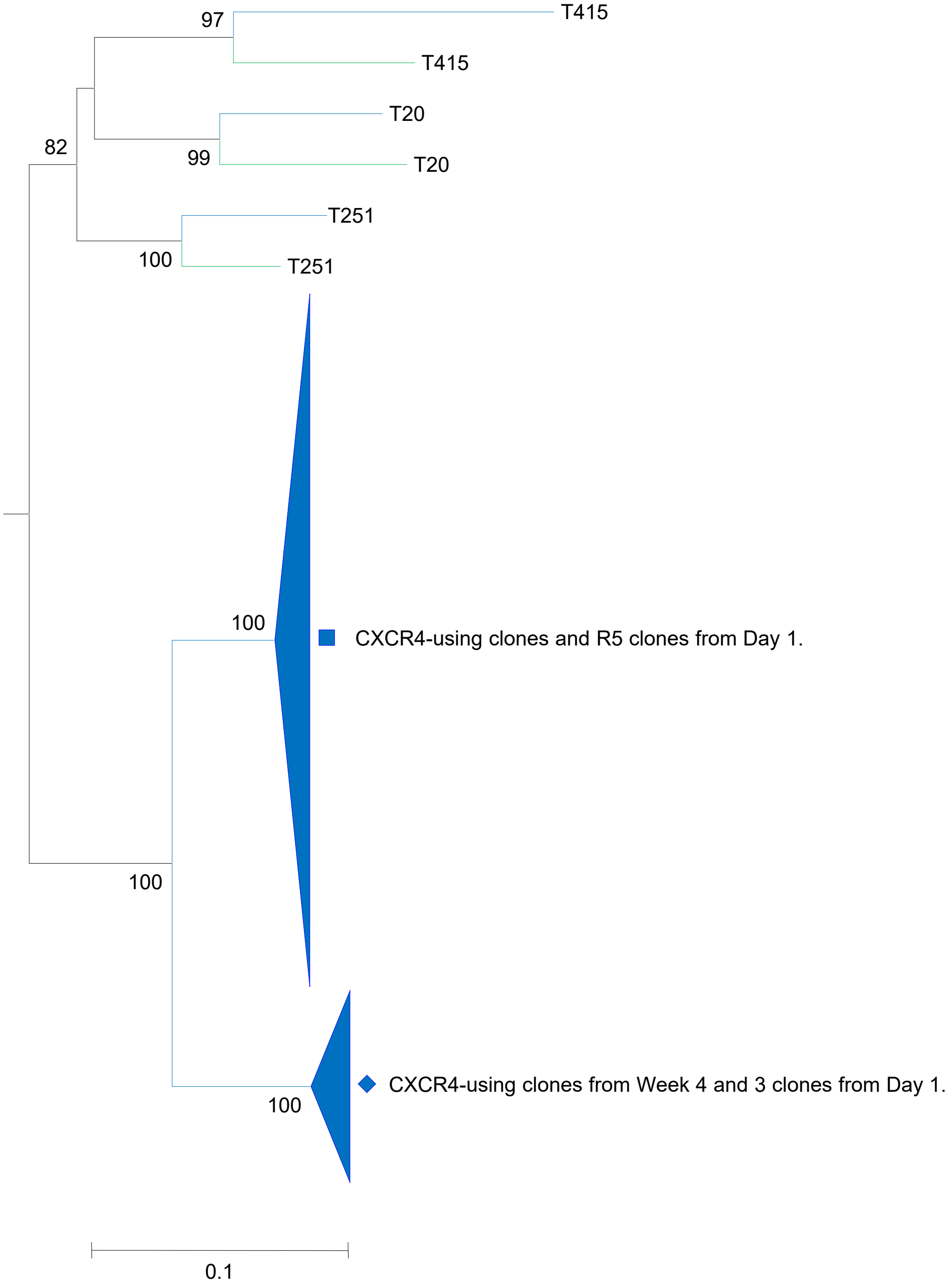

PID T347
Neighbor-joining tree

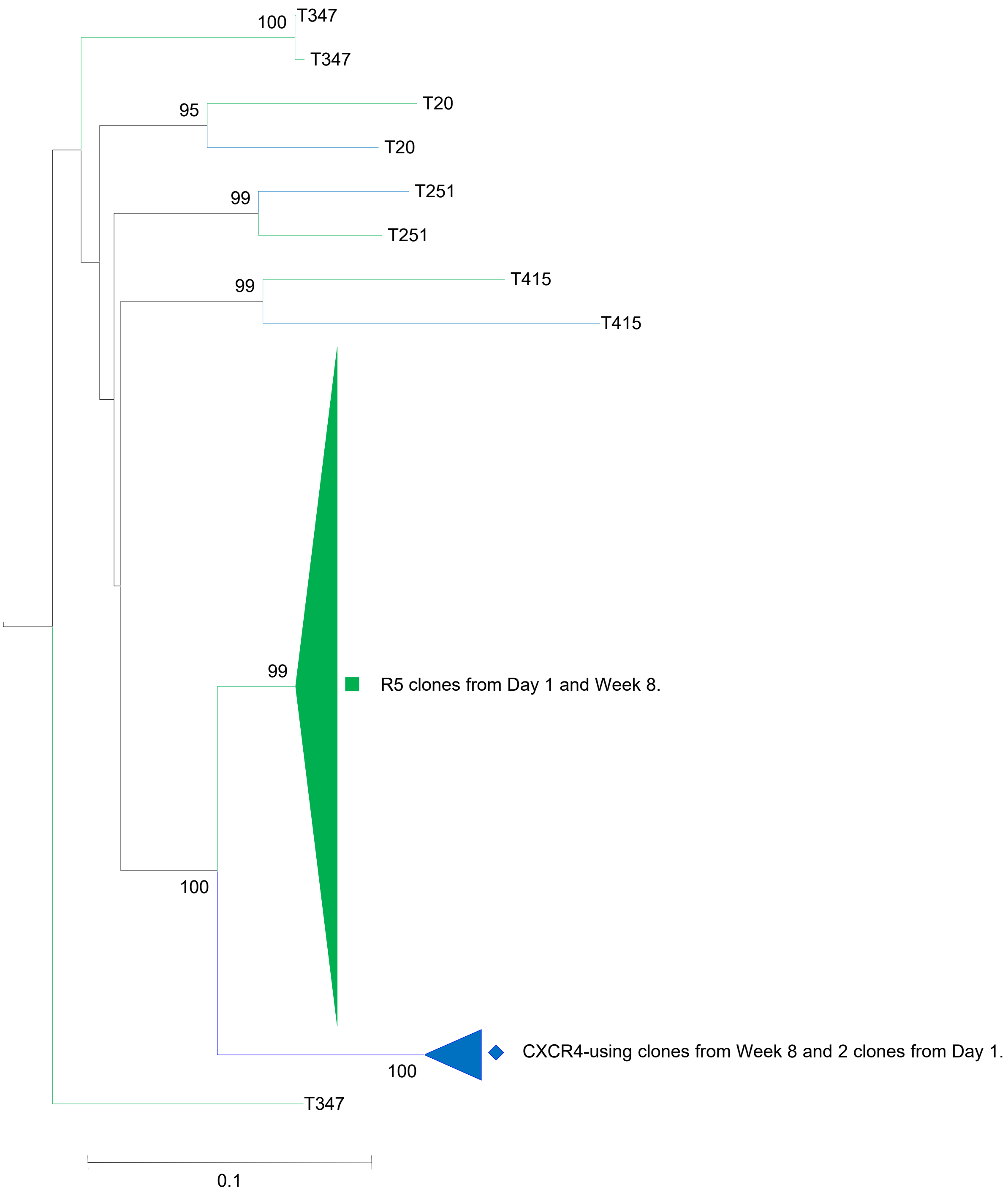

PID T377
Neighbor-joining tree

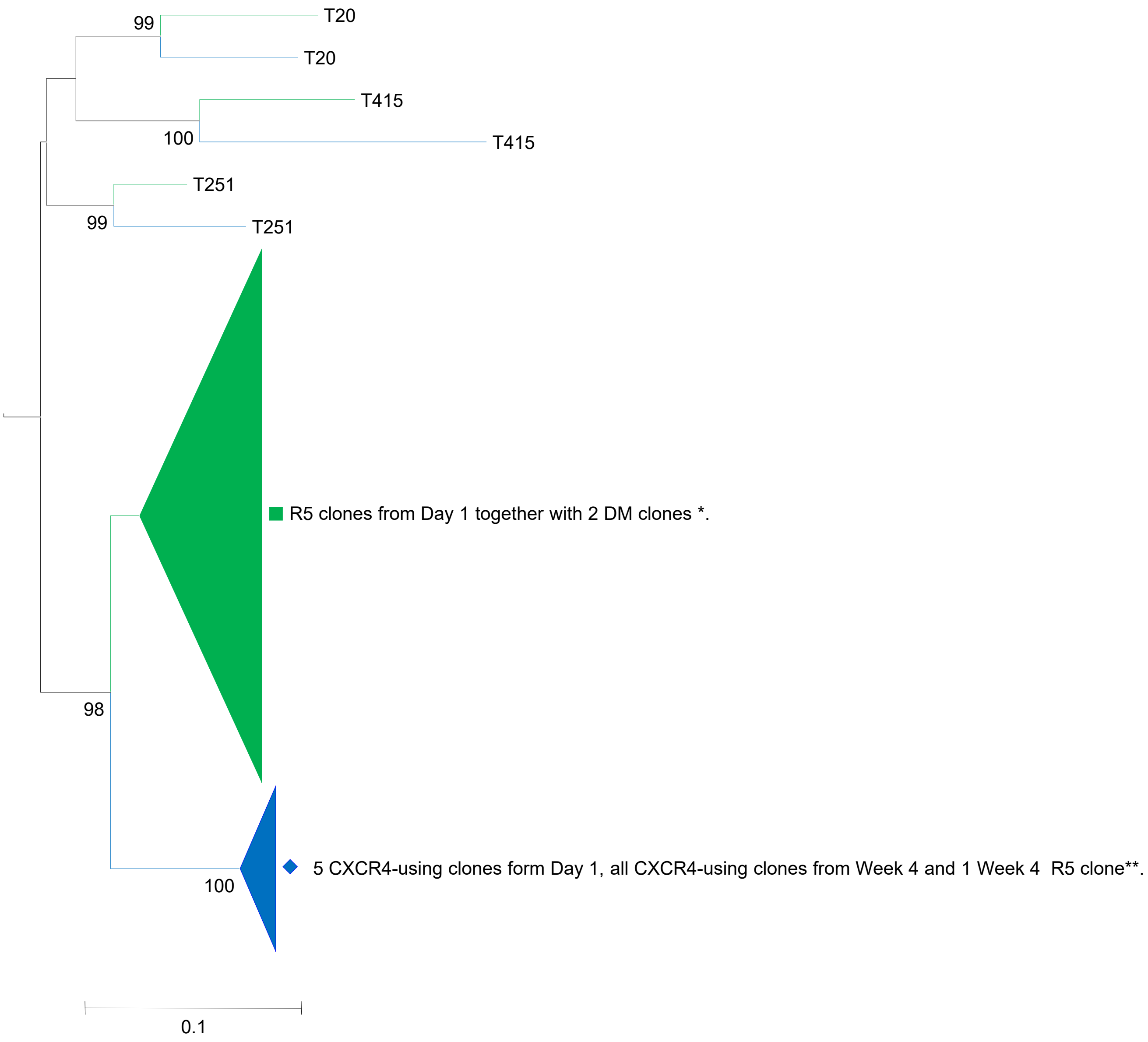

*These 2 clones were R5 in the Trofile assay.
**This clone was non-functional in the Trofile assay.

PID T397
Neighbor-joining tree

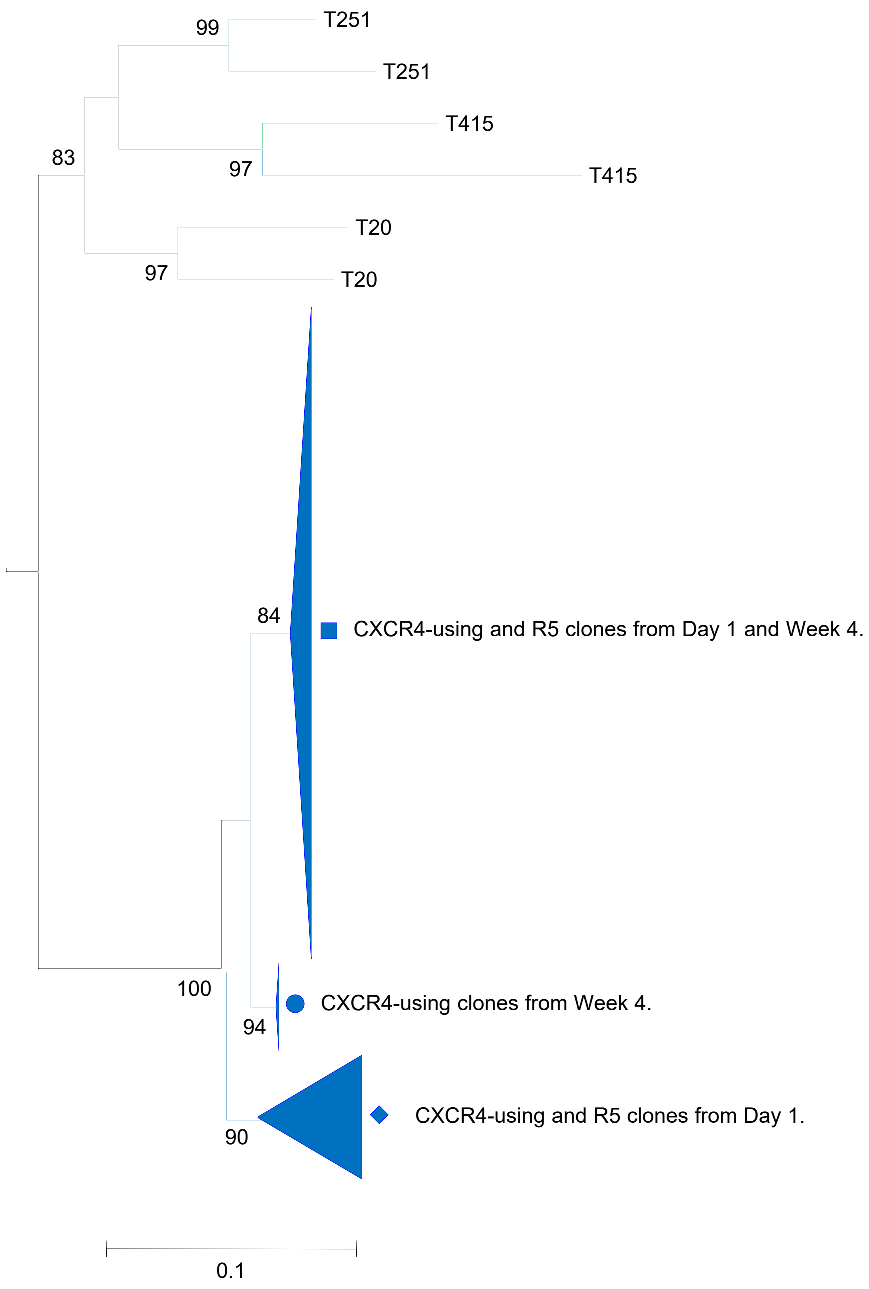

PID T57
Neighbor-joining tree

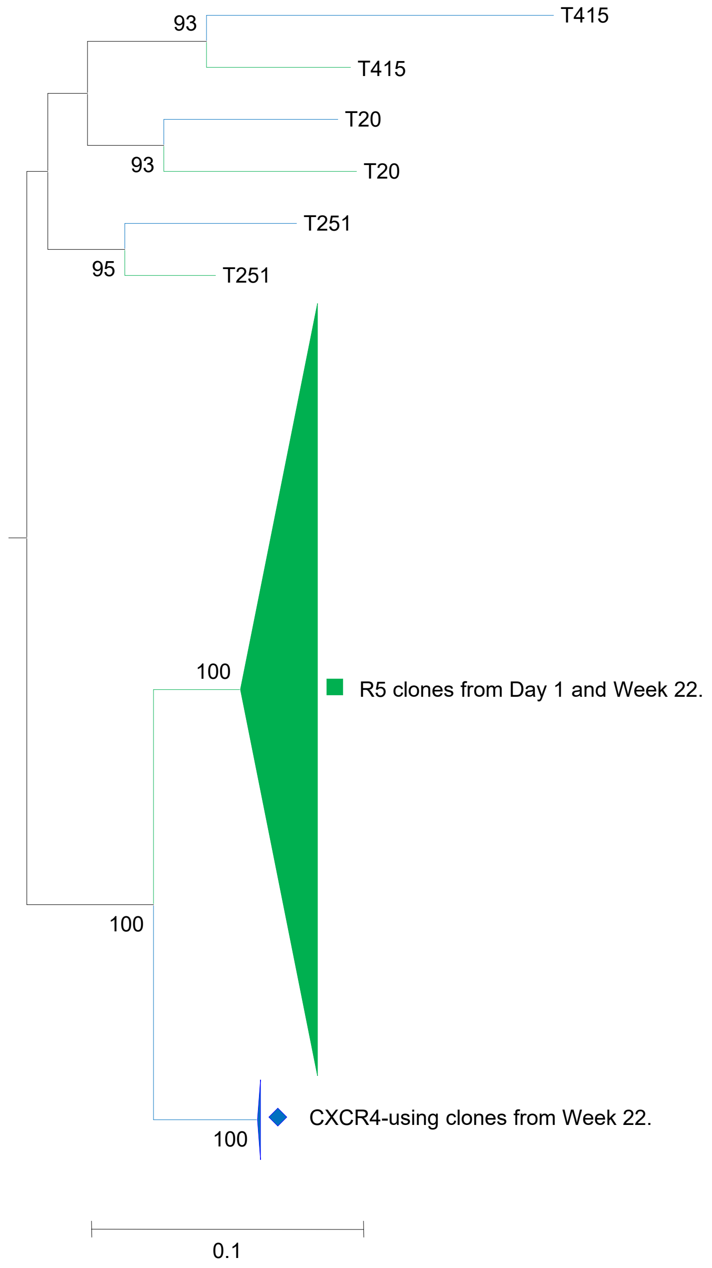

PID T210
Neighbour-joining tree

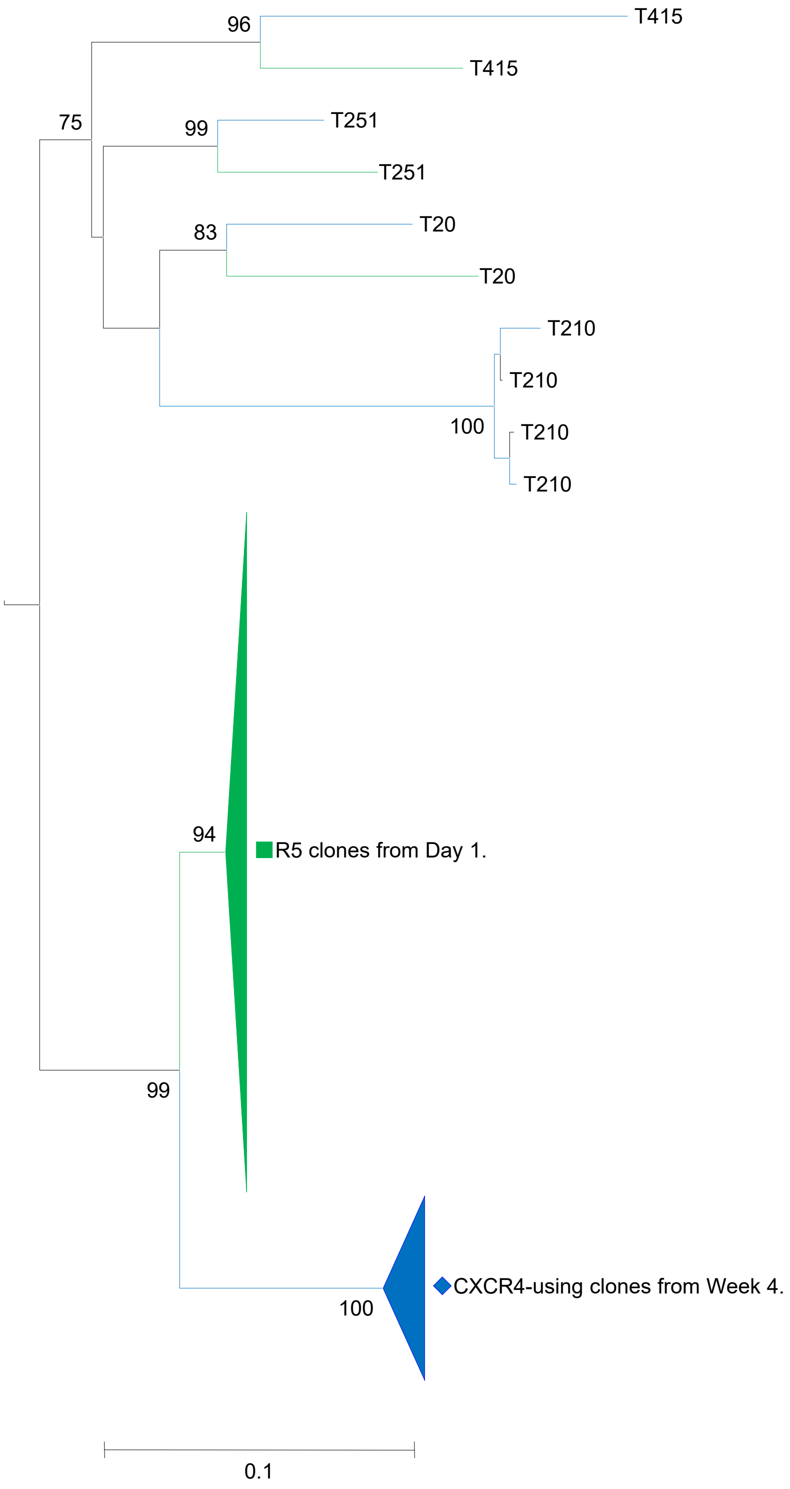

PID T398
Neighbor-joining tree

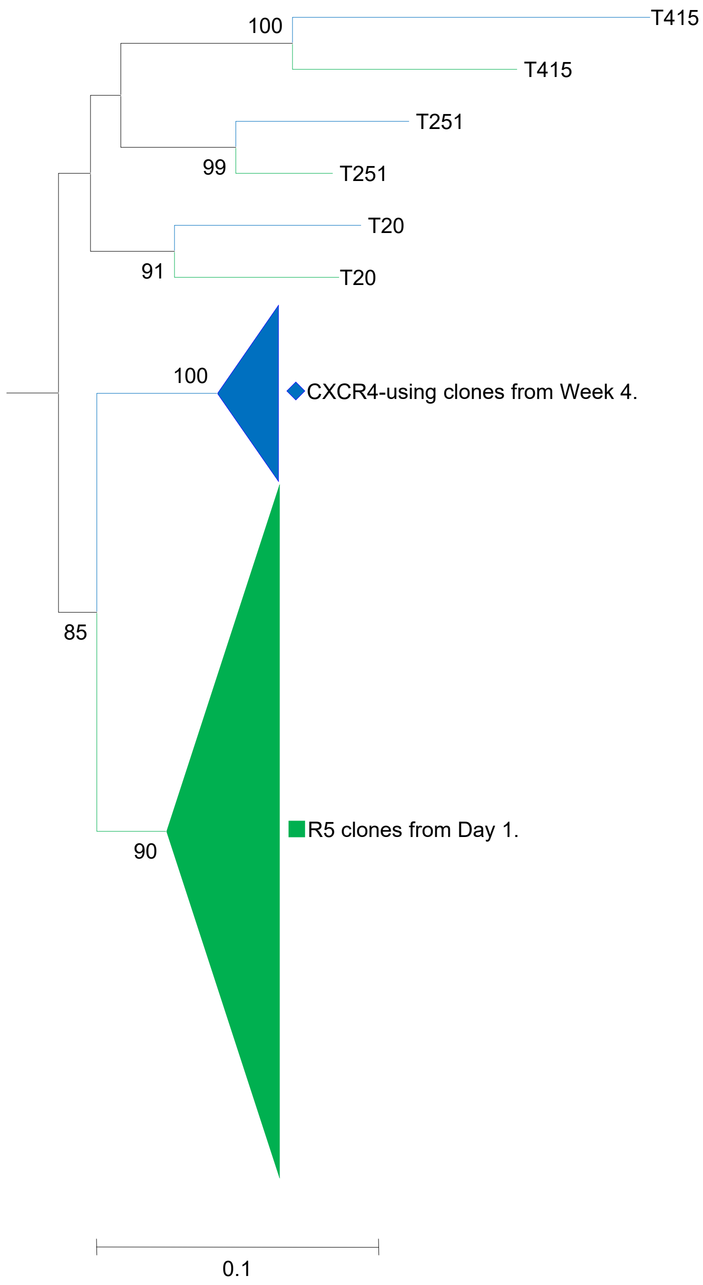

PID T415
Neighbor-joining tree

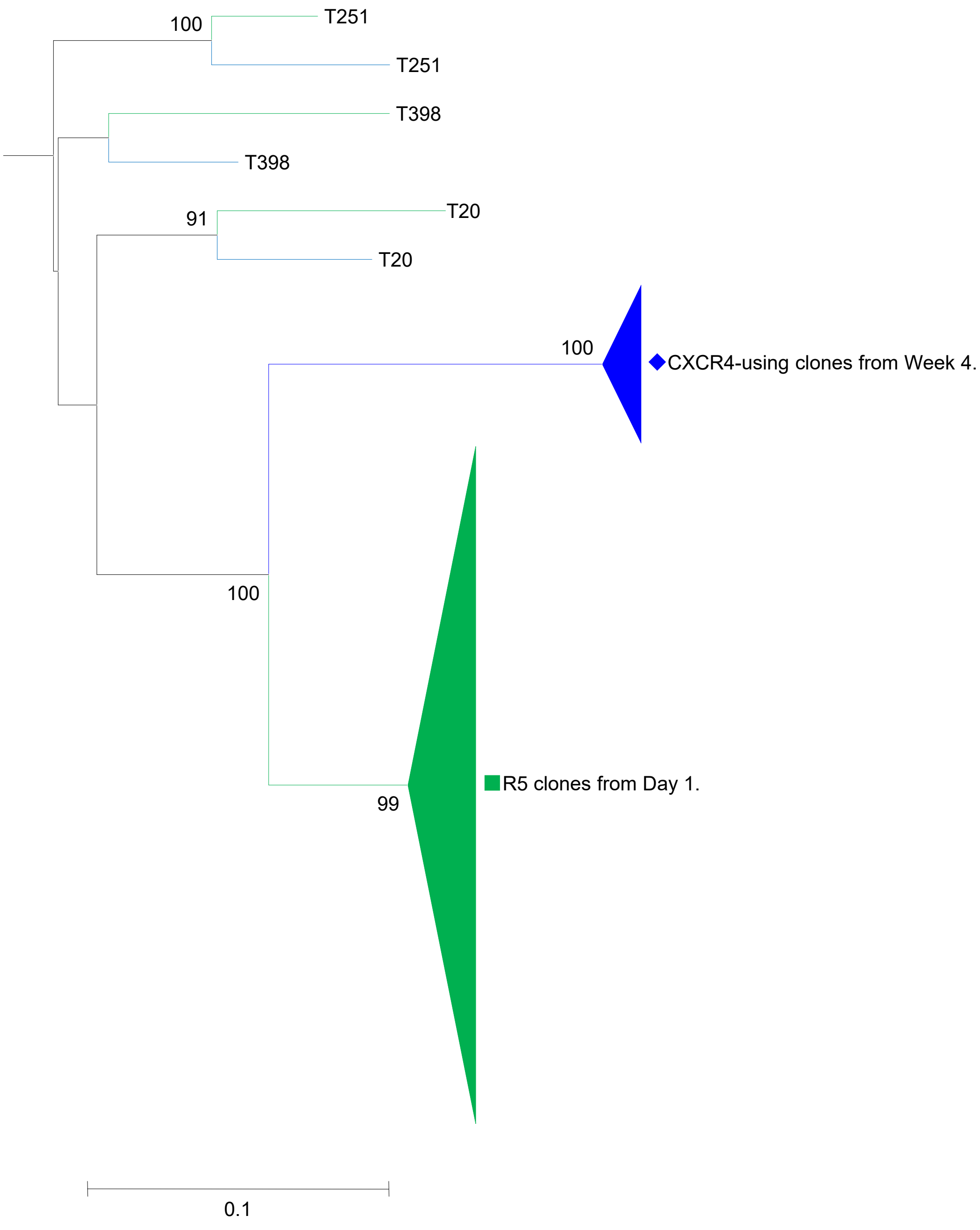

PID T629
Neighbor-joining tree

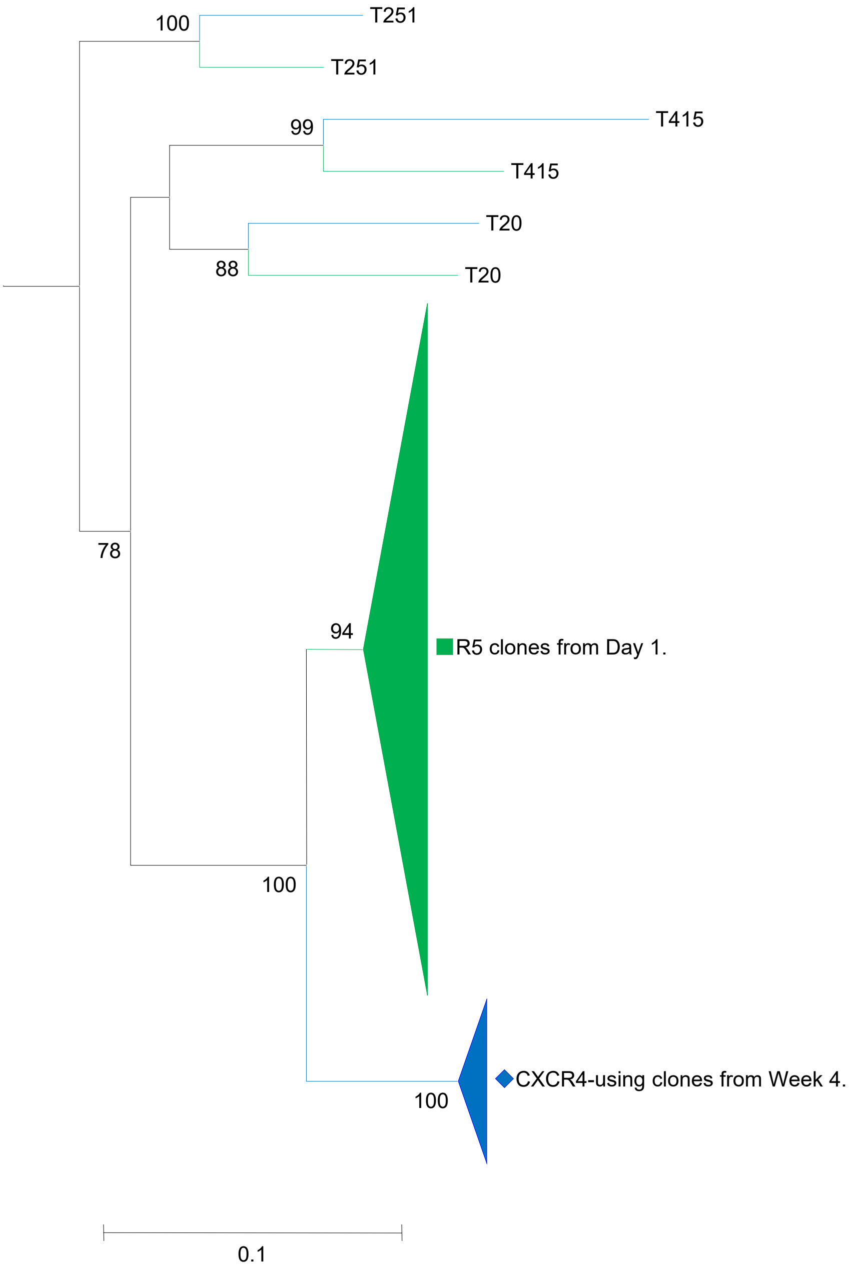

Patient T825
Neighbor-joining tree

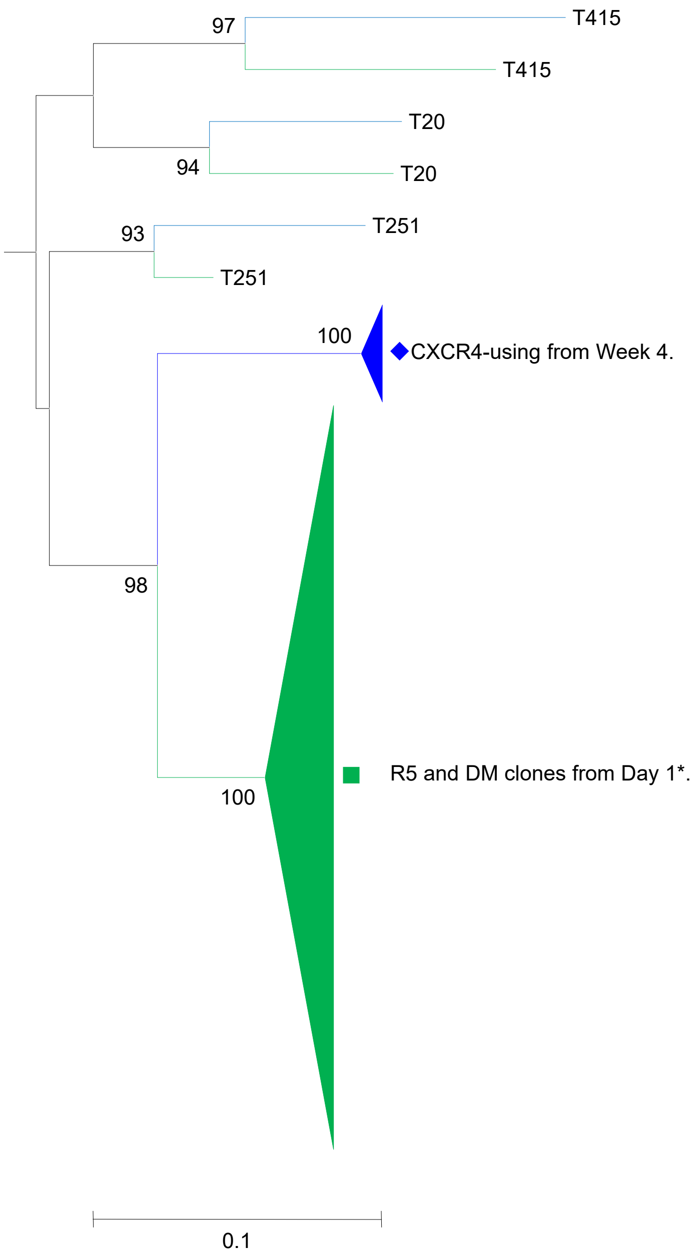

*The analysis demonstrated 29% of clones at Day 1 had weak DM charateristics of these 5 were tested in the Trofile assay and all 5 were found to show R5 tropism.
