# Supplementary material for: Clonal analysis of HIV-1 genotype and function associated with virologic failure in treatment-experienced persons receiving maraviroc: Results from the MOTIVATE phase 3 randomized, placebo-controlled trials
Source: PLoS One. 2018 Dec 26;13(12):e0204099. doi: 10.1371/journal.pone.0204099 (PMC6306210; doi:10.1371/journal.pone.0204099)

S2 Fig. Diagrammatic representation of maximum likelihood trees from clonal analysis of the HIV-1 envelope region from 20 patients with CXCR4-using infection on treatment.

Each tree is rooted using HXB2 (NCBI accession number K03455) and inferred using PAUP with the HKY+gamma model of nucleotide substitution and NNI heuristic settings. Day 1 clones are labeled D and in black font. On-treatment clones are labeled E and in blue font.

Patient T6

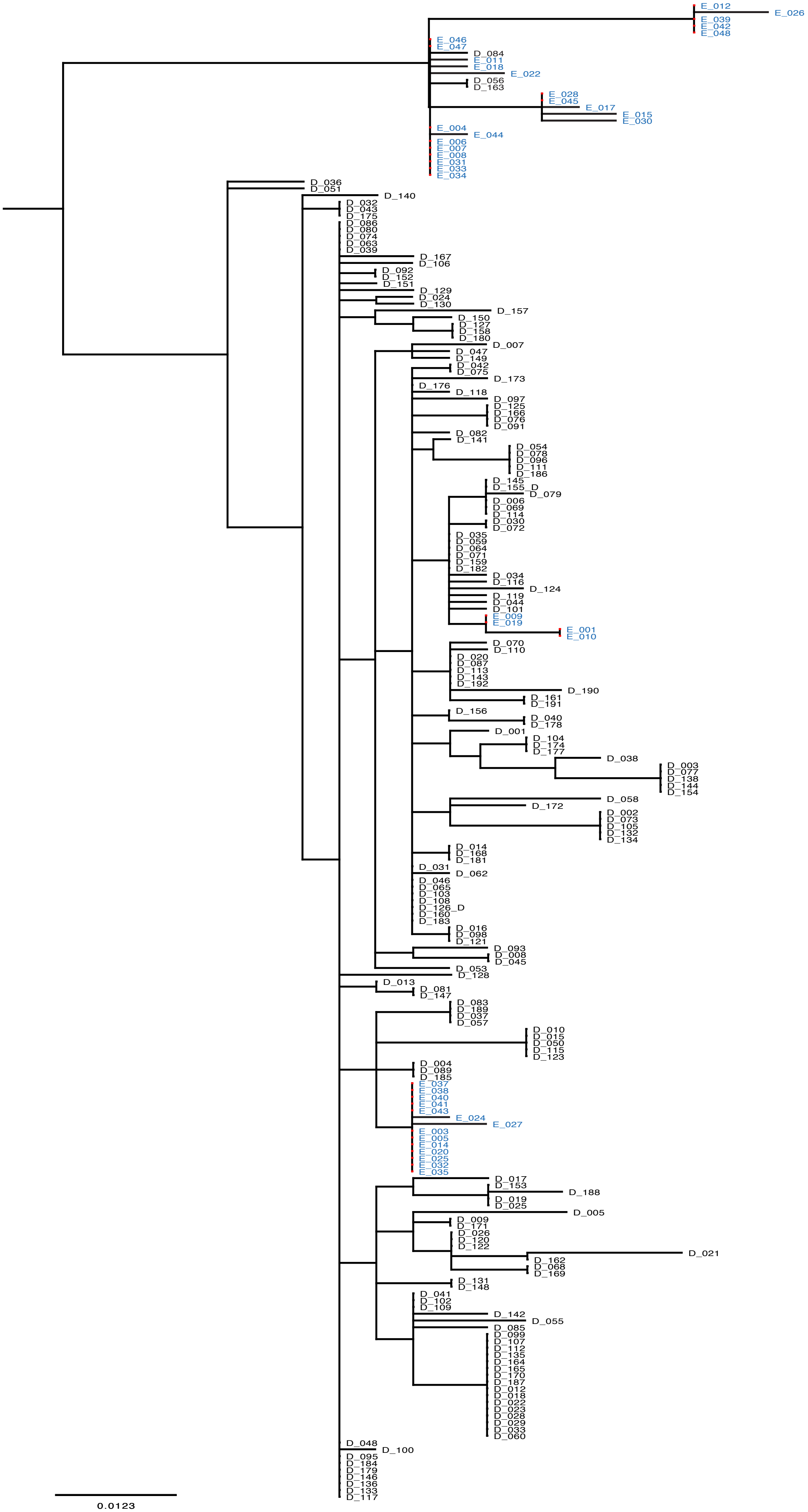
E_012
E_026
E_039
E_042
E_048
E_046
E_047
D_084
E_011
E_018
E_022
D_056
D_163
E_028
E_045
E_017
E_015
E_030
E_004
E_044
E_006
E_007
E_008
E_031
E_033
E_034
D_036
D_051
D_140
D_032
D_043
D_175
D_086
D_080
D_074
D_063
D_039
D_167
D_106
D_092
D_152
D_151
D_129
D_024
D_130
D_157
D_150
D_127
D_158
D_180
D_007
D_047
D_149
D_042
D_075
D_173
D_176
D_118
D_097
D_125
D_166
D_076
D_091
D_082
D_141
D_054
D_078
D_096
D_111
D_186
D_145
D_155
D
D_079
D_006
D_069
D_114
D_030
D_072
D_035
D_059
D_064
D_071
D_159
D_182
D_034
D_116
D_124
D_119
D_044
D_101
E_009
E_019
E_001
E_010
D_070
D_110
D_020
D_087
D_113
D_143
D_192
D_190
D_161
D_191
D_156
D_040
D_178
D_001
D_104
D_174
D_177
D_038
D_003
D_077
D_138
D_144
D_154
D_058
D_172
D_002
D_073
D_105
D_132
D_134
D_014
D_168
D_181
D_031
D_062
D_046
D_065
D_103
D_108
D_126
D
D_160
D_183
D_016
D_098
D_121
D_093
D_008
D_045
D_053
D_128
D_013
D_081
D_147
D_083
D_189
D_037
D_057
D_010
D_015
D_050
D_115
D_123
D_004
D_089
D_185
E_037
E_038
E_040
E_041
E_043
E_024
E_027
E_003
E_005
E_014
E_020
E_025
E_032
E_035
D_017
D_153
D_188
D_019
D_025
D_005
D_009
D_171
D_026
D_120
D_122
D_021
D_162
D_068
D_169
D_131
D_148
D_041
D_102
D_109
D_142
D_055
D_085
D_099
D_107
D_112
D_135
D_164
D_165
D_170
D_187
D_012
D_018
D_022
D_023
D_028
D_029
D_033
D_060
D_048
D_100
D_095
D_184
D_179
D_146
D_136
D_133
D_117
0.0123

Patient T16

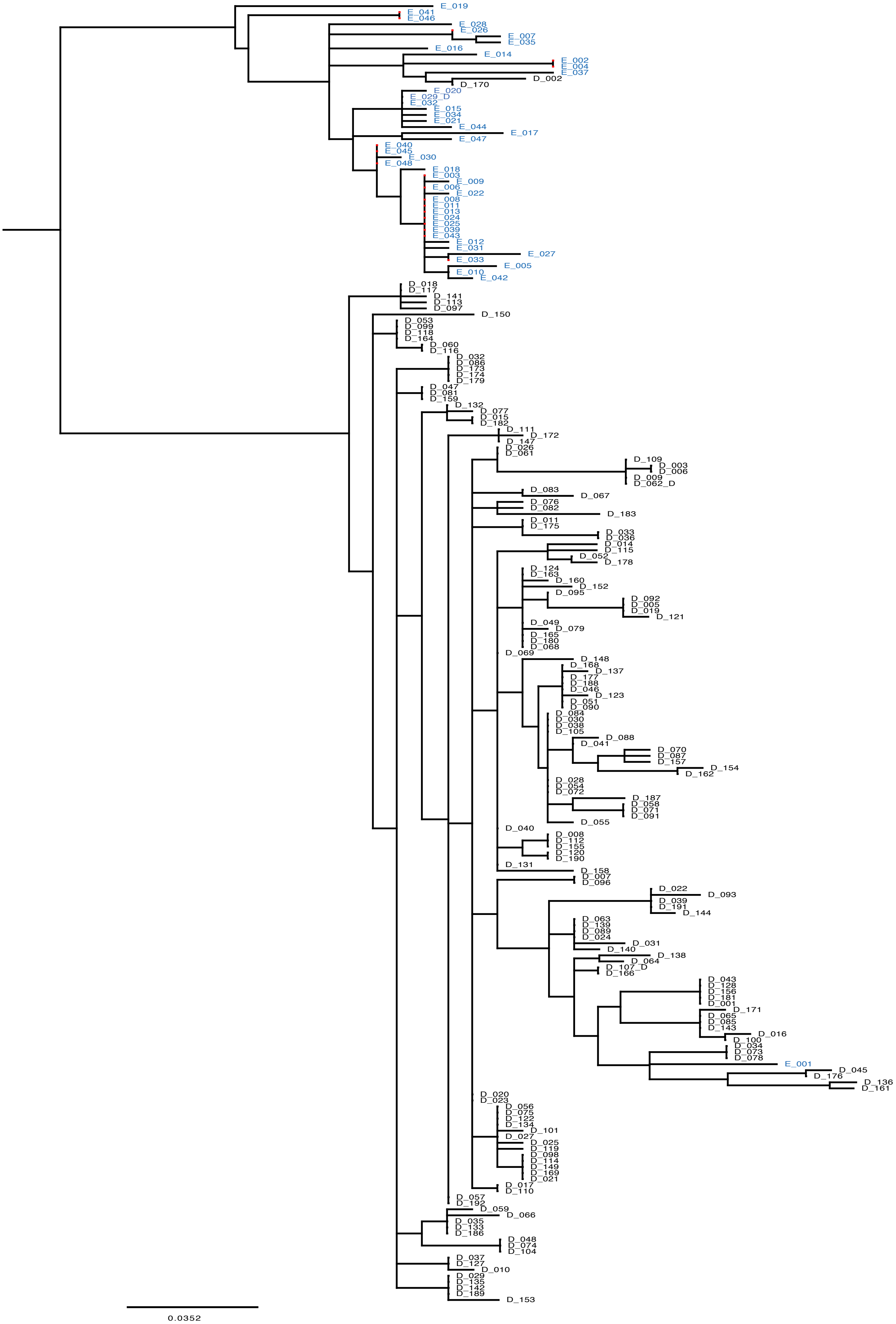
E_019
E_041
E_046
E_028
E_026
E_007
E_035
E_016
E_014
E_002
E_004
E_037
D_170
D_002
E_020
E_029_D
E_032
E_015
E_034
E_021
E_044
E_017
E_047
E_040
E_045
E_030
E_048
E_018
E_003
E_009
E_006
E_022
E_008
E_011
E_013
E_024
E_025
E_039
E_043
E_012
E_031
E_027
E_033
E_005
E_010
E_042
D_018
D_117
D_141
D_113
D_097
D_150
D_053
D_099
D_118
D_164
D_060
D_116
D_032
D_086
D_173
D_174
D_179
D_047
D_081
D_159
D_132
D_077
D_015
D_182
D_111
D_172
D_147
D_026
D_061
D_109
D_003
D_006
D_009
D_062_D
D_083
D_067
D_076
D_082
D_183
D_011
D_175
D_033
D_036
D_014
D_115
D_052
D_178
D_124
D_163
D_160
D_152
D_095
D_092
D_005
D_019
D_121
D_049
D_079
D_165
D_180
D_068
D_069
D_148
D_168
D_137
D_177
D_188
D_046
D_123
D_051
D_090
D_084
D_030
D_038
D_105
D_088
D_041
D_070
D_087
D_157
D_154
D_162
D_028
D_054
D_072
D_187
D_058
D_071
D_091
D_055
D_040
D_008
D_112
D_155
D_120
D_190
D_131
D_158
D_007
D_096
D_022
D_093
D_039
D_191
D_144
D_063
D_139
D_089
D_024
D_031
D_140
D_138
D_064
D_107_D
D_166
D_043
D_128
D_156
D_181
D_001
D_171
D_065
D_085
D_143
D_016
D_100
D_034
D_073
D_078
E_001
D_045
D_176
D_136
D_161
D_020
D_023
D_056
D_075
D_122
D_134
D_101
D_027
D_025
D_119
D_098
D_114
D_149
D_169
D_021
D_017
D_110
D_057
D_192
D_059
D_066
D_035
D_133
D_186
D_048
D_074
D_104
D_037
D_127
D_010
D_029
D_135
D_142
D_189
D_153
0.0352

Patient T17

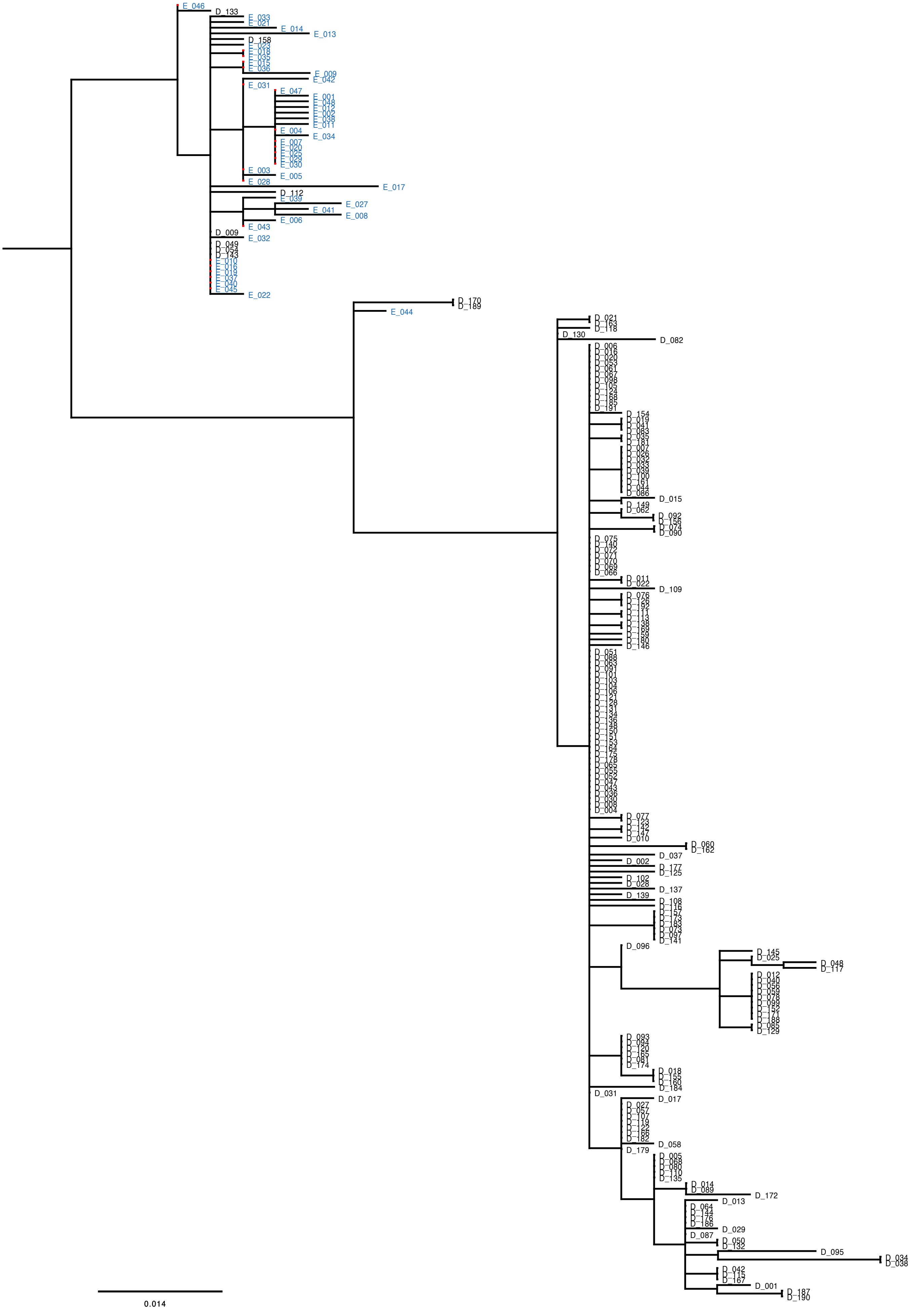

E_046
D_133
E_014
E_013
D_158
E_009
E_042
E_031
E_047
E_004
E_034
E_003
E_005
E_028
E_017
D_112
E_027
E_041
E_008
E_006
E_043
D_009
E_032
E_022
D_170
D_189
E_044
D_130
D_082
D_015
D_109
D_037
D_002
D_137
D_139
D_096
D_184
D_031
D_017
D_058
D_179
D_172
D_013
D_029
D_087
D_095
D_001
0.014

Patient T20

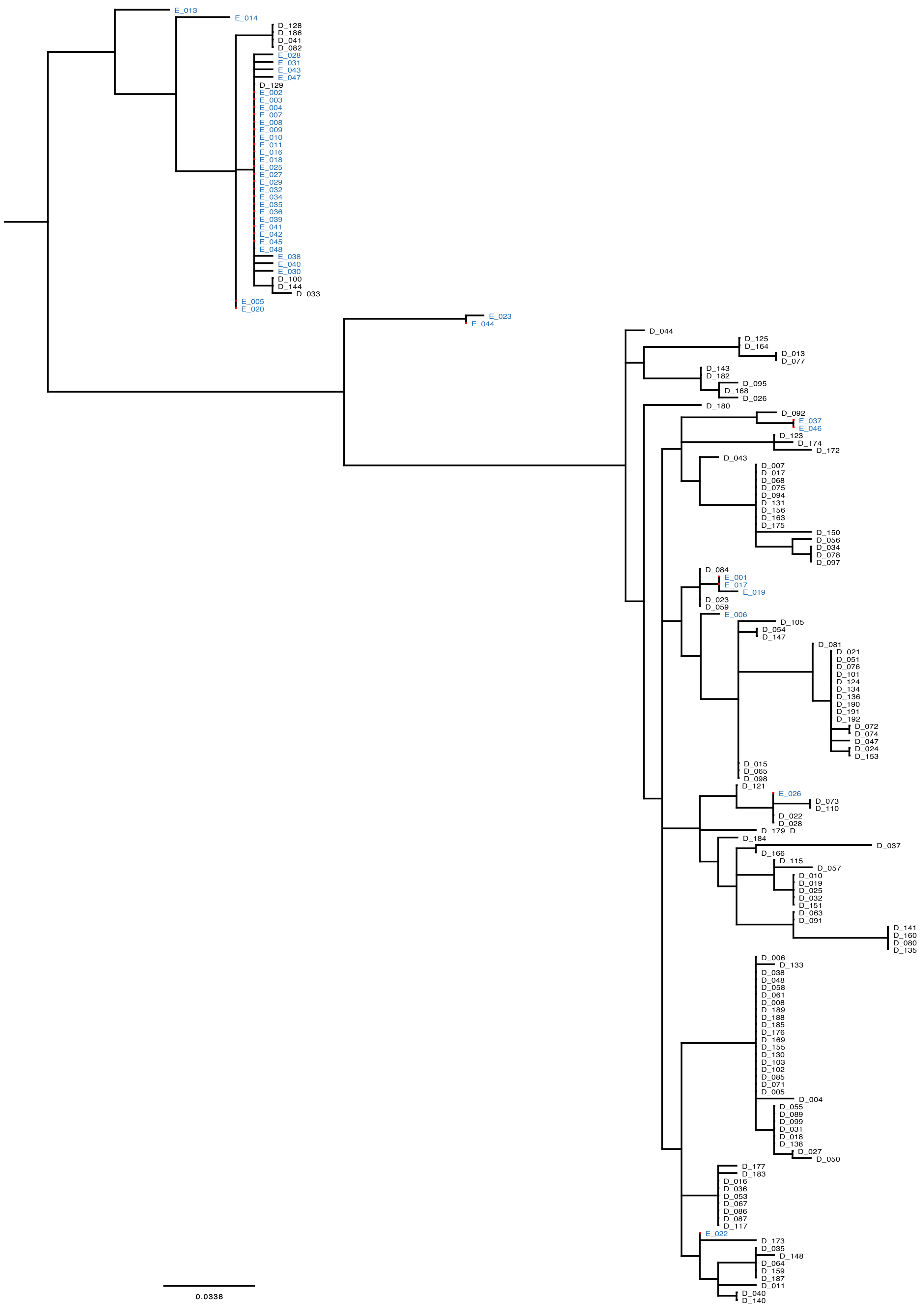

E_013
E_014
D_128
D_186
D_041
D_082
E_028
E_031
E_043
E_047
D_129
E_002
E_003
E_004
E_007
E_008
E_009
E_010
E_011
E_016
E_018
E_025
E_027
E_029
E_032
E_034
E_035
E_036
E_039
E_041
E_042
E_045
E_048
E_038
E_040
E_030
D_100
D_144
D_033
E_005
E_020
E_023
E_044
D_044
D_125
D_164
D_013
D_077
D_143
D_182
D_095
D_168
D_026
D_180
D_092
E_037
E_046
D_123
D_174
D_172
D_043
D_007
D_017
D_068
D_075
D_094
D_131
D_156
D_163
D_175
D_150
D_056
D_034
D_078
D_097
D_084
E_001
E_017
E_019
D_023
D_059
E_006
D_105
D_054
D_147
D_081
D_021
D_051
D_076
D_101
D_124
D_134
D_136
D_190
D_191
D_192
D_072
D_074
D_047
D_024
D_153
D_015
D_065
D_098
D_121
E_026
D_073
D_110
D_022
D_028
D_179_D
D_184
D_037
D_166
D_115
D_057
D_010
D_019
D_025
D_032
D_151
D_063
D_091
D_141
D_160
D_080
D_135
D_006
D_133
D_038
D_048
D_058
D_061
D_008
D_189
D_188
D_185
D_176
D_169
D_155
D_130
D_103
D_102
D_085
D_071
D_005
D_004
D_055
D_089
D_099
D_031
D_018
D_138
D_027
D_050
D_177
D_183
D_016
D_036
D_053
D_067
D_086
D_087
D_117
E_022
D_173
D_035
D_148
D_064
D_159
D_187
D_011
D_040
D_140
0.0338

Patient T69

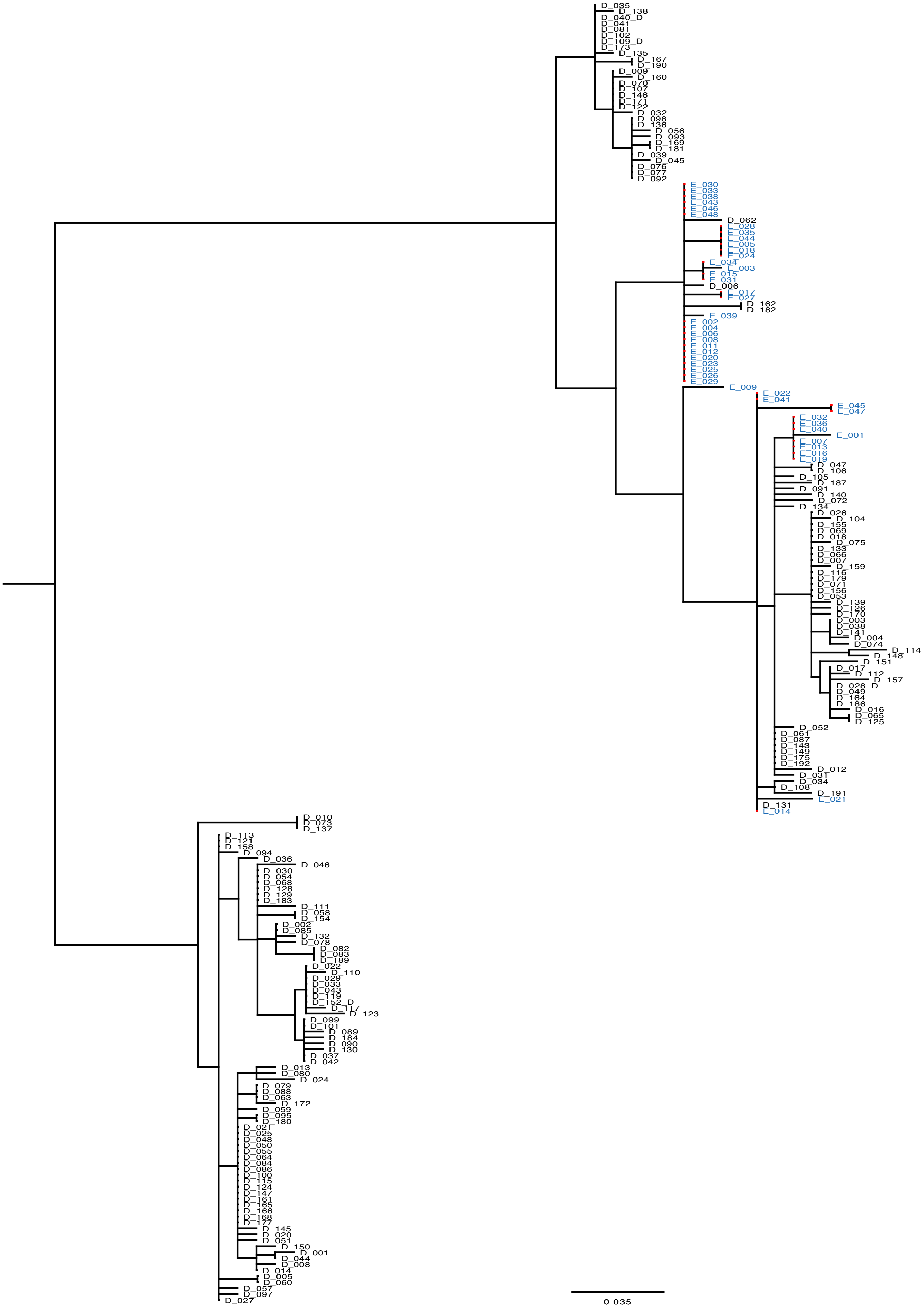

Patient T132

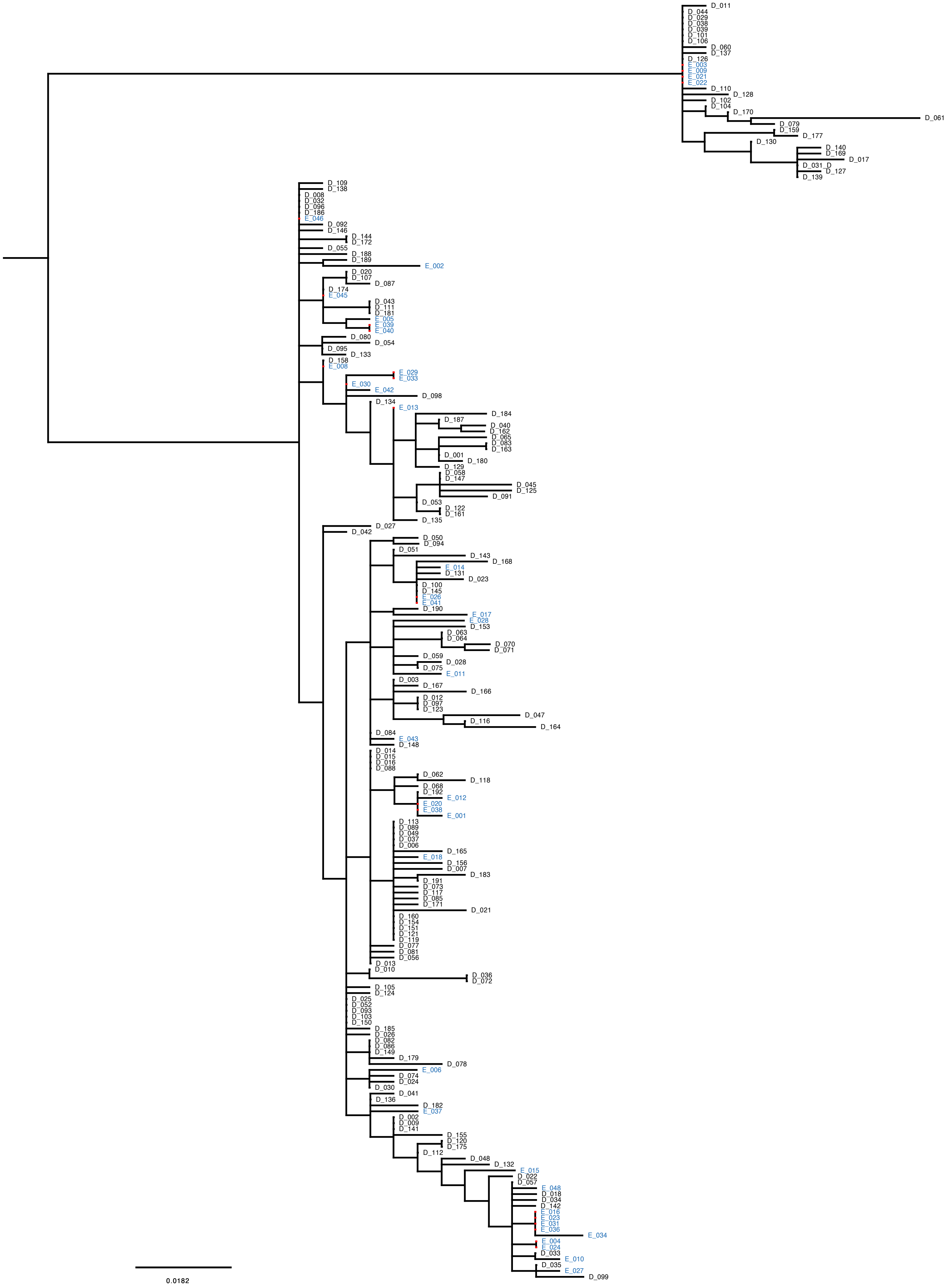

Patient T205

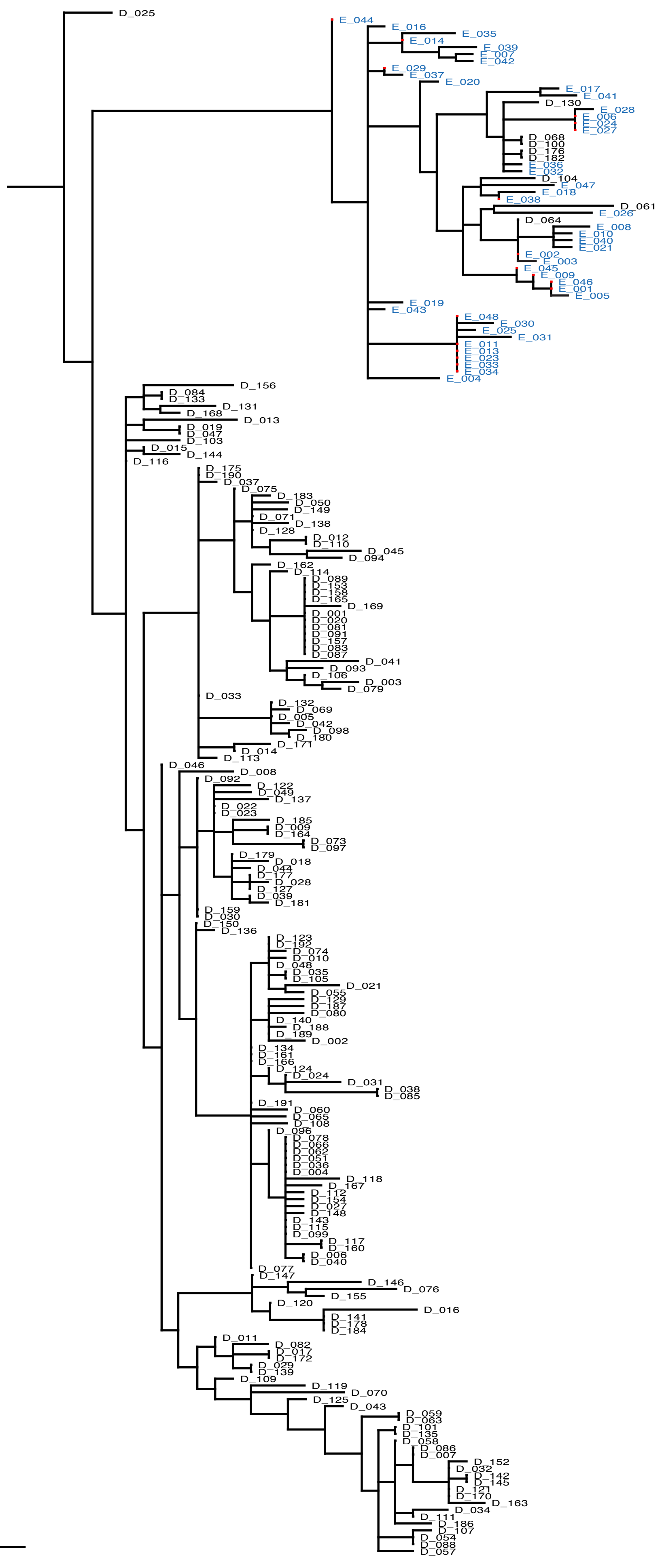

Patient T221

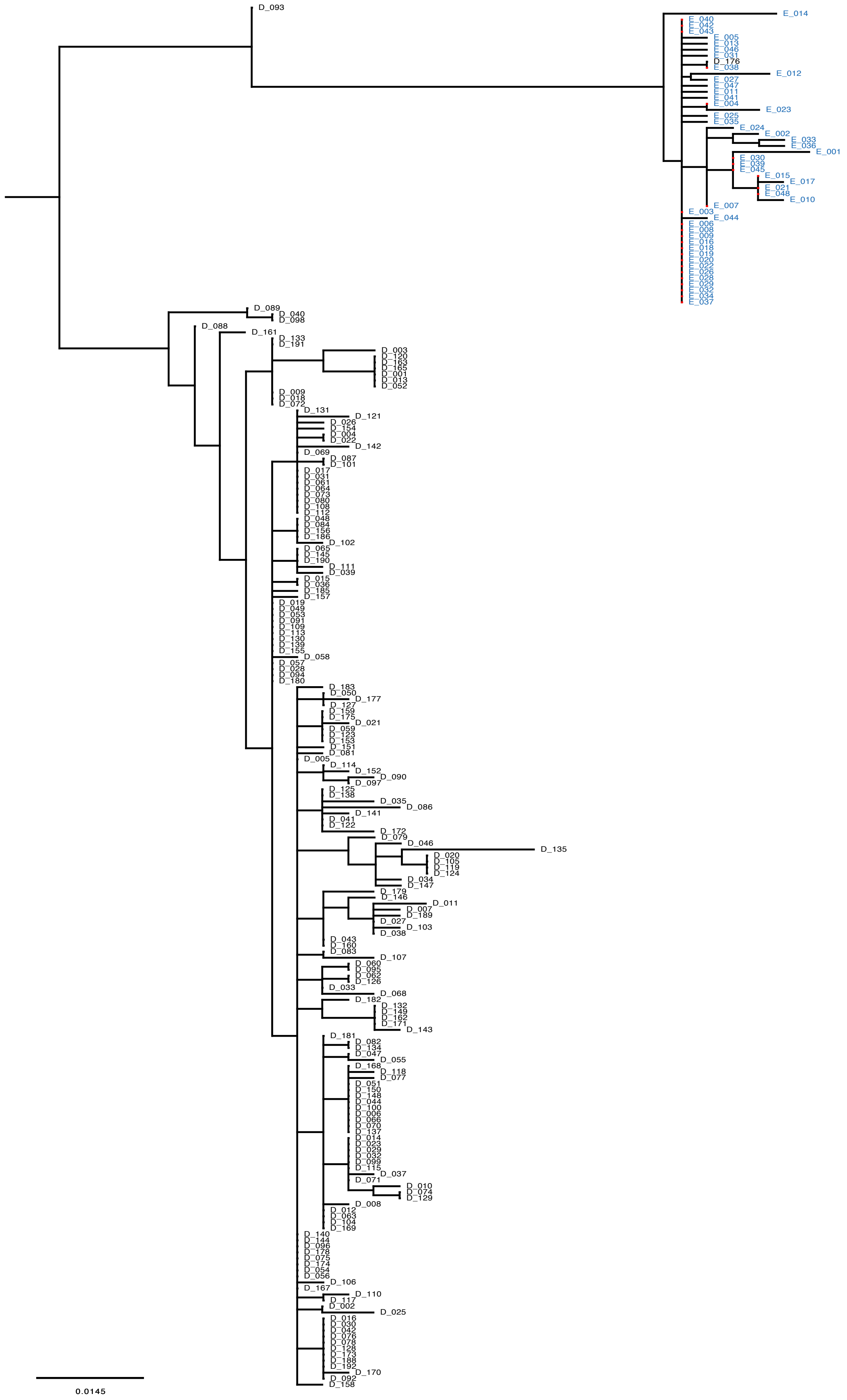

Patient T246

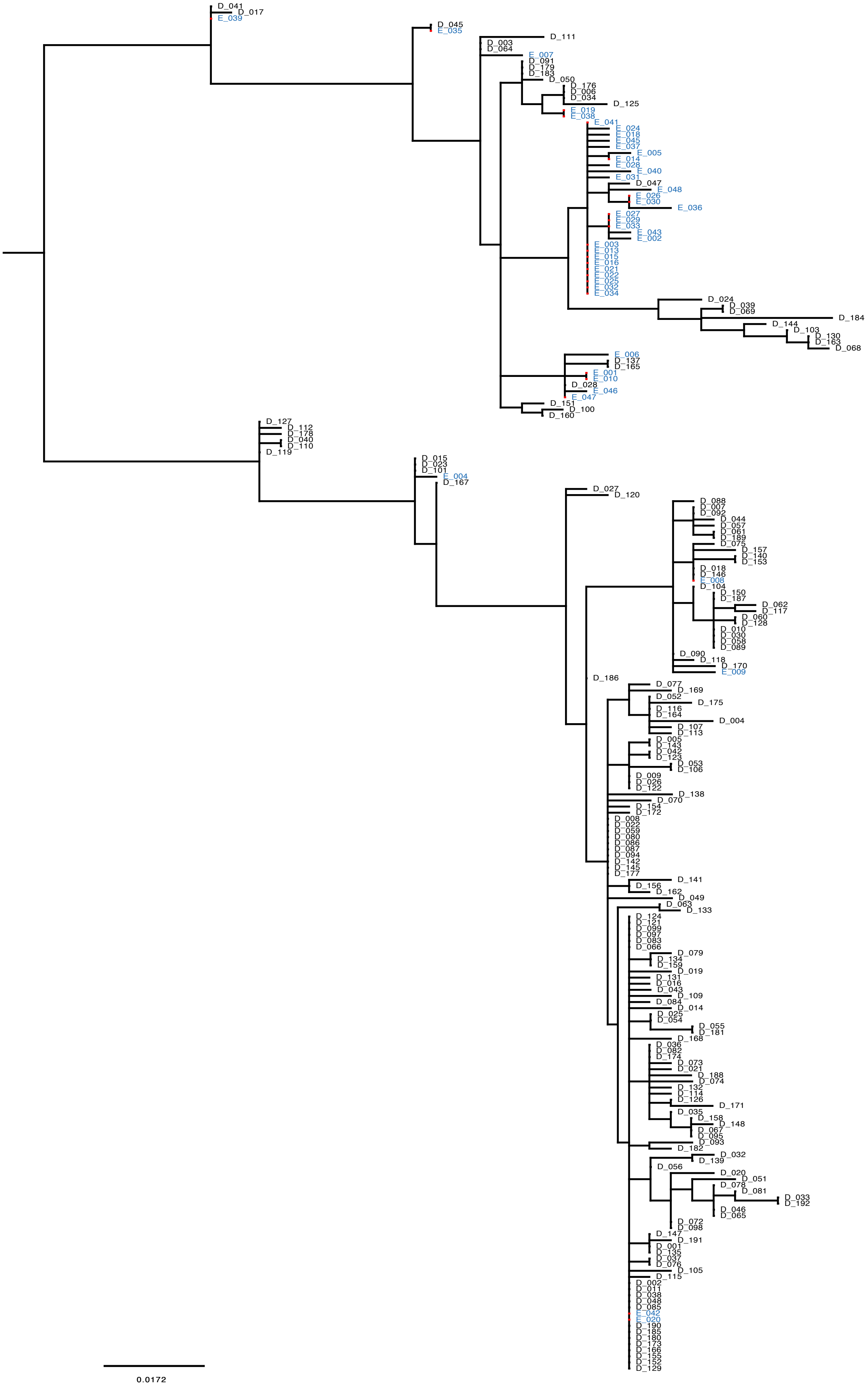

Patient T251

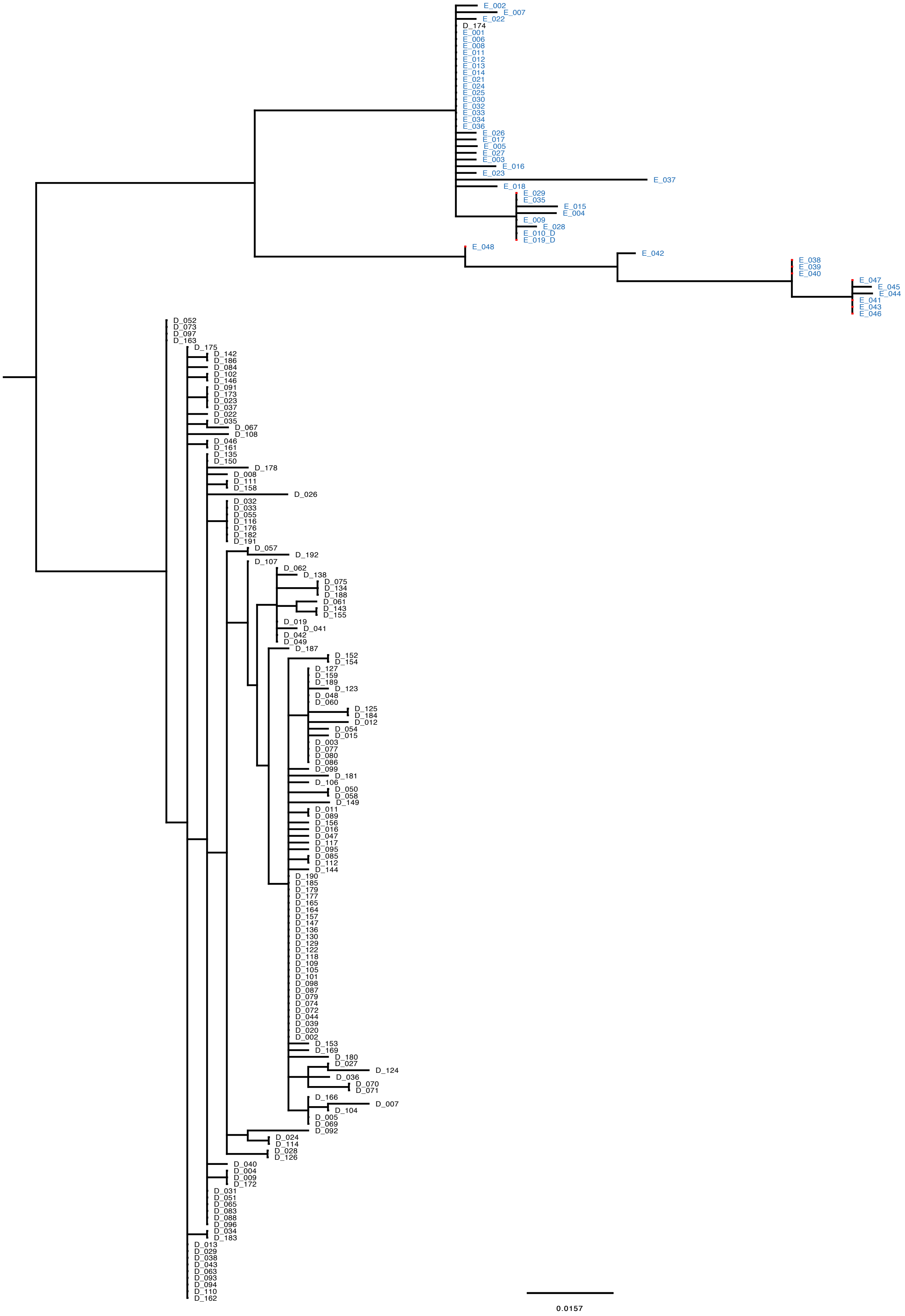

Patient T285

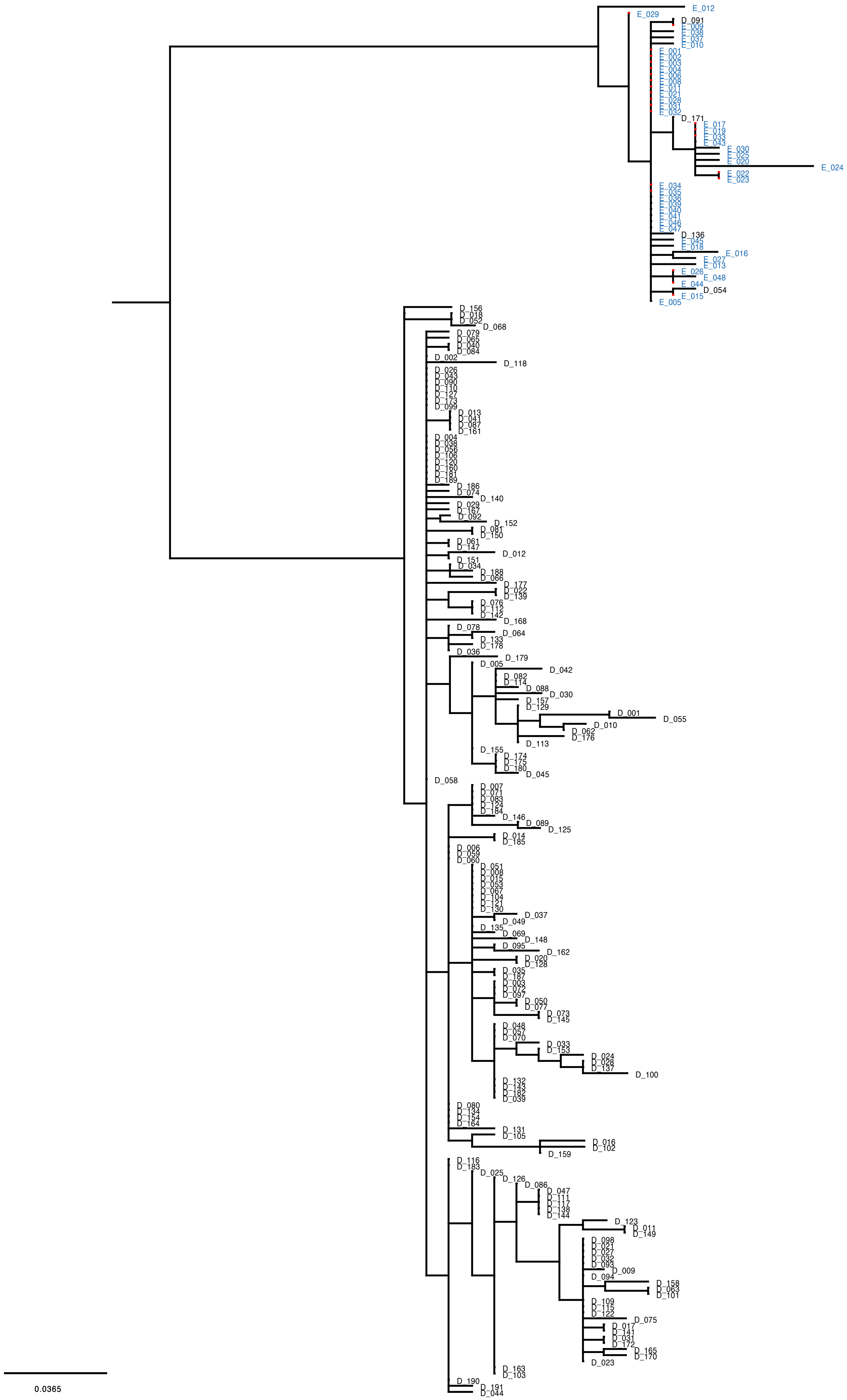

Patient T347

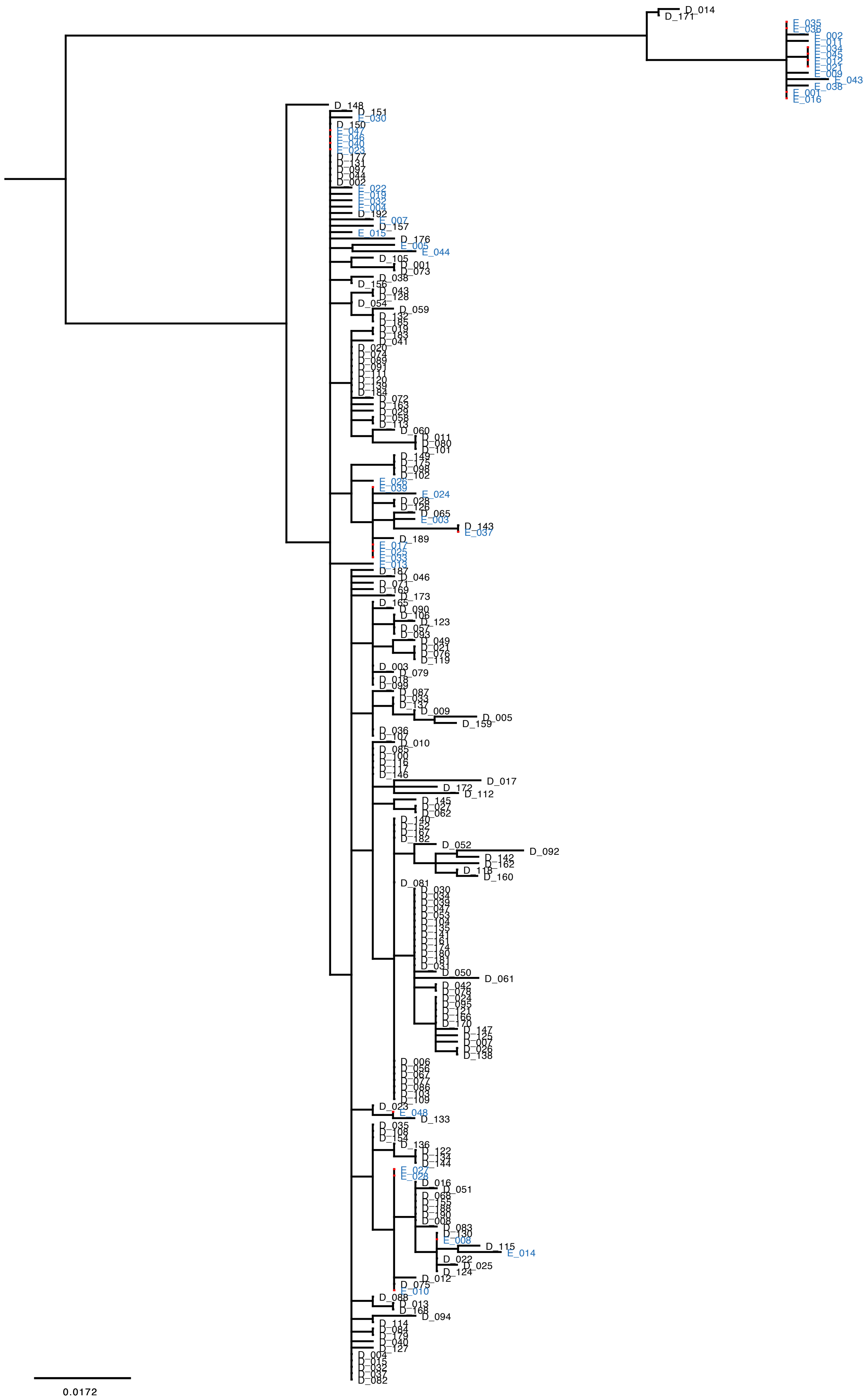

Patient T377

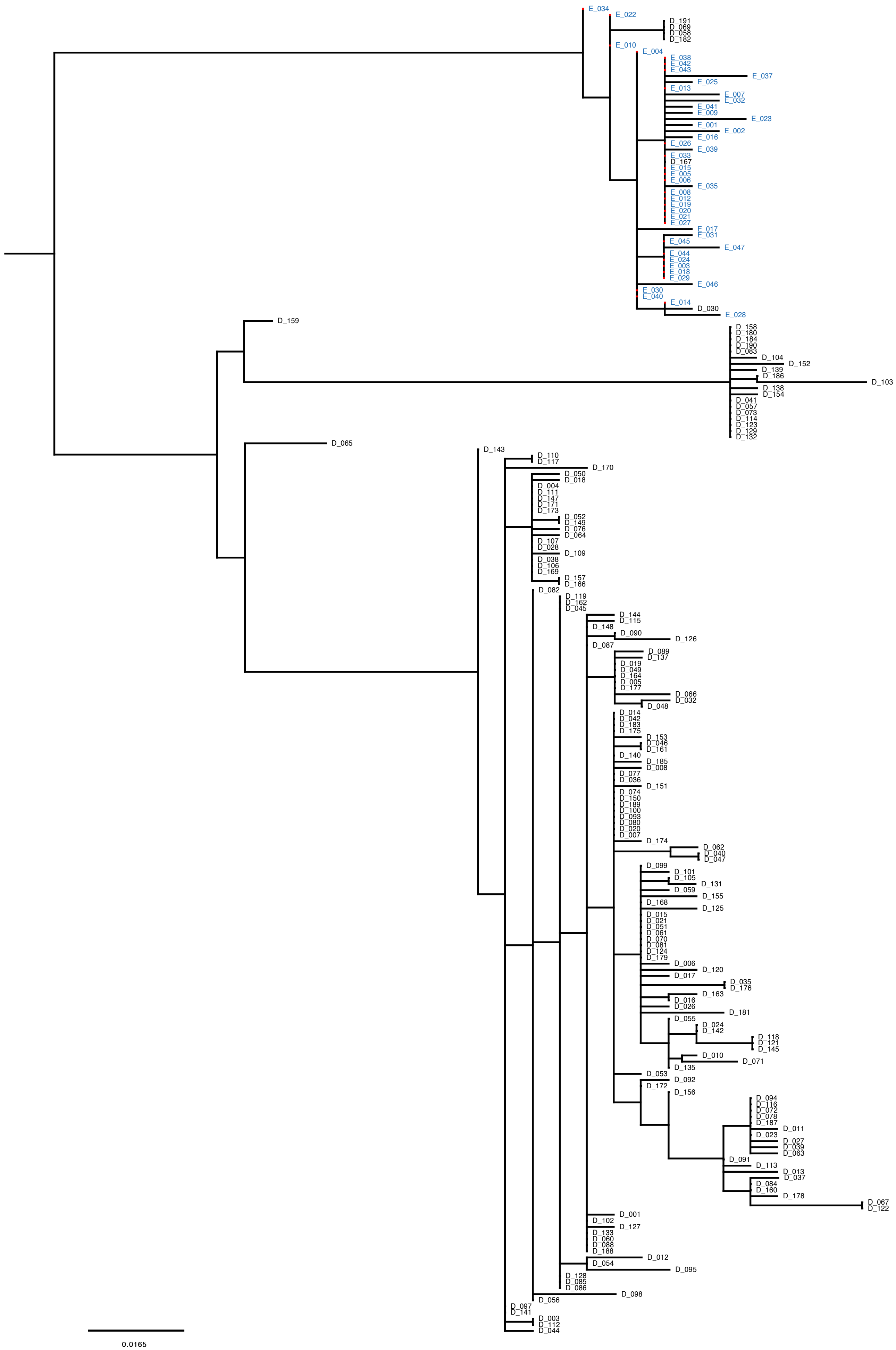

Patient T397

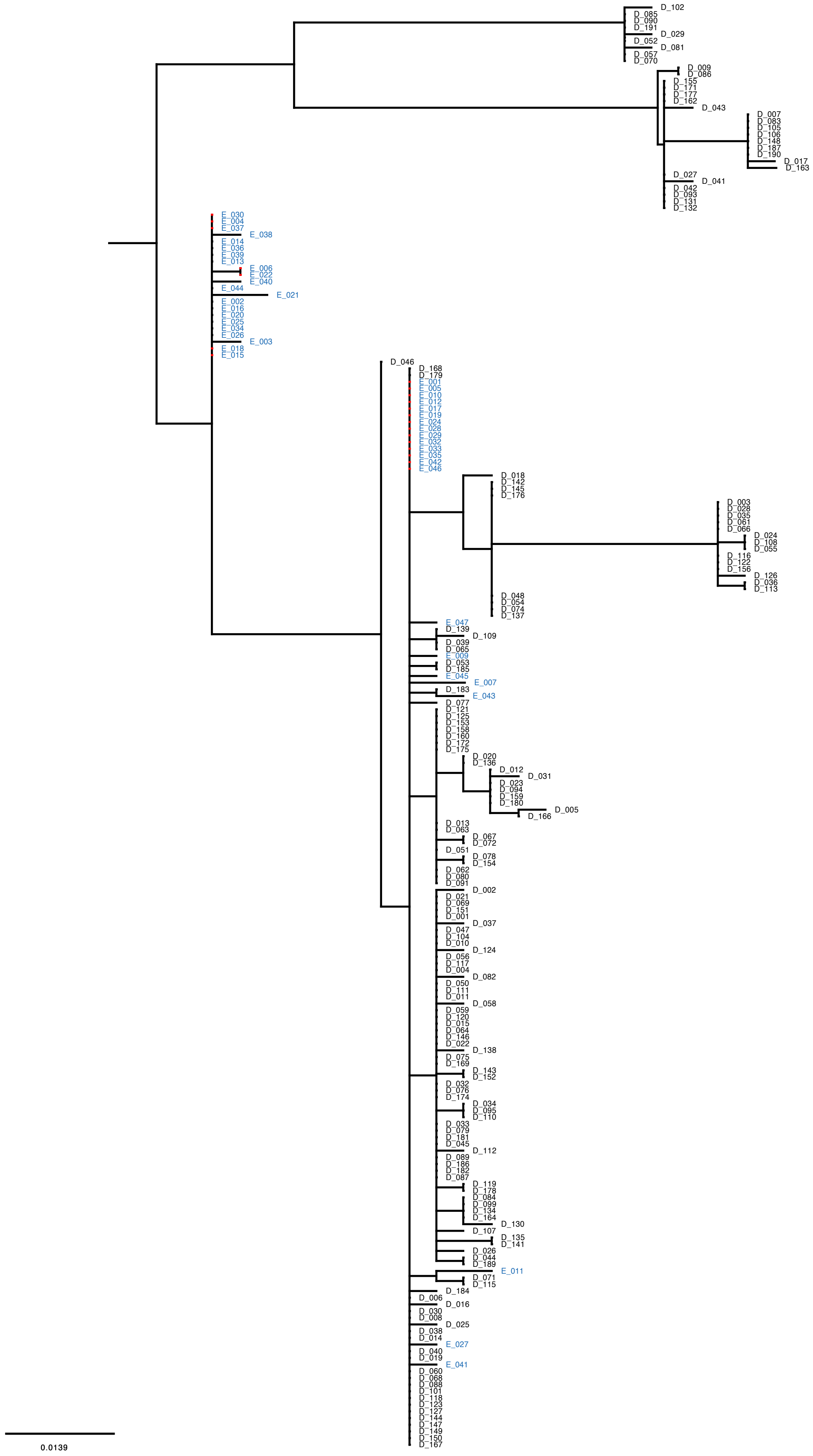

Patient T57

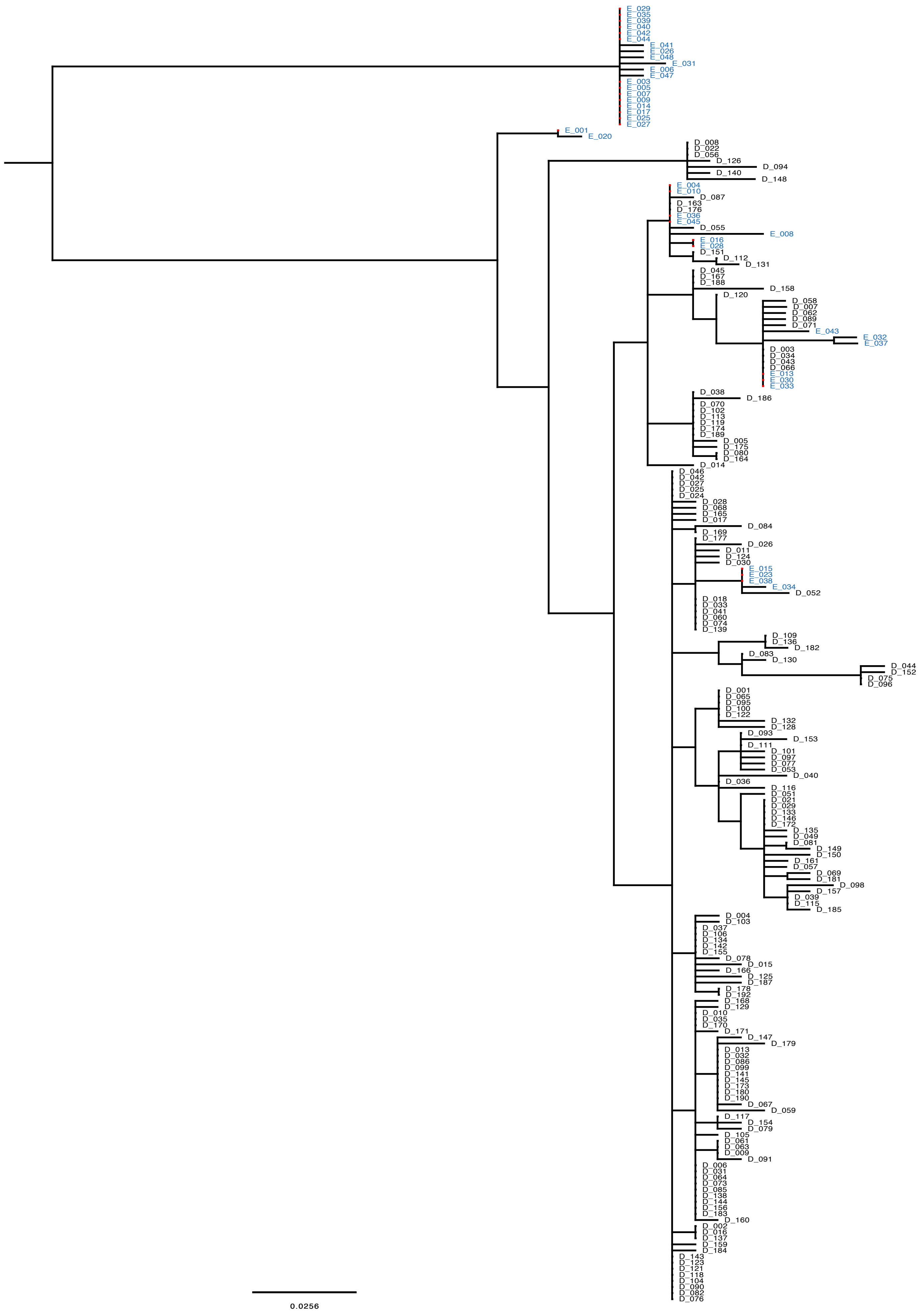
E_029
E_035
E_039
E_040
E_042
E_044
E_041
E_026
E_048
E_031
E_006
E_047
E_003
E_005
E_007
E_009
E_014
E_017
E_025
E_027
E_001
E_020
D_008
D_022
D_056
D_126
D_094
D_140
D_148
E_004
E_010
D_087
D_163
D_176
E_036
E_045
D_055
E_008
E_016
E_028
D_151
D_112
D_131
D_045
D_167
D_188
D_158
D_120
D_058
D_007
D_062
D_089
D_071
E_043
E_032
E_037
D_003
D_034
D_043
D_066
E_013
E_030
E_033
D_038
D_186
D_070
D_102
D_113
D_119
D_174
D_189
D_005
D_175
D_080
D_164
D_014
D_046
D_042
D_027
D_025
D_024
D_028
D_068
D_165
D_017
D_084
D_169
D_177
D_026
D_011
D_124
D_030
E_015
E_023
E_038
E_034
D_052
D_018
D_033
D_041
D_060
D_074
D_139
D_109
D_136
D_182
D_083
D_130
D_044
D_152
D_075
D_096
D_001
D_065
D_095
D_100
D_122
D_132
D_128
D_093
D_153
D_111
D_101
D_097
D_077
D_053
D_040
D_036
D_116
D_051
D_021
D_029
D_133
D_146
D_172
D_135
D_049
D_081
D_149
D_150
D_161
D_057
D_069
D_181
D_098
D_157
D_039
D_115
D_185
D_004
D_103
D_037
D_106
D_134
D_142
D_155
D_078
D_015
D_166
D_125
D_187
D_178
D_192
D_168
D_129
D_010
D_035
D_170
D_171
D_147
D_179
D_013
D_032
D_086
D_099
D_141
D_145
D_173
D_180
D_190
D_067
D_059
D_117
D_154
D_079
D_105
D_061
D_063
D_009
D_091
D_006
D_031
D_064
D_073
D_085
D_138
D_144
D_156
D_183
D_160
D_002
D_016
D_137
D_159
D_184
D_143
D_123
D_121
D_118
D_104
D_090
D_082
D_076
0.0256

Patient T210

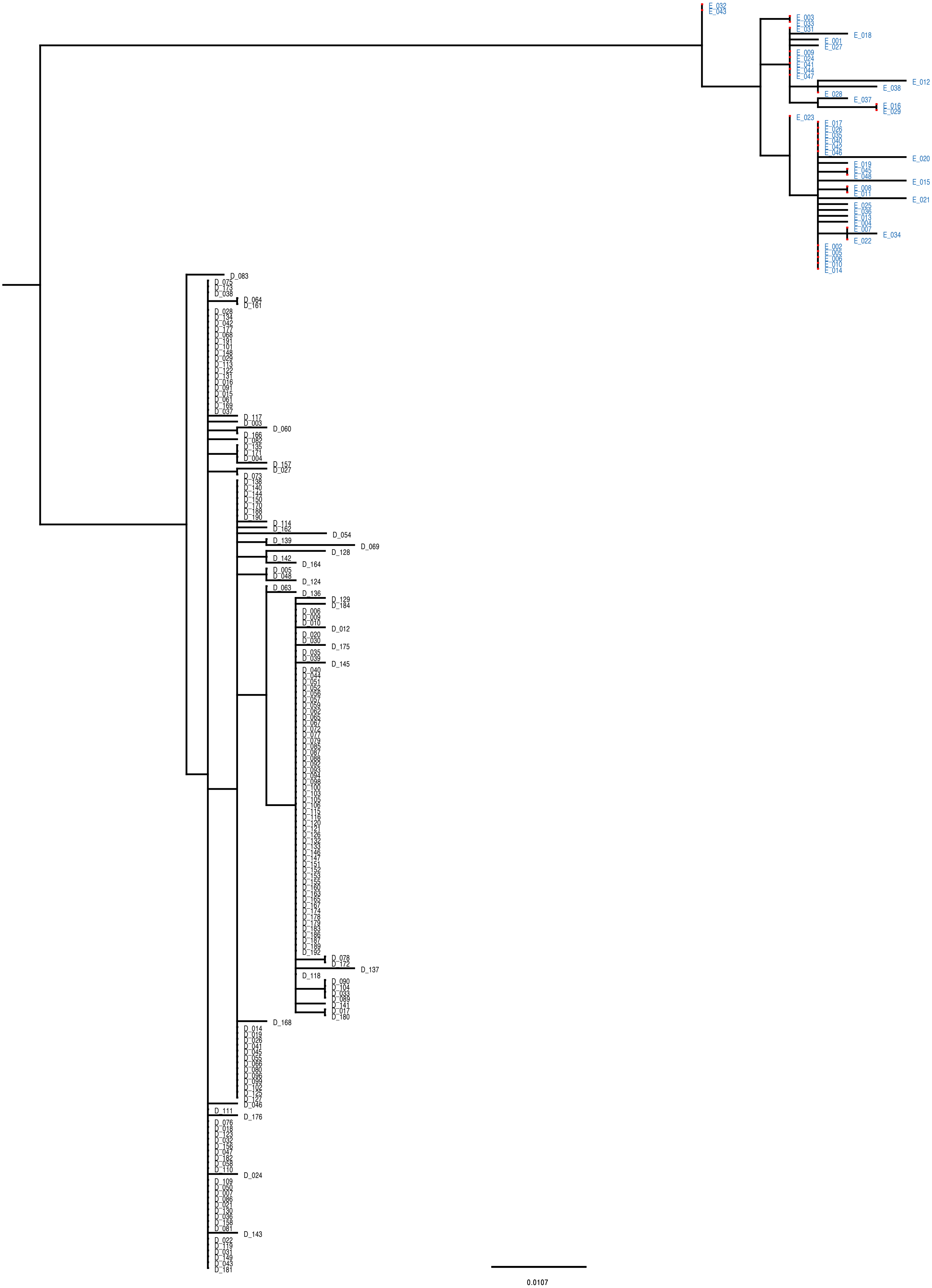

Patient T398

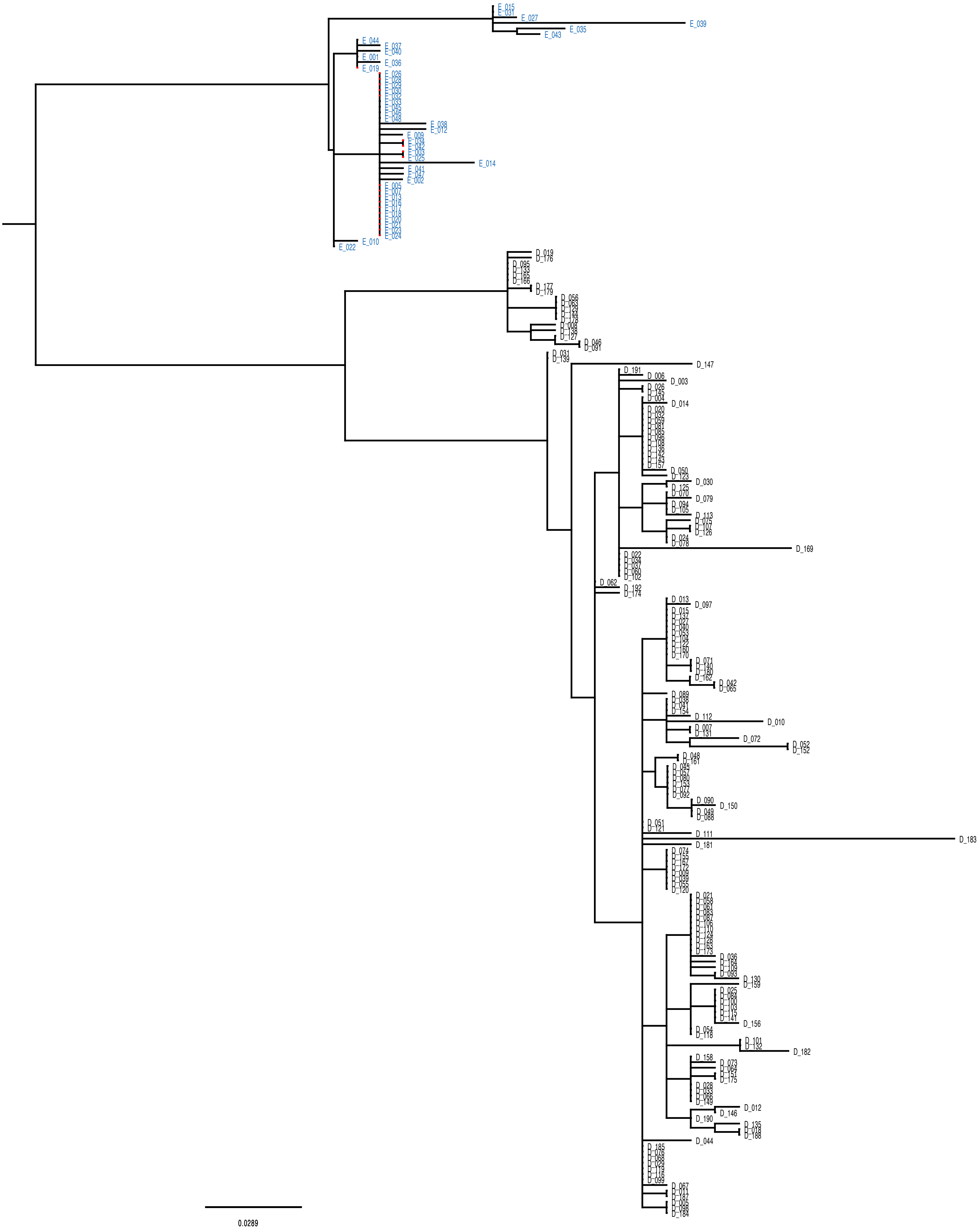

Patient T415

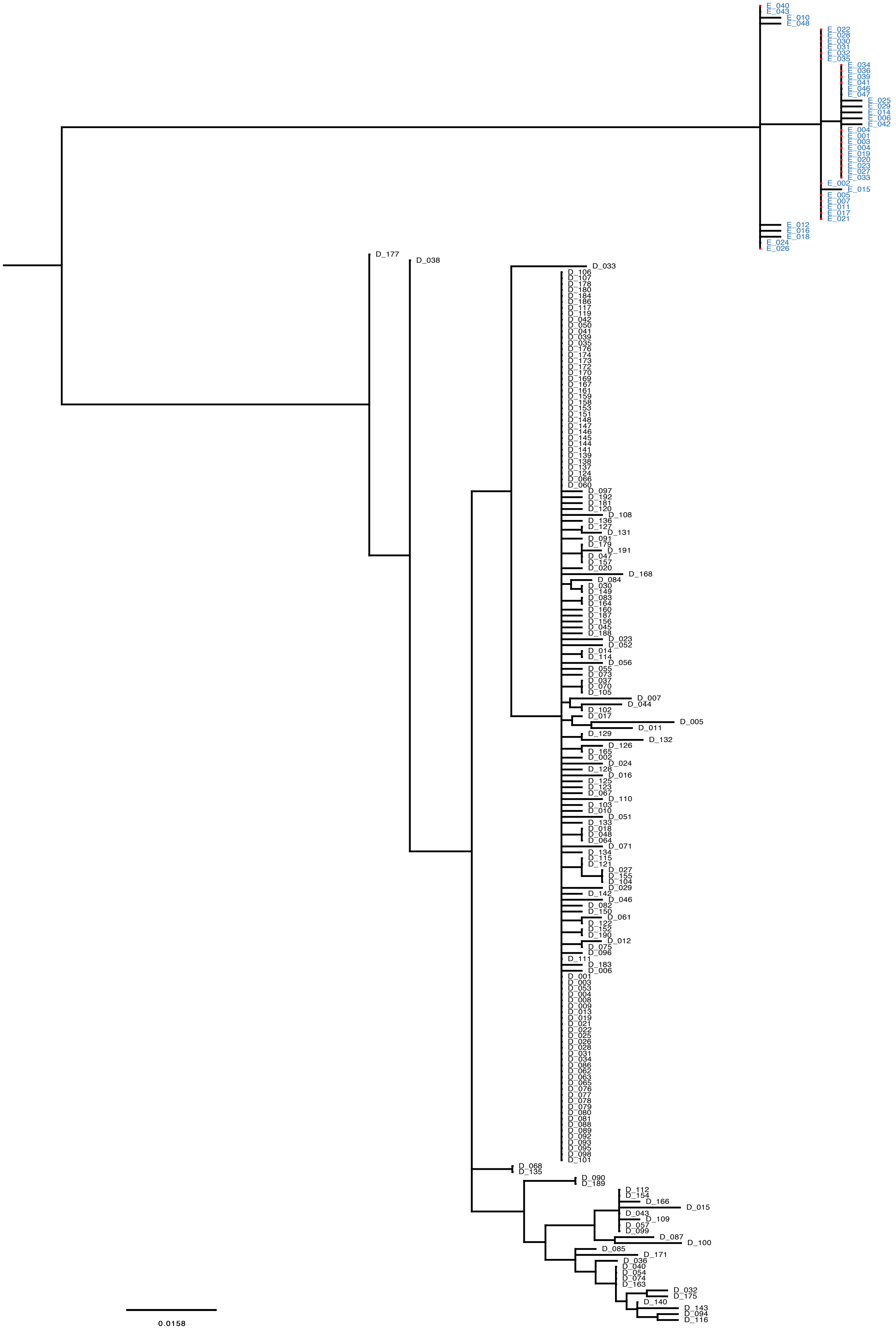

Patient T629

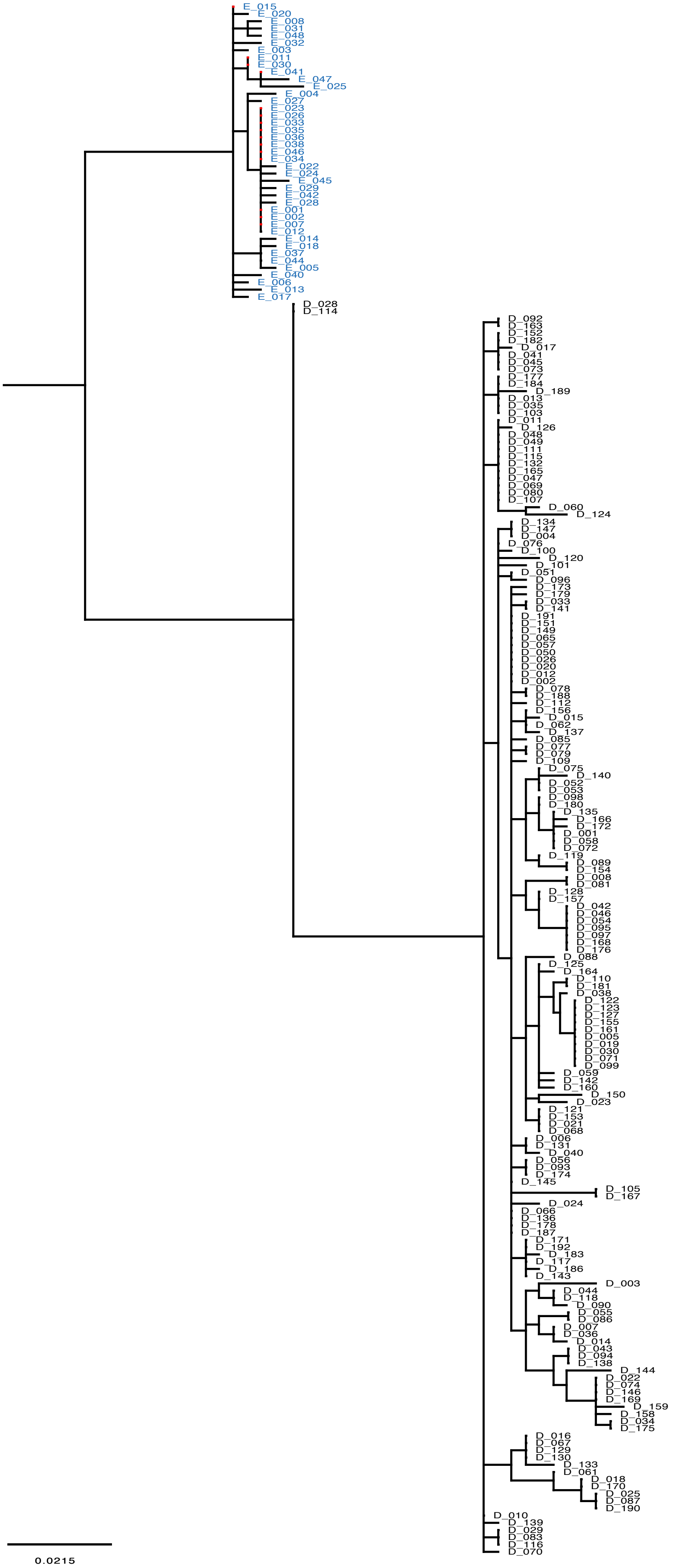

Patient T825

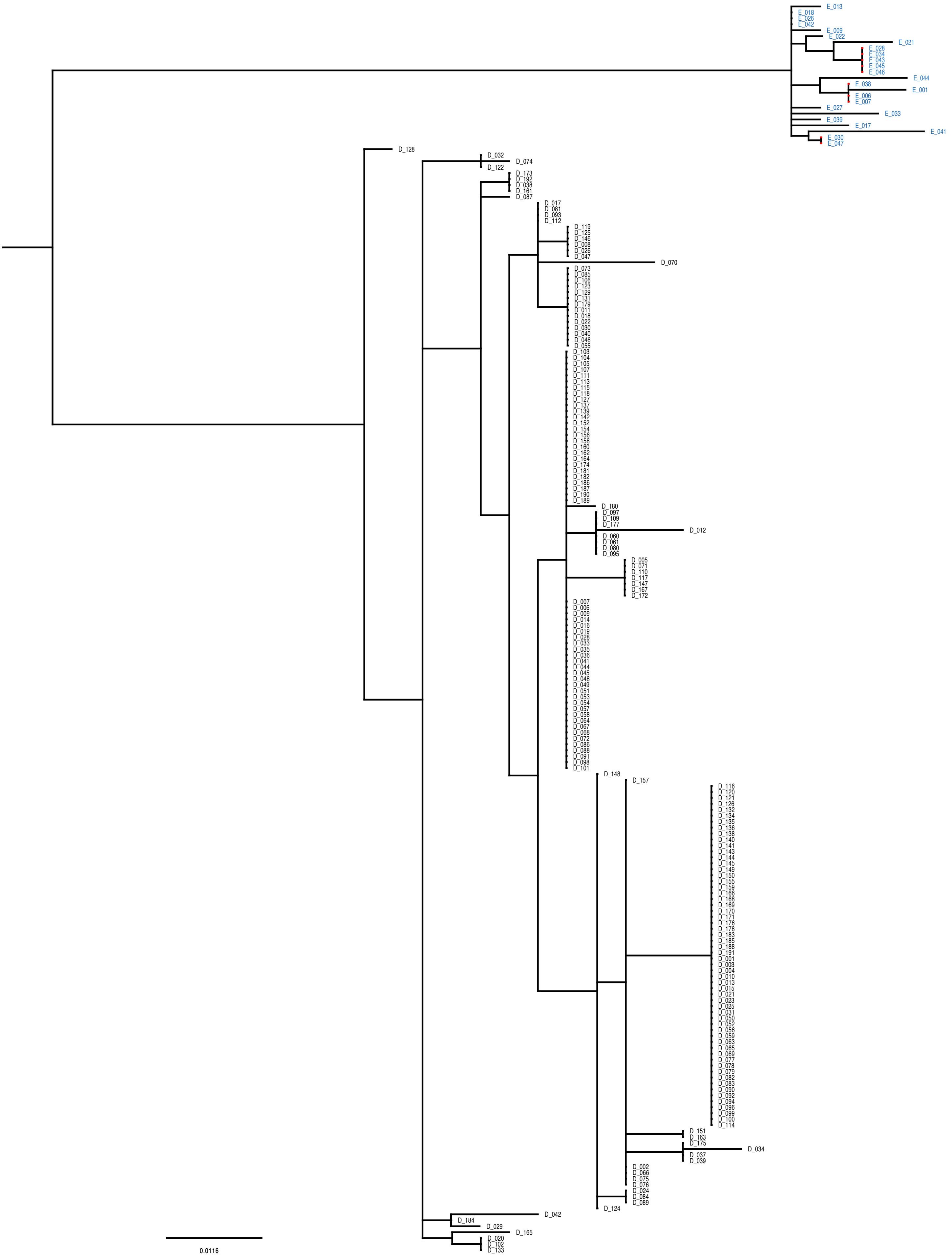

Supplement: S2 Fig — Each tree is rooted using HXB2 (NCBI accession number K03455). Examples of 2 clonal sequences: R5 (Day 1) and CXCR4-using (on-treatment) from 3 unrelated individuals were included together with the patients’ Day 1 and on-treatment nucleotide sequences. CLUSTAL_X was used to infer the neighbor-joining tree and the diagrammatic representation was created using MEGA, collapsing the branches with the size of the triangle representative of the number of sequences in the branch and the bootstrap values from ‘100 trials’ added to the nodes. Only those values greater than 75 were included. Green triangles and lines represents sequences from R5 clones and blue triangles and lines those from CXCR4-using clones. If the branch contained a mix of R5 and confirmed CXCR4-using clones, then that branch and triangle are colored blue. Black lines represent non-functional clones where the tropism could not be determined phenotypically. Individual clones not clustering in the main lineages are represented by single lines. Sequence diversity is represented by horizontal distance. (PDF) [file pone.0204099.s003.pdf]
