# Supplementary material for: Clonal analysis of HIV-1 genotype and function associated with virologic failure in treatment-experienced persons receiving maraviroc: Results from the MOTIVATE phase 3 randomized, placebo-controlled trials
Source: PLoS One. 2018 Dec 26;13(12):e0204099. doi: 10.1371/journal.pone.0204099 (PMC6306210; doi:10.1371/journal.pone.0204099)
Supplement: S3 Fig — Each tree is rooted using HXB2 (NCBI accession number: K03455) and inferred using PAUP with the HKY + gamma model of nucleotide substitution and NNI heuristic settings. Day 1 clones are labeled D and in black font. On-treatment clones are labeled E and in blue font. (PDF) [file pone.0204099.s004.pdf]

S3 Fig. Predicted number of Env clones required to screen to detect minority CXCR4-using variants.

(A) Number of test results predicted to be required for 95% and 99% certainty of detecting CXCR4-using virus. Values come from probability model, $n = \log(1-P)/\log(1-p)$, where the probability of detecting one or more CXCR4-using Env clones is P, the proportion of the CXCR4-using Env clones in the population is p, and the sample size is n. (B) Number of functional Env clones and total number of clones to be tested for 95% and 99% probability of detecting a clone with CXCR4-using properties when present with 1%, 5%, and 10% prevalence.

(A)

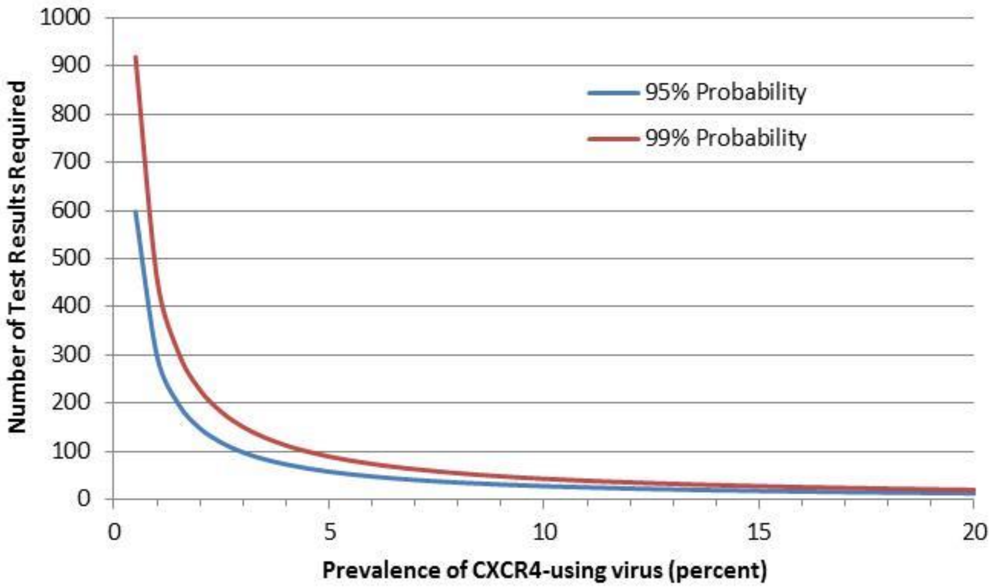

(B)

Actual incidence of CXCR4-using clones in population (%)	Number of functional Env clones needed (total clones to be screened)[a]	
	95% probability of detection	99% probability of detection
10	28 (56)	44 (88)
5	58 (116)	**90 (180)**
1	298 (596)	458 (916)

[a]Values shown in brackets assume 50% of all Env clones screened are non-functional. This model predicts that the cloning method used (i.e. testing 192 clones at the pre-treatment time point) gives a ~99% probability of detecting CXCR4-using Env clones present in the sample at ≥5% incidence (shaded box).

1. Gulick RM, Lalezari J, Goodrich J, Clumeck N, DeJesus E, Horban A, et al. Maraviroc for previously treated patients with R5 HIV-1 infection. N Engl J Med. 2008;359(14): 1429-1441.
2. Fatkenheuer G, Nelson M, Lazzarin A, Konourina I, Hoepelman AI, Lampiris H, et al. Subgroup analyses of maraviroc in previously treated R5 HIV-1 infection. N Engl J Med. 2008;359(14): 1442-1455.
