# Supplementary material for: Clonal analysis of HIV-1 genotype and function associated with virologic failure in treatment-experienced persons receiving maraviroc: Results from the MOTIVATE phase 3 randomized, placebo-controlled trials
Source: PLoS One. 2018 Dec 26;13(12):e0204099. doi: 10.1371/journal.pone.0204099 (PMC6306210; doi:10.1371/journal.pone.0204099)
Supplement: S4 Fig — (A) Change in viral load (◊ dashed line) and CD4 (▪ solid line) for participant 8, who received maraviroc in combination with didanosine (ddI), tenofovir (TFV), lopinavir/r (LPV/r), and enfuvirtide (ENF) for 27 weeks (shaded area) and failed therapy at Week 24 (red vertical dotted line) with R5 virus. Regimen prior to Day 1: ddI, LPV/r, ENF. OBT from Day 1: ddI, TFV, LPV/r, ENF. Screening: ddIsens, TFVsens, LPV/rres, ENFres (V38A). Failure: ddIres, TFVres, LPV/rres, ENFres (V38A). (B) Change in viral load (◊ dashed line) and CD4 (▪ solid line) for participant 11, who received maraviroc in combination with emtricitabine (FTC), tenofovir (TFV), stavudine (d4T), and enfuvirtide (ENF) for 15 weeks (shaded area) and failed therapy at Week 8 (red vertical dotted line) with R5 virus. Regimen prior to Day 1: FTC, TFV, d4T, TPV/r, ENF. OBT from Day 1: FTC, TFV, d4T, RTV, ENF. Screening: FTCres, TFVsens, d4Tsens, RTVres, ENFres (N43D/N). Failure: FTCres, TFVsens, d4Tres, RTVres, ENFres (N43D/N). (C) Change in viral load (◊ dashed line) and CD4 (▪ solid line) for participant 12, who received maraviroc in combination with didanosine (ddI), tenofovir (TFV), amprenavir (AMP), and enfuvirtide (ENF) for 12 weeks (shaded area) and failed therapy at Week 8 (red vertical dotted line) with R5 virus. Open-label therapy with maraviroc commenced Week 21 and finished Week 60 (shaded area). Regimen prior to Day 1: ddI, TFV, AMP/r, ENF. OBT from Day 1: ddI, TFV, FPV/r, ENF. Screening: ddIres, TFVres, FPV/rres, ENFres (N43D). Failure: ddIres, TFVres, FPV/rres, ENFres (N34D). (D) Change in viral load (◊ dashed line) and CD4 (▪ solid line) for participant 16, who received placebo in combination with abacavir (ABC) and tenofovir (TFV) for 14 weeks and failed therapy at Week 8 (red vertical dotted line) with R5 virus. Open-label therapy with maraviroc commenced Week 24 and finished Week 60 (shaded area). Regimen prior to Day 1: 3TC, TFV, ATZ/r. OBT from Day 1: ABC, 3TC, TFV. Screening: ABCsens,* [file pone.0204099.s005.pdf]

S4 Fig. Virologic and immunologic analyses for 4 participants who received open-label maraviroc following failure.

(A) Change in viral load (◊ dashed line) and CD4 (▪ solid line) for participant 8, who received maraviroc in combination with didanosine (ddI), tenofovir (TFV), lopinavir/r (LPV/r), and enfuvirtide (ENF) for 27 weeks (shaded area) and failed therapy at Week 24 (red vertical dotted line) with R5 virus. Regimen prior to Day 1: ddI, LPV/r, ENF. OBT from Day 1: ddI, TFV, LPV/r, ENF. Screening: ddIsens, TFVsens, LPV/rres, ENFres (V38A). Failure: ddIres, TFVres, LPV/rres, ENFres (V38A). (B) Change in viral load (◊ dashed line) and CD4 (▪ solid line) for participant 11, who received maraviroc in combination with emtricitabine (FTC), tenofovir (TFV), stavudine (d4T), and enfuvirtide (ENF) for 15 weeks (shaded area) and failed therapy at Week 8 (red vertical dotted line) with R5 virus. Regimen prior to Day 1: FTC, TFV, d4T, TPV/r, ENF. OBT from Day 1: FTC, TFV, d4T, RTV, ENF. Screening: FTCres, TFVsens, d4Tsens, RTVres, ENFres (N43D/N). Failure: FTCres, TFVsens, d4Tres, RTVres, ENFres (N43D/N). (C) Change in viral load (◊ dashed line) and CD4 (▪ solid line) for participant 12, who received maraviroc in combination with didanosine (ddI), tenofovir (TFV), amprenavir (AMP), and enfuvirtide (ENF) for 12 weeks (shaded area) and failed therapy at Week 8 (red vertical dotted line) with R5 virus. Open-label therapy with maraviroc commenced Week 21 and finished Week 60 (shaded area). Regimen prior to Day 1: ddI, TFV, AMP/r, ENF. OBT from Day 1: ddI, TFV, FPV/r, ENF. Screening: ddIres, TFVres, FPV/rres, ENFres (N43D). Failure: ddIres, TFVres, FPV/rres, ENFres (N34D). (D) Change in viral load (◊ dashed line) and CD4 (▪ solid line) for participant 16, who received placebo in combination with abacavir (ABC) and tenofovir (TFV) for 14 weeks and failed therapy at Week 8 (red vertical dotted line) with R5 virus. Open-label therapy with maraviroc commenced Week 24 and finished Week 60 (shaded area). Regimen prior to Day 1: 3TC, TFV, ATZ/r. OBT from Day 1: ABC, 3TC, TFV. Screening: ABCsens,* 3TCres, TFVsens.* Failure: ABCsens,* 3TCres, TFVsens.*

*Sensitive by phenotype but resistant by genotype.

(A)

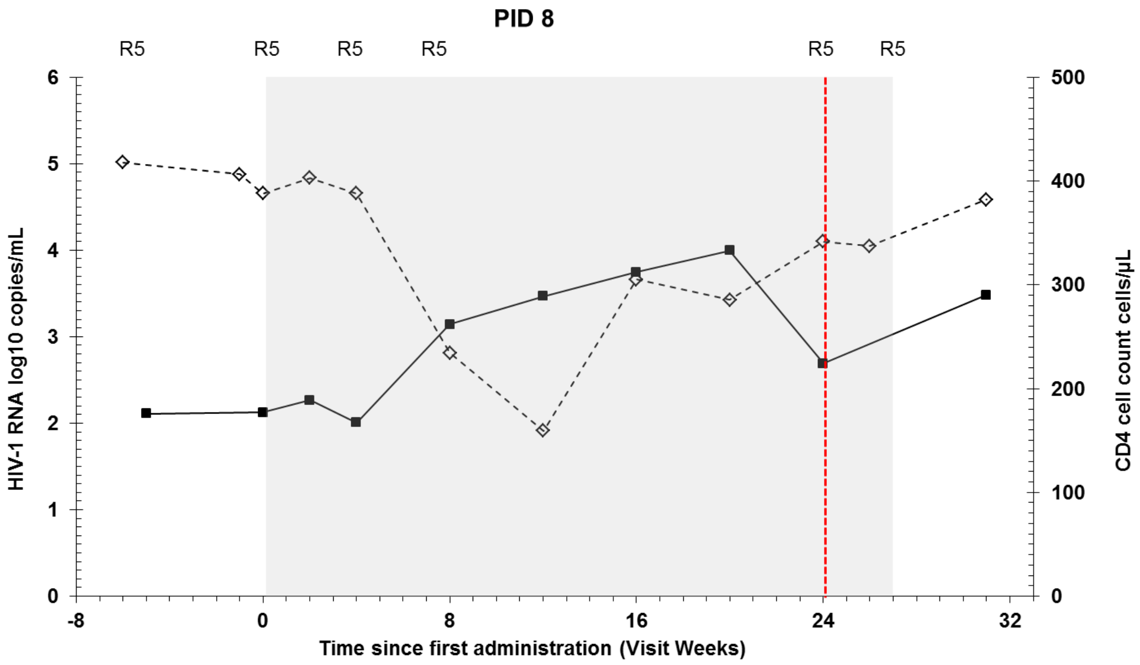
PID 8
R5
R5
R5
R5
R5
R5
HIV-1 RNA log10 copies/mL
CD4 cell count cells/μL
Time since first administration (Visit Weeks)

(B)

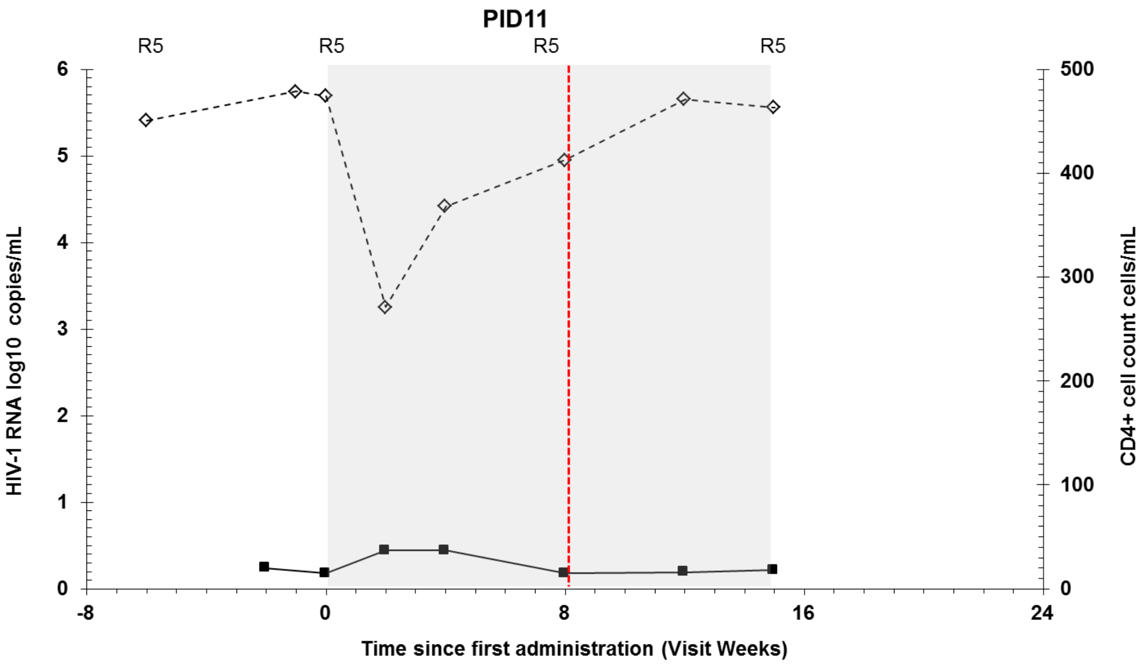
PID11
R5
R5
R5
R5
HIV-1 RNA log10 copies/mL
CD4+ cell count cells/mL
Time since first administration (Visit Weeks)

(C)

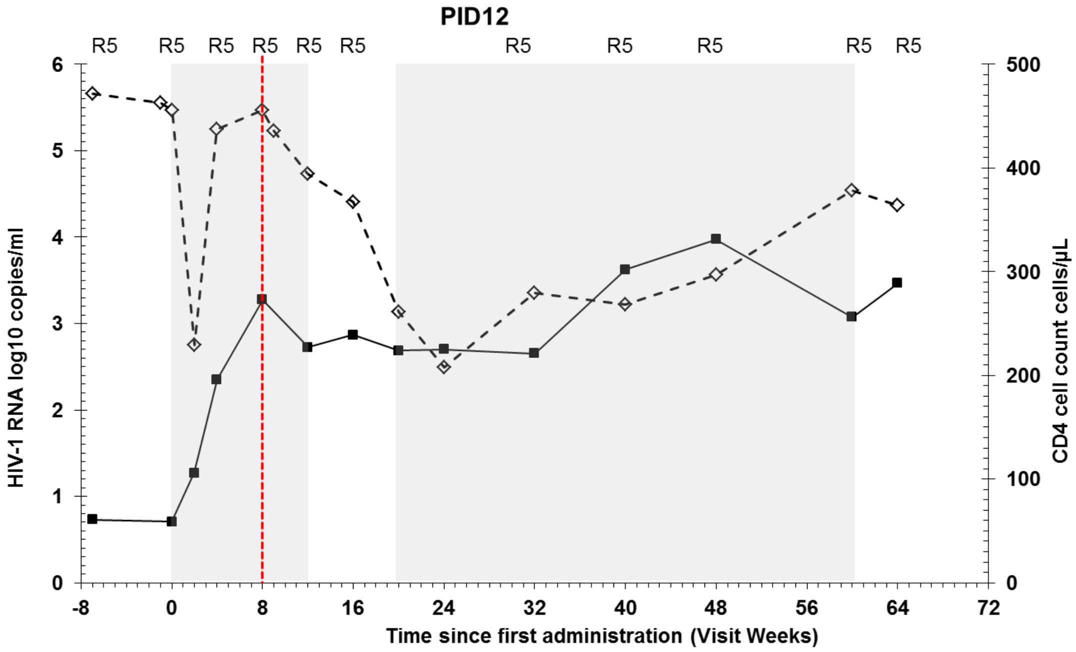

PID12
R5
R5
R5
R5
R5
R5
R5
R5
R5
R5
R5
HIV-1 RNA log10 copies/ml
CD4 cell count cells/µL
0
1
2
3
4
5
6
0
100
200
300
400
500
-8
0
8
16
24
32
40
48
56
64
72
Time since first administration (Visit Weeks)

(D)

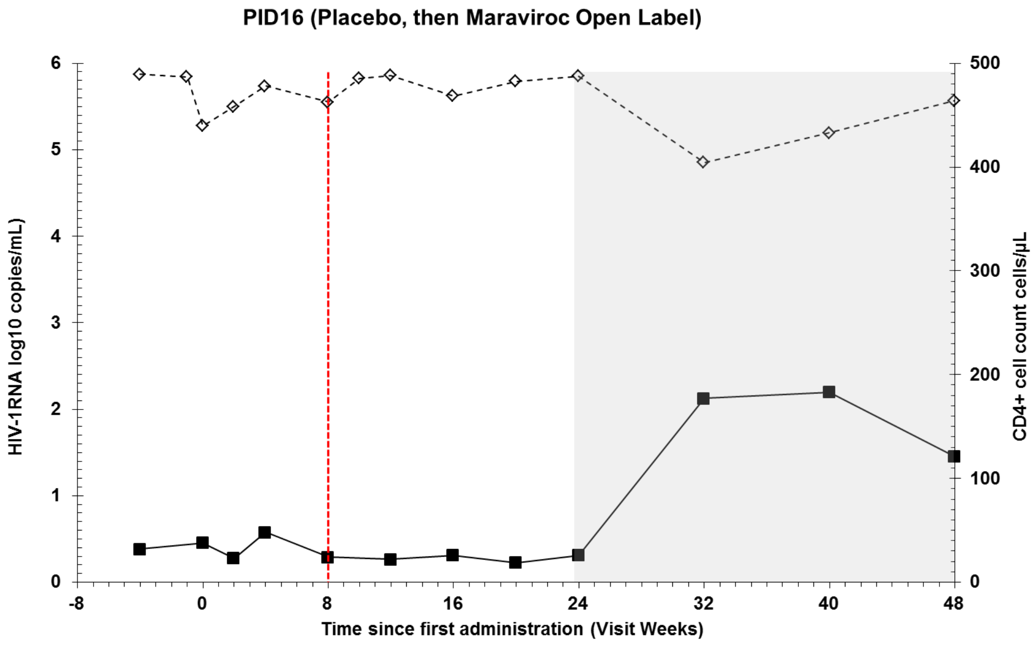

PID16 (Placebo, then Maraviroc Open Label)
HIV-1RNA log10 copies/mL)
CD4+ cell count cells/µL
0
1
2
3
4
5
6
0
100
200
300
400
500
-8
0
8
16
24
32
40
48
Time since first administration (Visit Weeks)
