# Supplementary material for: Clonal analysis of HIV-1 genotype and function associated with virologic failure in treatment-experienced persons receiving maraviroc: Results from the MOTIVATE phase 3 randomized, placebo-controlled trials
Source: PLoS One. 2018 Dec 26;13(12):e0204099. doi: 10.1371/journal.pone.0204099 (PMC6306210; doi:10.1371/journal.pone.0204099)
Supplement: S1 Table — (DOCX) [file pone.0204099.s006.docx]

**S1 Table. Demographic Characteristics of 58 Participants Studied in This Analysis Together With the Characteristics for all 267 Participants Included in the Population Subset and the Full Participant Population in the MOTIVATE Trials (N=1049)**

|  | Characteristic | Full participant population, N=1049,  n (%) | Subset population, n=267,  n (%) | Total participants included in virology analysis,  n=58, n (%) |
| --- | --- | --- | --- | --- |
| Sex | Male  Female | 930 (88.7)^a^  119 (11.3)^a^ | 248 (92.9)  19 (7.1) | 55 (94.8)  3 (5.2) |
| Race | White  Black  Other or unspecified | 877 (83.6)^a^  147 (14.0)^a^  25 (2.4)^a^ | 233 (87.3)  27 (10.1)  7 (2.6) | 47 (81.0)  11 (19.0)  0 (0) |
| Subtype | B  non-B  NR^b^ | 996 (95.1)  42 (4.0)  9 (0.9) | 253 (94.8)  13 (4.9)  1 (0.4) | 55 (94.8)  2 (3.4)  1 (1.7) |
| Plasma HIV-1 RNA | ≥100,000 copies/mL  <100,000 copies/mL | 438 (41.8)^c^ 611 (58.2)^c^ | 136 (50.9) 131 (49.1) | 34 (58.6) 24 (41.4) |
| Baseline CD4 count^d^ | <50 cells/µL  >50 cells/µL | 207 (19.7)^c^  842 (80.3)^c^ | 75 (28.1)  192 (71.9) | 28 (48.3)  30 (51.7) |
| Representation from MOTIVATE trials | MOTIVATE 1  MOTIVATE 2 | 585 (55.8)^a^  464 (44.2)^a^ | 188 (70.4)  79 (29.6) | 43 (74.1)  15 (25.9) |
| Randomization | Maraviroc QD  Maraviroc BID  Placebo | 414 (39.5)^a^  426 (40.6)^a^  209 (19.9)^a^ | 101 (37.8)  111 (41.6)  55 (20.6) | 14 (24.1)  15 (25.9)  29 (50.0) |

^a^Gulick et al. [1] (One race determination was missing).

^b^Two results are missing; N=1047.

^c^Faetkenheuer et al. [2]

^d^Baseline value for each participant was calculated as the mean of two assessments.
