# Supplementary material for: Clonal analysis of HIV-1 genotype and function associated with virologic failure in treatment-experienced persons receiving maraviroc: Results from the MOTIVATE phase 3 randomized, placebo-controlled trials
Source: PLoS One. 2018 Dec 26;13(12):e0204099. doi: 10.1371/journal.pone.0204099 (PMC6306210; doi:10.1371/journal.pone.0204099)
Supplement: S2 Table — (DOCX) [file pone.0204099.s007.docx]

**S2 Table. Genotypic and Phenotypic Clonal Analysis of Virus From 20 Participants With CXCR4-Using Infection on Treatment**

**Participants Whose CXCR4-Using Virus On-Treatment Was Related to a Component of the Pre-treatment Virus Population**

| PID T6 Day 1 Screening ESTA DM | | | | | | | | |
| --- | --- | --- | --- | --- | --- | --- | --- | --- |
| **Clone ID** | **V3 sequence^a^** | **Length** | **Viral infectivity assessment, number of clones** | | | | | Genotypic prediction of tropism using geno2pheno FPR 10^b^ |
|  |  |  | **R5** | **X4** | **DU** | **NF** | **Total clones** |  |
| 48 | CTRPNNNTRRSISMGPGRTLYATGQIIGDIRQAHC | 35 | 90 | 0 | 4 | 18 | 112 | R5 |
| 112 | ...................V............... | 35 | 24 | 0 | 0 | 10 | 34 | R5 |
| 10 | .......I.G......................... | 35 | 4 | 0 | 0 | 1 | 5 | R5 |
| 105 | ..........................M........ | 35 | 5 | 0 | 0 | 0 | 5 | R5 |
| 16 | .....D............................. | 35 | 3 | 0 | 0 | 0 | 3 | R5 |
| 56 | .........KA.GI.........ER....P..... | 35 | 0 | 0 | 0 | 3 | 3 | CXCR4-USING |
| 175 | .................G................. | 35 | 3 | 0 | 0 | 0 | 3 | R5 |
| 8 | .................S................. | 35 | 2 | 0 | 0 | 1 | 3 | R5 |
| 30 | ..........C........................ | 35 | 0 | 0 | 0 | 2 | 2 | R5 |
| 129 | ..G................................ | 35 | 0 | 0 | 0 | 1 | 1 | R5 |
| 34 | ........G.......................... | 35 | 1 | 0 | 0 | 0 | 1 | R5 |
| 58 | .................G..........G...... | 35 | 0 | 0 | 0 | 1 | 1 | R5 |
| 173 | .................K................. | 35 | 1 | 0 | 0 | 0 | 1 | R5 |
| 176 | ........................L.......... | 35 | 1 | 0 | 0 | 0 | 1 | R5 |
| 97 | .......................K........... | 35 | 1 | 0 | 0 | 0 | 1 | R5 |
| 5 | ...................V...........R... | 35 | 1 | 0 | 0 | 0 | 1 | CXCR4-USING |
| 190 | ...................V.........T....R | 35 | 1 | 0 | 0 | 0 | 1 | R5 |
| 142 | ....S.........................G.... | 35 | 1 | 0 | 0 | 0 | 1 | R5 |
| 118 | R.................................. | 35 | 1 | 0 | 0 | 0 | 1 | R5 |
| Dots indicate residues identical to the major baseline sequence Envs; dashes indicate gaps.  ^a^gp120 V3 loop sequence (equivalent to position 296 to 331 in HXB2 NCBI accession number K03455).  ^b^Genotypic tropism was assessed using the Geno2Pheno analysis with cut-off <10% FPR=CXCR4-using. | | | | | | | | |

| PID T6 Week 16 | | | | | | | | |
| --- | --- | --- | --- | --- | --- | --- | --- | --- |
| **Clone ID** | **V3 sequence^a^** | **Length** | **Viral infectivity assessment, number of clones** | | | | | Genotypic prediction of tropism using geno2pheno FPR 10^b^ |
|  |  |  | **R5** | **X4** | **DU** | **NF** | **Total clones** |  |
| .4 | CTRPNNNTRKAIGIGPGRTLYATERIIGDPRQAHC | 35 | 8 | 0 | 1 | 7 | 16 | CXCR4-USING |
| 19 | .........RS.SM.........GQ....I..... | 35 | 7 | 0 | 1 | 7 | 15 | R5 |
| 12 | .......................K........... | 35 | 1 | 0 | 4 | 0 | 5 | CXCR4-USING |
| 1 | .A.......RS.SM.........GQ....I..... | 35 | 1 | 0 | 0 | 1 | 2 | R5 |
| 17 | ........G.......................... | 35 | 1 | 0 | 0 | 0 | 1 | CXCR4-USING |
| 24 | .........RS.SM.........GQ....M..... | 35 | 1 | 0 | 0 | 0 | 1 | R5 |
| 22 | ....S.........R.................... | 35 | 0 | 0 | 0 | 1 | 1 | CXCR4-USING |
| Dots indicate residues identical to the major baseline sequence Envs; dashes indicate gaps.  ^a^gp120 V3 loop sequence (equivalent to position 296 to 331 in HXB2 NCBI accession number K03455).  ^b^Genotypic tropism was assessed using the Geno2Pheno analysis with cut-off <10% FPR=CXCR4-using. | | | | | | | | |

| PID T16 Day 1 Screening ESTA DM | | | | | | | | |
| --- | --- | --- | --- | --- | --- | --- | --- | --- |
| **Clone ID** | **V3 sequence^a^** | **Length** | **Viral infectivity assessment, number of clones** | | | | | Genotypic prediction of tropism using geno2pheno FPR 10^b^ |
|  |  |  | **R5** | **X4** | **DU** | **NF** | **Total clones** |  |
| 15 | CTRPNNNTRRGIHIAPGRAFYATGDIIGDIRQAYC | 35 | 37 | 0 | 0 | 32 | 69 | R5 |
| 11 | ........................E.......... | 35 | 29 | 0 | 0 | 18 | 47 | R5 |
| 22 | ............N.G.........E.......... | 35 | 16 | 0 | 1 | 4 | 21 | R5 |
| 62 | .........K....G.........E.......... | 35 | 4 | 0 | 1 | 4 | 9 | R5 |
| 33 | .........K..............E.......... | 35 | 5 | 0 | 0 | 2 | 7 | R5 |
| 1 | ............NLG.................... | 35 | 6 | 0 | 0 | 0 | 6 | R5 |
| 45 | ............NLG.........E.......... | 35 | 2 | 0 | 0 | 3 | 5 | R5 |
| 2 | ............RVG......~.TK......R... | 34 | 0 | 0 | 0 | 2 | 2 | CXCR4-USING |
| 93 | .A..........N.G.........E.......... | 35 | 1 | 0 | 0 | 0 | 1 | R5 |
| 158 | .........G....T.................... | 35 | 1 | 0 | 0 | 0 | 1 | R5 |
| 148 | ....................F.............. | 35 | 1 | 0 | 0 | 0 | 1 | R5 |
| 150 | .............................L..... | 35 | 0 | 0 | 0 | 1 | 1 | R5 |
| Dots indicate residues identical to the major baseline sequence Envs; dashes indicate gaps.  ^a^gp120 V3 loop sequence (equivalent to position 296 to 331 in HXB2 NCBI accession number K03455).  ^b^Genotypic tropism was assessed using the Geno2Pheno analysis with cut-off <10% FPR=CXCR4-using. | | | | | | | | |

| PID T16 Week 16 | | | | | | | | |
| --- | --- | --- | --- | --- | --- | --- | --- | --- |
| **Clone ID** | **V3 sequence^a^** | **Length** | **Viral infectivity assessment, number of clones** | | | | | Genotypic prediction of tropism using geno2pheno FPR 10^b^ |
|  |  |  | **R5** | **X4** | **DU** | **NF** | **Total clones** |  |
| 2 | CTRPNNNTRRGIRVGPGRAFY~TTKIVGDIRRAYC | 34 | 0 | 0 | 14 | 20 | 34 | CXCR4-USING |
| 17 | .....................~....I........ | 34 | 0 | 0 | 2 | 4 | 6 | CXCR4-USING |
| 15 | ........G............~............. | 34 | 0 | 0 | 0 | 1 | 1 | CXCR4-USING |
| 1 | ............NI.......A.GE.I....Q... | 35 | 1 | 0 | 0 | 0 | 1 | R5 |
| 9 | .....................~.......T..... | 34 | 0 | 0 | 1 | 0 | 1 | CXCR4-USING |
| 12 | .....................~.....R....... | 34 | 0 | 0 | 0 | 1 | 1 | CXCR4-USING |
| 5 | .....................~...M......... | 34 | 0 | 0 | 0 | 1 | 1 | CXCR4-USING |
| Dots indicate residues identical to the major baseline sequence Envs; dashes indicate gaps.  ^a^gp120 V3 loop sequence (equivalent to position 296 to 331 in HXB2 NCBI accession number K03455).  ^b^Genotypic tropism was assessed using the Geno2Pheno analysis with cut-off <10% FPR=CXCR4-using. | | | | | | | | |

| PID T17 Day 1 Screening ESTA DM | | | | | | | | |
| --- | --- | --- | --- | --- | --- | --- | --- | --- |
| **Clone ID** | **V3 sequence^a^** | **Length** | **Viral infectivity assessment, number of clones** | | | | | Genotypic prediction of tropism using geno2pheno FPR 10^b^ |
|  |  |  | **R5** | **X4** | **DU** | **NF** | **Total clones** |  |
| 5 | CTRPNNNTRISIPIGPGRAFYATGDIIGDIRQAHC | 35 | 96 | 0 | 0 | 74 | 170 | R5 |
| 133 | .........R..R..Q.KP..~~~~.......... | 31 | 1 | 1 | 0 | 6 | 8 | CXCR4-USING |
| 111 | ................................T.. | 35 | 2 | 0 | 0 | 0 | 2 | R5 |
| 15 | ...............................R... | 35 | 1 | 0 | 0 | 0 | 1 | R5 |
| 29 | ..................T................ | 35 | 0 | 0 | 0 | 1 | 1 | R5 |
| 112 | .........R..R..Q.KP..~~~~.V........ | 31 | 0 | 0 | 1 | 0 | 1 | CXCR4-USING |
| Dots indicate residues identical to the major baseline sequence Envs; dashes indicate gaps.  ^a^gp120 V3 loop sequence (equivalent to position 296 to 331 in HXB2 NCBI accession number K03455).  ^b^Genotypic tropism was assessed using the Geno2Pheno analysis with cut-off <10% FPR=CXCR4-using. | | | | | | | | |

| PID T17 Week 4 | | | | | | | | |
| --- | --- | --- | --- | --- | --- | --- | --- | --- |
| **Clone ID** | **V3 sequence^a^** | **Length** | **Viral infectivity assessment, number of clones** | | | | | Genotypic prediction of tropism using geno2pheno FPR 10^b^ |
|  |  |  | **R5** | **X4** | **DU** | **NF** | **Total clones** |  |
| 9 | CTRPNNNTRRSIRIGQGKPFYIIGDIRQAHC | 31 | 1 | 13 | 1 | 5 | 20 | CXCR4-USING |
| 7 | ......................V........ | 31 | 0 | 13 | 1 | 5 | 19 | CXCR4-USING |
| 8 | ....D.................V........ | 31 | 0 | 1 | 0 | 0 | 1 | CXCR4-USING |
| 33 | .....D......................... | 31 | 0 | 1 | 0 | 0 | 1 | CXCR4-USING |
| 22 | ...........................*... | 31 | 0 | 0 | 0 | 1 | 1 | CXCR4-USING |
| 17 | .........................T..... | 31 | 0 | 0 | 0 | 1 | 1 | CXCR4-USING |
| 11 | ...............R......V........ | 31 | 0 | 1 | 0 | 0 | 1 | CXCR4-USING |
| 1 | .........S............V........ | 31 | 0 | 0 | 0 | 1 | 1 | CXCR4-USING |
| 27 | ....S.................V........ | 31 | 0 | 0 | 0 | 1 | 1 | CXCR4-USING |
| Dots indicate residues identical to the major baseline sequence Envs; dashes indicate gaps.  ^a^gp120 V3 loop sequence (equivalent to position 296 to 331 in HXB2 NCBI accession number K03455).  ^b^Genotypic tropism was assessed using the Geno2Pheno analysis with cut-off <10% FPR=CXCR4-using. | | | | | | | | |

| PID T20 Day 1 Screening ESTA DM | | | | | | | | |
| --- | --- | --- | --- | --- | --- | --- | --- | --- |
| **Clone ID** | **V3 sequence^a^** | **Length** | **Viral infectivity assessment, number of clones** | | | | | Genotypic prediction of tropism using geno2pheno FPR 10^b^ |
|  |  |  | **R5** | **X4** | **DU** | **NF** | **Total clones** |  |
| 55 | CTRPNNNTRTSIHMGPGKAFYAGSIIGDIRQAHC | 34 | 23 | 0 | 0 | 15 | 38 | R5 |
| 87 | .................R................ | 34 | 13 | 0 | 1 | 16 | 30 | R5 |
| 84 | .I................................ | 34 | 21 | 0 | 0 | 8 | 29 | R5 |
| 32 | .....................T............ | 34 | 9 | 0 | 0 | 7 | 16 | R5 |
| 129 | .....YHI.RR..I...R.....GVK......Y. | 34 | 0 | 1 | 0 | 7 | 8 | CXCR4-USING |
| 40 | ................................R. | 34 | 0 | 0 | 0 | 3 | 3 | R5 |
| 13 | .........A.......R................ | 34 | 2 | 0 | 0 | 0 | 2 | R5 |
| 105 | .I........G....................... | 34 | 1 | 0 | 0 | 0 | 1 | R5 |
| 56 | ..G..............R................ | 34 | 0 | 0 | 0 | 1 | 1 | R5 |
| 180 | .....................T......M..... | 34 | 0 | 0 | 0 | 1 | 1 | R5 |
| 184 | .................R...T............ | 34 | 1 | 0 | 0 | 0 | 1 | R5 |
| 43 | ................R................. | 34 | 1 | 0 | 0 | 0 | 1 | R5 |
| Dots indicate residues identical to the major baseline sequence Envs; dashes indicate gaps.  ^a^gp120 V3 loop sequence (equivalent to position 296 to 331 in HXB2 NCBI accession number K03455).  ^b^Genotypic tropism was assessed using the Geno2Pheno analysis with cut-off <10% FPR=CXCR4-using. | | | | | | | | |

| PID T20 Week 4 | | | | | | | | |
| --- | --- | --- | --- | --- | --- | --- | --- | --- |
| **Clone ID** | **V3 sequence^a^** | **Length** | **Viral infectivity assessment, number of clones** | | | | | Genotypic prediction of tropism using geno2pheno FPR 10^b^ |
|  |  |  | **R5** | **X4** | **DU** | **NF** | **Total clones** |  |
| 4 | CTRPNYHIRRRIHIGPGRAFYA~GGVKGDIRQAYC | 34 | 0 | 3 | 8 | 20 | 31 | CXCR4-USING |
| 6 | .I...NNT.TS..M...K....~.SII......H. | 34 | 2 | 0 | 0 | 4 | 6 | R5 |
| 24 | .....NNT.KG...........T.EII......H. | 35 | 0 | 0 | 0 | 2 | 2 | R5 |
| 23 | ......NV..............~R..N........ | 34 | 0 | 1 | 0 | 1 | 2 | CXCR4-USING |
| 22 | .....NNT.TS..M...K....~.SII......H. | 34 | 1 | 0 | 0 | 0 | 1 | R5 |
| 26 | .....NNT.TS..M........~.SII......H. | 34 | 1 | 0 | 0 | 0 | 1 | R5 |
| 13 | ......N...............~............ | 34 | 0 | 0 | 0 | 1 | 1 | CXCR4-USING |
| 14 | ......N....M..........~............ | 34 | 0 | 0 | 0 | 1 | 1 | CXCR4-USING |
| Dots indicate residues identical to the major baseline sequence Envs; dashes indicate gaps.  ^a^gp120 V3 loop sequence (equivalent to position 296 to 331 in HXB2 NCBI accession number K03455).  ^b^Genotypic tropism was assessed using the Geno2Pheno analysis with cut-off <10% FPR=CXCR4-using. | | | | | | | | |

| PID T69 Day 1 Screening ESTA DM | | | | | | | | |
| --- | --- | --- | --- | --- | --- | --- | --- | --- |
| **Clone ID** | **V3 sequence^a^** | **Length** | **Viral infectivity assessment, number of clones** | | | | | Genotypic prediction of tropism using geno2pheno FPR 10^b^ |
|  |  |  | **R5** | **X4** | **DU** | **NF** | **Total clones** |  |
| 31 | CTRPKISKGRRIPVGPGTSFYTMGVAKGDIRKAHC | 35 | 0 | 17 | 15 | 40 | 72 | CXCR4-USING |
| 2 | ....NNNTRKS.NI...RA..AT.QII....Q... | 35 | 45 | 0 | 1 | 24 | 70 | R5 |
| 182 | ....Q.............................. | 35 | 0 | 0 | 1 | 3 | 4 | CXCR4-USING |
| 191 | .............I............R........ | 35 | 1 | 0 | 2 | 0 | 3 | CXCR4-USING |
| 82 | R...NNNTRKS.NI...RA..AT.QII....Q... | 35 | 0 | 0 | 0 | 3 | 3 | R5 |
| 169 | ..........G........................ | 35 | 0 | 2 | 0 | 0 | 2 | CXCR4-USING |
| 95 | ....NNNTRKS.NI...RA..AT.QII....R... | 35 | 0 | 0 | 0 | 2 | 2 | R5 |
| 160 | .A................................. | 35 | 0 | 0 | 0 | 1 | 1 | CXCR4-USING |
| 56 | .........K......................... | 35 | 0 | 1 | 0 | 0 | 1 | CXCR4-USING |
| 157 | ..........K........................ | 35 | 0 | 0 | 0 | 1 | 1 | CXCR4-USING |
| 91 | .................I................. | 35 | 0 | 0 | 1 | 0 | 1 | CXCR4-USING |
| 16 | ........................A.......... | 35 | 0 | 0 | 1 | 0 | 1 | CXCR4-USING |
| 12 | ..............................G.... | 35 | 0 | 1 | 0 | 0 | 1 | CXCR4-USING |
| 75 | ................................... | 35 | 0 | 0 | 0 | 1 | 1 | CXCR4-USING |
| 187 | .................................Y. | 35 | 0 | 0 | 0 | 1 | 1 | CXCR4-USING |
| 131 | ..........................R........ | 35 | 0 | 0 | 1 | 0 | 1 | CXCR4-USING |
| 140 | .........................T......... | 35 | 0 | 1 | 0 | 0 | 1 | CXCR4-USING |
| 145 | ....NDNTRKS.NI...RA..AT.QII....Q... | 35 | 1 | 0 | 0 | 0 | 1 | R5 |
| 57 | ....NNNTRES.NI...RA..AT.QII....Q... | 35 | 0 | 0 | 0 | 1 | 1 | R5 |
| 97 | ....NNNTRKS.NI.L.RA..AT.QII....Q... | 35 | 0 | 0 | 0 | 1 | 1 | R5 |
| 172 | ....NNNTRKS.NI...RA..AT.QII....QV.. | 35 | 0 | 0 | 0 | 1 | 1 | R5 |
| 36 | ....NNNTRKS.NI...RA..AT.QII..M.Q... | 35 | 1 | 0 | 0 | 0 | 1 | R5 |
| 111 | ....NNNTRKS.NI...RA..AT.QII.G..Q... | 35 | 1 | 0 | 0 | 0 | 1 | R5 |
| 94 | ....SNNTRKS.NI...RA..AT.QII....Q... | 35 | 1 | 0 | 0 | 0 | 1 | R5 |
| Dots indicate residues identical to the major baseline sequence Envs; dashes indicate gaps.  ^a^gp120 V3 loop sequence (equivalent to position 296 to 331 in HXB2 NCBI accession number K03455).  ^b^Genotypic tropism was assessed using the Geno2Pheno analysis with cut-off <10% FPR=CXCR4-using. | | | | | | | | |

| PID T69 Week 8 | | | | | | | | |
| --- | --- | --- | --- | --- | --- | --- | --- | --- |
| **Clone ID** | **V3 sequence^a^** | **Length** | **Viral infectivity assessment, number of clones** | | | | | Genotypic prediction of tropism using geno2pheno FPR 10^b^ |
|  |  |  | **R5** | **X4** | **DU** | **NF** | **Total clones** |  |
| 4 | CTRPQISKGRRIPVGPGTSFYTMGVAKGDIRKAHC | 35 | 0 | 0 | 16 | 14 | 30 | CXCR4-USING |
| 1 | ....K.............................. | 35 | 0 | 0 | 4 | 4 | 8 | CXCR4-USING |
| 14 | ....K.....................R........ | 35 | 0 | 3 | 0 | 3 | 6 | CXCR4-USING |
| 9 | ..........................R........ | 35 | 0 | 1 | 0 | 0 | 1 | CXCR4-USING |
| Dots indicate residues identical to the major baseline sequence Envs; dashes indicate gaps.  ^a^gp120 V3 loop sequence (equivalent to position 296 to 331 in HXB2 NCBI accession number K03455).  ^b^Genotypic tropism was assessed using the Geno2Pheno analysis with cut-off <10% FPR=CXCR4-using. | | | | | | | | |

| PID T132 Day 1 Screening ESTA DM | | | | | | | | |
| --- | --- | --- | --- | --- | --- | --- | --- | --- |
| **Clone ID** | **V3 sequence^a^** | **Length** | **Viral infectivity assessment, number of clones** | | | | | Genotypic prediction of tropism using geno2pheno FPR 10^b^ |
|  |  |  | **R5** | **X4** | **DU** | **NF** | **Total clones** |  |
| 147 | CTRPNNNTRKSINLGPGRAFYATGDIIGDIRQAHC | 35 | 49 | 0 | 0 | 35 | 84 | R5 |
| 95 | .............I..................... | 35 | 32 | 0 | 1 | 23 | 56 | R5 |
| 11 | ......F.E.RMT.....V..T..K.V....K... | 35 | 1 | 6 | 7 | 10 | 24 | CXCR4-USING |
| 127 | ......F.E.RLT.....V..T..K.V....K... | 35 | 0 | 0 | 0 | 1 | 1 | CXCR4-USING |
| 169 | ......F.E.RMT.....V..T..K.V...GK... | 35 | 0 | 1 | 0 | 0 | 1 | CXCR4-USING |
| 188 | .............I......H.............. | 35 | 1 | 0 | 0 | 0 | 1 | R5 |
| 99 | .............I.............E....... | 35 | 1 | 0 | 0 | 0 | 1 | R5 |
| 146 | .............I...........T......... | 35 | 1 | 0 | 0 | 0 | 1 | R5 |
| 56 | ..............................G.... | 35 | 0 | 0 | 0 | 1 | 1 | R5 |
| 68 | ................................T.. | 35 | 0 | 0 | 0 | 1 | 1 | R5 |
| 45 | ............................G....Y. | 35 | 1 | 0 | 0 | 0 | 1 | R5 |
| 125 | ..........................V........ | 35 | 0 | 0 | 0 | 1 | 1 | R5 |
| 40 | ...............S................... | 35 | 1 | 0 | 0 | 0 | 1 | R5 |
| 23 | .............V..................... | 35 | 0 | 0 | 0 | 1 | 1 | R5 |
| Dots indicate residues identical to the major baseline sequence Envs; dashes indicate gaps.  ^a^gp120 V3 loop sequence (equivalent to position 296 to 331 in HXB2 NCBI accession number K03455).  ^b^Genotypic tropism was assessed using the Geno2Pheno analysis with cut-off <10% FPR=CXCR4-using. | | | | | | | | |

| PID T132 Week 8 | | | | | | | | |
| --- | --- | --- | --- | --- | --- | --- | --- | --- |
| **Clone ID** | **V3 sequence^a^** | **Length** | **Viral infectivity assessment, number of clones** | | | | | Genotypic prediction of tropism using geno2pheno FPR 10^b^ |
|  |  |  | **R5** | **X4** | **DU** | **NF** | **Total clones** |  |
| 5 | CTRPNNNTRKSINIGPGRAFYATGDIIGDIRQAHC | 35 | 15 | 0 | 0 | 8 | 23 | R5 |
| 15 | .............L..................... | 35 | 2 | 0 | 0 | 4 | 6 | R5 |
| 3 | ......F.E.RMTL....V..T..K.V....K... | 35 | 0 | 2 | 0 | 2 | 4 | CXCR4-USING |
| 1 | ..G..........L..................... | 35 | 0 | 0 | 0 | 1 | 1 | R5 |
| 17 | ........G.......................... | 35 | 0 | 0 | 0 | 1 | 1 | R5 |
| 11 | .................G................. | 35 | 0 | 0 | 0 | 1 | 1 | R5 |
| 48 | ..................V................ | 35 | 0 | 0 | 0 | 1 | 1 | R5 |
| 37 | .............LE.................... | 35 | 1 | 0 | 0 | 0 | 1 | R5 |
| 18 | .............L............V........ | 35 | 0 | 0 | 0 | 1 | 1 | R5 |
| 28 | ......S............................ | 35 | 0 | 0 | 0 | 1 | 1 | R5 |
| 43 | ....S.............................. | 35 | 0 | 0 | 0 | 1 | 1 | R5 |
| Dots indicate residues identical to the major baseline sequence Envs; dashes indicate gaps.  ^a^gp120 V3 loop sequence (equivalent to position 296 to 331 in HXB2 NCBI accession number K03455).  ^b^Genotypic tropism was assessed using the Geno2Pheno analysis with cut-off <10% FPR=CXCR4-using. | | | | | | | | |

| PID T205 Day 1 Screening ESTA DM | | | | | | | | |
| --- | --- | --- | --- | --- | --- | --- | --- | --- |
| **Clone ID** | **V3 sequence^a^** | **Length** | **Viral infectivity assessment, number of clones** | | | | | Genotypic prediction of tropism using geno2pheno FPR 10^b^ |
|  |  |  | **R5** | **X4** | **DU** | **NF** | **Total clones** |  |
| 77 | CTRPNNNTRRSISIGPGRAFYTTGDIIGDIRQAHC | 35 | 90 | 0 | 0 | 56 | 146 | R5 |
| 104 | .......K.KRVTL....V.....E.T........ | 35 | 0 | 4 | 0 | 2 | 6 | CXCR4-USING |
| 17 | ....D.............................. | 35 | 0 | 0 | 0 | 2 | 2 | R5 |
| 52 | .........K..R....A...A............. | 35 | 0 | 0 | 0 | 2 | 2 | R5 |
| 59 | .........K................R........ | 35 | 0 | 0 | 0 | 2 | 2 | R5 |
| 8 | ............P...................... | 35 | 0 | 0 | 0 | 2 | 2 | R5 |
| 117 | ..................................R | 35 | 0 | 0 | 0 | 2 | 2 | R5 |
| 47 | ................................T.. | 35 | 2 | 0 | 0 | 0 | 2 | R5 |
| 148 | ........................G.......... | 35 | 1 | 0 | 0 | 1 | 2 | R5 |
| 9 | ...........M....................... | 35 | 2 | 0 | 0 | 0 | 2 | R5 |
| 72 | .A................................. | 35 | 1 | 0 | 0 | 0 | 1 | R5 |
| 61 | .......K.KRVTL....V.....E.V........ | 35 | 0 | 0 | 1 | 0 | 1 | CXCR4-USING |
| 13 | .........K......................... | 35 | 1 | 0 | 0 | 0 | 1 | R5 |
| 156 | ..........N........................ | 35 | 1 | 0 | 0 | 0 | 1 | R5 |
| 118 | .................G................. | 35 | 0 | 0 | 0 | 1 | 1 | R5 |
| 127 | .......................E........... | 35 | 1 | 0 | 0 | 0 | 1 | CXCR4-USING |
| 94 | .............................T..... | 35 | 1 | 0 | 0 | 0 | 1 | R5 |
| 65 | ............................G...... | 35 | 0 | 0 | 0 | 1 | 1 | R5 |
| 107 | ..........................M........ | 35 | 0 | 0 | 0 | 1 | 1 | R5 |
| 144 | .........................M......... | 35 | 1 | 0 | 0 | 0 | 1 | R5 |
| 146 | .............V..................... | 35 | 0 | 0 | 0 | 1 | 1 | R5 |
| 21 | ...........L....................... | 35 | 1 | 0 | 0 | 0 | 1 | CXCR4-USING |
| 76 | ...........V....................... | 35 | 1 | 0 | 0 | 0 | 1 | R5 |
| 64 | .....T.K.KRVTL....V.....E.T........ | 35 | 0 | 0 | 1 | 0 | 1 | CXCR4-USING |
| 188 | ....S.............................. | 35 | 0 | 0 | 0 | 1 | 1 | R5 |
| Dots indicate residues identical to the major baseline sequence Envs; dashes indicate gaps.  ^a^gp120 V3 loop sequence (equivalent to position 296 to 331 in HXB2 NCBI accession number K03455).  ^b^Genotypic tropism was assessed using the Geno2Pheno analysis with cut-off <10% FPR=CXCR4-using. | | | | | | | | |

| PID T205 Week 8 | | | | | | | | |
| --- | --- | --- | --- | --- | --- | --- | --- | --- |
| **Clone ID** | **V3 sequence^a^** | **Length** | **Viral infectivity assessment, number of clones** | | | | | Genotypic prediction of tropism using geno2pheno FPR 10^b^ |
|  |  |  | **R5** | **X4** | **DU** | **NF** | **Total clones** |  |
| 19 | CTRPNNNKRKRVTLGPGRVFYTTGEIMGDIRQAHC | 35 | 0 | 7 | 3 | 2 | 12 | CXCR4-USING |
| 24 | ..........................T........ | 35 | 0 | 2 | 2 | 5 | 9 | CXCR4-USING |
| 21 | .....T....................T........ | 35 | 0 | 3 | 1 | 3 | 7 | CXCR4-USING |
| 46 | .....S....................T........ | 35 | 0 | 3 | 0 | 2 | 5 | CXCR4-USING |
| 14 | .....T............................. | 35 | 0 | 0 | 2 | 1 | 3 | CXCR4-USING |
| 37 | ....T.............................. | 35 | 0 | 1 | 1 | 0 | 2 | CXCR4-USING |
| 18 | ....D.....................T........ | 35 | 0 | 1 | 0 | 0 | 1 | CXCR4-USING |
| 16 | ......H............................ | 35 | 0 | 1 | 0 | 0 | 1 | CXCR4-USING |
| 15 | ......K.TQGIHT....~A.F.RT.I........ | 34 | 0 | 0 | 1 | 0 | 1 | CXCR4-USING |
| 36 | .................K........T........ | 35 | 0 | 1 | 0 | 0 | 1 | CXCR4-USING |
| 30 | ..............................G.... | 35 | 0 | 1 | 0 | 0 | 1 | CXCR4-USING |
| 35 | .....T....G........................ | 35 | 0 | 0 | 0 | 1 | 1 | CXCR4-USING |
| 10 | .....T....................T....R... | 35 | 0 | 1 | 0 | 0 | 1 | CXCR4-USING |
| 42 | .....T...................T......... | 35 | 0 | 0 | 0 | 1 | 1 | CXCR4-USING |
| Dots indicate residues identical to the major baseline sequence Envs; dashes indicate gaps.  ^a^gp120 V3 loop sequence (equivalent to position 296 to 331 in HXB2 NCBI accession number K03455).  ^b^Genotypic tropism was assessed using the Geno2Pheno analysis with cut-off <10% FPR=CXCR4-using. | | | | | | | | |

| PID T221 Day 1 Screening ESTA DM | | | | | | | | |
| --- | --- | --- | --- | --- | --- | --- | --- | --- |
| **Clone ID** | **V3 sequence^a^** | **Length** | **Viral infectivity assessment, number of clones** | | | | | Genotypic prediction of tropism using geno2pheno FPR 10^b^ |
|  |  |  | **R5** | **X4** | **DU** | **NF** | **Total clones** |  |
| 60 | CTRPNNNTRKGIHMGPGRAYFTGDIIGDIRQAHC | 34 | 99 | 0 | 0 | 73 | 172 | R5 |
| 4 | ........G......................... | 34 | 2 | 0 | 0 | 0 | 2 | R5 |
| 87 | .............T.................... | 34 | 0 | 0 | 0 | 2 | 2 | CXCR4-USING |
| 90 | ...L.............................. | 34 | 0 | 0 | 0 | 1 | 1 | R5 |
| 176 | ......KKTQ...T........RT.......... | 34 | 0 | 0 | 1 | 0 | 1 | CXCR4-USING |
| 152 | ............................M..... | 34 | 1 | 0 | 0 | 0 | 1 | R5 |
| 142 | ............................V..... | 34 | 1 | 0 | 0 | 0 | 1 | R5 |
| 58 | .........................V........ | 34 | 1 | 0 | 0 | 0 | 1 | R5 |
| 110 | R................................. | 34 | 0 | 0 | 0 | 1 | 1 | R5 |
| Dots indicate residues identical to the major baseline sequence Envs; dashes indicate gaps.  ^a^gp120 V3 loop sequence (equivalent to position 296 to 331 in HXB2 NCBI accession number K03455).  ^b^Genotypic tropism was assessed using the Geno2Pheno analysis with cut-off <10% FPR=CXCR4-using. | | | | | | | | |

| PID T221 Week 4 | | | | | | | | |
| --- | --- | --- | --- | --- | --- | --- | --- | --- |
| **Clone ID** | **V3 sequence^a^** | **Length** | **Viral infectivity assessment, number of clones** | | | | | Genotypic prediction of tropism using geno2pheno FPR 10^b^ |
|  |  |  | **R5** | **X4** | **DU** | **NF** | **Total clones** |  |
| 3 | CTRPNNKKTQGIHTGPGRAYFTRTIIGDIRQAHC | 34 | 0 | 0 | 10 | 21 | 31 | CXCR4-USING |
| 21 | .....K............................ | 34 | 0 | 0 | 4 | 5 | 9 | CXCR4-USING |
| 31 | .................K................ | 34 | 0 | 0 | 0 | 1 | 1 | CXCR4-USING |
| 23 | ................................Y. | 34 | 0 | 0 | 0 | 1 | 1 | CXCR4-USING |
| 41 | ............................M..... | 34 | 0 | 0 | 0 | 1 | 1 | CXCR4-USING |
| 33 | ...........................G...... | 34 | 0 | 0 | 0 | 1 | 1 | CXCR4-USING |
| 5 | ..................V............... | 34 | 0 | 0 | 0 | 1 | 1 | CXCR4-USING |
| 2 | ...........M...................... | 34 | 0 | 0 | 1 | 0 | 1 | CXCR4-USING |
| 24 | .........R........................ | 34 | 1 | 0 | 0 | 0 | 1 | CXCR4-USING |
| 14 | .....S............................ | 34 | 0 | 0 | 1 | 0 | 1 | CXCR4-USING |
| Dots indicate residues identical to the major baseline sequence Envs; dashes indicate gaps.  ^a^gp120 V3 loop sequence (equivalent to position 296 to 331 in HXB2 NCBI accession number K03455).  ^b^Genotypic tropism was assessed using the Geno2Pheno analysis with cut-off <10% FPR=CXCR4-using. | | | | | | | | |

| PID T246 Day 1 Screening ESTA DM | | | | | | | | |
| --- | --- | --- | --- | --- | --- | --- | --- | --- |
| **Clone ID** | **V3 sequence^a^** | **Length** | **Viral infectivity assessment, number of clones** | | | | | Genotypic prediction of tropism using geno2pheno FPR 10^b^ |
|  |  |  | **R5** | **X4** | **DU** | **NF** | **Total clones** |  |
| 51 | CTRPNNNTRKGIHIGPGRAFYATGDIIGDIRRAYC | 35 | 69 | 0 | 8 | 52 | 129 | CXCR4-USING |
| 28 | .........R.........V.T..K......K... | 35 | 2 | 0 | 6 | 5 | 13 | CXCR4-USING |
| 119 | ........................E.......... | 35 | 0 | 0 | 1 | 3 | 4 | CXCR4-USING |
| 68 | ....Y.Y..R.........V.T..K...N..K... | 35 | 1 | 0 | 1 | 2 | 4 | CXCR4-USING |
| 42 | ...H............................... | 35 | 0 | 0 | 0 | 2 | 2 | R5 |
| 126 | ..........................V........ | 35 | 1 | 0 | 0 | 1 | 2 | CXCR4-USING |
| 34 | ...................V.T..K......K... | 35 | 0 | 0 | 2 | 0 | 2 | CXCR4-USING |
| 55 | ...........M....................... | 35 | 0 | 0 | 0 | 2 | 2 | CXCR4-USING |
| 40 | ...........T............E.......... | 35 | 0 | 0 | 0 | 2 | 2 | CXCR4-USING |
| 140 | ..........S........................ | 35 | 2 | 0 | 0 | 0 | 2 | R5 |
| 39 | ....Y.Y.K..........V.T..K...N..K... | 35 | 0 | 0 | 1 | 1 | 2 | CXCR4-USING |
| 24 | ....Y.Y.KR.........V.T..K...N..K... | 35 | 0 | 0 | 2 | 0 | 2 | CXCR4-USING |
| 17 | ..G.....................K......K... | 35 | 0 | 0 | 0 | 1 | 1 | CXCR4-USING |
| 120 | ................................V.. | 35 | 1 | 0 | 0 | 0 | 1 | R5 |
| 93 | .............................M..... | 35 | 1 | 0 | 0 | 0 | 1 | CXCR4-USING |
| 105 | .............................V..... | 35 | 1 | 0 | 0 | 0 | 1 | CXCR4-USING |
| 112 | ........................E.....S.... | 35 | 1 | 0 | 0 | 0 | 1 | R5 |
| 41 | ........................K......K... | 35 | 0 | 0 | 0 | 1 | 1 | CXCR4-USING |
| 182 | .....................V............. | 35 | 0 | 0 | 0 | 1 | 1 | CXCR4-USING |
| 6 | ...................V.T..R......K... | 35 | 1 | 0 | 0 | 0 | 1 | CXCR4-USING |
| 157 | ..............R.................... | 35 | 1 | 0 | 0 | 0 | 1 | R5 |
| 131 | .........R......................... | 35 | 1 | 0 | 0 | 0 | 1 | R5 |
| 111 | .........R.........V....K......K... | 35 | 0 | 0 | 0 | 1 | 1 | CXCR4-USING |
| 100 | .........R.........V.T..K...N..K... | 35 | 1 | 0 | 0 | 0 | 1 | CXCR4-USING |
| 148 | ....Y.............................. | 35 | 0 | 0 | 0 | 1 | 1 | CXCR4-USING |
| 184 | ....Y.Y.KR.........V....K...N..K... | 35 | 0 | 0 | 1 | 0 | 1 | CXCR4-USING |
| 125 | R..................V.T..K......K... | 35 | 0 | 0 | 0 | 1 | 1 | CXCR4-USING |
| Dots indicate residues identical to the major baseline sequence Envs; dashes indicate gaps.  ^a^gp120 V3 loop sequence (equivalent to position 296 to 331 in HXB2 NCBI accession number K03455).  ^b^Genotypic tropism was assessed using the Geno2Pheno analysis with cut-off <10% FPR=CXCR4-using. | | | | | | | | |

| PID T246 Week 4 | | | | | | | | |
| --- | --- | --- | --- | --- | --- | --- | --- | --- |
| **Clone ID** | **V3 sequence^a^** | **Length** | **Viral infectivity assessment, number of clones** | | | | | Genotypic prediction of tropism using geno2pheno FPR 10^b^ |
|  |  |  | **R5** | **X4** | **DU** | **NF** | **Total clones** |  |
| 2 | CTRPNNNTRRGIHIGPGRAVYTTGKIIGDIRKAYC | 35 | 2 | 0 | 16 | 14 | 32 | CXCR4-USING |
| 8 | .........K.........F.A..D......R... | 35 | 3 | 0 | 1 | 0 | 4 | CXCR4-USING |
| 10 | .........K......................... | 35 | 0 | 0 | 1 | 1 | 2 | CXCR4-USING |
| 30 | .....................A............. | 35 | 0 | 0 | 1 | 1 | 2 | CXCR4-USING |
| 4 | .........K.........FCA..D......R... | 35 | 0 | 0 | 0 | 1 | 1 | CXCR4-USING |
| 39 | .........K.........F.A............. | 35 | 0 | 0 | 1 | 0 | 1 | CXCR4-USING |
| 36 | .....................AA...........R | 35 | 0 | 0 | 0 | 1 | 1 | CXCR4-USING |
| Dots indicate residues identical to the major baseline sequence Envs; dashes indicate gaps.  ^a^gp120 V3 loop sequence (equivalent to position 296 to 331 in HXB2 NCBI accession number K03455).  ^b^Genotypic tropism was assessed using the Geno2Pheno analysis with cut-off <10% FPR=CXCR4-using. | | | | | | | | |

| PID T251 Day 1 Screening ESTA R5 | | | | | | | | |
| --- | --- | --- | --- | --- | --- | --- | --- | --- |
| **Clone ID** | **V3 sequence^a^** | **Length** | **Viral infectivity assessment, number of clones** | | | | | Genotypic prediction of tropism using geno2pheno FPR 10^b^ |
|  |  |  | **R5** | **X4** | **DU** | **NF** | **Total clones** |  |
| 2 | CTRPSNNTRKGIHIGPGRAFYATDIIGDIRQAHC | 34 | 73 | 0 | 0 | 56 | 129 | R5 |
| 123 | ...........T...................... | 34 | 0 | 0 | 0 | 3 | 3 | CXCR4-USING |
| 26 | ....G............................. | 34 | 0 | 0 | 0 | 2 | 2 | R5 |
| 112 | ......D........................... | 34 | 1 | 0 | 0 | 1 | 2 | R5 |
| 184 | ................................Y. | 34 | 2 | 0 | 0 | 0 | 2 | R5 |
| 22 | ....N............................. | 34 | 1 | 0 | 0 | 0 | 1 | R5 |
| 108 | .............................G.... | 34 | 1 | 0 | 0 | 0 | 1 | R5 |
| 61 | ..............................R... | 34 | 1 | 0 | 0 | 0 | 1 | CXCR4-USING |
| 92 | ............................M..... | 34 | 1 | 0 | 0 | 0 | 1 | R5 |
| 41 | ...........................G...... | 34 | 0 | 0 | 0 | 1 | 1 | R5 |
| 124 | ........................V......... | 34 | 0 | 0 | 0 | 1 | 1 | R5 |
| 178 | ................R................. | 34 | 1 | 0 | 0 | 0 | 1 | R5 |
| 84 | .........R........................ | 34 | 0 | 0 | 0 | 1 | 1 | R5 |
| 174 | .....QKQIRR..............K.N.K.... | 34 | 0 | 0 | 0 | 1 | 1 | CXCR4-USING |
| 117 | ..S............................... | 34 | 1 | 0 | 0 | 0 | 1 | R5 |
| Dots indicate residues identical to the major baseline sequence Envs; dashes indicate gaps.  ^a^gp120 V3 loop sequence (equivalent to position 296 to 331 in HXB2 NCBI accession number K03455).  ^b^Genotypic tropism was assessed using the Geno2Pheno analysis with cut-off <10% FPR=CXCR4-using. | | | | | | | | |

| PID T251 Week 4 | | | | | | | | |
| --- | --- | --- | --- | --- | --- | --- | --- | --- |
| **Clone ID** | **V3 sequence^a^** | **Length** | **Viral infectivity assessment, number of clones** | | | | | Genotypic prediction of tropism using geno2pheno FPR 10^b^ |
|  |  |  | **R5** | **X4** | **DU** | **NF** | **Total clones** |  |
| 1 | CTRPSQKQIRRIHIGPGRAFYATDIKGNIKQAHC | 34 | 0 | 10 | 2 | 21 | 33 | CXCR4-USING |
| 38 | ...LNNNTRKSMTL.................... | 34 | 0 | 3 | 0 | 8 | 11 | R5 |
| 28 | ..G............................... | 34 | 0 | 0 | 0 | 1 | 1 | CXCR4-USING |
| 4 | .......................G.......... | 34 | 0 | 0 | 0 | 1 | 1 | CXCR4-USING |
| Dots indicate residues identical to the major baseline sequence Envs; dashes indicate gaps.  ^a^gp120 V3 loop sequence (equivalent to position 296 to 331 in HXB2 NCBI accession number K03455).  ^b^Genotypic tropism was assessed using the Geno2Pheno analysis with cut-off <10% FPR=CXCR4-using. | | | | | | | | |

| PID T285 Day 1 Screening ESTA DM | | | | | | | | |
| --- | --- | --- | --- | --- | --- | --- | --- | --- |
| **Clone ID** | **V3 sequence^a^** | **Length** | **Viral infectivity assessment, number of clones** | | | | | Genotypic prediction of tropism using geno2pheno FPR 10^b^ |
|  |  |  | **R5** | **X4** | **DU** | **NF** | **Total clones** |  |
| 4 | CTRLNNNTRRSITIGPGRAFYTSDIIGNIRQAHC | 34 | 74 | 0 | 4 | 48 | 126 | R5 |
| 21 | .....................A.....D...... | 34 | 9 | 1 | 0 | 18 | 28 | R5 |
| 126 | ...........................D...... | 34 | 5 | 0 | 0 | 3 | 8 | R5 |
| 54 | .........K.M.L...KV...TGT......... | 34 | 0 | 0 | 3 | 1 | 4 | CXCR4-USING |
| 88 | ................................R. | 34 | 2 | 0 | 0 | 0 | 2 | R5 |
| 40 | ..............................R... | 34 | 2 | 0 | 0 | 0 | 2 | R5 |
| 16 | .....D............................ | 34 | 1 | 0 | 0 | 0 | 1 | R5 |
| 37 | ..........G....................... | 34 | 1 | 0 | 0 | 0 | 1 | R5 |
| 140 | ..........R....................... | 34 | 0 | 0 | 0 | 1 | 1 | CXCR4-USING |
| 170 | ............I..............D...... | 34 | 1 | 0 | 0 | 0 | 1 | R5 |
| 10 | ..............E................... | 34 | 0 | 0 | 0 | 1 | 1 | R5 |
| 162 | ...............L.................. | 34 | 1 | 0 | 0 | 0 | 1 | R5 |
| 188 | .................................R | 34 | 0 | 0 | 0 | 1 | 1 | R5 |
| 148 | ............................M..... | 34 | 1 | 0 | 0 | 0 | 1 | R5 |
| 176 | .........................T........ | 34 | 1 | 0 | 0 | 0 | 1 | R5 |
| 102 | ......S................N.......... | 34 | 1 | 0 | 0 | 0 | 1 | R5 |
| 167 | ....S............................. | 34 | 0 | 0 | 0 | 1 | 1 | R5 |
| 118 | ...P.............................. | 34 | 1 | 0 | 0 | 0 | 1 | R5 |
| Dots indicate residues identical to the major baseline sequence Envs; dashes indicate gaps.  ^a^gp120 V3 loop sequence (equivalent to position 296 to 331 in HXB2 NCBI accession number K03455).  ^b^Genotypic tropism was assessed using the Geno2Pheno analysis with cut-off <10% FPR=CXCR4-using. | | | | | | | | |

| PID T285 Week 4 | | | | | | | | |
| --- | --- | --- | --- | --- | --- | --- | --- | --- |
| **Clone ID** | **V3 sequence^a^** | **Length** | **Viral infectivity assessment, number of clones** | | | | | Genotypic prediction of tropism using geno2pheno FPR 10^b^ |
|  |  |  | **R5** | **X4** | **DU** | **NF** | **Total clones** |  |
| 1 | CTRLNNNTRKSMTLGPGKVFYTTGTIGNIRQAHC | 34 | 0 | 1 | 21 | 19 | 41 | CXCR4-USING |
| 18 | ......................A........... | 34 | 0 | 0 | 0 | 1 | 1 | CXCR4-USING |
| 30 | .......................D.......... | 34 | 0 | 0 | 1 | 0 | 1 | CXCR4-USING |
| 27 | ...............................T.. | 34 | 0 | 0 | 0 | 1 | 1 | CXCR4-USING |
| 16 | R................................. | 34 | 0 | 0 | 1 | 0 | 1 | CXCR4-USING |
| Dots indicate residues identical to the major baseline sequence Envs; dashes indicate gaps.  ^a^gp120 V3 loop sequence (equivalent to position 296 to 331 in HXB2 NCBI accession number K03455).  ^b^Genotypic tropism was assessed using the Geno2Pheno analysis with cut-off <10% FPR=CXCR4-using. | | | | | | | | |

| PID T347 Day 1 Screening ESTA DM | | | | | | | | |
| --- | --- | --- | --- | --- | --- | --- | --- | --- |
| **Clone ID** | **V3 sequence^a^** | **Length** | **Viral infectivity assessment, number of clones** | | | | | Genotypic prediction of tropism using geno2pheno FPR 10^b^ |
|  |  |  | **R5** | **X4** | **DU** | **NF** | **Total clones** |  |
| 16 | CTRPNNNTRRSIPIGPGRAFYATGDIIGDIRQAHC | 35 | 120 | 0 | 1 | 38 | 159 | R5 |
| 38 | .....................T............. | 35 | 3 | 0 | 0 | 0 | 3 | R5 |
| 45 | .A..G.......S.......F.............. | 35 | 2 | 0 | 0 | 0 | 2 | R5 |
| 14 | .I.......KRVTM....VW.T..E.....KK.Y. | 35 | 0 | 0 | 2 | 0 | 2 | CXCR4-USING |
| 1 | .................G................. | 35 | 0 | 0 | 0 | 2 | 2 | R5 |
| 58 | ..................................R | 35 | 1 | 0 | 0 | 1 | 2 | R5 |
| 189 | ..G................................ | 35 | 0 | 0 | 0 | 1 | 1 | R5 |
| 157 | ...L............................... | 35 | 0 | 0 | 0 | 1 | 1 | R5 |
| 50 | ....D.............................. | 35 | 1 | 0 | 0 | 0 | 1 | R5 |
| 159 | ....H.............................. | 35 | 0 | 0 | 0 | 1 | 1 | R5 |
| 178 | .........KG.N......W....E...N...... | 35 | 1 | 0 | 0 | 0 | 1 | R5 |
| 148 | .........K......................... | 35 | 1 | 0 | 0 | 0 | 1 | R5 |
| 46 | ......................S............ | 35 | 1 | 0 | 0 | 0 | 1 | R5 |
| 90 | ..........................T........ | 35 | 0 | 0 | 0 | 1 | 1 | R5 |
| Dots indicate residues identical to the major baseline sequence Envs; dashes indicate gaps.  ^a^gp120 V3 loop sequence (equivalent to position 296 to 331 in HXB2 NCBI accession number K03455).  ^b^Genotypic tropism was assessed using the Geno2Pheno analysis with cut-off <10% FPR=CXCR4-using. | | | | | | | | |

| PID T347 Week 8 | | | | | | | | |
| --- | --- | --- | --- | --- | --- | --- | --- | --- |
| **Clone ID** | **V3 sequence^a^** | **Length** | **Viral infectivity assessment, number of clones** | | | | | Genotypic prediction of tropism using geno2pheno FPR 10^b^ |
|  |  |  | **R5** | **X4** | **DU** | **NF** | **Total clones** |  |
| 13 | CTRPNNNTRRSIPIGPGRAFYATGDIIGDIRQAHC | 35 | 13 | 0 | 0 | 9 | 22 | R5 |
| 1 | .I........RVTM....VW.T..K.....KK.Y. | 35 | 1 | 1 | 6 | 4 | 12 | CXCR4-USING |
| 11 | .I........RVAM....VW.T..K.....KK.Y. | 35 | 0 | 0 | 0 | 1 | 1 | CXCR4-USING |
| 19 | .....D............................. | 35 | 0 | 0 | 0 | 1 | 1 | R5 |
| 22 | ......D............................ | 35 | 0 | 0 | 0 | 1 | 1 | R5 |
| 3 | .............................T..... | 35 | 1 | 0 | 0 | 0 | 1 | R5 |
| 5 | ..........................M........ | 35 | 1 | 0 | 0 | 0 | 1 | R5 |
| 32 | .................S................. | 35 | 0 | 0 | 0 | 1 | 1 | R5 |
| 30 | ...............S................... | 35 | 0 | 0 | 0 | 1 | 1 | R5 |
| Dots indicate residues identical to the major baseline sequence Envs; dashes indicate gaps.  ^a^gp120 V3 loop sequence (equivalent to position 296 to 331 in HXB2 NCBI accession number K03455).  ^b^Genotypic tropism was assessed using the Geno2Pheno analysis with cut-off <10% FPR=CXCR4-using. | | | | | | | | |

| PID T377 Day 1 Screening ESTA DM | | | | | | | | |
| --- | --- | --- | --- | --- | --- | --- | --- | --- |
| **Clone ID** | **V3 sequence^a^** | **Length** | **Viral infectivity assessment, number of clones** | | | | | Genotypic prediction of tropism using geno2pheno FPR 10^b^ |
|  |  |  | **R5** | **X4** | **DU** | **NF** | **Total clones** |  |
| 74 | CIRPNNNTRKSINIGPGRAFYAADQIIGDIRQAHC | 35 | 76 | 0 | 1 | 33 | 110 | R5 |
| 41 | .T................................. | 35 | 11 | 0 | 0 | 7 | 18 | R5 |
| 72 | .V................................. | 35 | 13 | 0 | 1 | 4 | 18 | R5 |
| 58 | .T.LSSY.KRR.Q.....S.HT.K.VK..L..... | 35 | 0 | 1 | 2 | 1 | 4 | CXCR4-USING |
| 10 | ..............................G.... | 35 | 1 | 0 | 0 | 2 | 3 | R5 |
| 35 | ............................G...... | 35 | 1 | 0 | 0 | 1 | 2 | R5 |
| 120 | ..K................................ | 35 | 1 | 0 | 0 | 0 | 1 | R5 |
| 185 | ....D.............................. | 35 | 0 | 0 | 0 | 1 | 1 | R5 |
| 66 | .....D..G.......................... | 35 | 0 | 0 | 0 | 1 | 1 | R5 |
| 53 | .....D............................. | 35 | 1 | 0 | 0 | 0 | 1 | R5 |
| 62 | ..........G........................ | 35 | 1 | 0 | 0 | 0 | 1 | R5 |
| 115 | .........................M......... | 35 | 0 | 0 | 0 | 1 | 1 | R5 |
| 155 | ...................L......M........ | 35 | 1 | 0 | 0 | 0 | 1 | R5 |
| 64 | ....S.............................. | 35 | 0 | 0 | 0 | 1 | 1 | R5 |
| 167 | .T.LSSY.KRR.Q.....S..T.KEVQ..L..... | 35 | 0 | 1 | 0 | 0 | 1 | CXCR4-USING |
| 30 | .T.LSSY.KRR.Q.....S..T.REVQ..L..... | 35 | 0 | 1 | 0 | 0 | 1 | CXCR4-USING |
| 152 | .T..........D...................... | 35 | 0 | 0 | 0 | 1 | 1 | R5 |
| 65 | .T.............................*... | 35 | 1 | 0 | 0 | 0 | 1 | R5 |
| 154 | .T................................ | 35 | 0 | 0 | 0 | 1 | 1 | R5 |
| 63 | .VG................................ | 35 | 0 | 0 | 0 | 1 | 1 | R5 |
| 170 | .V.....A..G........................ | 35 | 1 | 0 | 0 | 0 | 1 | R5 |
| 181 | .V..........D...................... | 35 | 1 | 0 | 0 | 0 | 1 | R5 |
| 11 | .V...............G................. | 35 | 0 | 0 | 0 | 1 | 1 | R5 |
| Dots indicate residues identical to the major baseline sequence Envs; dashes indicate gaps.  ^a^gp120 V3 loop sequence (equivalent to position 296 to 331 in HXB2 NCBI accession number K03455).  ^b^Genotypic tropism was assessed using the Geno2Pheno analysis with cut-off <10% FPR=CXCR4-using. | | | | | | | | |

| PID T377 Week 4 | | | | | | | | |
| --- | --- | --- | --- | --- | --- | --- | --- | --- |
| **Clone ID** | **V3 sequence^a^** | **Length** | **Viral infectivity assessment, number of clones** | | | | | Genotypic prediction of tropism using geno2pheno FPR 10^b^ |
|  |  |  | **R5** | **X4** | **DU** | **NF** | **Total clones** |  |
| 1 | CTRLSSYTKRRIQIGPGRSFYTAKEVQGDLRQAHC | 35 | 1 | 20 | 0 | 15 | 36 | CXCR4-USING |
| 10 | ........................Q.E........ | 35 | 0 | 0 | 0 | 2 | 2 | CXCR4-USING |
| 37 | ..G................................ | 35 | 0 | 1 | 0 | 0 | 1 | CXCR4-USING |
| 34 | ....N...................Q.E........ | 35 | 0 | 0 | 0 | 1 | 1 | CXCR4-USING |
| 9 | ......H............................ | 35 | 0 | 1 | 0 | 0 | 1 | CXCR4-USING |
| 7 | ..........G........................ | 35 | 0 | 1 | 0 | 0 | 1 | CXCR4-USING |
| 47 | .....................AT............ | 35 | 0 | 1 | 0 | 0 | 1 | CXCR4-USING |
| 28 | ............R...........Q.E........ | 35 | 0 | 0 | 0 | 1 | 1 | CXCR4-USING |
| 16 | ...P............................... | 35 | 0 | 0 | 0 | 1 | 1 | CXCR4-USING |
| Dots indicate residues identical to the major baseline sequence Envs; dashes indicate gaps.  ^a^gp120 V3 loop sequence (equivalent to position 296 to 331 in HXB2 NCBI accession number K03455).  ^b^Genotypic tropism was assessed using the Geno2Pheno analysis with cut-off <10% FPR=CXCR4-using. | | | | | | | | |

| PID T397 Day 1 Screening ESTA DM | | | | | | | | |
| --- | --- | --- | --- | --- | --- | --- | --- | --- |
| **Clone ID** | **V3 sequence^a^** | **Length** | **Viral infectivity assessment, number of clones** | | | | | Genotypic prediction of tropism using geno2pheno FPR 10^b^ |
|  |  |  | **R5** | **X4** | **DU** | **NF** | **Total clones** |  |
| 1 | CTRPGNNTSKSISIGPGRAFYARERIIGNIRQAHC | 35 | 36 | 0 | 28 | 42 | 106 | CXCR4-USING |
| 41 | .........RRV........L.......D.KK... | 35 | 3 | 0 | 6 | 10 | 19 | CXCR4-USING |
| 3 | ............P...............D....Y. | 35 | 9 | 0 | 0 | 5 | 14 | CXCR4-USING |
| 52 | .........RR..L..............D.KK... | 35 | 1 | 0 | 0 | 7 | 8 | CXCR4-USING |
| 12 | .........................V......... | 35 | 1 | 0 | 3 | 2 | 6 | CXCR4-USING |
| 185 | .I................................. | 35 | 0 | 0 | 2 | 0 | 2 | CXCR4-USING |
| 5 | ........................GV......... | 35 | 1 | 0 | 0 | 1 | 2 | CXCR4-USING |
| 44 | .................................Y. | 35 | 2 | 0 | 0 | 0 | 2 | CXCR4-USING |
| 115 | ..............................S.... | 35 | 2 | 0 | 0 | 0 | 2 | CXCR4-USING |
| 154 | ............................S...... | 35 | 2 | 0 | 0 | 0 | 2 | CXCR4-USING |
| 2 | .................................L. | 35 | 1 | 0 | 0 | 0 | 1 | CXCR4-USING |
| 107 | ................................S.. | 35 | 1 | 0 | 0 | 0 | 1 | CXCR4-USING |
| 18 | ................................T.. | 35 | 0 | 0 | 0 | 1 | 1 | CXCR4-USING |
| 63 | ..........................M........ | 35 | 1 | 0 | 0 | 0 | 1 | CXCR4-USING |
| 29 | .........RR..L.......V......D.KK... | 35 | 0 | 0 | 0 | 1 | 1 | CXCR4-USING |
| 162 | .........RRV........L.....V.D.KK... | 35 | 0 | 0 | 0 | 1 | 1 | CXCR4-USING |
| 163 | .........RRV........LV......D.KK... | 35 | 0 | 0 | 0 | 1 | 1 | CXCR4-USING |
| 17 | .........RRV.M......L.......D.KK... | 35 | 0 | 0 | 0 | 1 | 1 | CXCR4-USING |
| 16 | ......S............................ | 35 | 0 | 0 | 0 | 1 | 1 | CXCR4-USING |
| Dots indicate residues identical to the major baseline sequence Envs; dashes indicate gaps.  ^a^gp120 V3 loop sequence (equivalent to position 296 to 331 in HXB2 NCBI accession number K03455).  ^b^Genotypic tropism was assessed using the Geno2Pheno analysis with cut-off <10% FPR=CXCR4-using. | | | | | | | | |

| PID T397 Week 4 | | | | | | | | |
| --- | --- | --- | --- | --- | --- | --- | --- | --- |
| **Clone ID** | **V3 sequence^a^** | **Length** | **Viral infectivity assessment, number of clones** | | | | | Genotypic prediction of tropism using geno2pheno FPR 10^b^ |
|  |  |  | **R5** | **X4** | **DU** | **NF** | **Total clones** |  |
| 1 | CTRPGNNTSKSISIGPGRAFYARERIIGNIRQAHC | 35 | 4 | 0 | 6 | 9 | 19 | CXCR4-USING |
| 14 | .........RR...............T........ | 35 | 1 | 0 | 14 | 3 | 18 | CXCR4-USING |
| 6 | .........RR...............T......R. | 35 | 0 | 0 | 2 | 0 | 2 | CXCR4-USING |
| 38 | .I.......RR...............T........ | 35 | 0 | 0 | 0 | 1 | 1 | CXCR4-USING |
| 41 | ......D............................ | 35 | 1 | 0 | 0 | 0 | 1 | CXCR4-USING |
| 43 | ............................D...... | 35 | 0 | 0 | 0 | 1 | 1 | CXCR4-USING |
| 7 | ..................V................ | 35 | 1 | 0 | 0 | 0 | 1 | CXCR4-USING |
| 21 | .........RR...............T........ | 35 | 0 | 0 | 0 | 1 | 1 | CXCR4-USING |
| Dots indicate residues identical to the major baseline sequence Envs; dashes indicate gaps.  ^a^gp120 V3 loop sequence (equivalent to position 296 to 331 in HXB2 NCBI accession number K03455).  ^b^Genotypic tropism was assessed using the Geno2Pheno analysis with cut-off <10% FPR=CXCR4-using. | | | | | | | | |

**Participants Whose CXCR4-Using Virus On-Treatment Was Not Related to a Component of the Pre-treatment Virus Population**

| PID T57 Day 1 Screening ESTA R5 | | | | | | | | |
| --- | --- | --- | --- | --- | --- | --- | --- | --- |
| **Clone ID** | **V3 sequence^a^** | **Length** | **Viral infectivity assessment, number of clones** | | | | | Genotypic prediction of tropism using geno2pheno FPR 10^b^ |
|  |  |  | **R5** | **X4** | **DU** | **NF** | **Total clones** |  |
| 5 | CTRPNNNTRKSLNMGPGRAIYATGDIIGDIRQAHC | 35 | 59 | 1 | 0 | 64 | 124 | CXCR4-USING |
| 57 | ....................F.............. | 35 | 11 | 0 | 0 | 7 | 18 | CXCR4-USING |
| 86 | ..............E.................... | 35 | 9 | 0 | 1 | 1 | 11 | CXCR4-USING |
| 63 | ...............................R... | 35 | 6 | 0 | 0 | 0 | 6 | CXCR4-USING |
| 2 | ...S............................... | 35 | 1 | 0 | 0 | 2 | 3 | CXCR4-USING |
| 84 | ............S...................... | 35 | 2 | 0 | 0 | 0 | 2 | CXCR4-USING |
| 160 | .......A........................... | 35 | 0 | 0 | 0 | 1 | 1 | CXCR4-USING |
| 77 | ..........G........................ | 35 | 1 | 0 | 0 | 0 | 1 | CXCR4-USING |
| 148 | ..........G..............T......... | 35 | 0 | 0 | 0 | 1 | 1 | CXCR4-USING |
| 181 | ............D.......F.............. | 35 | 1 | 0 | 0 | 0 | 1 | CXCR4-USING |
| 67 | ..............E..G................. | 35 | 1 | 0 | 0 | 0 | 1 | CXCR4-USING |
| 7 | ...................F............... | 35 | 1 | 0 | 0 | 0 | 1 | R5 |
| 69 | ....................F....V......... | 35 | 1 | 0 | 0 | 0 | 1 | CXCR4-USING |
| 68 | .............................M..... | 35 | 1 | 0 | 0 | 0 | 1 | CXCR4-USING |
| 44 | ................R.................. | 35 | 0 | 0 | 0 | 1 | 1 | CXCR4-USING |
| 59 | ...........P..E.................... | 35 | 1 | 0 | 0 | 0 | 1 | R5 |
| Dots indicate residues identical to the major baseline sequence Envs; dashes indicate gaps.  ^a^gp120 V3 loop sequence (equivalent to position 296 to 331 in HXB2 NCBI accession number K03455).  ^b^Genotypic tropism was assessed using the Geno2Pheno analysis with cut-off <10% FPR=CXCR4-using. | | | | | | | | |

| PID T57 E_Term (Week 24) | | | | | | | | |
| --- | --- | --- | --- | --- | --- | --- | --- | --- |
| **Clone ID** | **V3 sequence^a^** | **Length** | **Viral infectivity assessment, number of clones** | | | | | Genotypic prediction of tropism using geno2pheno FPR 10^b^ |
|  |  |  | **R5** | **X4** | **DU** | **NF** | **Total clones** |  |
| 3 | CTRPNNYTRKSVRMGLGRSFYARKAIIGDIRQAYC | 35 | 0 | 12 | 1 | 8 | 21 | CXCR4-USING |
| 1 | ......N....LN..P..AI..TGD........H. | 35 | 16 | 0 | 0 | 2 | 18 | CXCR4-USING |
| 32 | ......N....LN..P.GAI..TGD........H. | 35 | 1 | 0 | 0 | 0 | 1 | CXCR4-USING |
| 14 | .....T...................Q.......... | 36 | 0 | 0 | 0 | 1 | 1 | CXCR4-USING |
| Dots indicate residues identical to the major baseline sequence Envs; dashes indicate gaps.  ^a^gp120 V3 loop sequence (equivalent to position 296 to 331 in HXB2 NCBI accession number K03455).  ^b^Genotypic tropism was assessed using the Geno2Pheno analysis with cut-off <10% FPR=CXCR4-using. | | | | | | | | |

| PID T210 Day 1 Screening ESTA DM | | | | | | | | |
| --- | --- | --- | --- | --- | --- | --- | --- | --- |
| **Clone ID** | **V3 sequence^a^** | **Length** | **Viral infectivity assessment, number of clones** | | | | | Genotypic prediction of tropism using geno2pheno FPR 10^b^ |
|  |  |  | **R5** | **X4** | **DU** | **NF** | **Total clones** |  |
| 64 | CTRPNNNTRKSISIGPGRAFYATGGIIGDIRKAYC | 35 | 50 | 0 | 0 | 44 | 94 | R5 |
| 35 | ............T...................... | 35 | 48 | 0 | 0 | 21 | 69 | R5 |
| 8 | ....HHTV.RR.H........~.TSA.SGP.YVH. | 34 | 0 | 1 | 0 | 2 | 3 | CXCR4-USING |
| 78 | ..........R.T...................... | 35 | 2 | 0 | 0 | 0 | 2 | CXCR4-USING |
| 25 | ....HHTVKRR.H........~.TSA.SGP.YVH. | 34 | 0 | 0 | 1 | 0 | 1 | CXCR4-USING |
| 157 | ........G.......................... | 35 | 1 | 0 | 0 | 0 | 1 | R5 |
| 136 | ................................V.. | 35 | 0 | 0 | 0 | 1 | 1 | R5 |
| 69 | .......................R........... | 35 | 0 | 0 | 0 | 1 | 1 | CXCR4-USING |
| 137 | ............T...............G...... | 35 | 1 | 0 | 0 | 0 | 1 | R5 |
| 128 | .........T.....................R... | 35 | 0 | 0 | 0 | 1 | 1 | CXCR4-USING |
| Dots indicate residues identical to the major baseline sequence Envs; dashes indicate gaps.  ^a^gp120 V3 loop sequence (equivalent to position 296 to 331 in HXB2 NCBI accession number K03455).  ^b^Genotypic tropism was assessed using the Geno2Pheno analysis with cut-off <10% FPR=CXCR4-using. | | | | | | | | |

| PID T210 Week 4 | | | | | | | | |
| --- | --- | --- | --- | --- | --- | --- | --- | --- |
| **Clone ID** | **V3 sequence^a^** | **Length** | **Viral infectivity assessment, number of clones** | | | | | Genotypic prediction of tropism using geno2pheno FPR 10^b^ |
|  |  |  | **R5** | **X4** | **DU** | **NF** | **Total clones** |  |
| 2 | CTRPYETQIKRRIYIGQGRAFSATKQVVGDPRKAYC | 36 | 0 | 17 | 4 | 19 | 40 | CXCR4-USING |
| 16 | .......H............................ | 36 | 0 | 0 | 0 | 2 | 2 | CXCR4-USING |
| 32 | ..........................I......... | 36 | 0 | 0 | 0 | 2 | 2 | CXCR4-USING |
| 20 | ..................K................. | 36 | 0 | 1 | 0 | 0 | 1 | CXCR4-USING |
| 15 | ................R................... | 36 | 0 | 0 | 0 | 1 | 1 | CXCR4-USING |
| Dots indicate residues identical to the major baseline sequence Envs; dashes indicate gaps.  ^a^gp120 V3 loop sequence (equivalent to position 296 to 331 in HXB2 NCBI accession number K03455).  ^b^Genotypic tropism was assessed using the Geno2Pheno analysis with cut-off <10% FPR=CXCR4-using. | | | | | | | | |

| PID T398 Day 1 Screening ESTA R5 | | | | | | | | | |
| --- | --- | --- | --- | --- | --- | --- | --- | --- | --- |
| **Clone ID** | **V3 sequence^a^** | **Length** | | **Viral infectivity assessment, number of clones** | | | | | Genotypic prediction of tropism using geno2pheno FPR 10^b^ |
|  |  |  |  | **R5** | **X4** | **DU** | **NF** | **Total clones** |  |
| 33 | CTRPNNNTRKSIPMGPGQAIYATGAIIGDIRQAHC | | 35 | 89 | 0 | 2 | 18 | 109 | R5 |
| 144 | ............................N...... | | 35 | 46 | 0 | 0 | 4 | 50 | R5 |
| 91 | .....D............................. | | 35 | 2 | 0 | 0 | 0 | 2 | R5 |
| 26 | .....D......................N...... | | 35 | 2 | 0 | 0 | 0 | 2 | R5 |
| 73 | ................................V.. | | 35 | 0 | 0 | 0 | 2 | 2 | R5 |
| 31 | ........................T.......... | | 35 | 1 | 0 | 0 | 1 | 2 | R5 |
| 183 | ..........CL..................T..L. | | 35 | 1 | 0 | 0 | 0 | 1 | CXCR4-USING |
| 182 | .................H................. | | 35 | 1 | 0 | 0 | 0 | 1 | R5 |
| 44 | ...............................R... | | 35 | 0 | 0 | 0 | 1 | 1 | R5 |
| 169 | ...........M...............RN.T.... | | 35 | 1 | 0 | 0 | 0 | 1 | R5 |
| 111 | ...........T....................... | | 35 | 1 | 0 | 0 | 0 | 1 | R5 |
| 8 | R.................................. | | 35 | 1 | 0 | 0 | 0 | 1 | R5 |
| Dots indicate residues identical to the major baseline sequence Envs; dashes indicate gaps.  ^a^gp120 V3 loop sequence (equivalent to position 296 to 331 in HXB2 NCBI accession number K03455).  ^b^Genotypic tropism was assessed using the Geno2Pheno analysis with cut-off <10% FPR=CXCR4-using. | | | | | | | | | |

| PID T398 Week 4 | | | | | | | | |
| --- | --- | --- | --- | --- | --- | --- | --- | --- |
| **Clone ID** | **V3 sequence^a^** | **Length** | **Viral infectivity assessment, number of clones** | | | | | Genotypic prediction of tropism using geno2pheno FPR 10^b^ |
|  |  |  | **R5** | **X4** | **DU** | **NF** | **Total clones** |  |
| 1 | CTRPNNNTRRKIHIGPRHGQVMYATEIIGNIRQAHC | 36 | 0 | 2 | 15 | 20 | 37 | CXCR4-USING |
| 42 | ..........................T......... | 36 | 0 | 0 | 1 | 1 | 2 | CXCR4-USING |
| 37 | ......................H............. | 36 | 0 | 0 | 1 | 0 | 1 | CXCR4-USING |
| 35 | ..................................Y. | 36 | 0 | 0 | 0 | 1 | 1 | CXCR4-USING |
| 38 | .....................V.............. | 36 | 0 | 0 | 0 | 1 | 1 | CXCR4-USING |
| 9 | ...........V........................ | 36 | 0 | 0 | 0 | 1 | 1 | CXCR4-USING |
| 14 | .....T...................Q.......... | 36 | 0 | 0 | 0 | 1 | 1 | CXCR4-USING |
| Dots indicate residues identical to the major baseline sequence Envs; dashes indicate gaps.  ^a^gp120 V3 loop sequence (equivalent to position 296 to 331 in HXB2 NCBI accession number K03455).  ^b^Genotypic tropism was assessed using the Geno2Pheno analysis with cut-off <10% FPR=CXCR4-using. | | | | | | | | |

| PID T415 Day 1 Screening ESTA R5 | | | | | | | | |
| --- | --- | --- | --- | --- | --- | --- | --- | --- |
| **Clone ID** | **V3 sequence^a^** | **Length** | **Viral infectivity assessment, number of clones** | | | | | Genotypic prediction of tropism using geno2pheno FPR 10^b^ |
|  |  |  | **R5** | **X4** | **DU** | **NF** | **Total clones** |  |
| 77 | CTRHNNNTRKSINIGPGRAWYTTGDITGDIRQAYC | 35 | 76 | 0 | 0 | 64 | 140 | R5 |
| 112 | .........R......................... | 35 | 10 | 0 | 0 | 8 | 18 | R5 |
| 177 | ...............................K... | 35 | 1 | 0 | 0 | 1 | 2 | R5 |
| 83 | .................................H. | 35 | 1 | 0 | 0 | 1 | 2 | R5 |
| 152 | ...............................R... | 35 | 2 | 0 | 0 | 0 | 2 | R5 |
| 87 | ............................N...... | 35 | 0 | 0 | 0 | 2 | 2 | R5 |
| 191 | ....D.............................. | 35 | 0 | 0 | 0 | 1 | 1 | R5 |
| 6 | ......D............................ | 35 | 0 | 0 | 0 | 1 | 1 | R5 |
| 122 | .......A........................... | 35 | 0 | 0 | 0 | 1 | 1 | R5 |
| 61 | .......A.....M..................... | 35 | 0 | 0 | 0 | 1 | 1 | R5 |
| 125 | ..........G........................ | 35 | 0 | 0 | 0 | 1 | 1 | R5 |
| 23 | ....................H.............. | 35 | 0 | 0 | 0 | 1 | 1 | R5 |
| 188 | ......................I............ | 35 | 1 | 0 | 0 | 0 | 1 | R5 |
| 29 | ...........................E....... | 35 | 1 | 0 | 0 | 0 | 1 | R5 |
| 133 | ...........................R....... | 35 | 0 | 0 | 0 | 1 | 1 | R5 |
| 120 | .........................S......... | 35 | 1 | 0 | 0 | 0 | 1 | R5 |
| 33 | .........................V......... | 35 | 1 | 0 | 0 | 0 | 1 | R5 |
| 44 | .............V..................... | 35 | 0 | 0 | 0 | 1 | 1 | R5 |
| 143 | .........R...............V......... | 35 | 0 | 0 | 0 | 1 | 1 | R5 |
| 96 | ...Y............................... | 35 | 1 | 0 | 0 | 0 | 1 | R5 |
| 97 | S.................................. | 35 | 0 | 0 | 0 | 1 | 1 | R5 |
| Dots indicate residues identical to the major baseline sequence Envs; dashes indicate gaps.  ^a^gp120 V3 loop sequence (equivalent to position 296 to 331 in HXB2 NCBI accession number K03455).  ^b^Genotypic tropism was assessed using the Geno2Pheno analysis with cut-off <10% FPR=CXCR4-using. | | | | | | | | |

| PID T415 Week 4 | | | | | | | | |
| --- | --- | --- | --- | --- | --- | --- | --- | --- |
| **Clone ID** | **V3 sequence^a^** | **Length** | **Viral infectivity assessment, number of clones** | | | | | Genotypic prediction of tropism using geno2pheno FPR 10^b^ |
|  |  |  | **R5** | **X4** | **DU** | **NF** | **Total clones** |  |
| 3 | CTRPGTKMIMRSMHIGPGRVFLTKDIEGDIRKASC | 35 | 0 | 11 | 1 | 17 | 29 | CXCR4-USING |
| 24 | .......~........................... | 34 | 1 | 0 | 0 | 6 | 7 | CXCR4-USING |
| 10 | ..G....~........................... | 34 | 0 | 0 | 0 | 1 | 1 | CXCR4-USING |
| 12 | .....A.~........................... | 34 | 0 | 1 | 0 | 0 | 1 | CXCR4-USING |
| 14 | ..........................G........ | 35 | 0 | 1 | 0 | 0 | 1 | CXCR4-USING |
| 25 | ....................L.............. | 35 | 0 | 0 | 0 | 1 | 1 | CXCR4-USING |
| 6 | .........T......................... | 35 | 0 | 0 | 0 | 1 | 1 | CXCR4-USING |
| Dots indicate residues identical to the major baseline sequence Envs; dashes indicate gaps.  ^a^gp120 V3 loop sequence (equivalent to position 296 to 331 in HXB2 NCBI accession number K03455).  ^b^Genotypic tropism was assessed using the Geno2Pheno analysis with cut-off <10% FPR=CXCR4-using. | | | | | | | | |

| PID T629 Day 1 Screening ESTA DM | | | | | | | | |
| --- | --- | --- | --- | --- | --- | --- | --- | --- |
| **Clone ID** | **V3 sequence^a^** | **Length** | **Viral infectivity assessment, number of clones** | | | | | Genotypic prediction of tropism using geno2pheno FPR 10^b^ |
|  |  |  | **R5** | **X4** | **DU** | **NF** | **Total clones** |  |
| 28 | CVRPNNNTVKGIHIGPGRAFYTTGQIVGNIRQAHC | 35 | 108 | 0 | 5 | 38 | 151 | R5 |
| 192 | ............................D...... | 35 | 3 | 0 | 0 | 3 | 6 | R5 |
| 29 | ....................D.............. | 35 | 1 | 0 | 0 | 2 | 3 | R5 |
| 105 | ........G...............R........R. | 35 | 2 | 0 | 0 | 0 | 2 | R5 |
| 55 | ........................E.......... | 35 | 2 | 0 | 0 | 0 | 2 | R5 |
| 33 | .........................M......... | 35 | 2 | 0 | 0 | 0 | 2 | R5 |
| 70 | .A................................. | 35 | 1 | 0 | 0 | 0 | 1 | R5 |
| 96 | .......I........................... | 35 | 1 | 0 | 0 | 0 | 1 | CXCR4-USING |
| 85 | ........I.......................... | 35 | 1 | 0 | 0 | 0 | 1 | R5 |
| 14 | ...............L................... | 35 | 1 | 0 | 0 | 0 | 1 | R5 |
| 24 | ........................L.......... | 35 | 1 | 0 | 0 | 0 | 1 | R5 |
| 140 | ..............................G.... | 35 | 1 | 0 | 0 | 0 | 1 | R5 |
| 17 | ...........M....................... | 35 | 0 | 0 | 0 | 1 | 1 | R5 |
| Dots indicate residues identical to the major baseline sequence Envs; dashes indicate gaps.  ^a^gp120 V3 loop sequence (equivalent to position 296 to 331 in HXB2 NCBI accession number K03455).  ^b^Genotypic tropism was assessed using the Geno2Pheno analysis with cut-off <10% FPR=CXCR4-using. | | | | | | | | |

| PID T629 Week 4 | | | | | | | | |
| --- | --- | --- | --- | --- | --- | --- | --- | --- |
| **Clone ID** | **V3 sequence^a^** | **Length** | **Viral infectivity assessment, number of clones** | | | | | Genotypic prediction of tropism using geno2pheno FPR 10^b^ |
|  |  |  | **R5** | **X4** | **DU** | **NF** | **Total clones** |  |
| 8 | CARPNNNTVKGIGIGPGRTVYTAEKIVGNIRQAHC | 35 | 0 | 1 | 9 | 10 | 20 | CXCR4-USING |
| 24 | .........R......................... | 35 | 0 | 1 | 7 | 12 | 20 | CXCR4-USING |
| 13 | ....D.............................. | 35 | 0 | 0 | 1 | 0 | 1 | CXCR4-USING |
| Dots indicate residues identical to the major baseline sequence Envs; dashes indicate gaps.  ^a^gp120 V3 loop sequence (equivalent to position 296 to 331 in HXB2 NCBI accession number K03455).  ^b^Genotypic tropism was assessed using the Geno2Pheno analysis with cut-off <10% FPR=CXCR4-using. | | | | | | | | |

| PID T825 Day 1 Screening ESTA DM | | | | | | | | |
| --- | --- | --- | --- | --- | --- | --- | --- | --- |
| **Clone ID** | **V3 sequence^a^** | **Length** | **Viral infectivity assessment, number of clones** | | | | | Genotypic prediction of tropism using geno2pheno FPR 10^b^ |
|  |  |  | **R5** | **X4** | **DU** | **NF** | **Total clones** |  |
| 2 | CTRPSNNTRKGIHIGPGSTFFATDIIGDIRQAHC | 34 | 71 | 0 | 34 | 66 | 171 | R5 |
| 128 | .................R................ | 34 | 7 | 0 | 0 | 6 | 13 | R5 |
| 70 | .............M.................... | 34 | 1 | 0 | 0 | 0 | 1 | R5 |
| 165 | .........R.......R................ | 34 | 0 | 0 | 0 | 1 | 1 | R5 |
| Dots indicate residues identical to the major baseline sequence Envs; dashes indicate gaps.  ^a^gp120 V3 loop sequence (equivalent to position 296 to 331 in HXB2 NCBI accession number K03455).  ^b^Genotypic tropism was assessed using the Geno2Pheno analysis with cut-off <10% FPR=CXCR4-using. | | | | | | | | |

| PID T825 Week 4 | | | | | | | | |
| --- | --- | --- | --- | --- | --- | --- | --- | --- |
| **Clone ID** | **V3 sequence^a^** | **Length** | **Viral infectivity assessment, number of clones** | | | | | Genotypic prediction of tropism using geno2pheno FPR 10^b^ |
|  |  |  | **R5** | **X4** | **DU** | **NF** | **Total clones** |  |
| 3 | CIRPGNNTKKFIHLGPRRGAFYATHNIGDARQAHC | 35 | 0 | 3 | 6 | 6 | 15 | CXCR4-USING |
| 1 | WI................................. | 35 | 0 | 0 | 0 | 4 | 4 | CXCR4-USING |
| 47 | ......S............................ | 35 | 0 | 1 | 1 | 0 | 2 | CXCR4-USING |
| 27 | ...L............................... | 35 | 0 | 0 | 0 | 1 | 1 | CXCR4-USING |
| 44 | ...........V....................... | 35 | 0 | 0 | 0 | 1 | 1 | CXCR4-USING |
| 21 | .........R......................... | 35 | 0 | 1 | 0 | 0 | 1 | CXCR4-USING |
| 12 | ......T............................ | 35 | 0 | 0 | 1 | 0 | 1 | CXCR4-USING |
| 41 | ......T...................V........ | 35 | 0 | 0 | 0 | 1 | 1 | CXCR4-USING |
| 33 | RI............................*.... | 35 | 0 | 0 | 0 | 1 | 1 | CXCR4-USING |
| Dots indicate residues identical to the major baseline sequence Envs; dashes indicate gaps.  ^a^gp120 V3 loop sequence (equivalent to position 296 to 331 in HXB2 NCBI accession number K03455).  ^b^Genotypic tropism was assessed using the Geno2Pheno analysis with cut-off <10% FPR=CXCR4-using. | | | | | | | | |
