# Supplementary material for: Clonal analysis of HIV-1 genotype and function associated with virologic failure in treatment-experienced persons receiving maraviroc: Results from the MOTIVATE phase 3 randomized, placebo-controlled trials
Source: PLoS One. 2018 Dec 26;13(12):e0204099. doi: 10.1371/journal.pone.0204099 (PMC6306210; doi:10.1371/journal.pone.0204099)
Supplement: S3 Table — (DOCX) [file pone.0204099.s008.docx]

**S3 Table. Genotypic and Phenotypic Analysis of 12 Clones From Virus Pre- and Post-treatment, From 20 Participants With CXCR4-Using Infection on Treatment**

**Participants Whose CXCR4-Using Virus On-Treatment Was Related to a Component of the Pre-treatment Virus Population**

| PID T6 Day 1 | | | | | | | | | |
| --- | --- | --- | --- | --- | --- | --- | --- | --- | --- |
| **Clone** | **V3 sequence^a^** | **Confirmation assay**  **Trofile** | | | **Pre-screening**  **infectivity assessment** | | | **Genotypic prediction^b^** | |
|  |  | **Reported tropism** | **R5 RLU** | **X4 RLU** | **Result** | **R5 score** | **X4 score** | **11/25 rule** | **g2p 10% FPR** |
| 48 | CTRPNNNTRRSISMGPGRTLYATGQIIGDIRQAHC | R5 | 2651948 | 163 | R5 | 5 | 0 | N (S/Q) | R5 |
| 79 | ................................... | DU | 1737638 | 523 | DU | 5 | 1 | N (S/Q) | R5 |
| 80 | ................................... | R5 | 210770 | 92 | R5 | 3 | 0 | N (S/Q) | R5 |
| 100 | ................................... | DU | 1592706 | 935 | DU | 5 | 1 | N (S/Q) | R5 |
| 105 | ..........................M........ | R5 | 2455579 | 81 | R5 | 4 | 0 | N (S/Q) | R5 |
| 112 | ...................V............... | R5 | 5687 | 82 | R5 | 2 | 0 | N (S/Q) | R5 |
| 114 | ................................... | R5 | 297959 | 78 | R5 | 4 | 0 | N (S/Q) | R5 |
| 126 | ................................... | DU | 2149690 | 236 | DU | 4 | 1 | N (S/Q) | R5 |
| 155 | ................................... | R5 | 1432920 | 130 | DU | 5 | 1 | N (S/Q) | R5 |
| 175 | .................G................. | R5 | 2193879 | 72 | R5 | 5 | 0 | N (S/Q) | R5 |
| 183 | ................................... | R5 | 1370030 | 93 | R5 | 4 | 0 | N (S/Q) | R5 |
| 187 | ...................V............... | F | 469 | 98 | R5 | 2 | 0 | N (S/Q) | R5 |
| Dots indicate residues identical to the major baseline sequence Envs; dashes indicate gaps.  ^a^gp120 V3 loop sequence (equivalent to position 296 to 331 in HXB2 NCBI accession number K03455).  ^b^Genotypic tropism was assessed using the 11/25 rule and the Geno2Pheno algorithm with cut-off <10% FPR=CXCR4-using**.** | | | | | | | | | |

| PID T6 Week 16 | | | | | | | | | |
| --- | --- | --- | --- | --- | --- | --- | --- | --- | --- |
| **Clone** | **V3 sequence^a^** | **Confirmation assay**  **Trofile** | | | **Pre-screening**  **infectivity assessment** | | | **Genotypic prediction^b^** | |
|  |  | **Reported tropism** | **R5 RLU** | **X4 RLU** | **Result** | **R5 score** | **X4 score** | **11/25 rule** | **g2p 10% FPR** |
| 4 | CTRPNNNTRKAIGIGPGRTLYATERIIGDPRQAHC | F | 356 | 60 | R5 | 2 | 0 | Y (A/R) | CXCR4-USING |
| 7 | ................................... | R5 | 9689 | 68 | R5 | 3 | 0 | Y (A/R) | CXCR4-USING |
| 12 | .......................K........... | DU | 939 | 74714 | DU | 1 | 3 | Y (A/R) | CXCR4-USING |
| 15 | ................................... | F | 144 | 62 | R5 | 2 | 0 | Y (A/R) | CXCR4-USING |
| 19 | .........RS.SM.........GQ....I..... | DU | 2149447 | 548 | DU | 5 | 1 | N (S/Q) | R5 |
| 26 | .......................K........... | F | 101 | 61 | R5 | 2 | 0 | Y (A/R) | CXCR4-USING |
| 39 | .......................K........... | DU | 58161 | 561780 | DU | 3 | 3 | Y (A/R) | CXCR4-USING |
| 40 | .........RS.SM.........GQ....I..... | R5 | 1915586 | 181 | R5 | 4 | 0 | N (S/Q) | R5 |
| 41 | .........RS.SM.........GQ....I..... | R5 | 9139 | 64 | R5 | 2 | 0 | N (S/Q) | R5 |
| 42 | .......................K........... | DU | 2028 | 28008 | DU | 2 | 2 | Y (A/R) | CXCR4-USING |
| 45 | ................................... | DU | 1035 | 45608 | DU | 2 | 2 | Y (A/R) | CXCR4-USING |
| 48 | .......................K........... | DU | 137354 | 448541 | DU | 3 | 3 | Y (A/R) | CXCR4-USING |
| Dots indicate residues identical to the major baseline sequence Envs; dashes indicate gaps.  ^a^gp120 V3 loop sequence (equivalent to position 296 to 331 in HXB2 NCBI accession number K03455).  ^b^Genotypic tropism was assessed using the 11/25 rule and the Geno2Pheno algorithm with cut-off <10% FPR=CXCR4-using**.** | | | | | | | | | |

| PID T16 Day 1 | | | | | | | | | | |
| --- | --- | --- | --- | --- | --- | --- | --- | --- | --- | --- |
| **Clone** | **V3 sequence^a^** | **Confirmation assay**  **Trofile** | | | **Pre-screening**  **infectivity assessment** | | | **Genotypic prediction^b^** | | |
|  |  | **Reported tropism** | **R5 RLU** | **X4 RLU** | **Result** | **R5 score** | **X4 score** | **11/25 rule** | | **g2p 10% FPR** |
| 11 | CTRPNNNTRRGIHIAPGRAFYATGEIIGDIRQAYC | R5 | 734524 | 94 | R5 | 4 | 0 | N (G/E) | R5 | |
| 15 | ........................D.......... | R5 | 1833 | 71 | R5 | 2 | 0 | N (G/D) | R5 | |
| 22 | ............N.G.................... | R5 | 1397934 | 65 | R5 | 4 | 0 | N (G/E) | R5 | |
| 37 | ........................D.......... | R5 | 444926 | 88 | R5 | 4 | 0 | N (G/D) | R5 | |
| 48 | ........................D.......... | R5 | 855441 | 73 | R5 | 5 | 0 | N (G/D) | R5 | |
| 62 | .........K....G.................... | DU | 2222 | 2037 | DU | 2 | 1 | N (G/E) | R5 | |
| 73 | ............N.G.................... | R5 | 1892384 | 80 | R5 | 5 | 0 | N (G/E) | R5 | |
| 107 | ............N.G.................... | R5 | 444321 | 72 | DU | 4 | 1 | N (G/E) | R5 | |
| 117 | ........................D.......... | R5 | 634090 | 68 | R5 | 4 | 0 | N (G/D) | R5 | |
| 119 | ........................D.......... | R5 | 10049 | 65 | R5 | 3 | 0 | N (G/D) | R5 | |
| 154 | ................................... | R5 | 73682 | 53 | R5 | 3 | 0 | N (G/E) | R5 | |
| 187 | ........................D.......... | R5 | 3015422 | 72 | R5 | 5 | 0 | N (G/D) | R5 | |
| Dots indicate residues identical to the major baseline sequence Envs; dashes indicate gaps.  ^a^gp120 V3 loop sequence (equivalent to position 296 to 331 in HXB2 NCBI accession number K03455).  ^b^Genotypic tropism was assessed using the 11/25 rule and the Geno2Pheno algorithm with cut-off <10% FPR=CXCR4-using**.** | | | | | | | | | | |

| PID T16 Week 16 | | | | | | | | | |
| --- | --- | --- | --- | --- | --- | --- | --- | --- | --- |
| **Clone** | **V3 sequence^a^** | **Confirmation assay**  **Trofile** | | | **Pre-screening**  **infectivity assessment** | | | **Genotypic prediction^b^** | |
|  |  | **Reported tropism** | **R5 RLU** | **X4 RLU** | **Result** | **R5 score** | **X4 score** | **11/25 rule** | **g2p 10% FPR** |
| 1 | CTRPNNNTRRGINIGPGRAFYATGEIIGDIRQAYC | R5 | 2733588 | 110 | R5 | 5 | 0 | N (G/E) | R5 |
| 2 | ............RV.......T.~K.V....R... | DU | 8056 | 18233 | DU | 3 | 3 | Y (G/K) | CXCR4-USING |
| 6 | ............RV.......T.~K.V....R... | DU | 422200 | 406475 | DU | 4 | 4 | Y (G/K) | CXCR4-USING |
| 8 | ............RV.......T.~K.V....R... | DU | 677294 | 595126 | DU | 5 | 4 | Y (G/K) | CXCR4-USING |
| 9 | ............RV.......T.~K.V..T.R... | DU | 18110 | 16916 | DU | 3 | 3 | Y (G/K) | CXCR4-USING |
| 17 | ............RV.......T.~K......R... | DU | 404695 | 441134 | DU | 4 | 4 | Y (G/K) | CXCR4-USING |
| 24 | ............RV.......T.~K.V....R... | DU | 950224 | 913011 | DU | 5 | 4 | Y (G/K) | CXCR4-USING |
| 25 | ............RV.......T.~K.V....R... | DU | 308217 | 359305 | DU | 4 | 3 | Y (G/K) | CXCR4-USING |
| 29 | ............RV.......T.~K.V....R... | DU | 812777 | 899342 | DU | 4 | 4 | Y (G/K) | CXCR4-USING |
| 39 | ............RV.......T.~K.V....R... | DU | 391845 | 364566 | DU | 4 | 4 | Y (G/K) | CXCR4-USING |
| 44 | ............RV.......T.~K.V....R... | DU | 233727 | 204841 | DU | 4 | 3 | Y (G/K) | CXCR4-USING |
| 47 | ............RV.......T.~K......R... | DU | 30703 | 25170 | DU | 3 | 2 | Y (G/K) | CXCR4-USING |
| Dots indicate residues identical to the major baseline sequence Envs; dashes indicate gaps.  ^a^gp120 V3 loop sequence (equivalent to position 296 to 331 in HXB2 NCBI accession number K03455).  ^b^Genotypic tropism was assessed using the 11/25 rule and the Geno2Pheno algorithm with cut-off <10% FPR=CXCR4-using**.** | | | | | | | | | |

| PID T17 Day 1 | | | | | | | | | |
| --- | --- | --- | --- | --- | --- | --- | --- | --- | --- |
| **Clone** | **V3 sequence^a^** | **Confirmation assay**  **Trofile** | | | **Pre-screening**  **infectivity assessment** | | | **Genotypic prediction^b^** | |
|  |  | **Reported tropism** | **R5 RLU** | **X4 RLU** | **Result** | **R5 score** | **X4 score** | **11/25 rule** | **g2p 10% FPR** |
| 5 | CTRPNNNTRISIPIGPGRAFYATGDIIGDIRQAHC | R5 | 2224727 | 111 | R5 | 5 | 0 | N (S/D) | R5 |
| 43 | ................................... | R5 | 79983 | 96 | R5 | 3 | 0 | N (S/D) | R5 |
| 61 | ................................... | R5 | 19071 | 96 | R5 | 3 | 0 | N (S/D) | R5 |
| 85 | ................................... | R5 | 2306 | 95 | R5 | 2 | 0 | N (S/D) | R5 |
| 105 | ................................... | R5 | 161776 | 103 | R5 | 4 | 0 | N (S/D) | R5 |
| 111 | ................................T.. | R5 | 176111 | 104 | R5 | 4 | 0 | N (S/D) | R5 |
| 112 | .........R..R..Q.KP..~~~~.V........ | DU | 706 | 103656 | DU | 1 | 3 | N (S/-) | CXCR4-USING |
| 130 | ................................... | R5 | 1671497 | 95 | R5 | 5 | 0 | N (S/D) | R5 |
| 133 | .........R..R..Q.KP..~~~~.......... | F | 679 | 97 | R5 | 1 | 0 | N (S/-) | CXCR4-USING |
| 158 | .........R..R..Q.KP..~~~~.......... | X4 | 78 | 82067 | X4 | 0 | 3 | N (S/-) | CXCR4-USING |
| 176 | ................................... | R5 | 624301 | 93 | R5 | 4 | 0 | N (S/D) | R5 |
| 180 | ................................... | R5 | 794856 | 90 | R5 | 5 | 0 | N (S/D) | R5 |
| Dots indicate residues identical to the major baseline sequence Envs; dashes indicate gaps.  ^a^gp120 V3 loop sequence (equivalent to position 296 to 331 in HXB2 NCBI accession number K03455).  ^b^Genotypic tropism was assessed using the 11/25 rule and the Geno2Pheno algorithm with cut-off <10% FPR=CXCR4-using**.** | | | | | | | | | |

| PID T17 Week 4 | | | | | | | | | |
| --- | --- | --- | --- | --- | --- | --- | --- | --- | --- |
| **Clone** | **V3 sequence^a^** | **Confirmation assay**  **Trofile** | | | **Pre-screening**  **infectivity assessment** | | | **Genotypic prediction^b^** | |
|  |  | **Reported tropism** | **R5 RLU** | **X4 RLU** | **Result** | **R5 score** | **X4 score** | **11/25 rule** | **g2p 10% FPR** |
| 7 | CTRPNNNTRRSIRIGQGKPFYIVGDIRQAHC | X4 | 72 | 30227 | X4 | 0 | 3 | N (S/-) | CXCR4-USING |
| 8 | ....D.......................... | X4 | 88 | 26091 | X4 | 0 | 2 | N (S/-) | CXCR4-USING |
| 9 | ......................I........ | F | 58 | 963 | X4 | 0 | 1 | N (S/-) | CXCR4-USING |
| 11 | ...............R............... | X4 | 70 | 4864 | X4 | 0 | 2 | N (S/-) | CXCR4-USING |
| 16 | ......................I........ | X4 | 56 | 450651 | X4 | 0 | 4 | N (S/-) | CXCR4-USING |
| 20 | ............................... | X4 | 86 | 172637 | X4 | 0 | 4 | N (S/-) | CXCR4-USING |
| 25 | ............................... | X4 | 92 | 292334 | X4 | 0 | 4 | N (S/-) | CXCR4-USING |
| 32 | ......................I........ | F | 579 | 661 | R5 | 2 | 0 | N (S/-) | CXCR4-USING |
| 33 | .....D................I........ | X4 | 53 | 223354 | X4 | 0 | 4 | N (S/-) | CXCR4-USING |
| 37 | ......................I........ | DU | 205 | 5947 | DU | 1 | 2 | N (S/-) | CXCR4-USING |
| 40 | ......................I........ | X4 | 61 | 14351 | X4 | 0 | 3 | N (S/-) | CXCR4-USING |
| 44 | ............................... | F | 192 | 927 | DU | 1 | 1 | N (S/-) | CXCR4-USING |
| Dots indicate residues identical to the major baseline sequence Envs; dashes indicate gaps.  ^a^gp120 V3 loop sequence (equivalent to position 296 to 331 in HXB2 NCBI accession number K03455).  ^b^Genotypic tropism was assessed using the 11/25 rule and the Geno2Pheno algorithm with cut-off <10% FPR=CXCR4-using**.** | | | | | | | | | |

| PID T20 Day 1 | | | | | | | | | |
| --- | --- | --- | --- | --- | --- | --- | --- | --- | --- |
| **Clone** | **V3 sequence^a^** | **Confirmation assay**  **Trofile** | | | **Pre-screening**  **infectivity assessment** | | | **Genotypic prediction^b^** | |
|  |  | **Reported tropism** | **R5 RLU** | **X4 RLU** | **Result** | **R5 score** | **X4 score** | **11/25 rule** | **g2p 10% FPR** |
| 32 | CTRPNNNTRTSIHMGPGKAFYTGSIIGDIRQAHC | R5 | 1665801 | 76 | R5 | 4 | 0 | N (S/S) | R5 |
| 55 | .....................A............ | R5 | 570394 | 77 | R5 | 4 | 0 | N (S/S) | R5 |
| 84 | .I...................A............ | R5 | 928109 | 85 | R5 | 4 | 0 | N (S/S) | R5 |
| 87 | .................R...A............ | R5 | 2456 | 60 | R5 | 2 | 0 | N (S/S) | R5 |
| 101 | .I...................A............ | R5 | 298336 | 93 | R5 | 4 | 0 | N (S/S) | R5 |
| 105 | .I........G..........A............ | R5 | 4769 | 50 | R5 | 3 | 0 | N (G/S) | R5 |
| 129 | .....YHI.RR..I...R...A.GVK......Y. | F | 70 | 65 | X4 | 0 | 1 | Y (R/G) | CXCR4-USING |
| 130 | .....................A............ | R5 | 1050234 | 85 | R5 | 4 | 0 | N (S/S) | R5 |
| 143 | .................................. | R5 | 1580835 | 115 | R5 | 5 | 0 | N (S/S) | R5 |
| 147 | .I...................A............ | R5 | 2717580 | 100 | R5 | 5 | 0 | N (S/S) | R5 |
| 175 | .................R...A............ | R5 | 127256 | 94 | R5 | 4 | 0 | N (S/S) | R5 |
| 179 | .................R...A............ | R5 | 1071005 | 80 | DU | 4 | 1 | N (S/S) | R5 |
| Dots indicate residues identical to the major baseline sequence Envs; dashes indicate gaps.  ^a^gp120 V3 loop sequence (equivalent to position 296 to 331 in HXB2 NCBI accession number K03455).  ^b^Genotypic tropism was assessed using the 11/25 rule and the Geno2Pheno algorithm with cut-off <10% FPR=CXCR4-using**.** | | | | | | | | | |

| PID T20 Week 4 | | | | | | | | | |
| --- | --- | --- | --- | --- | --- | --- | --- | --- | --- |
| **Clone** | **V3 sequence^a^** | **Confirmation assay**  **Trofile** | | | **Pre-screening**  **infectivity assessment** | | | **Genotypic prediction^b^** | |
|  |  | **Reported tropism** | **R5 RLU** | **X4 RLU** | **Result** | **R5 score** | **X4 score** | **11/25 rule** | **g2p 10% FPR** |
| 4 | CTRPNYHIRRRIHIGPGRAFYAGGVKGDIRQAYC | X4 | 132 | 1396 | X4 | 0 | 1 | Y (R/G) | CXCR4-USING |
| 6 | .I...NNT.TS..M...K.....SII......H. | R5 | 1128031 | 51 | R5 | 4 | 0 | N (S/S) | R5 |
| 10 | .................................. | DU | 13376 | 844163 | DU | 3 | 3 | Y (R/G) | CXCR4-USING |
| 11 | .................................. | DU | 2635 | 231959 | DU | 2 | 3 | Y (R/G) | CXCR4-USING |
| 22 | .....NNT.TS..M...K.....SII......H. | R5 | 575630 | 87 | R5 | 4 | 0 | N (S/S) | R5 |
| 23 | ......NV..............R..N........ | X4 | 73 | 25827 | X4 | 0 | 2 | Y (R/-) | CXCR4-USING |
| 26 | .....NNT.TS..M.........SII......H. | R5 | 1392630 | 91 | R5 | 4 | 0 | N (S/S) | R5 |
| 28 | .................................. | DU | 1545 | 369031 | DU | 2 | 3 | Y (R/G) | CXCR4-USING |
| 32 | .................................. | DU | 10394 | 770514 | DU | 3 | 4 | Y (R/G) | CXCR4-USING |
| 40 | .................................. | DU | 6387 | 673319 | DU | 3 | 3 | Y (R/G) | CXCR4-USING |
| 41 | .................................. | X4 | 76 | 95616 | X4 | 0 | 3 | Y (R/G) | CXCR4-USING |
| 47 | .................................. | DU | 736 | 80993 | DU | 2 | 3 | Y (R/G) | CXCR4-USING |
| Dots indicate residues identical to the major baseline sequence Envs; dashes indicate gaps.  ^a^gp120 V3 loop sequence (equivalent to position 296 to 331 in HXB2 NCBI accession number K03455).  ^b^Genotypic tropism was assessed using the 11/25 rule and the Geno2Pheno algorithm with cut-off <10% FPR=CXCR4-using**.** | | | | | | | | | |

| PID T69 Day 1 | | | | | | | | | |
| --- | --- | --- | --- | --- | --- | --- | --- | --- | --- |
| **Clone** | **V3 sequence^a^** | **Confirmation assay**  **Trofile** | | | **Pre-screening**  **infectivity assessment** | | | **Genotypic prediction^b^** | |
|  |  | **Reported tropism** | **R5 RLU** | **X4 RLU** | **Result** | **R5 score** | **X4 score** | **11/25 rule** | **g2p 10% FPR** |
| 2 | CTRPNNNTRKSINIGPGRAFYATGQIIGDIRQAHC | R5 | 584713 | 162 | R5 | 4 | 0 | N (S/Q) | R5 |
| 24 | ................................... | R5 | 119875 | 135 | R5 | 4 | 0 | N (S/Q) | R5 |
| 31 | ....KISKGRR.PV...TS..TM.VAK....K... | DU | 449 | 6898 | X4 | 0 | 2 | Y (R/V) | CXCR4-USING |
| 32 | ....KISKGRR.PV...TS..TM.VAK....K... | DU | 1996 | 123016 | DU | 2 | 3 | Y (R/V) | CXCR4-USING |
| 56 | ....KISKG.R.PV...TS..TM.VAK....K... | DU | 528 | 29250 | X4 | 0 | 2 | Y (R/V) | CXCR4-USING |
| 76 | ....KISKGRR.PV...TS..TM.VAK....K... | DU | 505 | 68834 | X4 | 0 | 3 | Y (R/V) | CXCR4-USING |
| 84 | ................................... | R5 | 629188 | 174 | R5 | 5 | 0 | N (S/Q) | R5 |
| 105 | ....KISKGRR.PV...TS..TM.VAK....K... | DU | 164 | 1868 | X4 | 0 | 2 | Y (R/V) | CXCR4-USING |
| 131 | ....KISKGRR.PV...TS..TM.VAR....K... | DU | 2615 | 45998 | DU | 2 | 3 | Y (R/V) | CXCR4-USING |
| 170 | ....KISKGRR.PV...TS..TM.VAK....K... | DU | 5998 | 67562 | DU | 3 | 3 | Y (R/V) | CXCR4-USING |
| 182 | ....QISKGRR.PV...TS..TM.VAK....K... | DU | 1328 | 70453 | DU | 2 | 3 | Y (R/V) | CXCR4-USING |
| 191 | ....KISKGRR.P....TS..TM.VAR....K... | DU | 1246 | 9841 | DU | 2 | 2 | Y (R/V) | CXCR4-USING |
| Dots indicate residues identical to the major baseline sequence Envs; dashes indicate gaps.  ^a^gp120 V3 loop sequence (equivalent to position 296 to 331 in HXB2 NCBI accession number K03455).  ^b^Genotypic tropism was assessed using the 11/25 rule and the Geno2Pheno algorithm with cut-off <10% FPR=CXCR4-using**.** | | | | | | | | | |

| PID T69 Week 8 | | | | | | | | | |
| --- | --- | --- | --- | --- | --- | --- | --- | --- | --- |
| **Clone** | **V3 sequence^a^** | **Confirmation assay**  **Trofile** | | | **Pre-screening**  **infectivity assessment** | | | **Genotypic prediction^b^** | |
|  |  | **Reported tropism** | **R5 RLU** | **X4 RLU** | **Result** | **R5 score** | **X4 score** | **11/25 rule** | **g2p 10% FPR** |
| 1 | CTRPKISKGRRIPVGPGTSFYTMGVAKGDIRKAHC | DU | 398 | 167311 | DU | 2 | 3 | Y (R/V) | CXCR4-USING |
| 4 | ....Q.............................. | DU | 709 | 154355 | DU | 2 | 3 | Y (R/V) | CXCR4-USING |
| 9 | ....Q.....................R........ | F | 74 | 647 | X4 | 0 | 1 | Y (R/V) | CXCR4-USING |
| 14 | ..........................R........ | X4 | 69 | 3290 | X4 | 0 | 2 | Y (R/V) | CXCR4-USING |
| 22 | ..........................R........ | X4 | 50 | 3065 | X4 | 0 | 2 | Y (R/V) | CXCR4-USING |
| 25 | ....Q.............................. | DU | 474 | 137130 | DU | 2 | 3 | Y (R/V) | CXCR4-USING |
| 32 | ................................... | DU | 396 | 95536 | DU | 2 | 3 | Y (R/V) | CXCR4-USING |
| 33 | ....Q.............................. | DU | 903 | 158076 | DU | 2 | 3 | Y (R/V) | CXCR4-USING |
| 40 | ................................... | X4 | 75 | 40473 | DU | 2 | 3 | Y (R/V) | CXCR4-USING |
| 41 | ..........................R........ | X4 | 53 | 4049 | X4 | 0 | 2 | Y (R/V) | CXCR4-USING |
| 43 | ....Q.............................. | DU | 257 | 39623 | DU | 1 | 2 | Y (R/V) | CXCR4-USING |
| 48 | ....Q.............................. | DU | 896 | 156279 | DU | 2 | 3 | Y (R/V) | CXCR4-USING |
| Dots indicate residues identical to the major baseline sequence Envs; dashes indicate gaps.  ^a^gp120 V3 loop sequence (equivalent to position 296 to 331 in HXB2 NCBI accession number K03455).  ^b^Genotypic tropism was assessed using the 11/25 rule and the Geno2Pheno algorithm with cut-off <10% FPR=CXCR4-using**.** | | | | | | | | | |

| PID T132 Day 1 | | | | | | | | | |
| --- | --- | --- | --- | --- | --- | --- | --- | --- | --- |
| **Clone** | **V3 sequence^a^** | **Confirmation assay**  **Trofile** | | | **Pre-screening**  **infectivity assessment** | | | **Genotypic prediction^b^** | |
|  |  | **Reported tropism** | **R5 RLU** | **X4 RLU** | **Result** | **R5 score** | **X4 score** | **11/25 rule** | **g2p 10% FPR** |
| 11 | CTRPNNFTEKRMTLGPGRVFYTTGKIVGDIRKAHC | X4 | 86 | 2397581 | X4 | 0 | 4 | Y (R/K) | CXCR4-USING |
| 31 | ................................... | DU | 273 | 3466022 | DU | 1 | 4 | Y (R/K) | CXCR4-USING |
| 61 | ................................... | DU | 371 | 981268 | X4 | 0 | 3 | Y (R/K) | CXCR4-USING |
| 95 | ......N.R.SINI....A..A..D.I....Q... | R5 | 7372 | 70 | R5 | 2 | 0 | N (S/D) | R5 |
| 101 | ................................... | DU | 301 | 2238663 | DU | 1 | 4 | Y (R/K) | CXCR4-USING |
| 110 | ................................... | DU | 423 | 2330347 | DU | 2 | 4 | Y (R/K) | CXCR4-USING |
| 116 | ......N.R.SINI....A..A..D.I....Q... | R5 | 3176401 | 92 | R5 | 5 | 0 | N (S/D) | R5 |
| 133 | ......N.R.SINI....A..A..D.I....Q... | R5 | 2992780 | 114 | DU | 5 | 1 | N (S/D) | R5 |
| 147 | ......N.R.SIN.....A..A..D.I....Q... | R5 | 1033820 | 76 | R5 | 4 | 0 | N (S/D) | R5 |
| 159 | ................................... | R5 | 1181 | 63 | R5 | 1 | 0 | Y (R/K) | CXCR4-USING |
| 177 | ................................... | DU | 2784 | 1332929 | DU | 2 | 4 | Y (R/K) | CXCR4-USING |
| 192 | ......N.R.SIN.....A..A..D.I....Q... | R5 | 3241330 | 139 | R5 | 5 | 0 | N (S/D) | R5 |
| Dots indicate residues identical to the major baseline sequence Envs; dashes indicate gaps.  ^a^gp120 V3 loop sequence (equivalent to position 296 to 331 in HXB2 NCBI accession number K03455).  ^b^Genotypic tropism was assessed using the 11/25 rule and the Geno2Pheno algorithm with cut-off <10% FPR=CXCR4-using**.** | | | | | | | | | |

| PID T132 Week 8 | | | | | | | | | |
| --- | --- | --- | --- | --- | --- | --- | --- | --- | --- |
| **Clone** | **V3 sequence^a^** | **Confirmation assay**  **Trofile** | | | **Pre-screening**  **infectivity assessment** | | | **Genotypic prediction^b^** | |
|  |  | **Reported tropism** | **R5 RLU** | **X4 RLU** | **Result** | **R5 score** | **X4 score** | **11/25 rule** | **g2p 10% FPR** |
| 3 | CTRPNNFTEKRMTLGPGRVFYTTGKIVGDIRKAHC | X4 | 55 | 518137 | X4 | 0 | 4 | Y (R/K) | CXCR4-USING |
| 5 | ......N.R.SINI....A..A..D.I....Q... | R5 | 48438 | 73 | R5 | 3 | 0 | N (S/D) | R5 |
| 9 | ................................... | X4 | 61 | 1551222 | X4 | 0 | 4 | Y (R/K) | CXCR4-USING |
| 15 | ......N.R.SIN.....A..A..D.I....Q... | R5 | 1100811 | 60 | R5 | 5 | 0 | N (S/D) | R5 |
| 20 | ......N.R.SIN.....A..A..D.I....Q... | R5 | 1845028 | 128 | R5 | 4 | 0 | N (S/D) | R5 |
| 26 | ......N.R.SINI....A..A..D.I....Q... | R5 | 866246 | 96 | R5 | 5 | 0 | N (S/D) | R5 |
| 31 | ......N.R.SINI....A..A..D.I....Q... | R5 | 1441709 | 59 | R5 | 5 | 0 | N (S/D) | R5 |
| 33 | ......N.R.SINI....A..A..D.I....Q... | R5 | 648442 | 59 | R5 | 4 | 0 | N (S/D) | R5 |
| 37 | ......N.R.SIN.E...A..A..D.I....Q... | R5 | 4598 | 72 | R5 | 2 | 0 | N (S/D) | R5 |
| 39 | ......N.R.SINI....A..A..D.I....Q... | R5 | 4259 | 86 | R5 | 2 | 0 | N (S/D) | R5 |
| 42 | ......N.R.SINI....A..A..D.I....Q... | R5 | 411059 | 95 | R5 | 4 | 0 | N (S/D) | R5 |
| 46 | ......N.R.SINI....A..A..D.I....Q... | R5 | 190770 | 84 | R5 | 4 | 0 | N (S/D) | R5 |
| Dots indicate residues identical to the major baseline sequence Envs; dashes indicate gaps.  ^a^gp120 V3 loop sequence (equivalent to position 296 to 331 in HXB2 NCBI accession number K03455).  ^b^Genotypic tropism was assessed using the 11/25 rule and the Geno2Pheno algorithm with cut-off <10% FPR=CXCR4-using**.** | | | | | | | | | |

| PID T205 Day 1 | | | | | | | | | |
| --- | --- | --- | --- | --- | --- | --- | --- | --- | --- |
| **Clone** | **V3 sequence^a^** | **Confirmation assay**  **Trofile** | | | **Pre-screening**  **infectivity assessment** | | | **Genotypic prediction^b^** | |
|  |  | **Reported tropism** | **R5 RLU** | **X4 RLU** | **Result** | **R5 score** | **X4 score** | **11/25 rule** | **g2p 10% FPR** |
| 13 | CTRPNNNTRKSISIGPGRAFYTTGDIIGDIRQAHC | R5 | 587144 | 89 | R5 | 4 | 0 | N (S/D) | R5 |
| 47 | .........R......................T.. | R5 | 240666 | 95 | R5 | 4 | 0 | N (S/D) | R5 |
| 61 | .......K..RVTL....V.....E.V........ | DU | 288 | 4851 | DU | 1 | 2 | Y (R/E) | CXCR4-USING |
| 64 | .....T.K..RVTL....V.....E.T........ | DU | 583 | 2706996 | DU | 1 | 4 | Y (R/E) | CXCR4-USING |
| 77 | .........R......................... | R5 | 49777 | 93 | R5 | 3 | 0 | N (S/D) | R5 |
| 104 | .......K..RVTL....V.....E.T........ | X4 | 72 | 344946 | X4 | 0 | 3 | Y (R/E) | CXCR4-USING |
| 111 | .........R......................... | R5 | 1388361 | 89 | R5 | 5 | 0 | N (S/D) | R5 |
| 130 | .......K..RVTL....V.....E.T........ | X4 | 94 | 230556 | X4 | 0 | 3 | Y (R/E) | CXCR4-USING |
| 148 | .........R..............G.......... | R5 | 1363661 | 153 | R5 | 4 | 0 | N (S/G) | R5 |
| 156 | .........RN........................ | R5 | 38951 | 90 | R5 | 3 | 0 | N (N/D) | R5 |
| 176 | .......K..RVTL....V.....E.T........ | X4 | 146 | 307980 | X4 | 0 | 4 | Y (R/E) | CXCR4-USING |
| 182 | .......K..RVTL....V.....E.T........ | X4 | 191 | 344145 | X4 | 0 | 3 | Y (R/E) | CXCR4-USING |
| Dots indicate residues identical to the major baseline sequence Envs; dashes indicate gaps.  ^a^gp120 V3 loop sequence (equivalent to position 296 to 331 in HXB2 NCBI accession number K03455).  ^b^Genotypic tropism was assessed using the 11/25 rule and the Geno2Pheno algorithm with cut-off <10% FPR=CXCR4-using**.** | | | | | | | | | |

| PID T205 Week 8 | | | | | | | | | |
| --- | --- | --- | --- | --- | --- | --- | --- | --- | --- |
| **Clone** | **V3 sequence^a^** | **Confirmation assay**  **Trofile** | | | **Pre-screening**  **infectivity assessment** | | | **Genotypic prediction^b^** | |
|  |  | **Reported tropism** | **R5 RLU** | **X4 RLU** | **Result** | **R5 score** | **X4 score** | **11/25 rule** | **g2p 10% FPR** |
| 14 | CTRPNTNKRKRVTLGPGRVFYTTGEIMGDIRQAHC | DU | 1898 | 2457592 | DU | 2 | 4 | Y (R/E) | CXCR4-USING |
| 15 | .....NK.TQGIHT....AYF.RTI.......... | F | 303 | 297 | DU | 2 | 1 | N (G/T) | CXCR4-USING |
| 19 | .....N............................. | X4 | 52 | 14196 | X4 | 0 | 2 | Y (R/E) | CXCR4-USING |
| 21 | ..........................T........ | X4 | 122 | 443311 | X4 | 0 | 4 | Y (R/E) | CXCR4-USING |
| 24 | .....N....................T........ | X4 | 101 | 6774 | DU | 1 | 2 | Y (R/E) | CXCR4-USING |
| 30 | .....N........................G.... | X4 | 138 | 425890 | X4 | 0 | 4 | Y (R/E) | CXCR4-USING |
| 31 | ..........................T........ | X4 | 83 | 1255575 | DU | 1 | 4 | Y (R/E) | CXCR4-USING |
| 36 | .....N...........K........T........ | X4 | 62 | 80896 | X4 | 0 | 3 | Y (R/E) | CXCR4-USING |
| 37 | ....TN............................. | X4 | 52 | 162300 | DU | 1 | 3 | Y (R/E) | CXCR4-USING |
| 38 | .....N....................T........ | X4 | 61 | 59258 | DU | 1 | 3 | Y (R/E) | CXCR4-USING |
| 44 | .....N............................. | X4 | 104 | 80660 | X4 | 0 | 3 | Y (R/E) | CXCR4-USING |
| 46 | .....S....................T........ | X4 | 120 | 962770 | X4 | 0 | 4 | Y (R/E) | CXCR4-USING |
| Dots indicate residues identical to the major baseline sequence Envs; dashes indicate gaps.  ^a^gp120 V3 loop sequence (equivalent to position 296 to 331 in HXB2 NCBI accession number K03455).  ^b^Genotypic tropism was assessed using the 11/25 rule and the Geno2Pheno algorithm with cut-off <10% FPR=CXCR4-using**.** | | | | | | | | | |

| PID T221 Day 1 | | | | | | | | | |
| --- | --- | --- | --- | --- | --- | --- | --- | --- | --- |
| **Clone** | **V3 sequence^a^** | **Confirmation assay**  **Trofile** | | | **Pre-screening**  **infectivity assessment** | | | **Genotypic prediction^b^** | |
|  |  | **Reported tropism** | **R5 RLU** | **X4 RLU** | **Result** | **R5 score** | **X4 score** | **11/25 rule** | **g2p 10% FPR** |
| 4 | CTRPNNNTGKGIHMGPGRAYFTGDIIGDIRQAHC | F | 236 | 73 | R5 | 2 | 0 | N (G/D) | R5 |
| 58 | ........R................V........ | R5 | 7840 | 55 | R5 | 3 | 0 | N (G/D) | R5 |
| 60 | ........R......................... | R5 | 105987 | 88 | R5 | 4 | 0 | N (G/D) | R5 |
| 112 | ........R......................... | R5 | 58697 | 70 | R5 | 4 | 0 | N (G/D) | R5 |
| 127 | ........R......................... | R5 | 76023 | 82 | R5 | 4 | 0 | N (G/D) | R5 |
| 139 | ........R......................... | R5 | 92996 | 51 | R5 | 3 | 0 | N (G/D) | R5 |
| 142 | ........R...................V..... | F | 852 | 90 | R5 | 2 | 0 | N (G/D) | R5 |
| 152 | ........R...................M..... | R5 | 15862 | 98 | R5 | 3 | 0 | N (G/D) | R5 |
| 158 | ........R......................... | R5 | 66475 | 53 | R5 | 4 | 0 | N (G/D) | R5 |
| 165 | ........R......................... | R5 | 70432 | 84 | R5 | 4 | 0 | N (G/D) | R5 |
| 176 | ......KKTQ...T........RT.......... | DU | 93828 | 76921 | DU | 3 | 3 | N (G/T) | CXCR4-USING |
| 181 | ........R......................... | R5 | 67652 | 87 | R5 | 3 | 0 | N (G/D) | R5 |
| Dots indicate residues identical to the major baseline sequence Envs; dashes indicate gaps.  ^a^gp120 V3 loop sequence (equivalent to position 296 to 331 in HXB2 NCBI accession number K03455).  ^b^Genotypic tropism was assessed using the 11/25 rule and the Geno2Pheno algorithm with cut-off <10% FPR=CXCR4-using**.** | | | | | | | | | |

| PID T221 Week 4 | | | | | | | | | |
| --- | --- | --- | --- | --- | --- | --- | --- | --- | --- |
| **Clone** | **V3 sequence^a^** | **Confirmation assay**  **Trofile** | | | **Pre-screening**  **infectivity assessment** | | | **Genotypic prediction^b^** | |
|  |  | **Reported tropism** | **R5 RLU** | **X4 RLU** | **Result** | **R5 score** | **X4 score** | **11/25 rule** | **g2p 10% FPR** |
| 2 | CTRPNNKKTQGMHTGPGRAYFTRTIIGDIRQAHC | DU | 536 | 3491 | DU | 1 | 2 | N (G/T) | CXCR4-USING |
| 3 | ...........I...................... | DU | 109496 | 78501 | DU | 4 | 3 | N (G/T) | CXCR4-USING |
| 14 | .....S.....I...................... | DU | 525 | 1305 | DU | 2 | 1 | N (G/T) | CXCR4-USING |
| 16 | ...........I...................... | DU | 103956 | 105579 | DU | 3 | 3 | N (G/T) | CXCR4-USING |
| 18 | ...........I...................... | DU | 94569 | 71384 | DU | 3 | 3 | N (G/T) | CXCR4-USING |
| 21 | .....K.....I...................... | DU | 27099 | 30540 | DU | 3 | 3 | N (G/T) | CXCR4-USING |
| 24 | .........R.I...................... | F | 184 | 105 | R5 | 1 | 0 | N (G/T) | CXCR4-USING |
| 25 | ...........I...................... | DU | 13217 | 10973 | DU | 3 | 2 | N (G/T) | CXCR4-USING |
| 32 | ...........I...................... | DU | 135257 | 129294 | DU | 4 | 3 | N (G/T) | CXCR4-USING |
| 38 | ...........I...................... | DU | 53755 | 54188 | DU | 3 | 2 | N (G/T) | CXCR4-USING |
| 47 | ...........I...................... | DU | 145522 | 142403 | DU | 4 | 3 | N (G/T) | CXCR4-USING |
| 48 | .....K.....I...................... | DU | 33613 | 39923 | DU | 3 | 2 | N (G/T) | CXCR4-USING |
| Dots indicate residues identical to the major baseline sequence Envs; dashes indicate gaps.  ^a^gp120 V3 loop sequence (equivalent to position 296 to 331 in HXB2 NCBI accession number K03455).  ^b^Genotypic tropism was assessed using the 11/25 rule and the Geno2Pheno algorithm with cut-off <10% FPR=CXCR4-using**.** | | | | | | | | | |

| PID T246 Day 1 | | | | | | | | | |
| --- | --- | --- | --- | --- | --- | --- | --- | --- | --- |
| **Clone** | **V3 sequence^a^** | **Confirmation assay**  **Trofile** | | | **Pre-screening**  **infectivity assessment** | | | **Genotypic prediction^b^** | |
|  |  | **Reported tropism** | **R5 RLU** | **X4 RLU** | **Result** | **R5 score** | **X4 score** | **11/25 rule** | **g2p 10% FPR** |
| 6 | CTRPNNNTRKGIHIGPGRAVYTTGRIIGDIRKAYC | F | 714 | 92 | R5 | 1 | 0 | Y (G/R) | CXCR4-USING |
| 24 | ....Y.Y.KR..............K...N...... | DU | 246064 | 124485 | DU | 4 | 3 | Y (G/K) | CXCR4-USING |
| 28 | .........R..............K.......... | DU | 958893 | 1287843 | DU | 4 | 4 | Y (G/K) | CXCR4-USING |
| 34 | ........................K.......... | DU | 1201848 | 1550983 | DU | 4 | 4 | Y (G/K) | CXCR4-USING |
| 50 | .........R..............K.......... | DU | 83461 | 52729 | DU | 3 | 2 | Y (G/K) | CXCR4-USING |
| 51 | ...................F.A..D......R... | R5 | 709650 | 56 | R5 | 4 | 0 | N (G/D) | CXCR4-USING |
| 100 | .........R..............K...N...... | DU | 1775 | 380 | R5 | 2 | 0 | Y (G/K) | CXCR4-USING |
| 119 | ...................F.A..E......R... | DU | 30718 | 1432 | DU | 3 | 1 | N (G/E) | CXCR4-USING |
| 128 | ...................F.A..D......R... | DU | 1800606 | 83277 | DU | 5 | 3 | N (G/D) | CXCR4-USING |
| 135 | ...................F.A..D......R... | R5 | 1612185 | 146 | R5 | 5 | 0 | N (G/D) | CXCR4-USING |
| 151 | .........R..............K.......... | R5 | 1310 | 81 | R5 | 1 | 0 | Y (G/K) | CXCR4-USING |
| 183 | .........R..............K.......... | DU | 1076 | 594 | DU | 1 | 1 | Y (G/K) | CXCR4-USING |
| Dots indicate residues identical to the major baseline sequence Envs; dashes indicate gaps.  ^a^gp120 V3 loop sequence (equivalent to position 296 to 331 in HXB2 NCBI accession number K03455).  ^b^Genotypic tropism was assessed using the 11/25 rule and the Geno2Pheno algorithm with cut-off <10% FPR=CXCR4-using**.** | | | | | | | | | |

| PID T246 Week 4 | | | | | | | | | |
| --- | --- | --- | --- | --- | --- | --- | --- | --- | --- |
| **Clone** | **V3 sequence^a^** | **Confirmation assay**  **Trofile** | | | **Pre-screening**  **infectivity assessment** | | | **Genotypic prediction^b^** | |
|  |  | **Reported tropism** | **R5 RLU** | **X4 RLU** | **Result** | **R5 score** | **X4 score** | **11/25 rule** | **g2p 10% FPR** |
| 2 | CTRPNNNTRRGIHIGPGRAVYTTGKIIGDIRKAYC | DU | 110198 | 65307 | DU | 3 | 2 | Y (G/K) | CXCR4-USING |
| 5 | ................................... | DU | 1193196 | 1234747 | DU | 4 | 4 | Y (G/K) | CXCR4-USING |
| 8 | .........K.........F.A..D......R... | R5 | 2860 | 77 | R5 | 1 | 0 | N (G/D) | CXCR4-USING |
| 9 | .........K.........F.A..D......R... | R5 | 569546 | 88 | R5 | 4 | 0 | N (G/D) | CXCR4-USING |
| 10 | .........K......................... | DU | 841670 | 864804 | DU | 4 | 4 | Y (G/K) | CXCR4-USING |
| 15 | ................................... | F | 529 | 244 | R5 | 1 | 0 | Y (G/K) | CXCR4-USING |
| 20 | .........K.........F.A..D......R... | DU | 57452 | 1525 | DU | 2 | 1 | N (G/D) | CXCR4-USING |
| 30 | .....................A............. | DU | 195889 | 140649 | DU | 3 | 3 | Y (G/K) | CXCR4-USING |
| 32 | ................................... | F | 76 | 95 | R5 | 1 | 0 | Y (G/K) | CXCR4-USING |
| 39 | .........K.........F.A............. | DU | 529256 | 114796 | DU | 4 | 3 | Y (G/K) | CXCR4-USING |
| 42 | .........K.........F.A..D......R... | DU | 63860 | 1458 | R5 | 3 | 0 | N (G/D) | CXCR4-USING |
| 43 | ................................... | DU | 303602 | 252004 | DU | 4 | 3 | Y (G/K) | CXCR4-USING |
| Dots indicate residues identical to the major baseline sequence Envs; dashes indicate gaps.  ^a^gp120 V3 loop sequence (equivalent to position 296 to 331 in HXB2 NCBI accession number K03455).  ^b^Genotypic tropism was assessed using the 11/25 rule and the Geno2Pheno algorithm with cut-off <10% FPR=CXCR4-using**.** | | | | | | | | | |

| PID T251 Day 1 SCREENING ESTA R5 | | | | | | | | | |
| --- | --- | --- | --- | --- | --- | --- | --- | --- | --- |
| **Clone** | **V3 sequence^a^** | **Confirmation assay**  **Trofile** | | | **Pre-screening**  **infectivity assessment** | | | **Genotypic prediction^b^** | |
|  |  | **Reported tropism** | **R5 RLU** | **X4 RLU** | **Result** | **R5 score** | **X4 score** | **11/25 rule** | **g2p 10% FPR** |
| 2 | CTRPSNNTRKGIHIGPGRAFYATDIIGDIRQAHC | R5 | 377161 | 83 | R5 | 5 | 0 | N (G/D) | R5 |
| 22 | ....N............................. | R5 | 74509 | 68 | R5 | 4 | 0 | N (G/D) | R5 |
| 32 | .................................. | F | 998 | 74 | R5 | 2 | 0 | N (G/D) | R5 |
| 57 | .................................. | R5 | 314787 | 69 | R5 | 4 | 0 | N (G/D) | R5 |
| 61 | ..............................R... | R5 | 17837 | 78 | R5 | 4 | 0 | N (G/D) | CXCR4-USING |
| 65 | .................................. | R5 | 4367 | 88 | R5 | 3 | 0 | N (G/D) | R5 |
| 92 | ............................M..... | R5 | 4787 | 69 | R5 | 2 | 0 | N (G/D) | R5 |
| 108 | .............................G.... | F | 243 | 87 | R5 | 1 | 0 | N (G/D) | R5 |
| 112 | ......D........................... | F | 206 | 78 | R5 | 1 | 0 | N (G/D) | R5 |
| 117 | ..S............................... | F | 67 | 91 | R5 | 2 | 0 | N (G/D) | R5 |
| 178 | ................R................. | R5 | 7790 | 56 | R5 | 3 | 0 | N (G/D) | R5 |
| 184 | ................................Y. | R5 | 68303 | 50 | R5 | 4 | 0 | N (G/D) | R5 |
| Dots indicate residues identical to the major baseline sequence Envs; dashes indicate gaps.  ^a^gp120 V3 loop sequence (equivalent to position 296 to 331 in HXB2 NCBI accession number K03455).  ^b^Genotypic tropism was assessed using the 11/25 rule and the Geno2Pheno algorithm with cut-off <10% FPR=CXCR4-using**.** | | | | | | | | | |

| PID T251 Week 4 | | | | | | | | | |
| --- | --- | --- | --- | --- | --- | --- | --- | --- | --- |
| **Clone** | **V3 sequence^a^** | **Confirmation assay**  **Trofile** | | | **Pre-screening**  **infectivity assessment** | | | **Genotypic prediction^b^** | |
|  |  | **Reported tropism** | **R5 RLU** | **X4 RLU** | **Result** | **R5 score** | **X4 score** | **11/25 rule** | **g2p 10% FPR** |
| 1 | CTRPSQKQIRRIHIGPGRAFYATDIKGNIKQAHC | X4 | 75 | 103997 | X4 | 0 | 4 | Y (R/D) | CXCR4-USING |
| 6 | .................................. | X4 | 50 | 122030 | X4 | 0 | 4 | Y (R/D) | CXCR4-USING |
| 8 | .................................. | X4 | 62 | 7950 | X4 | 0 | 2 | Y (R/D) | CXCR4-USING |
| 10 | .................................. | X4 | 57 | 133573 | DU | 1 | 4 | Y (R/D) | CXCR4-USING |
| 19 | .................................. | X4 | 52 | 149311 | DU | 1 | 3 | Y (R/D) | CXCR4-USING |
| 24 | .................................. | X4 | 50 | 605073 | X4 | 0 | 3 | Y (R/D) | CXCR4-USING |
| 25 | .................................. | X4 | 57 | 18229 | X4 | 0 | 2 | Y (R/D) | CXCR4-USING |
| 29 | .................................. | X4 | 90 | 257331 | X4 | 0 | 3 | Y (R/D) | CXCR4-USING |
| 36 | .................................. | X4 | 57 | 4264 | X4 | 0 | 2 | Y (R/D) | CXCR4-USING |
| 38 | ...LNNNTRKSMTL.................... | X4 | 58 | 290329 | X4 | 0 | 4 | N (S/D) | R5 |
| 44 | ...LNNNTRKSMTL.................... | X4 | 51 | 39631 | X4 | 0 | 3 | N (S/D) | R5 |
| 47 | ...LNNNTRKSMTL.................... | F | 61 | 308 | X4 | 0 | 1 | N (S/D) | R5 |
| Dots indicate residues identical to the major baseline sequence Envs; dashes indicate gaps.  ^a^gp120 V3 loop sequence (equivalent to position 296 to 331 in HXB2 NCBI accession number K03455).  ^b^Genotypic tropism was assessed using the 11/25 rule and the Geno2Pheno algorithm with cut-off <10% FPR=CXCR4-using**.** | | | | | | | | | |

| PID T285 Day 1 | | | | | | | | | |
| --- | --- | --- | --- | --- | --- | --- | --- | --- | --- |
| **Clone** | **V3 sequence^a^** | **Confirmation assay**  **Trofile** | | | **Pre-screening**  **infectivity assessment** | | | **Genotypic prediction^b^** | |
|  |  | **Reported tropism** | **R5 RLU** | **X4 RLU** | **Result** | **R5 score** | **X4 score** | **11/25 rule** | **g2p 10% FPR** |
| 4 | CTRLNNNTRRSITIGPGRAFYTSDIIGNIRQAHC | R5 | 256580 | 100 | R5 | 4 | 0 | N (S/D) | R5 |
| 21 | .....................A.....D...... | F | 147 | 82 | X4 | 0 | 1 | N (S/D) | R5 |
| 40 | ..............................R... | R5 | 74786 | 64 | R5 | 3 | 0 | N (S/D) | R5 |
| 44 | .................................. | R5 | 219468 | 74 | DU | 4 | 1 | N (S/D) | R5 |
| 54 | .........K.M.L...KV...TGT......... | DU | 1407128 | 1339500 | DU | 4 | 4 | N (S/-) | CXCR4-USING |
| 93 | .....................A.....D...... | R5 | 337842 | 101 | R5 | 4 | 0 | N (S/D) | R5 |
| 102 | ......S................N.......... | DU | 478245 | 847 | R5 | 4 | 0 | N (S/N) | R5 |
| 105 | .................................. | DU | 2495776 | 639 | DU | 4 | 1 | N (S/D) | R5 |
| 112 | .................................. | R5 | 360075 | 75 | DU | 4 | 1 | N (S/D) | R5 |
| 126 | ...........................D...... | R5 | 1509006 | 112 | R5 | 5 | 0 | N (S/D) | R5 |
| 136 | .........K.M.L...KV...TGT......... | DU | 7050 | 28615 | DU | 2 | 2 | N (S/-) | CXCR4-USING |
| 171 | .........K.M.L...KV...TGT......... | DU | 6958 | 5028 | DU | 2 | 1 | N (S/-) | CXCR4-USING |
| Dots indicate residues identical to the major baseline sequence Envs; dashes indicate gaps.  ^a^gp120 V3 loop sequence (equivalent to position 296 to 331 in HXB2 NCBI accession number K03455).  ^b^Genotypic tropism was assessed using the 11/25 rule and the Geno2Pheno algorithm with cut-off <10% FPR=CXCR4-using**.** | | | | | | | | | |

| PID T285 Week 4 | | | | | | | | | |
| --- | --- | --- | --- | --- | --- | --- | --- | --- | --- |
| **Clone** | **V3 sequence^a^** | **Confirmation assay**  **Trofile** | | | **Pre-screening**  **infectivity assessment** | | | **Genotypic prediction^b^** | |
|  |  | **Reported tropism** | **R5 RLU** | **X4 RLU** | **Result** | **R5 score** | **X4 score** | **11/25 rule** | **g2p 10% FPR** |
| 1 | CTRLNNNTRKSMTLGPGKVFYTTGTIGNIRQAHC | DU | 255943 | 179545 | DU | 4 | 3 | N (S/-) | CXCR4-USING |
| 12 | .................................. | DU | 328029 | 246792 | DU | 4 | 3 | N (S/-) | CXCR4-USING |
| 15 | .................................. | DU | 1071092 | 986122 | DU | 4 | 4 | N (S/-) | CXCR4-USING |
| 16 | R................................. | DU | 12683 | 8424 | DU | 2 | 1 | N (S/-) | CXCR4-USING |
| 20 | .................................. | DU | 618338 | 450193 | DU | 4 | 4 | N (S/-) | CXCR4-USING |
| 21 | .................................. | DU | 97968 | 62969 | DU | 3 | 3 | N (S/-) | CXCR4-USING |
| 23 | .................................. | DU | 953043 | 878728 | DU | 4 | 4 | N (S/-) | CXCR4-USING |
| 29 | .................................. | F | 302 | 144 | X4 | 0 | 1 | N (S/-) | CXCR4-USING |
| 30 | .......................D.......... | DU | 246073 | 134431 | DU | 3 | 2 | N (S/D) | CXCR4-USING |
| 38 | .................................. | DU | 217008 | 136996 | DU | 3 | 2 | N (S/-) | CXCR4-USING |
| 39 | .................................. | DU | 3626046 | 3650328 | DU | 5 | 4 | N (S/-) | CXCR4-USING |
| 46 | .................................. | DU | 1478339 | 1552305 | DU | 4 | 3 | N (S/-) | CXCR4-USING |
| Dots indicate residues identical to the major baseline sequence Envs; dashes indicate gaps.  ^a^gp120 V3 loop sequence (equivalent to position 296 to 331 in HXB2 NCBI accession number K03455).  ^b^Genotypic tropism was assessed using the 11/25 rule and the Geno2Pheno algorithm with cut-off <10% FPR=CXCR4-using**.** | | | | | | | | | |

| PID T347 Day 1 | | | | | | | | | |
| --- | --- | --- | --- | --- | --- | --- | --- | --- | --- |
| **Clone** | **V3 sequence^a^** | **Confirmation assay**  **Trofile** | | | **Pre-screening**  **infectivity assessment** | | | **Genotypic prediction^b^** | |
|  |  | **Reported tropism** | **R5 RLU** | **X4 RLU** | **Result** | **R5 score** | **X4 score** | **11/25 rule** | **g2p 10% FPR** |
| 14 | CIRPNNNTRKRVTMGPGRVWYTTGEIIGDIKKAYC | DU | 3516 | 4475 | DU | 2 | 2 | Y (R/E) | CXCR4-USING |
| 16 | .T.......RSIPI....AF.A..D.....RQ.H. | R5 | 2984 | 83 | R5 | 2 | 0 | N (S/D) | R5 |
| 32 | .T.......RSIPI....AF.A..D.....RQ.H. | R5 | 162837 | 65 | R5 | 4 | 0 | N (S/D) | R5 |
| 38 | .T.......RSIPI....AF....D.....RQ.H. | F | 73 | 77 | R5 | 2 | 0 | N (S/D) | R5 |
| 45 | .A..G....RSISI....AFFA..D.....RQ.H. | R5 | 553023 | 75 | R5 | 4 | 0 | N (S/D) | R5 |
| 94 | .T.......RSIPI....AF.A..D.....RQ.H. | R5 | 2992422 | 82 | R5 | 5 | 0 | N (S/D) | R5 |
| 101 | .T.......RSIPI....AF.A..D.....RQ.H. | R5 | 642412 | 82 | R5 | 5 | 0 | N (S/D) | R5 |
| 148 | .T........SIPI....AF.A..D.....RQ.H. | R5 | 13975 | 87 | R5 | 3 | 0 | N (S/D) | R5 |
| 156 | .T.......RSIPI....AF....D.....RQ.H. | R5 | 1763379 | 175 | R5 | 5 | 0 | N (S/D) | R5 |
| 171 | ................................... | DU | 33131 | 80563 | DU | 3 | 3 | Y (R/E) | CXCR4-USING |
| 177 | .T.......RSIPI....AF.A..D.....RQ.H. | R5 | 1752026 | 190 | DU | 5 | 1 | N (S/D) | R5 |
| 178 | .T........GINI....A..A......N.RQ.H. | R5 | 604833 | 61 | R5 | 4 | 0 | N (G/E) | R5 |
| Dots indicate residues identical to the major baseline sequence Envs; dashes indicate gaps.  ^a^gp120 V3 loop sequence (equivalent to position 296 to 331 in HXB2 NCBI accession number K03455).  ^b^Genotypic tropism was assessed using the 11/25 rule and the Geno2Pheno algorithm with cut-off <10% FPR=CXCR4-using**.** | | | | | | | | | |

| PID T347 Week 8 | | | | | | | | | |
| --- | --- | --- | --- | --- | --- | --- | --- | --- | --- |
| **Clone** | **V3 sequence^a^** | **Confirmation assay** | | | **Pre-screening**  **infectivity assessment** | | | **Genotypic prediction^b^** | |
|  |  | **Reported tropism** | **R5 RLU** | **X4 RLU** | **Result** | **R5 score** | **X4 score** | **11/25 rule** | **g2p 10% FPR** |
| 1 | CIRPNNNTRRRVTMGPGRVWYTTGKIIGDIKKAYC | R5 | 7019 | 75 | R5 | 2 | 0 | Y (R/K) | CXCR4-USING |
| 2 | ................................... | X4 | 54 | 2819 | DU | 1 | 2 | Y (R/K) | CXCR4-USING |
| 9 | ................................... | X4 | 81 | 7466 | X4 | 0 | 2 | Y (R/K) | CXCR4-USING |
| 12 | ................................... | DU | 264 | 189032 | DU | 2 | 3 | Y (R/K) | CXCR4-USING |
| 13 | .T........SIPI....AF.A..D.....RQ.H. | R5 | 9332 | 74 | R5 | 2 | 0 | N (S/D) | R5 |
| 15 | .T........SIPI....AF.A..D.....RQ.H. | R5 | 652507 | 92 | R5 | 4 | 0 | N (S/D) | R5 |
| 16 | ................................... | DU | 301 | 419320 | DU | 2 | 4 | Y (R/K) | CXCR4-USING |
| 21 | ................................... | DU | 224 | 163631 | DU | 1 | 3 | Y (R/K) | CXCR4-USING |
| 33 | .T........SIPI....AF.A..D.....RQ.H. | R5 | 1768893 | 98 | R5 | 5 | 0 | N (S/D) | R5 |
| 34 | ................................... | DU | 264 | 160859 | DU | 1 | 3 | Y (R/K) | CXCR4-USING |
| 44 | .T........SIPI....AF.A..D.....RQ.H. | R5 | 24768 | 64 | R5 | 3 | 0 | N (S/D) | R5 |
| 45 | ................................... | DU | 630 | 163112 | DU | 2 | 3 | Y (R/K) | CXCR4-USING |
| Dots indicate residues identical to the major baseline sequence Envs; dashes indicate gaps.  ^a^gp120 V3 loop sequence (equivalent to position 296 to 331 in HXB2 NCBI accession number K03455).  ^b^Genotypic tropism was assessed using the 11/25 rule and the Geno2Pheno algorithm with cut-off <10% FPR=CXCR4-using**.** | | | | | | | | | |

| PID T377 Day 1 | | | | | | | | | |
| --- | --- | --- | --- | --- | --- | --- | --- | --- | --- |
| **Clone** | **V3 sequence^a^** | **Confirmation assay**  **Trofile** | | | **Pre-screening**  **infectivity assessment** | | | **Genotypic prediction^b^** | |
|  |  | **Reported tropism** | **R5 RLU** | **X4 RLU** | **Result** | **R5 score** | **X4 score** | **11/25 rule** | **g2p 10% FPR** |
| 30 | CTRLSSYTKRRIQIGPGRSFYTAREVQGDLRQAHC | X4 | 68 | 882217 | X4 | 0 | 4 | Y (R/E) | CXCR4-USING |
| 41 | ...PNNN.RKS.N.....A..A.DQII..I..... | R5 | 6644 | 65 | R5 | 3 | 0 | N (S/Q) | R5 |
| 58 | ....................H..KQ.K........ | DU | 703 | 1481897 | DU | 2 | 4 | Y (R/Q) | CXCR4-USING |
| 69 | ....................H..KQ.K........ | X4 | 102 | 1055459 | X4 | 0 | 4 | Y (R/Q) | CXCR4-USING |
| 72 | .V.PNNN.RKS.N.....A..A.DQII..I..... | R5 | 6168 | 69 | DU | 3 | 1 | N (S/Q) | R5 |
| 73 | ...PNNN.RKS.N.....A..A.DQII..I..... | R5 | 3200 | 104 | R5 | 2 | 0 | N (S/Q) | R5 |
| 74 | .I.PNNN.RKS.N.....A..A.DQII..I..... | R5 | 10065 | 71 | R5 | 4 | 0 | N (S/Q) | R5 |
| 111 | .I.PNNN.RKS.N.....A..A.DQII..I..... | R5 | 377035 | 58 | R5 | 5 | 0 | N (S/Q) | R5 |
| 140 | .I.PNNN.RKS.N.....A..A.DQII..I..... | R5 | 735176 | 89 | DU | 5 | 1 | N (S/Q) | R5 |
| 167 | .......................K........... | X4 | 82 | 768476 | X4 | 0 | 4 | Y (R/E) | CXCR4-USING |
| 181 | .V.PNNN.RKS.D.....A..A.DQII..I..... | R5 | 205756 | 89 | R5 | 4 | 0 | N (S/Q) | R5 |
| 182 | ....................H..KQ.K........ | DU | 333 | 1057303 | DU | 1 | 4 | Y (R/Q) | CXCR4-USING |
| Dots indicate residues identical to the major baseline sequence Envs; dashes indicate gaps.  ^a^gp120 V3 loop sequence (equivalent to position 296 to 331 in HXB2 NCBI accession number K03455).  ^b^Genotypic tropism was assessed using the 11/25 rule and the Geno2Pheno algorithm with cut-off <10% FPR=CXCR4-using**.** | | | | | | | | | |

| PID T377 Week 4 | | | | | | | | | |
| --- | --- | --- | --- | --- | --- | --- | --- | --- | --- |
| **Clone** | **V3 sequence^a^** | **Confirmation assay**  **Trofile** | | | **Pre-screening**  **infectivity assessment** | | | **Genotypic prediction^b^** | |
|  |  | **Reported tropism** | **R5 RLU** | **X4 RLU** | **Result** | **R5 score** | **X4 score** | **11/25 rule** | **g2p 10% FPR** |
| 1 | CTRLSSYTKRRIQIGPGRSFYTAKEVQGDLRQAHC | X4 | 52 | 4844 | X4 | 0 | 2 | Y (R/E) | CXCR4-USING |
| 4 | ................................... | X4 | 50 | 284012 | X4 | 0 | 4 | Y (R/E) | CXCR4-USING |
| 7 | ..........G........................ | X4 | 79 | 96387 | X4 | 0 | 3 | N (G/E) | CXCR4-USING |
| 9 | ......H............................ | X4 | 82 | 350091 | X4 | 0 | 3 | Y (R/E) | CXCR4-USING |
| 12 | ................................... | X4 | 65 | 123427 | X4 | 0 | 3 | Y (R/E) | CXCR4-USING |
| 14 | ................................... | X4 | 80 | 299838 | X4 | 0 | 4 | Y (R/E) | CXCR4-USING |
| 21 | ................................... | X4 | 88 | 682798 | X4 | 0 | 4 | Y (R/E) | CXCR4-USING |
| 29 | ................................... | F | 80 | 93 | R5 | 1 | 0 | Y (R/E) | CXCR4-USING |
| 35 | ................................... | X4 | 73 | 579113 | X4 | 0 | 4 | Y (R/E) | CXCR4-USING |
| 39 | ................................... | X4 | 105 | 372596 | X4 | 0 | 4 | Y (R/E) | CXCR4-USING |
| 44 | ................................... | X4 | 107 | 1239601 | X4 | 0 | 4 | Y (R/E) | CXCR4-USING |
| 47 | .....................AT............ | X4 | 72 | 1868 | X4 | 0 | 1 | Y (R/E) | CXCR4-USING |
| Dots indicate residues identical to the major baseline sequence Envs; dashes indicate gaps.  ^a^gp120 V3 loop sequence (equivalent to position 296 to 331 in HXB2 NCBI accession number K03455).  ^b^Genotypic tropism was assessed using the 11/25 rule and the Geno2Pheno algorithm with cut-off <10% FPR=CXCR4-using**.** | | | | | | | | | |

| PID T397 Day 1 | | | | | | | | | |
| --- | --- | --- | --- | --- | --- | --- | --- | --- | --- |
| **Clone** | **V3 sequence^a^** | **Confirmation assay**  **Trofile** | | | **Pre-screening**  **infectivity assessment** | | | **Genotypic prediction^b^** | |
|  |  | **Reported tropism** | **R5 RLU** | **X4 RLU** | **Result** | **R5 score** | **X4 score** | **11/25 rule** | **g2p 10% FPR** |
| 1 | CTRPGNNTSKSISIGPGRAFYARERIIGNIRQAHC | R5 | 111606 | 131 | DU | 4 | 1 | Y (S/R) | CXCR4-USING |
| 6 | ................................... | DU | 25470 | 7359 | DU | 3 | 2 | Y (S/R) | CXCR4-USING |
| 41 | .........RRV........L.......D.KK... | DU | 348267 | 136149 | DU | 4 | 3 | Y (R/R) | CXCR4-USING |
| 48 | ................................... | R5 | 546650 | 73 | R5 | 5 | 0 | Y (S/R) | CXCR4-USING |
| 60 | ................................... | DU | 2559239 | 8744 | DU | 5 | 2 | Y (S/R) | CXCR4-USING |
| 89 | ................................... | DU | 764543 | 705 | DU | 5 | 1 | Y (S/R) | CXCR4-USING |
| 109 | ................................... | DU | 351024 | 195 | DU | 4 | 1 | Y (S/R) | CXCR4-USING |
| 115 | ..............................S.... | R5 | 202228 | 94 | R5 | 4 | 0 | Y (S/R) | CXCR4-USING |
| 154 | ............................S...... | R5 | 943169 | 54 | R5 | 4 | 0 | Y (S/R) | CXCR4-USING |
| 155 | .........RRV........L.......D.KK... | DU | 590617 | 254627 | DU | 4 | 4 | Y (R/R) | CXCR4-USING |
| 171 | .........RRV........L.......D.KK... | DU | 624399 | 217636 | DU | 4 | 3 | Y (R/R) | CXCR4-USING |
| 185 | .I................................. | DU | 717478 | 2119 | DU | 4 | 1 | Y (S/R) | CXCR4-USING |
| Dots indicate residues identical to the major baseline sequence Envs; dashes indicate gaps.  ^a^gp120 V3 loop sequence (equivalent to position 296 to 331 in HXB2 NCBI accession number K03455).  ^b^Genotypic tropism was assessed using the 11/25 rule and the Geno2Pheno algorithm with cut-off <10% FPR=CXCR4-using**.** | | | | | | | | | |

| PID T397 Week 4 | | | | | | | | | |
| --- | --- | --- | --- | --- | --- | --- | --- | --- | --- |
| **Clone** | **V3 sequence^a^** | **Confirmation assay**  **Trofile** | | | **Pre-screening**  **infectivity assessment** | | | **Genotypic prediction^b^** | |
|  |  | **Reported tropism** | **R5 RLU** | **X4 RLU** | **Result** | **R5 score** | **X4 score** | **11/25 rule** | **g2p 10% FPR** |
| 1 | CTRPGNNTSKSISIGPGRAFYARERIIGNIRQAHC | DU | 158745 | 657 | DU | 4 | 1 | Y (S/R) | CXCR4-USING |
| 5 | ................................... | R5 | 75276 | 108 | R5 | 4 | 0 | Y (S/R) | CXCR4-USING |
| 6 | .........RR...............T......R. | DU | 249130 | 59996 | DU | 4 | 3 | Y (R/R) | CXCR4-USING |
| 7 | ..................V................ | DU | 166721 | 429 | R5 | 4 | 0 | Y (S/R) | CXCR4-USING |
| 14 | .........RR...............T........ | DU | 34021 | 3385 | DU | 3 | 2 | Y (R/R) | CXCR4-USING |
| 19 | ................................... | R5 | 288315 | 118 | DU | 4 | 1 | Y (S/R) | CXCR4-USING |
| 26 | .........RR...............T........ | DU | 618393 | 211265 | DU | 5 | 4 | Y (R/R) | CXCR4-USING |
| 29 | ................................... | DU | 292383 | 724 | DU | 4 | 1 | Y (S/R) | CXCR4-USING |
| 34 | .........RR...............T........ | DU | 375947 | 110090 | DU | 5 | 4 | Y (R/R) | CXCR4-USING |
| 40 | .........RR...............T........ | DU | 578246 | 212985 | DU | 5 | 3 | Y (R/R) | CXCR4-USING |
| 44 | .........RR...............T........ | DU | 1259 | 180 | R5 | 2 | 0 | Y (R/R) | CXCR4-USING |
| 47 | ................................... | DU | 301747 | 1027 | DU | 4 | 1 | Y (S/R) | CXCR4-USING |
| Dots indicate residues identical to the major baseline sequence Envs; dashes indicate gaps.  ^a^gp120 V3 loop sequence (equivalent to position 296 to 331 in HXB2 NCBI accession number K03455).  ^b^Genotypic tropism was assessed using the 11/25 rule and the Geno2Pheno algorithm with cut-off <10% FPR=CXCR4-using**.** | | | | | | | | | |

**Participants Whose CXCR4-Using Virus On-Treatment Was Not Related to a Component of the Pre-treatment Virus Population**

| PID T57 Day 1 | | | | | | | | | |
| --- | --- | --- | --- | --- | --- | --- | --- | --- | --- |
| **Clone** | **V3 sequence^a^** | **Confirmation assay**  **Trofile** | | | **Pre-screening**  **infectivity assessment** | | | **Genotypic prediction^b^** | |
|  |  | **Reported tropism** | **R5 RLU** | **X4 RLU** | **Result** | **R5 score** | **X4 score** | **11/25 rule** | **g2p 10% FPR** |
| 5 | CTRPNNNTRKSLNMGPGRAIYATGDIIGDIRQAHC | R5 | 11771 | 89 | R5 | 3 | 0 | N (S/D) | CXCR4-USING |
| 45 | ................................... | R5 | 3462 | 78 | R5 | 2 | 0 | N (S/D) | CXCR4-USING |
| 57 | ....................F.............. | R5 | 830582 | 132 | R5 | 4 | 0 | N (S/D) | CXCR4-USING |
| 60 | ................................... | R5 | 2098 | 63 | R5 | 2 | 0 | N (S/D) | CXCR4-USING |
| 63 | ...............................R... | R5 | 862988 | 81 | R5 | 4 | 0 | N (S/D) | CXCR4-USING |
| 86 | ..............E.................... | R5 | 1665042 | 86 | DU | 4 | 1 | N (S/D) | CXCR4-USING |
| 89 | ................................... | R5 | 1164090 | 96 | R5 | 4 | 0 | N (S/D) | CXCR4-USING |
| 112 | ................................... | R5 | 1552590 | 163 | R5 | 4 | 0 | N (S/D) | CXCR4-USING |
| 121 | ................................... | R5 | 126861 | 89 | R5 | 3 | 0 | N (S/D) | CXCR4-USING |
| 167 | ................................... | F | 146 | 79 | X4 | 0 | 1 | N (S/D) | CXCR4-USING |
| 180 | ..............E.................... | R5 | 1916341 | 87 | R5 | 5 | 0 | N (S/D) | CXCR4-USING |
| Dots indicate residues identical to the major baseline sequence Envs; dashes indicate gaps.  ^a^gp120 V3 loop sequence (equivalent to position 296 to 331 in HXB2 NCBI accession number K03455).  ^b^Genotypic tropism was assessed using the 11/25 rule and the Geno2Pheno algorithm with cut-off <10% FPR=CXCR4-using**.** | | | | | | | | | |

| PID T57 Week 24 (22 weeks) | | | | | | | | | |
| --- | --- | --- | --- | --- | --- | --- | --- | --- | --- |
| **Clone** | **V3 sequence^a^** | **Confirmation assay**  **Trofile** | | | **Pre-screening**  **infectivity assessment** | | | **Genotypic prediction^b^** | |
|  |  | **Reported tropism** | **R5 RLU** | **X4 RLU** | **Result** | **R5 score** | **X4 score** | **11/25 rule** | **g2p 10% FPR** |
| 1 | CTRPNNNTRKSLNMGPGRAIYATGDIIGDIRQAHC | R5 | 6191 | 79 | R5 | 3 | 0 | N (S/D) | CXCR4-USING |
| 3 | ......Y....VR..L..SF..RKA........Y. | X4 | 70 | 64916 | X4 | 0 | 2 | N (S/A) | CXCR4-USING |
| 4 | ................................... | F | 267 | 74 | R5 | 1 | 0 | N (S/D) | CXCR4-USING |
| 25 | ......Y....VR..L..SF..RKA........Y. | DU | 263 | 49658 | X4 | 0 | 2 | N (S/A) | CXCR4-USING |
| 27 | ......Y....VR..L..SF..RKA........Y. | X4 | 50 | 74337 | X4 | 0 | 3 | N (S/A) | CXCR4-USING |
| 30 | ................................... | R5 | 1918565 | 98 | R5 | 4 | 0 | N (S/D) | CXCR4-USING |
| 36 | ................................... | R5 | 1069191 | 100 | R5 | 5 | 0 | N (S/D) | CXCR4-USING |
| 38 | ................................... | R5 | 1004899 | 140 | R5 | 4 | 0 | N (S/D) | CXCR4-USING |
| 41 | ......Y....VR..L..SF..RKA........Y. | X4 | 66 | 686562 | X4 | 0 | 4 | N (S/A) | CXCR4-USING |
| 44 | ......Y....VR..L..SF..RKA........Y. | X4 | 78 | 628631 | X4 | 0 | 3 | N (S/A) | CXCR4-USING |
| 46 | ......Y....VR..L..SF..RKA........Y. | DU | 205446 | 15277 | DU | 4 | 2 | N (S/A) | CXCR4-USING |
| 47 | ......Y....VR..L..SF..RKA........Y. | X4 | 85 | 1176443 | X4 | 0 | 4 | N (S/A) | CXCR4-USING |
| Dots indicate residues identical to the major baseline sequence Envs; dashes indicate gaps.  ^a^gp120 V3 loop sequence (equivalent to position 296 to 331 in HXB2 NCBI accession number K03455).  ^b^Genotypic tropism was assessed using the 11/25 rule and the Geno2Pheno algorithm with cut-off <10% FPR=CXCR4-using**.** | | | | | | | | | |

| PID T210 Day 1 | | | | | | | | | |
| --- | --- | --- | --- | --- | --- | --- | --- | --- | --- |
| **Clone** | **V3 sequence^a^** | **Confirmation assay**  **Trofile** | | | **Pre-screening**  **infectivity assessment** | | | **Genotypic prediction^b^** | |
|  |  | **Reported tropism** | **R5 RLU** | **X4 RLU** | **Result** | **R5 score** | **X4 score** | **11/25 rule** | **g2p 10% FPR** |
| 8 | CTRPHHTVRRRIHIGPGRAFYTTSAISG~PRYVHC | X4 | 86 | 116370 | X4 | 0 | 3 | Y (R/A) | CXCR4-USING |
| 25 | ........K...................~...... | DU | 301 | 879185 | DU | 1 | 4 | Y (R/A) | CXCR4-USING |
| 35 | ....NNNT.KS.T........A.GG.I.DI.KAY. | R5 | 135253 | 90 | R5 | 4 | 0 | N (S/G) | R5 |
| 64 | ....NNNT.KS.S........A.GG.I.DI.KAY. | R5 | 6445 | 110 | R5 | 2 | 0 | N (S/G) | R5 |
| 72 | ....NNNT.KS.T........A.GG.I.DI.KAY. | R5 | 13165 | 92 | R5 | 3 | 0 | N (S/G) | R5 |
| 78 | ....NNNT.K..T........A.GG.I.DI.KAY. | R5 | 6979 | 118 | R5 | 2 | 0 | Y (R/G) | CXCR4-USING |
| 114 | ....NNNT.KS.S........A.GG.I.DI.KAY. | R5 | 262357 | 82 | R5 | 4 | 0 | N (S/G) | R5 |
| 117 | ....NNNT.KS.S........A.GG.I.DI.KAY. | R5 | 6814 | 86 | R5 | 3 | 0 | N (S/G) | R5 |
| 130 | ....NNNT.KS.S........A.GG.I.DI.KAY. | R5 | 1012290 | 102 | R5 | 5 | 0 | N (S/G) | R5 |
| 140 | ....NNNT.KS.S........A.GG.I.DI.KAY. | R5 | 106566 | 77 | R5 | 3 | 0 | N (S/G) | R5 |
| 155 | ....NNNT.KS.T........A.GG.I.DI.KAY. | R5 | 512069 | 53 | R5 | 4 | 0 | N (S/G) | R5 |
| 166 | ....NNNT.KS.S........A.GG.I.DI.KAY. | R5 | 477790 | 99 | R5 | 4 | 0 | N (S/G) | R5 |
| Dots indicate residues identical to the major baseline sequence Envs; dashes indicate gaps.  ^a^gp120 V3 loop sequence (equivalent to position 296 to 331 in HXB2 NCBI accession number K03455).  ^b^Genotypic tropism was assessed using the 11/25 rule and the Geno2Pheno algorithm with cut-off <10% FPR=CXCR4-using**.** | | | | | | | | | |

| PID T210 Week 4 | | | | | | | | | |
| --- | --- | --- | --- | --- | --- | --- | --- | --- | --- |
| **Clone** | **V3 sequence^a^** | **Confirmation assay**  **Trofile** | | | **Pre-screening**  **infectivity assessment** | | | **Genotypic prediction^b^** | |
|  |  | **Reported tropism** | **R5 RLU** | **X4 RLU** | **Result** | **R5 score** | **X4 score** | **11/25 rule** | **g2p 10% FPR** |
| 2 | CTRPYETQIKRRIYIGQGRAFSATKQVVGDPRKAYC | X4 | 76 | 50555 | X4 | 0 | 3 | Y (R/Q) | CXCR4-USING |
| 8 | .................................... | X4 | 50 | 221142 | X4 | 0 | 3 | Y (R/Q) | CXCR4-USING |
| 10 | .................................... | X4 | 66 | 27075 | X4 | 0 | 2 | Y (R/Q) | CXCR4-USING |
| 12 | .................................... | X4 | 87 | 1437084 | X4 | 0 | 4 | Y (R/Q) | CXCR4-USING |
| 19 | .................................... | X4 | 82 | 583066 | X4 | 0 | 4 | Y (R/Q) | CXCR4-USING |
| 25 | .................................... | X4 | 63 | 856411 | DU | 1 | 4 | Y (R/Q) | CXCR4-USING |
| 27 | .................................... | X4 | 131 | 1074319 | DU | 1 | 4 | Y (R/Q) | CXCR4-USING |
| 28 | .................................... | X4 | 77 | 1377780 | DU | 1 | 4 | Y (R/Q) | CXCR4-USING |
| 33 | .................................... | X4 | 50 | 503796 | X4 | 0 | 4 | Y (R/Q) | CXCR4-USING |
| 35 | .................................... | X4 | 55 | 41856 | X4 | 0 | 2 | Y (R/Q) | CXCR4-USING |
| 38 | .................................... | X4 | 101 | 923329 | DU | 1 | 4 | Y (R/Q) | CXCR4-USING |
| 47 | .................................... | X4 | 75 | 1207862 | X4 | 0 | 4 | Y (R/Q) | CXCR4-USING |
| Dots indicate residues identical to the major baseline sequence Envs; dashes indicate gaps.  ^a^gp120 V3 loop sequence (equivalent to position 296 to 331 in HXB2 NCBI accession number K03455).  ^b^Genotypic tropism was assessed using the 11/25 rule and the Geno2Pheno algorithm with cut-off <10% FPR=CXCR4-using**.** | | | | | | | | | |

| PID T398 Day 1 | | | | | | | | | |
| --- | --- | --- | --- | --- | --- | --- | --- | --- | --- |
| **Clone** | **V3 sequence^a^** | **Confirmation assay**  **Trofile** | | | **Pre-screening**  **infectivity assessment** | | | **Genotypic prediction^b^** | |
|  |  | **Reported tropism** | **R5 RLU** | **X4 RLU** | **Result** | **R5 score** | **X4 score** | **11/25 rule** | **g2p 10% FPR** |
| 8 | RTRPNNNTRKSIPMGPGQAIYATGAIIGDIRQAHC | R5 | 24272 | 58 | R5 | 3 | 0 | N (S/A) | R5 |
| 26 | C....D......................N...... | F | 312 | 82 | R5 | 2 | 0 | N (S/A) | R5 |
| 33 | C.................................. | R5 | 1262254 | 81 | R5 | 5 | 0 | N (S/A) | R5 |
| 42 | C.................................. | R5 | 27143 | 81 | DU | 3 | 1 | N (S/A) | R5 |
| 91 | C....D............................. | R5 | 1140490 | 78 | R5 | 5 | 0 | N (S/A) | R5 |
| 111 | C..........T....................... | R5 | 65345 | 55 | R5 | 4 | 0 | N (S/A) | R5 |
| 144 | C...........................N...... | R5 | 264950 | 56 | R5 | 5 | 0 | N (S/A) | R5 |
| 145 | C....D......................N...... | R5 | 89298 | 58 | R5 | 3 | 0 | N (S/A) | R5 |
| 150 | C.................................. | R5 | 32528 | 64 | DU | 5 | 1 | N (S/A) | R5 |
| 169 | C..........M...............RN.T.... | R5 | 57152 | 110 | R5 | 3 | 0 | N (S/A) | R5 |
| 179 | C.................................. | R5 | 621444 | 97 | R5 | 5 | 0 | N (S/A) | R5 |
| 191 | C...........................N...... | R5 | 91467 | 60 | R5 | 4 | 0 | N (S/A) | R5 |
| Dots indicate residues identical to the major baseline sequence Envs; dashes indicate gaps.  ^a^gp120 V3 loop sequence (equivalent to position 296 to 331 in HXB2 NCBI accession number K03455).  ^b^Genotypic tropism was assessed using the 11/25 rule and the Geno2Pheno algorithm with cut-off <10% FPR=CXCR4-using**.** | | | | | | | | | |

| PID T398 Week 4 | | | | | | | | | |
| --- | --- | --- | --- | --- | --- | --- | --- | --- | --- |
| **Clone** | **V3 sequence^a^** | **Confirmation assay**  **Trofile** | | | **Pre-screening**  **infectivity assessment** | | | **Genotypic prediction^b^** | |
|  |  | **Reported tropism** | **R5 RLU** | **X4 RLU** | **Result** | **R5 score** | **X4 score** | **11/25 rule** | **g2p 10% FPR** |
| 1 | CTRPNNNTRRKIHIGPRHGQVMYATEIIGNIRQAHC | DU | 16450 | 102474 | DU | 3 | 3 | Y (K/E) | CXCR4-USING |
| 3 | .................................... | X4 | 123 | 291660 | X4 | 0 | 4 | Y (K/E) | CXCR4-USING |
| 10 | .................................... | DU | 43912 | 836577 | DU | 3 | 4 | Y (K/E) | CXCR4-USING |
| 13 | .................................... | DU | 395664 | 1119707 | DU | 4 | 4 | Y (K/E) | CXCR4-USING |
| 23 | .................................... | DU | 179998 | 803160 | DU | 4 | 4 | Y (K/E) | CXCR4-USING |
| 27 | .................................... | DU | 10522 | 26299 | DU | 2 | 2 | Y (K/E) | CXCR4-USING |
| 33 | .................................... | DU | 2256 | 8515 | DU | 2 | 2 | Y (K/E) | CXCR4-USING |
| 36 | .................................... | X4 | 70 | 44392 | X4 | 0 | 3 | Y (K/E) | CXCR4-USING |
| 37 | ......................H............. | DU | 52116 | 225436 | DU | 4 | 3 | Y (K/E) | CXCR4-USING |
| 40 | .................................... | DU | 30956 | 330078 | DU | 4 | 4 | Y (K/E) | CXCR4-USING |
| 42 | ..........................T......... | DU | 20245 | 1330420 | DU | 3 | 4 | Y (K/E) | CXCR4-USING |
| 44 | .................................... | DU | 140391 | 807668 | DU | 4 | 4 | Y (K/E) | CXCR4-USING |
| Dots indicate residues identical to the major baseline sequence Envs; dashes indicate gaps.  ^a^gp120 V3 loop sequence (equivalent to position 296 to 331 in HXB2 NCBI accession number K03455).  ^b^Genotypic tropism was assessed using the 11/25 rule and the Geno2Pheno algorithm with cut-off <10% FPR=CXCR4-using**.** | | | | | | | | | |

| PID T415 Day 1 | | | | | | | | | |
| --- | --- | --- | --- | --- | --- | --- | --- | --- | --- |
| **Clone** | **V3 sequence^a^** | **Confirmation assay**  **Trofile** | | | **Pre-screening**  **infectivity assessment** | | | **Genotypic prediction^b^** | |
|  |  | **Reported tropism** | **R5 RLU** | **X4 RLU** | **Result** | **R5 score** | **X4 score** | **11/25 rule** | **g2p 10% FPR** |
| 29 | CTRHNNNTRKSINIGPGRAWYTTGDITEDIRQAYC | R5 | 2409 | 77 | R5 | 2 | 0 | N (S/D) | R5 |
| 33 | .........................V.G....... | F | 857 | 76 | R5 | 1 | 0 | N (S/D) | R5 |
| 77 | ...........................G....... | R5 | 709576 | 85 | R5 | 4 | 0 | N (S/D) | R5 |
| 96 | ...Y.......................G....... | R5 | 69626 | 72 | R5 | 4 | 0 | N (S/D) | R5 |
| 102 | ...........................G....... | F | 233 | 64 | R5 | 2 | 0 | N (S/D) | R5 |
| 112 | .........R.................G....... | R5 | 21470 | 76 | R5 | 3 | 0 | N (S/D) | R5 |
| 120 | .........................S.G....... | F | 98 | 64 | R5 | 1 | 0 | N (S/D) | R5 |
| 137 | ...........................G....... | R5 | 42318 | 59 | R5 | 3 | 0 | N (S/D) | R5 |
| 138 | ...........................G....... | F | 388 | 53 | R5 | 1 | 0 | N (S/D) | R5 |
| 140 | .........R.................G....... | R5 | 344955 | 73 | R5 | 4 | 0 | N (S/D) | R5 |
| 152 | ...........................G...R... | R5 | 302402 | 84 | R5 | 4 | 0 | N (S/D) | R5 |
| 177 | ...........................G...K... | R5 | 1671 | 69 | R5 | 2 | 0 | N (S/D) | R5 |
| Dots indicate residues identical to the major baseline sequence Envs; dashes indicate gaps.  ^a^gp120 V3 loop sequence (equivalent to position 296 to 331 in HXB2 NCBI accession number K03455).  ^b^Genotypic tropism was assessed using the 11/25 rule and the Geno2Pheno algorithm with cut-off <10% FPR=CXCR4-using**.** | | | | | | | | | |

| PID T415 Week 4 | | | | | | | | | |
| --- | --- | --- | --- | --- | --- | --- | --- | --- | --- |
| **Clone** | **V3 sequence^a^** | **Confirmation assay**  **Trofile** | | | **Pre-screening**  **infectivity assessment** | | | **Genotypic prediction^b^** | |
|  |  | **Reported tropism** | **R5 RLU** | **X4 RLU** | **Result** | **R5 score** | **X4 score** | **11/25 rule** | **g2p 10% FPR** |
| 3 | CTRPGTKMIMRSMHIGPGRVFLTKDIEGDIRKASC | X4 | 63 | 114925 | X4 | 0 | 3 | N (S/D) | CXCR4-USING |
| 4 | ................................... | X4 | 58 | 74965 | DU | 1 | 3 | N (S/D) | CXCR4-USING |
| 12 | .....A.~........................... | X4 | 57 | 389849 | X4 | 0 | 4 | N (S/D) | CXCR4-USING |
| 14 | ..........................G........ | X4 | 73 | 30813 | X4 | 0 | 3 | N (S/D) | CXCR4-USING |
| 19 | ................................... | X4 | 71 | 148663 | X4 | 0 | 3 | N (S/D) | CXCR4-USING |
| 24 | .......~........................... | F | 54 | 77 | R5 | 1 | 0 | N (S/D) | CXCR4-USING |
| 29 | ................................... | X4 | 52 | 132003 | X4 | 0 | 3 | N (S/D) | CXCR4-USING |
| 33 | ................................... | X4 | 58 | 53479 | X4 | 0 | 3 | N (S/D) | CXCR4-USING |
| 41 | ................................... | X4 | 79 | 222894 | X4 | 0 | 3 | N (S/D) | CXCR4-USING |
| 42 | ................................... | X4 | 77 | 3499 | X4 | 0 | 1 | N (S/D) | CXCR4-USING |
| 46 | ................................... | F | 50 | 247 | X4 | 0 | 1 | N (S/D) | CXCR4-USING |
| 47 | ................................... | X4 | 58 | 170962 | X4 | 0 | 3 | N (S/D) | CXCR4-USING |
| Dots indicate residues identical to the major baseline sequence Envs; dashes indicate gaps.  ^a^gp120 V3 loop sequence (equivalent to position 296 to 331 in HXB2 NCBI accession number K03455).  ^b^Genotypic tropism was assessed using the 11/25 rule and the Geno2Pheno algorithm with cut-off <10% FPR=CXCR4-using**.** | | | | | | | | | |

| PID T629 Day 1 | | | | | | | | | |
| --- | --- | --- | --- | --- | --- | --- | --- | --- | --- |
| **Clone** | **V3 sequence^a^** | **Confirmation assay**  **Trofile** | | | **Pre-screening**  **infectivity assessment** | | | **Genotypic prediction^b^** | |
|  |  | **Reported tropism** | **R5 RLU** | **X4 RLU** | **Result** | **R5 score** | **X4 score** | **11/25 rule** | **g2p 10% FPR** |
| 14 | CVRPNNNTVKGIHIGLGRAFYTTGQIVGNIRQAHC | R5 | 4281 | 89 | R5 | 2 | 0 | N (G/Q) | R5 |
| 24 | ...............P........L.......... | R5 | 4770 | 93 | R5 | 2 | 0 | N (G/L) | R5 |
| 28 | ...............P................... | R5 | 391443 | 92 | R5 | 4 | 0 | N (G/Q) | R5 |
| 35 | ...............P................... | R5 | 722547 | 270 | DU | 4 | 1 | N (G/Q) | R5 |
| 59 | ...............P................... | R5 | 184781 | 226 | DU | 4 | 1 | N (G/Q) | R5 |
| 68 | ...............P................... | R5 | 21355 | 102 | R5 | 2 | 0 | N (G/Q) | R5 |
| 94 | ...............P................... | R5 | 305125 | 121 | DU | 4 | 1 | N (G/Q) | R5 |
| 107 | ...............P................... | R5 | 834837 | 112 | R5 | 4 | 0 | N (G/Q) | R5 |
| 139 | ...............P................... | DU | 400492 | 446 | DU | 4 | 1 | N (G/Q) | R5 |
| 178 | ...............P................... | R5 | 256872 | 155 | DU | 4 | 1 | N (G/Q) | R5 |
| 192 | ...............P............D...... | R5 | 131084 | 93 | R5 | 4 | 0 | N (G/Q) | R5 |
| Dots indicate residues identical to the major baseline sequence Envs; dashes indicate gaps.  ^a^gp120 V3 loop sequence (equivalent to position 296 to 331 in HXB2 NCBI accession number K03455).  ^b^Genotypic tropism was assessed using the 11/25 rule and the Geno2Pheno algorithm with cut-off <10% FPR=CXCR4-using**.** | | | | | | | | | |

| PID T629 Week 4 | | | | | | | | | |
| --- | --- | --- | --- | --- | --- | --- | --- | --- | --- |
| **Clone** | **V3 sequence^a^** | **Confirmation assay**  **Trofile** | | | **Pre-screening**  **infectivity assessment** | | | **Genotypic prediction^b^** | |
|  |  | **Reported tropism** | **R5 RLU** | **X4 RLU** | **Result** | **R5 score** | **X4 score** | **11/25 rule** | **g2p 10% FPR** |
| 8 | CARPNNNTVKGIGIGPGRTVYTAEKIVGNIRQAHC | DU | 289310 | 1255080 | DU | 4 | 4 | Y (G/K) | CXCR4-USING |
| 13 | ....D.............................. | DU | 1650 | 71965 | DU | 2 | 3 | Y (G/K) | CXCR4-USING |
| 24 | .........R......................... | F | 122 | 373 | X4 | 0 | 1 | Y (G/K) | CXCR4-USING |
| 27 | .........R......................... | DU | 225941 | 955634 | DU | 4 | 4 | Y (G/K) | CXCR4-USING |
| 31 | ................................... | DU | 119416 | 707847 | DU | 4 | 4 | Y (G/K) | CXCR4-USING |
| 34 | .........R......................... | DU | 1128 | 11499 | DU | 2 | 3 | Y (G/K) | CXCR4-USING |
| 38 | .........R......................... | DU | 47885 | 334985 | DU | 3 | 4 | Y (G/K) | CXCR4-USING |
| 40 | ................................... | F | 345 | 554 | X4 | 0 | 2 | Y (G/K) | CXCR4-USING |
| 41 | ................................... | DU | 1497 | 47745 | DU | 3 | 3 | Y (G/K) | CXCR4-USING |
| 44 | ................................... | DU | 61096 | 340057 | DU | 3 | 4 | Y (G/K) | CXCR4-USING |
| 47 | ................................... | DU | 383155 | 1587447 | DU | 4 | 4 | Y (G/K) | CXCR4-USING |
| Dots indicate residues identical to the major baseline sequence Envs; dashes indicate gaps.  ^a^gp120 V3 loop sequence (equivalent to position 296 to 331 in HXB2 NCBI accession number K03455).  ^b^Genotypic tropism was assessed using the 11/25 rule and the Geno2Pheno algorithm with cut-off <10% FPR=CXCR4-using**.** | | | | | | | | | |

| PID T825 Day 1 | | | | | | | | | |
| --- | --- | --- | --- | --- | --- | --- | --- | --- | --- |
| **Clone** | **V3 sequence^a^** | **Confirmation assay**  **Trofile** | | | **Pre-screening**  **infectivity assessment** | | | **Genotypic prediction^b^** | |
|  |  | **Reported tropism** | **R5 RLU** | **X4 RLU** | **Result** | **R5 score** | **X4 score** | **11/25 rule** | **g2p 10% FPR** |
| 2 | CTRPSNNTRKGIHIGPGSTFFATDIIGDIRQAHC | R5 | 173208 | 82 | R5 | 4 | 0 | N (G/D) | R5 |
| 7 | .................................. | R5 | 1339490 | 407 | DU | 5 | 1 | N (G/D) | R5 |
| 34 | .................................. | R5 | 1477642 | 585 | DU | 5 | 1 | N (G/D) | R5 |
| 37 | .................................. | R5 | 14222 | 69 | R5 | 3 | 0 | N (G/D) | R5 |
| 55 | .................................. | R5 | 81393 | 98 | R5 | 4 | 0 | N (G/D) | R5 |
| 58 | .................................. | R5 | 1910242 | 340 | DU | 4 | 1 | N (G/D) | R5 |
| 72 | .................................. | R5 | 275392 | 103 | R5 | 4 | 0 | N (G/D) | R5 |
| 91 | .................................. | R5 | 1409148 | 433 | DU | 5 | 1 | N (G/D) | R5 |
| 109 | .................................. | F | 322 | 83 | R5 | 2 | 0 | N (G/D) | R5 |
| 128 | .................R................ | R5 | 391217 | 52 | R5 | 4 | 0 | N (G/D) | R5 |
| 167 | .................................. | R5 | 11751 | 69 | R5 | 3 | 0 | N (G/D) | R5 |
| Dots indicate residues identical to the major baseline sequence Envs; dashes indicate gaps.  ^a^gp120 V3 loop sequence (equivalent to position 296 to 331 in HXB2 NCBI accession number K03455).  ^b^Genotypic tropism was assessed using the 11/25 rule and the Geno2Pheno algorithm with cut-off <10% FPR=CXCR4-using**.** | | | | | | | | | |

| PID T825 Week 4 | | | | | | | | | |
| --- | --- | --- | --- | --- | --- | --- | --- | --- | --- |
| **Clone** | **V3 sequence^a^** | **Confirmation assay**  **Trofile** | | | **Pre-screening**  **infectivity assessment** | | | **Genotypic prediction^b^** | |
|  |  | **Reported tropism** | **R5 RLU** | **X4 RLU** | **Result** | **R5 score** | **X4 score** | **11/25 rule** | **g2p 10% FPR** |
| 3 | CIRPGNNTKKFIHLGPRRGAFYATHNIGDARQAHC | X4 | 81 | 217871 | DU | 1 | 4 | N (F/N) | CXCR4-USING |
| 9 | ................................... | X4 | 92 | 32041 | DU | 1 | 3 | N (F/N) | CXCR4-USING |
| 12 | ......T............................ | X4 | 54 | 37366 | DU | 1 | 3 | N (F/N) | CXCR4-USING |
| 13 | ................................... | X4 | 66 | 21446 | X4 | 0 | 2 | N (F/N) | CXCR4-USING |
| 16 | ................................... | F | 86 | 124 | DU | 2 | 4 | N (F/N) | CXCR4-USING |
| 17 | ................................... | X4 | 74 | 56193 | X4 | 0 | 3 | N (F/N) | CXCR4-USING |
| 18 | ................................... | DU | 261 | 189996 | DU | 1 | 4 | N (F/N) | CXCR4-USING |
| 21 | .........R......................... | X4 | 73 | 1624 | X4 | 0 | 2 | N (F/N) | CXCR4-USING |
| 22 | ................................... | DU | 415 | 533040 | DU | 2 | 4 | N (F/N) | CXCR4-USING |
| 26 | ................................... | F | 78 | 335 | X4 | 0 | 1 | N (F/N) | CXCR4-USING |
| 34 | ................................... | X4 | 94 | 181305 | DU | 2 | 4 | N (F/N) | CXCR4-USING |
| Dots indicate residues identical to the major baseline sequence Envs; dashes indicate gaps.  ^a^gp120 V3 loop sequence (equivalent to position 296 to 331 in HXB2 NCBI accession number K03455).  ^b^Genotypic tropism was assessed using the 11/25 rule and the Geno2Pheno algorithm with cut-off <10% FPR=CXCR4-using**.** | | | | | | | | | |
