# Supplementary material for: Clonal analysis of HIV-1 genotype and function associated with virologic failure in treatment-experienced persons receiving maraviroc: Results from the MOTIVATE phase 3 randomized, placebo-controlled trials
Source: PLoS One. 2018 Dec 26;13(12):e0204099. doi: 10.1371/journal.pone.0204099 (PMC6306210; doi:10.1371/journal.pone.0204099)
Supplement: S4 Table — (DOCX) [file pone.0204099.s009.docx]

**S4 Table. Summary of MVC IC_50_ FC and MPI for CCR5 Tropic Virus From 36 Participants Failing Blinded Therapy in the MOTIVATE Trials**

|  | IC_50_ FC^a^ | | MPI <95  (Number of participants) | |
| --- | --- | --- | --- | --- |
|  | BL | Fail | BL | Fail |
|  | MVC Group (N=11^b^) | | | |
| Value | 0.84 | 1 | 0 | 4 |
| Range (min-max) | 0.32-1.58 | 0.38-2.42 | 95-100 | 30-85^c^ |
|  | Control Group (N=25) | | | |
| Value | 0.67 | 0.65 | 0 | 0 |
| Range (min-max) | 0.22-2.65 | 0.22-2.82 | 96-100 | 97-100 |
| *P*-value^d^ | 0.13 | |  | |
| BL, baseline Day 1 sample; Fail, sample taken following protocol defined treatment failure( PDTF); FC JRCSF, fold change in IC_50_ between participant-derived virus and the reference strain, JRCSF, tested in the same assay; MPI, maximum percentage inhibition in dose response; MVC, maraviroc; IC50 Δ Resist.  ^a^IC_50_ Δ Resist. JRCSF reported as geometric mean values;  ^b^Excludes 2 of the 13 participants on MVC: PID 40 (MVC QD, study A4001027) who had a plateau in inhibition <50% at failure (therefore no IC50 was reached) and PID 23 (MVC BD, study A4001027) is excluded as no valid PhenoSense Susceptibility data was obtained.  ^c^Range is given for the 4 participants with MPI <95%; virus from the other 7 participants showed MPI range 96-100.  ^d^Analysis of covariance at failure (using the Day 1 response as a covariate) between treatment arm and placebo, mean fold difference between treatment groups = 0.82 (95% CI, 0.63-1.07). | | | | |
