# Supplementary material for: Clonal analysis of HIV-1 genotype and function associated with virologic failure in treatment-experienced persons receiving maraviroc: Results from the MOTIVATE phase 3 randomized, placebo-controlled trials
Source: PLoS One. 2018 Dec 26;13(12):e0204099. doi: 10.1371/journal.pone.0204099 (PMC6306210; doi:10.1371/journal.pone.0204099)
Supplement: S5 Table — (DOCX) [file pone.0204099.s010.docx]

**S5 Table. Genotypic and Phenotypic Analysis of 12 Clones From Virus Pre- and Post-Treatment Obtained From 5 Maraviroc-Treated Participants Failing With Maraviroc-Resistant R5 Virus and 4 Participants Receiving Placebo and Failing with R5 Virus**

| **Participant identifier** | **VISIT**  **(MPI pool)** | **No. of clones**  **(MPI range)** | **V3 sequence^a^** |
| --- | --- | --- | --- |
| PID 3 | DAY 1 (100) | 10(98-100) | CTRPNNNTRKSIPIG-PGRAFYATGDIIGDIRQAHC |
|  |  | 1(96) | ............S..-.................... |
|  |  | 1(100) | ...............-...........V........ |
|  | WEEK 24 (30) MVC | 10(36-67) | ............S..A.................... |
|  |  | 1(77) | ............S..A..............V..... |
| PID 12 | Day 1 (100) | 4(95-100) | CIRPNNNTRKGIHIGPGRSFYATGDIIGDIRQAHC |
|  |  | 2(95-97) | .T......................E.......... |
|  |  | 2(99) | ............................N...... |
|  |  | 1(100) | .T..............................V.. |
|  |  | 1(100) | ................................T.. |
|  |  | 2(100) | .T................................. |
|  | WEEK 8 (85) MVC | 11(21-79) 1(92) | ..........S..............V......... |
|  | WEEK 16 (91) OFF STUDY DRUG | 6(63-86) | ..........S..............V......... |
|  |  | 1(100) | ..........S..............V....G.... |
|  |  | 2(99) | .........................V......... |
|  |  | 1(88) | .T........S..............V......... |
|  |  | 2(99-100) | .T................................. |
|  | WEEK 32 (57) MVC | 10(58-94) | ..........S..............V......... |
|  |  | 1(NA) | NA |
|  |  | 1(70) | ..........SV.............V......... |

| **Participant identifier** | **VISIT**  **(MPI pool)** | **No. of clones**  **(MPI range)** | **V3 Sequence^a^** |
| --- | --- | --- | --- |
| PID 8 | DAY 1 (100) | 9(98-100) | CTRPGNNTRKSIHMGPGSSIYATGAIIGDIRQAHC |
|  |  | 1(100) | .....D............................. |
|  |  | 2(99) | ................E.................. |
|  | WEEK 24 (84) MVC | 8(61-84) 1(99) | ...................F....DV......... |
|  |  | 2(76-81) 1(99) | ..................RF....DV......... |
| PID 11 | DAY 1 (100) | 2(95-96) | CTRPNNNTRKSINIGPGKAWYTTGDIIGDIRQAHC |
|  |  | 1(99) | .................R................. |
|  |  | 3(100) | ..........G......R.....E........... |
|  |  | 1(100) | .I.....A..G......R.....E........... |
|  |  | 1(100) | .I.....A........................... |
|  |  | 1(97) | .I...............R................. |
|  |  | 2(99) | .I........G......R.....E........... |
|  |  | 1(96) | .I...............R................. |
|  | WEEK 8 (80) MVC | 6(50-84) | ............H........A............. |
|  |  | 1(57) | ............H.....T................ |
|  |  | 5(61-91) | ............H...................... |
| PID 21 | DAY 1 (100) | 10(100) | CTRPSNNTSKGIHMGPGKAFYATGQITGDIRRAYC |
|  |  | 1(100) | .........................M......... |
|  |  | 1(100) | .................R................. |
|  | WEEK 24 (100) MVC | 7(100) | .................R................. |
|  |  | 4(100) | .................R.............K... |
|  |  | 1(100) | .................R........I........ |

| **Participant identifier** | **VISIT**  **(MPI pool)** | **No. of clones**  **(MPI range)** | **V3 Sequence^a^** |
| --- | --- | --- | --- |
| PID 16 | DAY 1 (96) | 7(95-99) | CIRPNNNTRKSISIGPGRAFYATGDIIGDIRQAHC |
|  |  | 3(98-100) | .T..S.............................. |
|  |  | 1(99) | .................K................. |
|  |  | 1(100) | .......................E........... |
|  | WEEK 12 (98) PLACEBO | 7(98-100) | ................................... |
|  |  | 5(99-100) | .T..S.............................. |
|  | WEEK 48 (41) MVC | 9(48-57) | .T..........H.....T......V......... |
|  |  | 1(95) | .T..........H.....T.H....V......... |
|  |  | 2(52-56) | ...............A..S................ |
| PID 18 | Day 1 (96) | 8 (83-100) | CTRLNNNTRKSIQMGPGRAFYATGDIIGNIRKAHC |
|  |  | 1 (100) | ....S.............................. |
|  |  | 1 (95) | ............................D...... |
|  |  | 1 (94) | ...I........................D...... |
|  |  | 1 (100) | ....S.......................D...... |
|  | Week 12 (98) PLACEBO | 2 (80-90) | ................................... |
|  |  | 10 (99-100) | ...P........HI..........EV.....Q... |
| PID 19 | Day 1 (98) | 1 (99) | CTRPNNNTRKGIHMGPGRAFYTTGGIIGDIRQAHC |
|  |  | 7 (96-100) | .................K......E.......... |
|  |  | 1 (99) | .................K.L....E.......... |
|  |  | 1 (99) | ....S............K......E.......... |
|  |  | 2 (97-99) | ........................E.......... |
|  | WEEK 12 (98)  PLACEBO | 1 (100) | .............T..........E.......... |
|  |  | 7 (93-99) | .................K......E.......... |
|  |  | 4 (93-100) | ........................E.......... |

| **Participant Identifier** | **VISIT**  **(MPI pool)** | **No of clones**  **(MPI (%) range)** | **V3 Sequence^a^** |
| --- | --- | --- | --- |
| PID 20 | Day 1 (96) | 6 (86-92) | CTRPGNNTRRSIPMGPGKAFFTTGDIIGDIRQAHC |
|  |  | 4 (90-98) | ....................YA............. |
|  |  | 2 (92-93) | .....................A............. |
|  | Week 8 (97)  PLACEBO | 9 (94-100) | ....................YA............. |
|  |  | 2 (100) | .........K..........YA............. |
|  |  | 1 (100) | ....................YA...........Y. |
| Dots indicate residues identical to the major baseline sequence Envs; dashes indicate gaps.  ^a^gp120 V3 loop sequence (equivalent to position 296 to 331 in HXB2 (NCBI accession number K03455). | | | |
