# Supplementary material for: Clonal analysis of HIV-1 genotype and function associated with virologic failure in treatment-experienced persons receiving maraviroc: Results from the MOTIVATE phase 3 randomized, placebo-controlled trials
Source: PLoS One. 2018 Dec 26;13(12):e0204099. doi: 10.1371/journal.pone.0204099 (PMC6306210; doi:10.1371/journal.pone.0204099)
Supplement: S6 Table — (DOCX) [file pone.0204099.s011.docx]

**S6 Table. PhenoSense HIV Entry and PBL Assay Results for Maraviroc Susceptibility (IC_50_ Fold Change and MPI) From Individual Viral Clones**

| **MVC arm** | **Participant identifier** | **Study visit** | **Susceptibility to maraviroc** | | | | **V3 loop sequence^a^** |
| --- | --- | --- | --- | --- | --- | --- | --- |
|  |  |  | **PhenoSenseTM** | | **PBL** | | 10 20 30 |
|  |  |  | **IC_50_FC** | **MPI** | **IC_50_FC^b^** | **MPI^c^** | ....\|....\|....\|....\|....\|....\|....\| |
| BID | PID 12 | Day 1^d^ | 0.69 | 100 | 0.11 | 99 | CTRPNNNTRKGIHIGPGRSFYATGDIIGDIRQVHC |
|  |  | Week 8 | *>MAX* | **21** | *>MAX* | **46** | .I........S..............V......A.. |
|  | PID 21 | Day 1 | 1.28 | 100 | 0.05 | 98 | CTRPSNNTSKGIHMGPGKAFYATGQITGDIRRAYC |
|  |  | Week 24 | **2.03** | 100 | **0.25**^e^ | 106 | .................R................. |
| QD | PID 3 | Day 1 | 0.52 | 100 | 0.07 | 99 | CTRPNNNTRKSIPIG-PGRAFYATGDIIGDIRQAHC |
|  |  | E_Term | *>MAX* | **37** | *>MAX* | **-27** | ............S..A.................... |
|  | PID 8 | Day 1 | 0.25 | 100 | 0.04 | 101 | CTRPGNNTRKSIHMGPGSSIYATGAIIGDIRQAHC |
|  |  | Week 24 | 0.47 | **62** | *0.09* | **75** | ...................F....DV......... |
|  | PID 11 | Day 1 | 1.02 | 97 | 0.19 | 98 | CIRPNNNTRKSINIGPGRAWYTTGDIIGDIRQAHC |
|  |  | Week 8 | *2.3* | **50** | *0.14* | **69** | .T..........H....K...A............. |
|  |  | Week 8 | *2.2* | **61** | *0.6* | **70** | .T..........H....K................. |
| Dots indicate residues identical to the major baseline sequence Envs; dashes indicate gaps.  ^a^gp120 V3 loop sequence (equivalent to position 296 to 331 in HXB2 (NCBI accession number K03455).  ^b^Geometric mean from n=3  ^c^Mean from n=3  ^d^The presence of the V at position 33 in the V3 sequence in the Day 1 clones is atypical and present in only 1of the 12 clones, the remaining 11 clones had A at this position  ^e^Statistically significant from Day 1 (p-value=<0.01) | | | | | | | |
