# Supplementary material for: Clonal analysis of HIV-1 genotype and function associated with virologic failure in treatment-experienced persons receiving maraviroc: Results from the MOTIVATE phase 3 randomized, placebo-controlled trials
Source: PLoS One. 2018 Dec 26;13(12):e0204099. doi: 10.1371/journal.pone.0204099 (PMC6306210; doi:10.1371/journal.pone.0204099)
Supplement: S1 Protocol — (PDF) [file pone.0204099.s012.pdf]

CLINICAL PROTOCOL

A MULTICENTER, RANDOMIZED, DOUBLE-BLIND, PLACEBO-CONTROLLED TRIAL OF A NOVEL CCR5 ANTAGONIST, UK-427,857, IN COMBINATION WITH OPTIMIZED BACKGROUND THERAPY VERSUS OPTIMIZED BACKGROUND THERAPY ALONE FOR THE TREATMENT OF ANTIRETROVIRAL-EXPERIENCED HIV-1 INFECTED SUBJECTS

**Compound:**	UK-427,857	
**Compound Name:**		
**US IND Number:**	65,229	
**Protocol Number:**	A4001027	
**Phase:**	Phase 2b/3	
**Version and Date:**	Original Version Date:	24 August 2004
	Amendment #1:	05 May 2005
	Amendment #2:	20 December 2005
	Amendment #3:	02 March 2006
	Amendment #4	24 October 2007
	Amendment #5	17 June 2008
	Amendment #6	10 August 2010

This document contains confidential information belonging to ViiV Healthcare. Except as otherwise agreed to in writing, by accepting or reviewing this document, you agree to hold this information in confidence and not to disclose it to others (except where required by applicable law) or to use it for unauthorized purposes. In the event of any actual or suspected breach of this obligation, Pfizer and/or ViiV Healthcare should be promptly notified.

090177e1815d915c\Approved\Approved On: 24-Aug-2010 20:15

090177e1815d915c\Approved\Approved On: 24-Aug-2010 20:15

SUMMARY

Indication:

HIV-1 treatment in combination with other agents.

Rationale:

There is a desperate need for new drugs with novel mechanisms of action to be developed for treatment-experienced patients who have few or no remaining options due to multi-class resistance, toxicity to existing classes or both. UK-427,857, a selective and reversible CCR5 co-receptor antagonist, has been shown to be active in vitro against a wide range of clinical isolates (including those resistant to existing classes). In HIV-1 infected patients, UK-427,857 given as monotherapy for 10 days reduced HIV-1 viral load by up to 1.6 log_{10}, consistent with currently available agents comprising the cornerstone of HAART (eg, protease inhibitors, non-nucleoside reverse transcriptase inhibitors). An excellent safety and toleration profile has been demonstrated with dosing for up to 28 days at 300 mg BID, including no significant effect on QTc interval. Since most patients who are heavily antiretroviral experienced remain infected with CCR5-tropic virus only, studies with UK-427,857 in this patient population are warranted.

Objectives:

- The primary objective is to confirm the hypothesis that UK-427,857 added to Optimized Background Therapy (OBT) provides an additional reduction in plasma HIV-1 RNA compared to OBT alone, as measured by the difference between each of the two UK-427,857 regimens versus the placebo regimen in the mean changes from baseline in plasma HIV-1 RNA at Week 48. An interim analysis will be performed at Week 24.
- The secondary objectives include assessments of safety and tolerability of UK-427,857 when added to OBT versus OBT alone.

Trial Design:

Multi-center, double blind, randomized (2:2:1), placebo-controlled, superiority Phase 2b/3 study to determine the antiviral activity and safety of UK-427,857 (150 mg once daily and 150 mg twice daily) added to optimized background therapy, compared to optimized background therapy alone, in CCR5-tropic, HIV-1 infected subjects. As UK-427,857 is a substrate for CYP3A4 inhibition, a nominal unit dose of 150 mg is being studied, assuming that most patients will be receiving at least one PI in their background treatment regimen. This study will involve approximately 90 centers to achieve a total randomized subject population of 600 subjects.

Subjects must have had at least 6 months of prior treatment with at least one agent (two for protease inhibitors) from 3 of the 4 antiretroviral drug classes or multi-class resistance and treatment failure to an existing regimen, defined by a plasma HIV-1 RNA ≥5,000 copies/mL. All subjects will undergo genotypic and phenotypic testing for the presence of CCR5-tropic HIV-1 and for resistance to reverse transcriptase inhibitors, protease inhibitors and fusion

inhibitors. Investigators will optimize therapy, with 3-6 agents excluding low-dose ritonavir, on the basis of these resistance tests, treatment history and safety/adverse event considerations. Mean reduction in HIV-1 RNA will be assessed at 24 and 48 weeks or at the time of discontinuation from the study. All subjects who receive at least one dose of study medication will be assessed for safety. The study will enroll over approximately a 9-month period with 48 weeks of treatment. As part of this clinical study a blood sample will be taken for non-anonymized pharmacogenetic analysis.

Observational Phase

After a patient has reached their Week 48 visit, they will continue to visit the investigator site for every 12 weeks and on-study evaluations completed, until they reach their Week 96 visit, as stated in section 5.6 of the protocol.

When the last subject enrolled in the study reaches their Week 48 visit the study will be unblinded and following completion of the formal Week 48 analysis by the sponsor, open label drug will be provided to subjects for whom it is medically appropriate to continue, or begin, therapy with UK-427,857. Open label drug will be supplied to these subjects, until each subject reaches 5 years from their first dose of double-blind study treatment.

Because the study population has a diverse visit schedule, subjects entering the observational phase will be divided based on whether they have or not had a Week 96 visit at the time. Subjects may transition to the observational phase at an unplanned visit.

Subjects that will reach their Week 96 visit

Subject's reaching their Week 96 visit, (which will also be classified as their End of Study visit) this visit will mark the completion of the active phase of the study. All subjects, upon discontinuation from the active phase of the study, will begin the follow-up phase of the study. This phase of the study will include subjects being reconsented and collection of long term survival and selected endpoint (LTS/SE) data including liver failure, myocardial infarction and ischemia, malignancies, Category C events, infections reported as serious adverse events, and rhabdomyolysis. This data will be collected every six months, for a minimum of 5 years timed from first dose of double-blind study treatment. If subjects are receiving maraviroc, it will continue to be provided until the end of the observational portion of the study.

Subjects that have passed their Week 96 visit

Subject's who have passed their Week 96 visit will have their next scheduled visit as the completion of the active phase of the study. All subjects, upon discontinuation from the active phase of the study, will begin the follow-up phase of the study. This phase of the study will include subjects being reconsented and collection of LTS/SE data including liver failure, myocardial infarction and ischemia, malignancies, Category C events, infections reported as serious adverse events, and rhabdomyolysis every six months, for a minimum of 5 years timed from first dose of double-blind study treatment. If subjects are receiving maraviroc, it will continue to be provided until the end of the observational portion of the trial.

090177e1815d915c\Approved\Approved On: 24-Aug-2010 20:15

090177e1815d915c\Approved\Approved On: 24-Aug-2010 20:15

Previously Discontinued Subjects

In compliance with regulatory recommendations, an attempt (two documented telephone calls and one certified letter) will be made to follow up on subjects who have previously permanently discontinued study treatment but who are no longer returning for visits under the "*In study Off Drug*" phase, for LTS/SE data. If the subject has died any information concerning the clinical aspects and circumstances of death, death certificate, and autopsy should be obtained. This will be initiated by the investigator.

Endpoints:

Primary Endpoint:

Change from baseline to 48 weeks in HIV-1 RNA measured on a logarithmic [base 10] scale. An interim analysis will be performed at Week 24.

Secondary Endpoints:

The percentage of patients with fewer than 50/400 copies of HIV-1 RNA per milliliter of plasma, time to loss of virological response, change in CD4 and CD8 counts, changes in genotype, phenotype and/or tropism in treatment failures, % with AIDS-defining illness.

Trial Treatments:

1. Optimized Background Therapy (3-6 drugs based on treatment history and resistance testing) + UK-427,857 150 mg PO taken once daily.
2. Optimized Background Therapy (3-6 drugs based on treatment history and resistance testing) + UK-427,857 150 mg PO taken twice daily.
3. Optimized Background Therapy (3-6 drugs based on treatment history and resistance testing) + UK-427,857 placebo.

Statistical Methods:

The primary efficacy variable is the change from baseline in $\log_{10}$ transformed HIV-1 RNA levels. The primary efficacy time point is 48 weeks. The primary efficacy variable will also be analyzed at 24 weeks. An ANCOVA model will be fitted with screening viral load level (randomization strata), enfuvirtide use, geographic region and treatment arm as the main effects. The least squares mean treatment difference between each UK-427,857 dose group and placebo will be presented. The main secondary efficacy variables will be percentage of subjects responding based on a number of definitions (analyzed using statistical methods for binary data), change from baseline in CD4 cell count, change from baseline in CD8 cell count (analyzed using ANCOVA), time to virological failure (analyzed using survival methods), time averaged difference (TAD) in $\log_{10}$ HIV-1 RNA, genotype and phenotype at baseline and at the time of failure, tropism at baseline and at the time of failure and association between baseline resistance and virological response.

Schedule of Activities

090177e1815d915c\Approved\Approved On: 24-Aug-2010 20:15

VISIT SCHEDULE:

Procedures	Screening (Day – 42 to -28)	Randomization (Day –7 to –4)	Baseline[a] Day 1	Week 2[b]	Weeks 4, 8, 12, 16, 20, 32, 40[b,s]	Weeks 24 and 48 or Early Termination[b, c]	Observational Follow-Up (Every 6 months)
Informed Consent and Eligibility Check	X						X[t]
Medical History			X				
Physical Exam/Vital Signs			X			X	
Targeted Physical Exam/Vital Signs					X		
Body Weight/Height[r]			X	X	X	X	
Selection/confirmation of OBT Regimen		X					X
Adverse Events			X	X	X	X	
Concomitant Medications			X	X	X	X	
Chemistry, Hematology	X	X[q]	X	X	X	X	
Fasting Metabolic Assessment (total cholesterol, HDL/LDL, triglycerides, glucose, glycosylated hemoglobin)			X			X	
12-lead Electrocardiogram			X			X[u]	
Orthostatic BP Monitoring	X		X[d]	X		X	
PK Sampling[e]				X	X	X	
Urinalysis			X			X	
Hepatitis screen (B core Ab, sAg, sAb, C Ab)	X						
Hepatitis C Virus RNA[f]			X		X	X	
CD4/CD8	X		X	X	X	X	
Plasma HIV-1 RNA	X	X	X	X	X	X	
Pregnancy Test[g]	X		X		X	X	
Plasma/PBMC/Proviral DNA Storage[h]			X		X	X	
Viral Resistance (Phenotype, Genotype)[i]	X				X[j]	X[o]	

090177e1815d915c\Approved\Approved On: 24-Aug-2010 20:15

Procedures	Screening (Day – 42 to -28)	Randomization (Day –7 to –4)	Baseline[a] Day 1	Week 2[b]	Weeks 4, 8, 12, 16, 20, 32, 40[b,s]	Weeks 24 and 48 or Early Termination[b, c]	Observational Follow-Up (Every 6 months)
Co-receptor tropism (Phenotype, Genotype[k])	X		X		X[j, l]	X[o]	
Host Genotyping			X[p]				
Free T4, TSH			X			X	
Dispense Study Medication			X	X[m]	X	X[n]	X
Assess Dosing Compliance				X	X	X	
Long term survival and selected endpoint assessment (LTS/SE)							X

a. Day 1, prior to dosing.
b. All visits must occur within ±4 days.
c. Subjects who discontinue study drug due to treatment failure or for other reasons must be followed per protocol until Week 48.
d. Patients with asymptomatic postural hypotension at the Baseline visit will be monitored for 4 hours following the first dose of study drug.
e. Two 5 ml PK samples are required at Weeks 2 and 24 and must be at least 30 minutes apart. One 5 ml PK sample required at other visits. Through Week 24 only.
f. If Hepatitis C antibody is positive at Screening Visit, to be performed at Baseline, Weeks 12, 24 and 48 or Early Termination.
g. For Women of Child Bearing Potential. Serum pregnancy at Screening and Urine Tests at the following visits. A positive Urine test must be confirmed with a serum test.
h. Plasma aliquots (2 of 1 mL each) at all timepoints. PBMCs, and proviral DNA will be stored at Baseline and at Weeks 24 and 48 or upon treatment failure only.
i. Reverse transcriptase, protease and fusion inhibitor resistance testing at screening to determine background regimen and at Week 24/48 if viral load >500 copies/mL or upon treatment failure.
j. Upon treatment failure as defined in the protocol (this sample is to be drawn when the confirmatory plasma HIV-1 RNA sample is collected).
k. Genotype (V3 loop alone or as part of gp 160 sequencing) at Baseline, Weeks 24 and 48 and at treatment failure only.
l. Weeks 4, 8, 16, 32 and 40 only.
m. Container from previous visit.
n. At Week 48 or Early Termination, medication will be dispensed to subjects who have completed 48 weeks of therapy and for whom it is medically appropriate to continue or begin therapy with UK-427,857. (See Section 5.6).
o. Except at Early Termination if a treatment failure (sample should be drawn when confirmatory HIV-1 RNA is collected).
p. Unless prohibited by local regulations.
q. Chemistry only.
r. Height recorded once. Weight through Week 24 only.
s. Also applicable for visits every 12 weeks post-Week 48 (ie. Week 60, 72, 84, 96, 108, 120, etc.) until the last enrolled subject has reached the Week 96 visit (with the exception of PK sampling and ECGs)
t. Informed consent for the observational phase will be sought prior to enrolment from subjects who have permanently discontinued or were lost to follow-up and from subjects who are still enrolled in the study whether in open-label or in the "in-study off drug".
u. ECGs are not required at scheduled or early termination visits post Week 48. Any ECGs done after week 48 will be done at the clinical discretion of the investigator and will not be sent to eRT.

TABLE OF CONTENTS

1. INTRODUCTION....11
1.1. Background....11
1.1.1. Data From Healthy Volunteer Studies....11
1.1.2. Data From Patient Studies....13
1.2. Rationale....14
1.2.1. UK-427,857 In Treatment-Experienced Patients:....14
1.2.2. Dose Selection:....14
2. TRIAL OBJECTIVES....16
2.1. Primary Objective....16
2.2. Secondary Objectives....16
3. TRIAL DESIGN....17
4. SUBJECT SELECTION....20
4.1. Inclusion Criteria....20
4.2. Exclusion Criteria....21
4.3. Life Style Guidelines....23
5. TRIAL TREATMENTS....23
5.1. Allocation to Treatment....23
5.2. Breaking the Blind....24
5.3. Drug Supplies....24
5.3.1. Formulation and Packaging....25
5.3.2. Preparation and Dispensing....25
5.3.3. Administration....25
5.3.4. Compliance....25
5.4. Drug Storage and Drug Accountability....25
5.5. Concomitant Medication(s)....26
5.6. Rescue Therapy....27
5.6.1. Subject Eligibility for Open Label Maraviroc After Unblinding of Subjects at Week 48....28
6. TRIAL PROCEDURES....29
6.1. Screening Visit (Day –42 to Day -28)....30

090177e1815d915c\Approved\Approved On: 24-Aug-2010 20:15

6.2. Trial Period 30
6.2.1. Randomization Visit (Day -7 to Day -4) 30
6.2.2. Baseline (Day 1) Evaluation – Prior to first dose 30
6.2.3. Week 2 Visit 31
6.2.4. On Study Evaluations (Week 4, 8, 12, 16, 20, 32 and 40). Post Week 48, every 12 weeks thereafter: Week 60, 72, 84, 96, etc. 32
6.2.5. Week 24 and 48 or Early Termination Visit 33
6.2.5.1. Subjects Who Previously Discontinued 35
6.3. Population Pharmacokinetics 35
6.3.1. Population Pharmacokinetics: Dosing History 35
6.3.2. Population Pharmacokinetics: Meal Times History 36
6.4. Drug Assay 36
6.5. Virology Testing 36
6.5.1. Viral Load Measurements 36
6.5.2. Virus Tropism 36
6.5.3. Viral Resistance 37
6.5.4. Virus Susceptibility to UK-427,857 37
6.5.5. Blood Pressure, Heart Rate and ECG Monitoring 38
6.5.6. Host Genotyping 38
6.6. Subject Withdrawal 39
7. ASSESSMENTS 40
7.1. Efficacy Assessments 40
7.2. Safety Assessments 41
8. ADVERSE EVENT REPORTING 41
8.1. Adverse Events 41
8.2. Definition of an Adverse Event 42
8.3. Abnormal Laboratory Findings 43
8.3.1. Laboratory Tests will include: 43
8.3.2. Follow Up of Laboratory Test Abnormalities 44
8.4. Serious Adverse Events 45
8.5. Hospitalization 45
8.6. Severity Assessment 46

090177e1815d915c\Approved\Approved On: 24-Aug-2010 20:15

8.7. Exposure In Utero....47
8.8. Discontinuations (See Also Subject Withdrawal, Section 6.6)....48
8.9. Eliciting Adverse Event Information....48
8.10. Reporting Requirements (Serious and Nonserious)....48
8.11. Additional Safety Information....50
9. DATA ANALYSIS/STATISTICAL METHODS....50
9.1. Sample Size Determination....50
9.2. Efficacy Analysis....50
9.2.1. Analysis Populations....50
9.2.2. Analysis of Primary Endpoint....51
9.2.2.1. Primary Endpoint....51
9.2.2.2. Method of Analysis....51
9.2.3. Analysis of Secondary Endpoints....52
9.2.3.1. Secondary Endpoints....52
9.2.3.2. Method of Analysis....53
9.3. Analysis of Other Endpoints....55
9.3.1. Pharmacokinetic Sampling and Population PK....55
9.4. Safety Analysis....55
9.5. Interim Analysis....55
9.6. Data Monitoring Committee....56
10. QUALITY CONTROL AND QUALITY ASSURANCE....56
11. DATA HANDLING AND RECORD KEEPING....56
11.1. Case Report Forms/Electronic Data Record....56
11.2. Record Retention....57
12. ETHICS....57
12.1. Institutional Review Board (IRB)/Independent Ethics Committee (IEC)....57
12.2. Ethical Conduct of the Trial....58
12.3. Subject Information and Consent....58
13. SPONSOR DISCONTINUATION CRITERIA....58
14. PUBLICATION OF STUDY RESULTS....58
15. REFERENCES....59

090177e1815d915c\Approved\Approved On: 24-Aug-2010 20:15

APPENDICES

Appendix 1. AIDS-DEFINING OPPORTUNISTIC ILLNESSES (OIS) 60

Appendix 2. Liver Enzyme Work-up...62

Appendix 3. COLLECTION OF CEREBROSPINAL FLUID 63

Appendix 4. Actg Grading Severity Of Adult Adverse Events 64

Appendix 5. Information Pertaining to the Extension Phase of Protocol A4001027 70

Appendix 6. Clinical Protocol Amendment #1..73

Appendix 7. Clinical Protocol Amendment #2..91

Appendix 8. Clinical Protocol Amendment #3..112

Appendix 9. Clinical Protocol Amendment #4..115

Appendix 10. Clinical Protocol Amendment 5..135

Appendix 11. Clinical Protocol Amendment 6..141

090177e1815d915c\Approved\Approved On: 24-Aug-2010 20:15

1. INTRODUCTION

Over 40 million people worldwide are now infected with HIV with an estimated 5 million new infections in 2003.[1] At present the only means of slowing disease progression is to use a multi-drug combination strategy working against three HIV targets, protease, reverse transcriptase and gp-41. These agents reduce the rate of virus replication, while seeking to prevent the rapid emergence of resistant virus. While several of these combinations are successful, the side-effect profiles can reduce patient compliance. In addition, current drug combination therapies are unable to overcome the emergence of multi-drug resistant viruses. Thus there is a high medical need for better tolerated, conveniently administered agents to prevent the development and progression of AIDS in HIV infected individuals, reduce susceptibility to secondary infections, and return the patient to a normal lifestyle. Furthermore, in order to delay the development of resistance, the development of new classes of drugs, which work on different stages of the life cycle of the virus, is necessary.

An essential step in the replication cycle of HIV is attachment to both the CD4 receptor and one of the chemokine receptors (CCR5 in the case of primary infection and earlier stages of disease). Blocking of the CCR5 receptor prevents viral entry into the host cell and will therefore lead to a drop in viral load. This has been demonstrated in initial Phase 2a studies, with the CCR5 antagonists SCH-C and UK-427,857, and more recently with SCH-D. All compounds significantly reduced the viral load in HIV infected patients receiving monotherapy over 10-14 days.[2, 3, 4] These results confirmed genetic evidence from previous in vitro studies that indicated that blocking of the CCR5 receptor has a profound inhibitory effect on viral infection.

CCR5-tropic HIV-1 is known to predominate during early HIV-infection with the risk of harboring CXCR4-tropic virus (either with R5 virus or as the predominant viral species) increasing significantly over time. A recent analysis of the enfuvirtide TORO trials in patients who were heavily treatment-experienced and/or multi-class resistant, however, demonstrated that 62% of patients in the combined trials remained CCR5-tropic.[5] Thus, CCR5 inhibition may provide additional benefit for a sizable portion of treatment-experienced patients, including as part of salvage therapy.

1.1. Background

UK-427,857 is a small molecule antagonist of the CCR5 receptor with potent antiviral activity in vitro (geometric mean antiviral IC_{90} against a laboratory-adapted strain [BaL] of 5.57 nM, 95% CI: 3.98 -7.78 nM [2.85 ng/ml, 95% CI: 2.04-4.0 ng/ml]; and against a range of primary HIV-1 isolates [n=44] of 2.03 nM, 95% CI: 1.75-2.36 nM [1.04, 95% CI: 0.9-1.22 ng/ml]).

Further details of pre-clinical and toxicological data may be found in the Investigator's Brochure.

1.1.1. Data From Healthy Volunteer Studies

To date a total of 501 healthy volunteers have received at least one dose of UK-427,857. Single oral doses up to 1200 mg and multiple oral doses up to 300 mg BID for 28 days and up to 900 mg BID and 1200 mg QD for 7 days were well tolerated.

090177e1815d915c\Approved\Approved On: 24-Aug-2010 20:15

The most common all-causality Adverse Events (AEs) reported following single and multiple UK-427,857 dosing have been asthenia, headache, dizziness, nausea, rhinitis and postural hypotension. Postural hypotension is potentially the dose limiting adverse event. The adverse event profile seen in the single and multiple dose populations were similar and adverse events did not happen significantly more on UK-427,857 than on placebo at doses up to and including 300 mg BID. No Serious Adverse Events (SAEs) were reported in the Phase 1 program and most AEs were judged to be mild to moderate in severity

In the single dose study (A4001001) four out of nine subjects who were administered a single 1200 mg dose of UK-427,857 experienced postural hypotension. Clinically significant postural hypotension caused dosing to be halted for Cohort 3 of the multiple dose study (A4001002) with 2/9 subjects on 600 mg QD and 1/3 on placebo. Dosing was repeated at 600 mg QD in an additional group of subjects (Cohort 5) and was well tolerated, with one subject experiencing mild postural hypotension. Hence the maximum tolerated multiple dose remains undefined. Cross-protocol analysis of adverse event data from the Phase 1 studies has indicated a dose relationship for episodes of postural hypotension and dizziness. A logistic regression analysis demonstrated that the incidence of postural hypotension increased above the placebo incidence at unit doses of above 600 mg. Single and repeated dosing with UK-427,857 had no apparent effects on supine systolic or diastolic blood pressure, although there was evidence of a treatment-related increase in supine pulse rate after a single 1200 mg dose (mean increase from baseline of 9 bpm at 1 hour post- dose). There were dose-related reductions in standing systolic blood pressure at doses of 300 mg BID and above and for standing diastolic blood pressure at 600 mg QD.

Analysis of the single dose, adequately powered QT study with active control (A4001016) has shown that the mean difference in QTcI from placebo for all primary endpoints was less than 4 msec for all doses of UK-427,857 (100 mg, 300 mg and 900 mg). Mean differences from placebo in QTcI for UK-427,857 were not clinically significant for single doses up to and including 900 mg.

UK-427,857 absorption is rapid but variable after oral dosing and C_{max} typically occurs 0.5–4h after dosing. Assessment of UK-427,857 over the dose range 50 to 600 mg confirmed that the pharmacokinetics of UK-427,857 are not dose proportional. It is estimated that a doubling in dose will lead to a 2.3-fold increase (95% CI; 2.2, 2.4-fold) in mean AUC over this dose range. UK-427,857 has biphasic elimination with a measured terminal half-life of 9 to 14 hours following single doses.

UK-427,857 is a CYP3A4 substrate. Hence its concentrations are increased by potent CYP3A4 inhibitors (such as ketoconazole, saquinavir and ritonavir) and decreased by CYP3A4 inducers (such as rifampin and efavirenz). UK-427,857 dose adjustments are recommended if it is to be co-administered with such agents. Specific guidelines on doses are given in Section 5.5 of the protocol.

090177e1815d915c\Approved\Approved On: 24-Aug-2010 20:15

From pre-clinical data, UK-427,857 is not predicted to affect the pharmacokinetics of other drugs that are metabolized by any of the major P450 enzymes. Clinical studies have demonstrated that UK-427,857 does not lead to clinically significant changes in plasma concentrations of oral contraceptives (ethinyloestradiol and levonorgestrel) or CYP3A4 substrates such as midazolam.

1.1.2. Data From Patient Studies

In two short-term monotherapy studies, A4001007 and A4001015, 79 asymptomatic CCR5 tropic HIV-1 infected patients with CD4 count >250 cells/mm^3 and plasma viral load >5000 copies/ml received UK-427,857 (in total daily doses ranging from 25 mg to 600 mg) or placebo for 10 days. All doses were given in the fasted state or under food restrictions, apart from 150 mg BID, where both a fed and fasted dose were given. Mean viral load reductions of 0.43, 1.13, and 1.35 log_{10} were seen for doses of 25, 100 and 300 mg QD, respectively. BID doses of 50, 100, 150 and 300 mg resulted in viral load reductions of 0.66, 1.42, 1.45, and 1.6 log_{10}, respectively. Patients receiving 150 mg BID fed had a viral load decline of 1.34 log_{10}, indicated a negligible impact of food on viral load reduction. UK-427,857 was safe and well-tolerated in this patient population. The AE profile was similar to that seen for healthy volunteers.

There was a change in virus tropism in two individuals treated with UK-427,857, both in study A4001015. One subject received UK-427,857 100 mg QD and had a viral load decline of 0.710 log_{10} from baseline. Analysis of virus tropism on Day 11 demonstrated the presence of virus with the ability to enter cells expressing the CXCR4 receptor. By Day 40 the virus population had reverted to CCR5 only usage. Screening of multiple clones obtained from the virus population at the different time points revealed the presence of a small number of clones (2 out of 97 viable clones) with the ability to enter cells expressing CXCR4 at baseline. By Day 11, 40 of 68 clones could enter CXCR4 expressing cells, while only 1 of 91 could enter into CXCR4 expressing cells by Day 40. These data are consistent with the presence of a small population (<5%) with the ability to enter into cells expressing the CXCR4 receptor at baseline with a relative increase on Day 11, reverting back to baseline following withdrawal of UK-427,857.

The second subject received UK-427,857 100 mg QD and had a viral load decrease of 1.256 log_{10} from baseline. In this subject R5X4 virus emerged by Day 11 and has been detectable at all time points since. A detailed clonal analysis of the virus population at the different time points was performed. Of 118 viable clones evaluated at baseline, none had the ability to use CXCR4 for entry. At Day 11, 21 of 52 clones could use the CXCR4 receptor for entry, while 17 of 63 could use CXCR4 at Day 40. To evaluate the possibility that the R5X4 tropic virus present at Days 11 and 40 could have arisen from the CCR5 population present at baseline, gp160 sequencing and phylogenetic analyses of 12 randomly selected clones from each time point were performed. Analysis of this data revealed that the R5X4 viruses present at Days 11 and 40 were closely related, but genetically distinct from the R5 population. Direct sequence comparisons indicated that there were 104 nucleotide differences between a representative clone from the R5X4 population and the most closely related R5 virus. At the amino acid level there were 55 differences, including conserved amino acids, using the same

090177e1815d915c\Approved\Approved On: 24-Aug-2010 20:15

comparison. It is, therefore, very unlikely that the R5X4 viruses could have originated from the R5 population during the 10 day period of dosing with the CCR5 antagonist. It is more likely, therefore, that the R5X4 virus has been present prior to dosing with UK-427,857, either at a very low prevalence (<1%) in the peripheral circulation, or as archived virus in a tissue compartment. The large CD4 T-cell increase (173 cells/mm^3 over 10 days) in this patient could indicate redistribution of cells from central compartments to the periphery and may have resulted in archived virus entering the circulation.

Further follow-up samples taken from this patient at days 203, 251, 308, 373 and 433 demonstrated that the R5X4 virus was still present in the peripheral circulation, with emergence of X4 variants over the later sampling points. Over the >1 year period since the patient received UK-427,857, his viral load has been variable but remains within the range of his pre-study values (viral load at randomization visit was 80471 copies/mL). However, there has been a gradual decline in his CD4 count to a value of 219 cells/mm^3 on Day 433. This is a significant decline from his immediate pre-study CD4 count and is also significantly lower than his lowest count (421 copies/mm^3) recorded over the past >5 years of follow-up. The precise role of R5 and X4 viruses in HIV pathogenesis is not known, but there is an hypothesis that the X4 species is responsible for faster progression to late-stage AIDS. Therefore, in light of the evolving data, the possibility that UK-427,857 selected the X4 species over R5 and that this may be causally related to the decline in CD4 cell counts cannot be excluded. The patient remained clinically well and eventually started HAART on Day 433.

Further information can be obtained from the current version of the Investigator's Brochure.

1.2. Rationale

1.2.1. UK-427,857 In Treatment-Experienced Patients:

There is a desperate need for new drugs with novel mechanisms of action to be developed for treatment-experienced patients who have few or no remaining options due to multi-class resistance, toxicity to existing classes or both. UK-427,857, a selective and reversible CCR5 co-receptor antagonist, has been shown to be active in vitro against a wide range of clinical isolates (including those resistant to existing classes). In HIV-1 infected patients, UK-427,857 given as monotherapy for 10 days reduced HIV-1 viral load by up to 1.6 $\log_{10}$, consistent with currently available agents comprising the cornerstone of HAART (eg, protease inhibitors, non-nucleoside reverse transcriptase inhibitors). An excellent safety and toleration profile has been demonstrated with dosing for up to 28 days at 300 mg BID, including no significant effect on QTc interval. Since most patients who are heavily antiretroviral experienced remain infected with CCR5-tropic virus only, studies with UK-427,857 in this patient population are warranted.

1.2.2. Dose Selection:

The rationale for dose selection is to maximize the benefit: risk ratio for UK-427,857 in treatment experienced patients. The dose selection is based on 10 day monotherapy data, PK/PD modeling, clinical trial simulations, pharmacokinetics, drug-drug interaction studies, preclinical serial passage resistance studies and a safety database of over 400 subjects followed for up to 4 weeks.

090177e1815d915c\Approved\Approved On: 24-Aug-2010 20:15

Consideration of the integrated safety and toleration data generated in the Phase 1 healthy volunteer studies and Phase 2a monotherapy studies has enabled the optimal balance between the efficacy data discussed above and the potential safety and toleration burden. The major factor identified thus far that determines the maximum tolerated/safe clinical dose is postural hypotension. However, no safety data are available for long-term combination therapy in HIV infected patients at this stage and therefore the assumption is that short-term monotherapy data are predictive of that situation.

As previously noted, 2 studies (A4001007 and A4001015), have been performed in treatment naïve patients, investigating the anti-viral effects, pharmacokinetics, safety and toleration of various doses and regimens of UK-427,857. The conclusions from these studies are that anti-viral effect appears to be related to daily dose/exposure, such that similar anti-viral effects are seen for total daily doses of above 200 mg, administered as a once or twice a day regimen. In addition the anti-viral effects of a 150 mg BID dose do not appear to be affected by co-administration with food. Maximal anti-viral effects appear to occur at doses of 100 mg BID (200 mg/day) and above. The anti-viral effects of these doses are in line with those seen with short term monotherapy for other anti-retroviral agents, which have subsequently shown robust long term anti-viral effects in combination with other anti-retroviral agents in treatment experienced patients.

The anti-viral data from these short term monotherapy studies have been incorporated into a fully integrated drug-disease model which has subsequently been used to simulate the outcome of Phase 2b/3 trials in treatment naïve patients receiving zidovudine/lamivudine in combination with UK-427,857. Given that there is no long-term combination data available for any CCR5 inhibitor, a number of assumptions around resistance, compliance, adherence and baseline viral load have been made. Together, the monotherapy and clinical trial simulation (CTS) data have enabled rationale selection of doses to be taken into the Phase 2b/3 studies based on anti-viral efficacy.

A model was used to simulate the UK-427,857 (plus zidovudine/lamivudine) dose response curve for the success rate measured as the proportion of patients with <400 copies/mL at Week 24 and Week 48, assuming no UK-427,857 resistance. The proportion of patients with <400 HIV-1 RNA copies/mL increased with increasing dose. The results from the simulations suggested that a 71% success rate at 48 weeks in an intention-to-treat population is predicted for 300 mg BID in treatment naïve patients with a baseline viral load of 4.8 $\log_{10}$. For 300 mg QD, a 62% success rate is predicted at 48 weeks, however a much higher success rate is predicted at Week 24 (approximately 77%). The failure rate post 24 weeks is primarily driven by predicted emergence of resistance to zidovudine/lamivudine rather than to UK-427,857.

Limited clinical trial simulations for the treatment-experienced program have been conducted due to the difficulty in modeling variable background regimens. Based on the magnitude of the monotherapy response we predict however, by analogy to other effective agents, doses that lead to more than a 1 $\log_{10}$ drop should demonstrate at least a 0.5 $\log_{10}$ drop in a treatment experienced patient on optimized background therapy.

090177e1815d915c\Approved\Approved On: 24-Aug-2010 20:15

Taken together, the efficacy data and clinical trial modeling, demonstrate that doses of 200 mg to 600 mg daily have similar effects on viral load. Efficacy appears to be driven by daily dose and is independent of the regimen by which it is administered at daily doses of above 100 mg. A daily dose of 300 mg BID (600 mg daily) is likely to be towards the top of the dose response curve, while a daily dose of 150 mg BID (300 mg daily) is also likely to provide a good response. These doses produced a short-term decline in HIV-1 RNA comparable to other agents, which are considered to be acceptable “anchor drugs” in a typical HAART regimen. Additionally, since it has been difficult to demonstrate a dose-response based on efficacy in Phase 2b studies with other antiretroviral agents, a 50% reduction in the total daily dose is likely to result in the best opportunity to achieve this. It is also clear that the dose limiting adverse event (postural hypotension) is related to plasma levels of UK-427,857, with the incidence being the greatest around the C_{max} of the doses at which it occurs. Given the high medical need to simplify dose regimens and enhance adherence, as well as the potency of UK-427,857 when given as a 300 mg once-daily dose compared with other agents (eg, atazanavir), we propose to study doses equivalent to 300 mg QD and 300 mg BID when adjusting for the components of background therapy. These doses will maximize the antiviral effects while minimizing the chances of postural hypotension and other potential safety and toleration issues.

Finally, the drug interaction profile of UK-427,857 has been investigated showing that its systemic exposure is significantly increased in the presence of CYP3A4 inhibitors. These data have lead to the proposed dose adjustments in treatment-experienced patients receiving protease inhibitors as part of their optimized background therapy. The assumption is that most OBT regimens will contain at least one PI. Based on the results of drug interaction studies, doses of 150 mg QD and 150 mg BID are proposed for this study to compensate for the likely presence of a metabolic inhibitor. Patients whose OBT does not contain a PI will receive doses of 300 mg QD and 300 mg BID.

2. TRIAL OBJECTIVES

2.1. Primary Objective

To assess whether UK-427,857 added to Optimized Background Therapy (OBT) provides an additional reduction in plasma HIV-1 RNA compared to OBT alone, as measured by the difference between each of the two UK-427,857 regimens versus the placebo regimen in the mean changes from baseline in plasma HIV-1 RNA at Week 48. This variable will also be analyzed at Week 24.

2.2. Secondary Objectives

1. To compare the percentage of subjects with HIV-1 RNA less than 400 copies per mL at Weeks 24 and 48 for each of the two UK-427,857 regimens versus the placebo regimen.
2. To compare the percentage of subjects with HIV-1 RNA less than 50 copies per mL at Weeks 24 and 48 for each of the two UK-427,857 regimens versus the placebo regimen.

090177e1815d915c\Approved\Approved On: 24-Aug-2010 20:15

3. To compare the percentage of subjects who achieve at least a 0.5 $\log_{10}$ reduction in HIV-1 RNA from baseline at Weeks 24 and 48 for each of the two UK-427,857 regimens versus the placebo regimen.
4. To compare the percentage of subjects who achieve at least a 1 $\log_{10}$ reduction in HIV-1 RNA from baseline at Weeks 24 and 48 for each of the two UK-427,857 regimens versus the placebo regimen.
5. To compare the time to loss of virological response through Week 48 for each of the two UK-427,857 regimens versus the placebo regimen.
6. To compare the differences in the magnitude of changes in CD4 cell counts from baseline through Weeks 24 and 48 for each of the two UK-427,857 regimens versus the placebo regimen.
7. To compare the differences in the magnitude of changes in CD8 cell counts from baseline through Weeks 24 and 48 for each of the two UK-427,857 regimens versus the placebo regimen.
8. To compare the Time-Averaged Difference (TAD) in $\log_{10}$ HIV-1 RNA at Weeks 24 and 48 for each of the two UK-427,857 regimens versus the placebo regimen.
9. To assess HIV-1 genotype and phenotype at baseline and at the time of failure (patients with HIV-1 RNA greater than 400 copies/mL at any visit after Week 4, or other reasons for treatment failure listed at end of Section 3).
10. To assess HIV-1 tropism at baseline and at the time of failure (patients with HIV-1 RNA greater than 400 copies/mL at any visit after Week 4, or other reasons for treatment failure listed at end of Section 3).
11. To assess the association between baseline resistance and virological response.
12. To compare the safety and tolerability of each of the two UK-427,857 regimens versus the placebo regimen.

3. TRIAL DESIGN

This is a 48-week, 2:2:1 randomized, double-blind, superiority study designed to compare the safety and antiviral activity of UK-427,857 at two different doses versus placebo, each in combination with optimized background therapy. This study is planned to enroll 600 subjects. Subjects must be infected with CCR5-tropic HIV-1 and have had at least 6 months of prior treatment with at least one agent (two for protease inhibitors) from 3 of the 4 antiretroviral drug classes or multi-class resistance and treatment failure to an existing regimen, defined by a plasma HIV-1 RNA ≥5,000 copies/mL. All subjects will undergo genotypic and phenotypic testing for the presence of CCR5-tropic HIV-1 and for resistance to reverse transcriptase inhibitors, protease inhibitors and fusion inhibitors. Screening genotype and phenotype results will be available approximately 3 to 5 weeks after receipt of samples. Subjects must be on a stable regimen for at least 4 weeks prior to randomization and therefore will be required to remain on their existing regimen during the screening period. Subjects not on any antiretroviral therapy for 4 weeks ending prior to the baseline visit should remain off therapy until the baseline visit.

090177e1815d915c\Approved\Approved On: 24-Aug-2010 20:15

Investigators will optimize therapy, with 3-6 (excluding low-dose ritonavir) branded, open-label agents, on the basis of these resistance tests, treatment history and safety/adverse event considerations. No more than one non-nucleoside reverse transcriptase inhibitor may be included in the background regimen. Changes to background therapy may only be made after the baseline visit under the following circumstances and after consultation with the medical monitor: 1) toxicity attributed to one or more background agents - a drug of the same class may be substituted; 2) documented human error in interpretation of Screening resistance test results – during the 2 week period following the baseline visit but before the Week 2 visit, one agent may be substituted or added to the background regimen without requiring the patient to be discontinued from study drug; 3) dose adjustments to existing background agents due to reason 1) above or to the addition of a required concomitant agent that results in an anticipated new drug interaction. Experimental antiretroviral agents available through pre-approval access programs or by other means may be appropriate for use as part of background therapy following approval by the medical monitor on an individualized basis. Subjects taking efavirenz as part of their OBT should consider the addition of a PI as part of their OBT. Mean reduction in HIV-1 RNA will be assessed at 24 and 48 weeks or at the time of discontinuation from the study. All subjects who receive at least one dose of study medication will be assessed for safety. The study will enroll over approximately a 9-month period with 48 weeks of treatment. As part of this clinical study a blood sample will be taken for non-anonymized pharmacogenetic analysis, unless prohibited by local regulations.

A Data Safety Monitoring Board (DSMB) will review the results following treatment of 100 patients for at least 8 weeks. No formal statistical tests will be performed. If the DSMB feels that either of the doses of UK-427,857 show substantial evidence of harm to patients, then the DSMB will recommend ending recruitment to this treatment arm. In addition, The Sponsor or its designee will conduct regular blinded reviews of serious adverse events (weekly) and adverse clinical and laboratory events (monthly) with particular focus on new infections, liver enzyme abnormalities and postural hypotension.

This study will evaluate two doses of UK-427,857 (150 mg once daily and 150 mg twice daily) when taken in combination with optimized background therapy. Subjects randomized to the UK-427,857 150 mg QD arm will receive UK-427,857 placebo drug in the morning and UK-427,857 active drug in the evening. Subjects randomized to the UK-427,857 150 mg BID arm will receive UK-427,857 active drug in the morning and in the evening. Subjects randomized to the UK-427,857 placebo arm will receive UK-427,857 placebo drug in the morning and in the evening. As UK-427,857 is a substrate for CYP3A4 inhibition, a nominal unit dose of 150 mg will be studied assuming that most patients will be receiving at least one PI in their background treatment regimen. Patients on regimens that do not include a PI will receive a UK-427,857 unit dose of 300 mg. UK-427,857 may be taken with or without food. Antiretroviral agents comprising the background regimen should be taken according to the manufacturer's product labeling. Randomization will be stratified by screening HIV-1 RNA levels (<100,000 versus ≥100,000 copies/mL) and by inclusion of enfuvirtide in the background regimen.

090177e1815d915c\Approved\Approved On: 24-Aug-2010 20:15

Subjects will be randomly assigned to one of the following treatment groups:

- Optimized Background Therapy (3-6 drugs based on treatment history and resistance testing) + UK-427,857 150 mg PO taken once daily;
- Optimized Background Therapy (3-6 drugs based on treatment history and resistance testing) + UK-427,857 150 mg PO taken twice daily;
- Optimized Background Therapy (3-6 drugs based on treatment history and resistance testing) + UK-427,857 placebo.

Subjects will remain on their assigned therapy for 48 weeks, unless the subject is discontinued early for protocol-defined treatment failure or other reasons such as adverse event, loss to follow-up, withdrawal of consent or death. These subjects will be required to remain in the studies on their new regimen and be followed according to the protocol. Subjects experiencing toxicity, but not failure, attributed to drugs in the background regimen would be able to substitute a drug of the same class during the trial in consultation with the medical monitor. The primary efficacy analysis will be conducted following 24 weeks of therapy. Co-receptor tropism testing will be performed in all patients with HIV-1 RNA greater than 500 copies/mL at Weeks 4, 8, and then every 8 weeks and in all patients who meet any of the criteria for treatment failure.

Treatment failure is defined as meeting any one of the following virological endpoints:

- An increase to at least 3 times the baseline (mean of all 3 values before start of dosing) plasma HIV-1 RNA level at the Week 2 visit or thereafter (confirmed by a second measurement taken no more than 14 days after the first measurement);
- HIV-1 RNA <0.5 $\log_{10}$ decrease from baseline (mean of all 3 values before start of dosing) on two consecutive measurements starting at Week 8 (second measurement taken no more than 14 days after the first measurement);
- HIV-1 RNA <1.0 $\log_{10}$ decrease from baseline (mean of all 3 values before start of dosing) on two consecutive measurements starting at Week 8 (second measurement taken no more than 14 days after the first measurement), in a patient who had previously achieved a ≥2.0 $\log_{10}$ decrease from baseline; or
- An increase in HIV-1 RNA to ≥5,000 copies/mL on two consecutive measurements taken no more than 14 days apart, in subjects previously confirmed to have undetectable levels of <400 copies/mL on 2 consecutive visits.

090177e1815d915c\Approved\Approved On: 24-Aug-2010 20:15

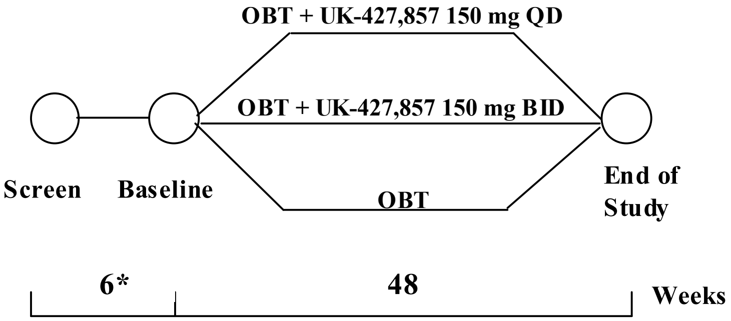

* Subjects requiring enfuvirtide may require more time to obtain medication

4. SUBJECT SELECTION

The target population for this study is HIV-1 infected patients over the age of 16 who have previous antiretroviral experience with ≥1 agent (≥2 for protease inhibitors) from 3 of the 4 antiretroviral drug classes for ≥6 months or documented resistance to members of 3 of 4 classes, a stable antiretroviral regimen for at least 4 weeks prior to randomization, HIV-1 RNA ≥5,000 copies/mL, CCR5-tropic HIV-1 and no evidence of infection with CXCR4- or dual-tropic HIV-1.

This clinical trial can fulfill its objectives only if appropriate subjects are enrolled. The following eligibility criteria are designed to select subjects for whom protocol treatment is considered appropriate. All relevant medical and non-medical conditions should be taken into consideration when deciding whether this protocol is suitable for a particular subject.

4.1. Inclusion Criteria

Subjects must meet all of the following inclusion criteria to be eligible for enrollment into the trial:

1. Provide a signed and dated written informed consent document indicating that the subject (or a legally acceptable representative) has been informed of all pertinent aspects of the trial;
2. Men or women at least 16 years of age (or minimum age as determined by local regulatory authorities or as legal requirements dictate) available for a follow-up period of at least 48 weeks;
3. HIV-1 RNA viral load of ≥5,000 copies/mL measured by Roche Amplicor HIV-1 Monitor (version 1.5) at the screening visit;

090177e1815d915c\Approved\Approved On: 24-Aug-2010 20:15

4. Stable pre-study antiretroviral regimen, or on no antiretroviral agents, for at least 4 weeks;
5. Documented genotypic (http://iasusa.org/resistance_mutations/index.html as determined by the Monogram Biosciences (formerly Virologic) GeneSeq HIV drug resistance assay) or phenotypic (fold change of patient's virus ≥ cutoff value based on Monogram Biosciences PhenoSense HIV drug resistance assay) resistance to three of the four antiretroviral drug classes [Nucleoside or nucleotide reverse transcriptase inhibitors (nRTIs), Non-nucleoside reverse transcriptase inhibitors (NNRTIs), Protease Inhibitors (PIs) or Entry Inhibitors (EIs)].

   OR

   Antiretroviral-class experience ≥6 months (sequential or cumulative) with at least three of the following:

   a. One nucleoside or nucleotide reverse transcriptase inhibitor;
   b. One non-nucleoside reverse transcriptase inhibitor;
   c. Two protease inhibitors (excluding low-dose ritonavir);
   d. Enfuvirtide.
6. Be willing to initiate and remain on randomized treatment without any changes or additions to the OBT regimen, except for toxicity management or upon meeting criteria for treatment failure.
7. A negative urine pregnancy test at the baseline visit, prior to receiving the first dose of study medication, for Women of Child Bearing Potential (WOCBP).

   NOTE: WOCBP include any female who has experienced menarche and who has not undergone successful surgical sterilization or is not post-menopausal (ie, no menstrual periods for at least 2 years). Even women who are using oral, implanted or injectable contraceptive hormones or mechanical products (intrauterine devices; barrier methods: eg, condom or diaphragm with spermicide) to prevent pregnancy, who are practicing abstinence, or who have a partner that is sterile (eg, vasectomy), should be considered to be of child bearing potential.
8. Effective barrier contraception for WOCBP and males. In addition, WOCBP must use another acceptable method of contraception for the duration of the study. Acceptable contraception includes, but is not limited to, oral, implanted or injectable hormone therapy and intrauterine devices.

4.2. Exclusion Criteria

Subjects presenting with any of the following will not be included in the trial:

1. Patients requiring treatment with more than 6 antiretroviral agents (excluding low-dose ritonavir);

090177e1815d915c\Approved\Approved On: 24-Aug-2010 20:15

2. Prior treatment with UK-427,857 or another experimental HIV entry inhibitor for more than 14 days;
3. Active alcohol or substance abuse sufficient, in the Investigator's judgment, to prevent adherence to study medication and/or follow-up;
4. Lactating women, or planned pregnancy during the trial period;
5. Suspected or documented active, untreated HIV-1 related opportunistic infection (OI) or other condition requiring acute therapy (eg, hepatitis C virus infection) at the time of randomization [patients on a stable (>1 month) secondary OI prophylaxis regimen or chronic treatment (eg, for hepatitis C virus infection) are eligible for the study; patients on a primary OI prophylaxis regimen of any duration are also eligible for the study];
6. Treatment for an active opportunistic infection, or unexplained temperature >38.5°C for 7 consecutive days, within 30 days prior to randomization;
7. Malignancy requiring parenteral chemotherapy that must be continued for the duration of the trial;
8. Initiating therapy with a potentially myelosuppressive, neurotoxic, hepatotoxic and/or cytotoxic agent within 60 days prior to randomization, or the expected need for such therapy during the study period. NOTE: Trimethoprim-sulfamethoxazole **may not** be initiated 60 days prior to randomization but may be continued if the subject is on stable therapy. The use of isoniazid is prohibited;
9. Documented or suspected acute hepatitis or pancreatitis within 30 days prior to randomization;
10. Renal insufficiency defined as a serum creatinine greater than 3 times the upper limit of normal;
11. Total bilirubin, greater than 2.5 times the upper limit of normal (unless unconjugated hyperbilirubinemia due to atazanavir or indinavir). Changes of one or more grades in total bilirubin between screening and the randomization visit should be reviewed with the Study medical monitor before the initiation of double-blind study medication;
12. AST or ALT greater than 5 times the upper limit of normal. Changes of one or more grades in AST or ALT between screening and the randomization visit should be reviewed with the Study medical monitor before the initiation of double-blind study medication;
13. Cirrhosis of the liver;
14. Absolute neutrophil count ≤750 cells/mm^3;
15. Platelet count ≤50,000 cells/mm^3;
16. Hemoglobin ≤7 g/dL;
17. Clinically significant malabsorption syndrome (eg, ≥6 loose stools per day for at least 7 consecutive days) within 30 days prior to randomization;
18. Inability to tolerate oral medication;

090177e1815d915c\Approved\Approved On: 24-Aug-2010 20:15

19. X4-or dual/mixed-tropic virus detected by the PhenoSense™ viral entry assay or repeated assay failure;
20. Concomitant therapy with other investigational agents (other than experimental antiretroviral agents available through pre-approval access programs);
21. Contraindicated medications (eg, immunomodulators for the treatment of HIV-1 infection; interferon for the ongoing treatment of hepatitis C infection is permitted) being taken by the subject at the time of randomization that must be continued during the study period (See Section 5.5);
22. Any safety, behavioral, clinical, or administrative reasons that, in the Investigator's judgment, would potentially compromise study compliance or the ability to evaluate safety/efficacy.

4.3. Life Style Guidelines

Women of childbearing potential [any female who has experienced menarche and who has not undergone successful surgical sterilization or is not post-menopausal (ie, no menstrual periods for at least 2 years)] must agree to avoid pregnancy during the study. WOCBP must use an acceptable method of contraception for the duration of the study and will be given advice on the correct use of contraceptives. Acceptable contraception includes, but is not limited to, oral, implanted (eg, Norplant) or injectable hormone therapy (eg, Depo Provera) and intrauterine devices. In addition both males and females must agree to use a barrier method of contraception (eg, condom or diaphragm with spermicide) for the duration of the study.

5. TRIAL TREATMENTS

5.1. Allocation to Treatment

Eligible subjects who have provided written informed consent will be randomized into one of the three treatment groups in a 2:2:1 ratio (UK-427,857 150 mg QD : UK-427,857 150 mg BID : placebo). Subjects will be stratified at the time of randomization by the use of enfuvirtide in the background regimen and by Screening HIV-1 RNA (<100,000 or ≥100,000 copies/mL). Subjects will be randomized according to a computer generated pseudo-random code using the method of permutated blocks, balanced within each randomization strata (screening HIV-1 RNA ≥100,000 and using enfuvirtide, screening HIV-1 RNA ≥100,000 and not using enfuvirtide, etc).

Randomization numbers will be assigned by a central web/telephone computer-based telerandomization system (IVRS). The randomization call must be made at the Randomization Visit to allow for medication to be shipped to the site. This system will dispense medication by assigning appropriate container numbers to subjects based on their assigned treatment groups. The appropriate dose will be assigned by the system based on the specific background medications selected by the Investigator at the Randomization Visit. Subject numbers will not appear on study medication containers, only container numbers will appear.

090177e1815d915c\Approved\Approved On: 24-Aug-2010 20:15

Only authorized site personnel will be granted access to the central randomization system. The following information will be required to randomize a subject:

- Site User ID (identification) and PIN (personal identification number), which are assigned to the investigational site by the randomization system;
- Protocol number;
- Subject's date of birth;
- Subject's gender.

The randomization system will generate a randomization number and a Single-Subject Identifier, which must be used on all documentation and correspondence. The Single-Subject Identifier will consist of the center number and a four-digit extension and will be assigned to all patients that sign a Patient Informed Consent document.

It is the responsibility of the Principal Investigator to ensure that the subject is eligible for the study before requesting randomization.

5.2. Breaking the Blind

Optimized background Therapy (OBT) will be selected by the Investigator and will be administered in open-label fashion. UK-427,857 will be administered in double-blind fashion such that placebo will be indistinguishable from the active study drug. The Investigator, study staff, subject and sponsor will be kept unaware of the treatment assignments until all outcome assessments (efficacy and safety) have been completed and the database has been locked. The sponsor will be unblinded for the 24-week analysis while investigator and patient will remain blinded until the completion of the trial.

At the initiation of the study, the study site will be instructed on the method for blind breaking. The method will be either a manual or electronic process. Blinding codes should only be broken in emergency situations for reasons of subject safety. The Investigator should contact Pfizer before breaking the blind. When the blinding code is broken, the reason must be fully documented and entered on the case report form.

In the event that an emergency break blind is required, authorized/approved randomization system users will have the ability to retrieve subject treatment groups through the randomization system.

5.3. Drug Supplies

- ViiV Healthcare or it designee will supply UK-427,857 and matching placebo tablets. For the observational portion of study, the Sponsor will supply open label UK-427, 857 for subjects for whom it is medically appropriate.
- Route of administration is oral.

090177e1815d915c\Approved\Approved On: 24-Aug-2010 20:15

- All drugs relating to the optimized background therapy (OBT) will be supplied via the Investigator site.

5.3.1. Formulation and Packaging

UK-427,857 tablets will be supplied as 150 mg dosage units. Matching placebo for UK-427,857 will also be supplied. For the observational portion of study, the Sponsor will also supply open label UK-427, 857 in 300 mg dosage units.

Drug supplies will be provided to the study sites as pre-packaged bottles. Subjects will receive two bottles per month of treatment, as defined by randomization. Subjects whose background regimen does not contain a protease inhibitor will receive double the number of tablets per bottle. During the observational phase of the study, subjects may receive one or two bottles per month of open label UK-427,857 depending on the optimized background therapy.

5.3.2. Preparation and Dispensing

The quantities of study drugs dispensed per visit will be defined by the date of the next study visit. For UK-427,857 and placebo tablets, drug supplies will be managed via a telerandomization system.

5.3.3. Administration

UK-427,857 may be taken with or without food. The patient should only take missed doses if it is not within 6 hours prior to the planned next dose. Antiretroviral agents comprising the background regimen should be taken according to the manufacturer's product labeling.

5.3.4. Compliance

Subjects will bring unused UK-427,857/placebo tablets to each study visit. The number of tablets will be counted and, if more than expected, subjects will be asked to account for missed doses. The Investigator and medical monitor will also evaluate subject compliance with the study regimen based on information such as plasma HIV-1 RNA levels. Potential reasons for non-compliance with UK-427,857 dosing (ie, AEs, lost medication) will be followed up by the study site personnel and strategies to improve dosing compliance will be explored. Compliance with optimized background therapy will be assessed through the use of a patient adherence diary. Compliance will not be assessed during the observational phase of the study.

5.4. Drug Storage and Drug Accountability

The Investigator, or an approved representative (eg, pharmacist), will ensure that all study drug is stored in a secured area, under recommended storage conditions and in accordance with applicable regulatory requirements. Antiretroviral agents comprising the background regimen must be stored according to manufacturer's guidelines.

To ensure adequate records, all study drug will be accounted for in the case report form and drug accountability inventory forms as instructed by Pfizer. During the observational phase of the study, study drug will be accounted for only in the drug accountability inventory form. Unless otherwise authorized by Pfizer, all unallocated or unused drug supplies must be returned to the

090177e1815d915c\Approved\Approved On: 24-Aug-2010 20:15

090177e1815d915c\Approved\Approved On: 24-Aug-2010 20:15

Sponsor or its appointed agent (eg, CRO) at the end of the clinical trial. Subjects must return all bottles to the Investigator, who will then return the bottles to the Sponsor or an appointed agent (eg, CRO) for review of dosing records and destruction.

5.5. Concomitant Medication(s)

Optimized background therapy, consisting of three to six approved branded antiretroviral agents will be selected by the Investigator on the basis of resistance testing, treatment history and safety considerations. These will be open-label and not provided by the sponsor. Experimental antiretroviral agents available through pre-approval access programs or by other means may be appropriate for use as part of background therapy following approval by the medical monitor on an individualized basis. Patients experiencing toxicity attributed to drugs in the background regimen would be able to substitute a drug of the same class during the trial in consultation with the medical monitor. All concomitant antiretroviral agents must be recorded on the CRF. No other anti-HIV therapy will be allowed while on study medication.

As UK-427,857 is a substrate for CYP3A4 inhibition, the dose of UK-427,857 will require adjustment based on the concomitant antiretroviral administered in the background regimen.

Concomitant Antiretrovirals	Recommended UK-427,857 Dose
≥1 PI (except tipranavir/ritonavir)** and/or delavirdine	150 mg
All other regimens	300 mg
Efavirenz or etravirine when not in the presence of a PI-or other strong CYP3A4 inhibitor*	600 mg

PI = protease inhibitor
* The use of efavirenz or etravirine with tipranavir is not recommended.
**The recommended dose of maraviroc is 300 mg unit dose twice daily when tipranavir/ritonavir is included as part of optimized background therapy.

Subjects taking efavirenz as part of their OBT should consider the addition of a PI as part of their OBT. In a pharmacokinetic study (A4001011), the mean reduction in UK-427,857 AUC_τ and C_{max} was approximately 50% in the presence of efavirenz. Increasing the UK-427,857 dose compensated for this reduction. As demonstrated in a follow-up pharmacokinetic study (A4001021), the effect of this induction due to efavirenz was similar in the presence and absence of a CYP3A4 inhibitor (Kaletra™, boosted saquinavir), resulting in an approximate 50% reduction in C_{max} and AUC_τ from that seen in the presence of the inhibitor alone. However, in this study, which evaluated the effects of combinations of metabolic inducers and inhibitors on the pharmacokinetics of UK-427,857, the net effect was that of inhibition.

Medications such as analgesics, anti-inflammatory agents, antibiotics and nutritional supplements other than those listed as contraindicated below, may be used as needed for treatment of adverse events and preexisting conditions. Prophylaxis for *Pneumocystis carinii* pneumonia (PCP) is strongly recommended for subjects with CD4 cell counts ≤200 cells/mm^3 or who have had a prior episode of PCP. Routine immunizations are permitted, as well as the use of erythropoietin and/or G-CSF and/or blood transfusions in the appropriate clinical situation.

Patients who require the use of rifampin for the treatment of either a *M. tuberculosis* or non-tuberculosis mycobacterial infection will be allowed to continue on study drug, assuming no changes need to be made in background therapy other than dose modification of existing therapy or changes within a class (eg, efavirenz to nevirapine). UK-427,857 (or placebo) dose will not be adjusted.

As UK-427,857 is a substrate for CYP3A4 inhibition, the following agents should not be co-administered during the study period to patients receiving a 300 mg unit dose, due the risk of drug-drug interactions: ketoconazole, itraconazole, miconazole, clotrimazole, troleandomycin, nefazadone, clarithromycin, rifampin and rifabutin. The use of rifampin (rifabutin) for the treatment of a mycobacterial infection may be considered following consultation between the clinician and Pfizer medical monitor.

The following agents should not be co-administered during the study period:

- Immunomodulators (for the treatment of HIV-1 infection; required agents such as interferon for the ongoing treatment of hepatitis C infection is permitted);
- Grapefruit or grapefruit juice;
- Over-the-counter medicines (except paracetamol and multivitamins) including St. John's Wort; or
- Other herbal or food supplements;
- Isoniazid;

This list is not exhaustive and new therapies may arise that would need to be considered as exclusionary.

5.6. Rescue Therapy

If a patient meets the criteria for treatment failure or discontinues for another reason (eg, pregnancy, adverse event) and requires an alternative regimen, they will be followed where possible until they reach their Week 96 visit according to protocol visit and assessment guidelines (ie, In-Study Off Drug). Each subject will be included in the observational follow-up phase for long-term survival and selected endpoints (LTS/SE) data collection.

The new regimen, selected by the Investigator based on the results of resistance testing at the time of failure, must be recorded in the CRF. For patients whose virus still remains CCR5-tropic and potentially sensitive to UK-427,857, open-label study drug may be continued during this period. The Investigator, in consultation with the medical monitor, will evaluate the appropriateness of continued therapy with open-label UK-427,857 based on ongoing review of clinical and laboratory parameters. Patients that receive open-label UK-427,857 will follow the protocol designed visit schedule and procedures. If a patients viral load increases beyond three times the mean baseline value upon confirmation of such value, the patient will be considered a treatment failure during the open-label dosing phase. The primary investigator will discontinue open label drug following consultation with the Study medical monitor.

090177e1815d915c\Approved\Approved On: 24-Aug-2010 20:15

Women who become pregnant during the study period must be unblinded to study drug following end of study assessments. The Investigator, in consultation with the medical monitor, should decide on the optimal antiretroviral regimen on an individualized basis. Protocol-required procedures for follow-up must be performed unless contraindicated by pregnancy. Other appropriate pregnancy follow-up procedures related to perinatal care and neonatal outcome should be performed and data provided via an updated Serious Adverse Event Monitoring (AEM) form. A detailed fetal anomalies ultrasound scan should be performed between Weeks 12 and 16 of gestation and recorded via submission of an updated AEM form. In addition, the Investigator must complete an Exposure in utero form. Infants inadvertently exposed to maraviroc in utero during this clinical trial will be referred, if geographical access permits, to select US and European sites that are already conducting follow-up studies of ARV-exposed infants.

All subjects will continue on double-blind therapy (via IVRS), until the last subject enrolled has reached Week 48 and the study data have undergone formal analysis. Following unblinding of the study, the Sponsor will provide open label UK-427,857 for study subjects for whom it is medically appropriate (see below) to continue or begin therapy with UK-427,857 until each subject reaches 5 years from their first dose of double-blind study treatment. The protocol assessment period will continue until each enrolled subject reaches their Week 96 visit or has been enrolled in the LTS/SE portion of the study; during this time subjects will continue to be monitored in accordance with protocol-specific investigations on an every 12 week visit schedule, but the "On study" procedures (Section 6.2.4) will exclude pharmacokinetic sampling and the collection of ECGs (See Appendix 5). ECGs are not required after the subject's Week 48 visit. While the study endpoints will have been reached, regulatory agencies require further long-term safety data of UK-427,857 in antiretroviral-experienced patients.

As each subject reaches Week 96, they will enter the LTS/SE with their follow up period extending 5 years from their first dose of double-blind study treatment. For subjects that have already passed their Week 96 visit, they will roll into the LTS/SE at their next scheduled regular visit.

5.6.1. Subject Eligibility for Open Label Maraviroc After Unblinding of Subjects at Week 48.

After subjects have reached week 48 and have been unblinded, they may be eligible for open label maraviroc BID. Eligibility will be determined as follows:

- Subjects who were on maraviroc and are ≤50 copies/ml may continue on maraviroc. All subjects receiving maraviroc QD will be changed to maraviroc BID. Consultation with the medical monitor is not required.
- Subjects who were on maraviroc and are ≥50 copies/ml and are pending or have met a protocol viral load stopping rule may be eligible for maraviroc BID. Consultation with the medical monitor is required.

090177e1815d915c\Approved\Approved On: 24-Aug-2010 20:15

- Subjects who were on placebo and are ≤50 copies/ml will not be eligible for maraviroc. However, if the subject is experiencing toxicity due to their OBT and the investigator feels it would be in the best interest of the subject to change, maraviroc may be considered Consultation with the medical monitor is required.
- Subjects who were on placebo and are pending or have met a protocol viral load stopping rule may be eligible for maraviroc. If subjects are enrolled in the LTS/SE and fail on their OBT and have never received maraviroc, may be eligible for open label maraviroc BID. Consultation with the medical monitor is required.

Since the primary endpoint of the study was week 48 and in the interest of the retention of subjects to week 96 and into the LTS/SE, changes in OBT will be allowed. As new anti-retrovirals become available, guidelines for dosing with maraviroc will change. **Any changes in the OBT may require dose adjustment for open label maraviroc: please consult the Study Medical Monitor, if you are planning any OBT changes. It is important to continue recording OBT, even when the subject is enrolled in the LTS/SE.**

6. TRIAL PROCEDURES

An overview of the schedule of study visits and procedures is presented as a flowchart in the Protocol Synopsis. Subjects will have study related visits with site personnel at the following timepoints:

- Screening: will occur within ~6 weeks prior to initial dosing with study medication. Patients requiring additional time in obtaining their OBT may have an extension of the screening window. All deviations in the screening window will require discussion with the Pfizer medical monitor;
- Randomization: will occur within 1 week of dosing with study medication;
- Baseline: Is the day that dosing with study medication will begin. Study procedures should be performed prior to the first dose;
- Weeks 2, 4 and every 4 weeks thereafter until Week 24;
- Every 8 weeks until Week 48;
- Every 12 weeks after Week 48 until each subject reaches Week 96 or beyond but prior to the enactment of the LTS/SE;
- Every 6 months after the LTS/SE observational study begins.

Every attempt should be made to keep subjects to visit schedules ±4 days. It is anticipated that subjects may require additional unscheduled clinic visits. If a subject discontinues study therapy (either blinded or open label) prior to reaching the Week 96 visit, they will complete the End of Study procedures but will remain “in study off drug”, keeping to the scheduled visits and procedures as outlined. The schedule of evaluations is listed below.

090177e1815d915c\Approved\Approved On: 24-Aug-2010 20:15

6.1. Screening Visit (Day –42 to Day -28)

- Written informed consent;
- Review of inclusion/exclusion criteria;
- Orthostatic blood pressure monitoring;
- Serum chemistry and hematology;
- CD4 and CD8 lymphocyte count determinations (absolute and percent);
- Hepatitis screening panel (hepatitis B core antibody and surface antigen/antibody, hepatitis C antibody);
- Plasma HIV-1 RNA level as determined by the Roche Amplicor HIV-1 MONITOR test, standard method;
- Plasma sample for HIV-1 co-receptor tropism phenotype as determined by the Monogram Biosciences recombinant virus entry assay;
- Plasma sample for HIV-1 genotype and phenotype as determined by the Monogram Biosciences PhenoSense™ GT assay; and,
- Serum pregnancy testing for WOCBP (See Section 4.1).

6.2. Trial Period

6.2.1. Randomization Visit (Day -7 to Day -4)

Patients will have a randomization visit between Days –7 and –4 for all screening results to be reviewed and be randomized into the study if all the entry criteria are met. In addition, the following will be performed:

- Serum chemistry (review of Inclusion/Exclusion criteria);
- Plasma HIV-1 RNA level as determined by the Roche Amplicor HIV-1 MONITOR test, standard method (if the results of the standard method are <400 copies/mL, the ultrasensitive method will automatically be performed); (Note: this result will not be used to determine eligibility); and,
- Selection of background regimen by the Investigator and agreement by the subject; confirmation (via FAX) to the medical monitor (sponsor). The OBT selection may be chosen when PSGT and gp41 drug resistance results are available, as such final selection could take place prior to the Randomization visit. Assurance that the patient will have access to these medications at the Baseline/Day 1 visit (they must be brought by the patient to the investigative site at the Baseline Visit).

6.2.2. Baseline (Day 1) Evaluation – Prior to first dose

- Medical history, including HIV-related diagnoses and past antiretroviral use;
- Complete physical examination and vital signs (including orthostatic BP monitoring);

090177e1815d915c\Approved\Approved On: 24-Aug-2010 20:15

- Body weight (record height at baseline or at a visit through Week 24 only);
- Assessment of signs, symptoms and adverse events;
- Review of concomitant medications;
- Serum chemistry and hematology;
- Fasting Metabolic Assessment (total cholesterol, HDL/LDL, triglycerides, glucose, glycosylated hemoglobin);
- 12-lead electrocardiogram;
- Urinalysis;
- Hepatitis C virus RNA if hepatitis C virus antibody positive at screening;
- CD4 and CD8 lymphocyte count determinations (absolute and percent);
- Plasma HIV-1 RNA level as determined by the Roche Amplicor HIV-1 MONITOR test, standard method (If the results of the standard method are <400 copies/mL, the ultrasensitive method will automatically be performed);
- Plasma sample for HIV-1 co-receptor tropism phenotype as determined by the Monogram Biosciences recombinant virus entry assay;
- Plasma sample stored for potential HIV-1 gp160 sequencing;
- Blood sample for preparation of two 1 mL plasma aliquots (frozen) and whole blood (17 mL) sample at ambient temperature for the purpose of PBMC and proviral DNA preparation and storage for future testing;
- Urine pregnancy testing for WOCBP (See Section 4.1). A positive urine test will require a confirmatory serum pregnancy test;
- 9 mL blood sample for host genotyping (unless prohibited by local regulations);
- Thyroid Function Tests (free T4, TSH); and,
- Dispense Study medication.

6.2.3. Week 2 Visit

- Assessment of signs, symptoms and adverse events;
- Body weight;
- Review of concomitant medications;
- Serum chemistry and hematology;
- Orthostatic BP monitoring (to be performed immediately prior to the first pharmacokinetic sample);
- Two 5 mL pharmacokinetic samples separated by at least 30 minutes;
- CD4 and CD8 lymphocyte count determinations (absolute and percent);

090177e1815d915c\Approved\Approved On: 24-Aug-2010 20:15

- Plasma HIV-1 RNA level as determined by the Roche Amplicor HIV-1 MONITOR test, standard method (If the results of the standard method are <400 copies/mL, the ultrasensitive method will automatically be performed);
- Review dosing compliance; and,
- Dispense Study medication (container from previous visit).

6.2.4. On Study Evaluations (Week 4, 8, 12, 16, 20, 32 and 40). Post Week 48, every 12 weeks thereafter: Week 60, 72, 84, 96, etc.

- Targeted physical examination and vital signs;
- Body weight at all visits through Week 20;
- Assessment of signs, symptoms and adverse events;
- Review of concomitant medications;
- Serum chemistry and hematology;
- One 5 mL pharmacokinetic sample at all visits through Week 20;
- Hepatitis C virus RNA if hepatitis C virus antibody positive at screening (Week 12 only);
- CD4 and CD8 lymphocyte count determinations (absolute and percent);
- Plasma HIV-1 RNA level as determined by the Roche Amplicor HIV-1 MONITOR test, standard method (if the results of the standard method are <400 copies/mL, the ultrasensitive method will automatically be performed);
- Blood sample for preparation of two 1 mL plasma aliquots (frozen) stored for future testing;
- Urine pregnancy testing for WOCBP (See Section 4.1). A positive urine test will require a confirmatory serum pregnancy test;
- Plasma sample for HIV-1 co-receptor tropism phenotype as determined by the Monogram Biosciences recombinant virus entry assay upon treatment failure as defined in the protocol, (this sample is to be drawn when the confirmatory plasma HIV-1 RNA sample is collected) if this occurs and for patients with HIV-1 RNA >500 copies/mL at Weeks 4, 8, 16, 32 and 40 only;
- Plasma sample for HIV-1 genotype and phenotype as determined by the Monogram Biosciences PhenoSense GT assay upon treatment failure only as defined in the protocol (this sample is to be drawn when the confirmatory plasma HIV-1 RNA sample is collected);
- Plasma sample for potential HIV-1 gp160 sequencing upon treatment failure only (as defined in the protocol);
- Review dosing compliance; and,
- Dispense Study medication (except where the study subject is “in study, off drug”).

090177e1815d915c\Approved\Approved On: 24-Aug-2010 20:15

6.2.5. Week 24 and 48 or Early Termination Visit

- Complete physical examination and vital signs;
- Body weight (at Week 24 only);
- Assessment of signs, symptoms and adverse events;
- Review of concomitant medications;
- Serum chemistry and hematology;
- Fasting Metabolic Assessment (total cholesterol, HDL/LDL, triglycerides, glucose, glycosylated hemoglobin);
- 12-lead electrocardiogram and orthostatic BP monitoring (to be performed immediately prior to the first pharmacokinetic sample). This is NOT required at scheduled or Early Termination visits occurring after Week 48;
- Two 5 mL pharmacokinetic samples separated by at least 30 minutes (at Week 24 only);
- Urinalysis;
- Hepatitis C virus RNA if hepatitis C virus antibody positive at screening;
- CD4 and CD8 lymphocyte count determinations (absolute and percent);
- Plasma HIV-1 RNA level as determined by the Roche Amplicor HIV-1 MONITOR test, standard method (If the results of the standard method are <400 copies/mL, the ultrasensitive method will automatically be performed);
- Blood sample for preparation of two 1 mL plasma aliquots (frozen) and whole blood (17 mL) sample at ambient temperature for the purpose of PBMC and proviral DNA preparation and storage for future testing;
- Urine pregnancy testing for WOCBP (See Section 4.1). A positive urine test will require a confirmatory serum pregnancy test;
- Plasma sample for HIV-1 co-receptor tropism phenotype as determined by the Monogram Biosciences recombinant virus entry assay upon treatment failure as defined in the protocol (except at the Early Termination visit for those subjects who are treatment failures) or for patients with HIV-1 RNA >500 copies/mL. Subjects that are treatment failures should have a co-receptor tropism sample drawn when the confirmatory plasma HIV-1 RNA sample is collected;
- Plasma sample for HIV-1 genotype and phenotype as determined by the Monogram Biosciences PhenoSense GT assay upon treatment failure as defined in the protocol (except at the Early Termination visit for those subjects who are treatment failures) or for patients with HIV-1 RNA >500 copies/mL. Subjects that are treatment failures should have a PSGT sample drawn when the confirmatory plasma HIV-1 RNA sample is collected;

090177e1815d915c\Approved\Approved On: 24-Aug-2010 20:15

- Plasma sample for potential HIV-1 gp160 sequencing upon treatment failure (as defined in the protocol) or for patients with HIV-1 RNA >500 copies/mL;
- Thyroid Function Tests (free T4, TSH);
- Assess dosing compliance; and,
- Dispense Study medication.

6.2.6 Observation Follow-Up Phase: Long Term Survival and Selected Endpoint (LTS/SE)

Ongoing study subjects will be asked for their consent prior to entering the LTS/SE observational follow up phase. Subjects will be asked for their consent:

1. to be evaluated for LTS/SE every 6 months; the follow-up phase will continue for the duration of 5 years after first dose of double-blind study treatment;
2. to provide at least one named secondary contact/next-of-kin who may be contacted by the primary investigator in order to collect LTS/SE data;
3. to permit the investigator to contact the subject’s primary/other treating physician in order to collect LTS/SE data;
4. to provide their national identification or insurance number (where applicable and allowed by local law), so publicly available death registries can be consulted by the investigator site for LTS/SE data;
5. to allow the investigator site to collect any information concerning the clinical aspects and circumstances of death, death certificate, and autopsy in the event of the subject’s death.

Only anonymous survival outcomes will be made available to the sponsor and regulatory authorities. Personal information will remain confidential with the investigator.

For ongoing subjects, survival status will be collected every 6 months from their final scheduled visit. Every 6 months after Week 96 (or final scheduled visit), subjects will be questioned about the occurrence of the following events:

- liver failure;
- myocardial infarction and ischemia;
- malignancies;
- Category C events;
- infections reported as serious adverse events;
- rhabdomyolysis.

In subjects who are ISOD or previously discontinued, the follow up may be accomplished by a telephone interview. However, it is the Sponsor’s preference to conduct the assessment face to face.

090177e1815d915c\Approved\Approved On: 24-Aug-2010 20:15

6.2.5.1. Subjects Who Previously Discontinued

Subjects who have already discontinued from the study or were lost to follow-up, but did not explicitly withdraw consent, will be contacted (two documented telephone calls and one certified letter) to consent for the observational follow-up phase (LTS/SE). Information concerning mortality will be recorded and each subject will be followed for LTS/SE for 5 years from their first dose of double-blind study treatment.

If the subject consents for LTS/SE, they will be followed for mortality and selected endpoints as above. If the subject consents for follow up of survival status, it will be collected at the time of consent and every 6 months thereafter. If the subject has died any information concerning the clinical aspects and circumstances of death, death certificate, and autopsy should be obtained. In particular, deaths that are selected endpoints of interest should be recorded as such in the CRF. The status of each subject should be recorded even though the investigator has had no contact or knowledge of the subject. If the subject's status is unknown, the last date the subject was known to be alive will be recorded. In the course of clinical practice, information may become available about the mortality status of a subject by public sources, other patients, or relatives, the report of a death should be recorded.

090177e1815d915c\Approved\Approved On: 24-Aug-2010 20:15

6.3. Population Pharmacokinetics

During each of the Week 4, 8, 12, 16 and 20 visits, one venous blood samples should be taken from each patient. During each of the Week 2 and 24 visits, two venous blood samples should be taken from each patient. The time of draw of the two samples should be as far apart as possible and should be separated by at least 30 minutes. It is recommended that the first sample be obtained as soon as the possible upon arrival, and the second immediately prior to leaving the clinic at each appointment.

Where possible, the samples at each visit should be obtained at different times post-dose relative to each other. To this end it is recommended that the:

- Week 2 and 8 visit appointments should be scheduled in the morning.
- Week 4 and 16 visit appointments should be scheduled at around midday.
- Week 12 visit appointment should be scheduled in the afternoon.
- Week 20 and 24 visits appointment should be scheduled in the morning.

For subjects who have been discontinued from the study and remain in-study, off drug, samples for pharmacokinetic testing are not required after the early termination visit is completed.

6.3.1. Population Pharmacokinetics: Dosing History

For the final pharmacokinetic analysis, it is important that the date and time of administration of the previous study drug dose before collection of the pharmacokinetic sample (PK dose), and the dose before that, be accurately recorded in the source documents and CRF at all visits. Similarly, it is important that the date and time that each blood sample is drawn is accurately recorded in the CRF.

090177e1815d915c\Approved\Approved On: 24-Aug-2010 20:15

6.3.2. Population Pharmacokinetics: Meal Times History

The date and time of the last meal prior to the PK dose should also be recorded in the CRF.

6.4. Drug Assay

At a time determined by the study monitor, the samples will be transported in dry ice to Covance Central Laboratory and stored until being shipped to Tandem Analytics where they will be assayed for UK-427,857 using a previously validated method. Final UK-427,857 plasma concentration data will be transferred to the Sponsor. All samples must be stored and shipped according to the procedures detailed in the Central Laboratory Manual.

6.5. Virology Testing

6.5.1. Viral Load Measurements

Viral load will be determined using the RT-PCR (Roche Amplicor v1.5) assay with a lower limit of detection of 400 copies/mL as standard. For samples with a reading of <400 copies/mL, the ultrasensitive method with a lower limit of detection of 50 copies/mL will be used. Blood samples (10 mL) will be taken at the following timepoints:

- Screening;
- Randomization;
- Baseline;
- Weeks 2, 4, 8, 12, 16, 20, 24, 32, 40 and 48;
- Early Termination Visit.

Blood will be collected, processed, stored and shipped to Covance in accordance with the procedure documented in a separate sample-handling document. Baseline will be calculated as the mean of the three pre-dose values, however, only the screening value will be used to determine eligibility for the study.

6.5.2. Virus Tropism

Virus tropism will be determined using the Monogram Biosciences PhenoSense™ Entry Assay. Samples will not be analyzed if the viral load is <500 copies/mL. Blood samples (10 mL) will be taken at the following time points:

- Screening;
- Baseline;
- Weeks 4, 8, 16, 24, 32, 40 and 48;
- At the time of treatment failure (to be drawn when the confirmatory plasma HIV-1 RNA sample is collected);

- Early Termination Visit (if not due to treatment failure).

Blood will be collected, processed, stored and shipped in accordance with the procedure documented in a separate handling document. A part of the sample may be retained and archived for potential analysis of other virus tropism assays if deemed necessary.

V3 loop sequencing, alone or as part of gp160 sequencing, will be performed at baseline, Weeks 24 and 48 and at treatment failure.

6.5.3. Viral Resistance

Phenotypic and genotypic resistance to PIs, NRTIs and NNRTIs will be evaluated using the Monogram Biosciences PhenoSense™ GT assay. Genotypic resistance to enfuvirtide will be determined using gp41 sequencing. Blood samples (10 mL) will be taken at the following timepoints:

- Screening;
- Weeks 24 and 48;
- At the time of treatment failure (to be drawn when the confirmatory plasma HIV-1 RNA sample is collected);
- Early Termination Visit (if not due to treatment failure).

Blood will be collected, processed, stored and shipped in accordance with the procedure documented in a separate handling document.

6.5.4. Virus Susceptibility to UK-427,857

Blood samples (17 mL) will be taken to provide stored PBMCs and proviral DNA at the following timepoints:

- Baseline;
- Weeks 24 and 48;
- At the time of treatment failure;
- Early Termination Visit.

Blood will be collected, processed, stored and shipped in accordance with the procedure documented in a separate handling document after which PBMCs will be prepared and stored according to a standardized protocol. At time of treatment failure these samples will be used to attempt virus isolation and susceptibility testing according to a standardized protocol.

Other virus characteristics that could impact on susceptibility to UK-427,857 and virological response may also be evaluated on a subset of patient samples at baseline and at the time of treatment failure. These potentially will include gp160 sequencing, virus replication assays and virus subtyping.

090177e1815d915c\Approved\Approved On: 24-Aug-2010 20:15

6.5.5. Blood Pressure, Heart Rate and ECG Monitoring

Supine and standing BP and HR measurements will be recorded using a semi-automated sphygmomanometer, and a 12-lead ECG will be recorded at the following times:

- Screening (BP and HR only);
- Baseline;
- Weeks 2 (BP and HR only), 24 and 48;
- Early Termination Visit (ECG is not required after Week 48*)*.

Supine blood pressure will be recorded after 5 minutes lying down; patients will then sit for 2 minutes, followed by standing for 2 minutes after which a standing blood pressure will be recorded.

Patients with postural hypotension at the baseline visit will be monitored for 4 hours following the first dose of study drug and any adverse events recorded. Any adverse events felt potentially related to postural hypotension at this visit should be discussed immediately with the medial monitor or designee.

Supine BP and HR measurements will be recorded using a semi-automated sphygmomanometer at the following times:

- Weeks 4, 8, 12, 16, 20, and 32.

During the subsequent evaluation and treatment period, episodes of postural hypotension and potentially related events should be managed in the same fashion as other adverse events, with causality and severity assessed by the Investigator and a determination as to whether the event meets the criteria for a Serious Adverse Event and/or requires discontinuation of study drug. The Investigator and patient will assess the risk/benefit of continued dosing with study drug in consultation with the medical monitor.

6.5.6. Host Genotyping

In recognition of the fact that genetic variation in the CCR5 locus is well documented and that it is possible that the antiretroviral activity of UK-427,857 may vary between individuals based on their CCR5 genotype and in response to a request from regulators to include pharmacogenetic analysis in the development program. Prior to the first dose (of UK-427,857 or placebo) a 9 ml blood sample will be collected for non-anonymous genotyping. In this context, non-anonymous means that the genotyping results can be linked to the subject who donated the sample via the patient identifier. Initially, samples will be genotyped to determine the patient's CCR5 Δ32 status and for other CCR5 locus polymorphisms. A patient's CCR5 Δ32 genotyping results will be transferred to the appropriate investigator following completion of the trial if the patient requests the information. The results will only be forwarded on a per patient basis.

090177e1815d915c\Approved\Approved On: 24-Aug-2010 20:15

In addition, genotyping of drug metabolizing enzymes, drug transport proteins (for example CYP3A4, CYP3A5, MDR1) or other genes that might influence, safety, toleration or efficacy of UK-427,857 (for example, those involved in blood pressure homeostasis such as ADRA1A, ACE) may also be performed in the event of unusual patterns of response, or an unexplained excess of adverse events. If additional genotyping is performed these results will **ONLY** be used to assess the impact of genetic variation on response to UK-427,857 and will not be transferred back to the investigators. The blood samples will initially be stored at the central laboratory prior to being transferred to Pfizer Global Research and Development, 2800 Plymouth Road, Safety Sciences-Bldg 30 Rm 151C, Ann Arbor, MI 48105 USA or an appropriate contract laboratory for genotyping. The samples will be retained until all regulatory post approval commitments have been satisfied, after which they will be destroyed. Genotyping samples will be collected from all subjects unless prohibited by local regulations.

6.6. Subject Withdrawal

A subject may withdraw from the trial at any time at their own request, or they may be withdrawn at any time at the discretion of the investigator or sponsor for safety, behavioral, or administrative reasons. If a subject does not return for a scheduled visit, every effort should be made to contact the subject. In any circumstance, every effort should be made to document subject outcome, if possible.

If the subject withdraws consent, no further evaluations should be performed and no attempts should be made to collect additional data.

The primary reason for a patient discontinuing from study drug or the clinical study will be recorded in the CRF. The investigator must determine the primary reason for discontinuation. Withdrawal due to adverse events must be distinguished from withdrawal due to insufficient efficacy. A discontinuation must be reported immediately to the Study medical monitor or his/her designated representative if it is due to a serious adverse event. If a subject is discontinued from the study or study treatment due to a drug-related adverse event, the subject must be followed until the adverse event has resolved or until the event is determined to be chronic or stable in nature. The End of Study procedures must be performed at the time of discontinuation (and prior to unblinding) from either study drug or from the clinical study.

All planned discontinuations must be discussed with the medical monitor or his/her designated representative prior to investigator unblinding. Pfizer personnel directly involved in the conduct of the study will remain blinded to these individual subjects who continue “in study off drug”.

A subject may be withdrawn from study therapy at any time for any of the following reasons; however, they will remain “in study, off drug” and will be monitored according to the protocol specified guidelines until each subject enrolled reaches Week 96 or enrolls in the observational phase of the study:

- An adverse event or laboratory abnormality requiring drug discontinuation;
- Pregnancy;

090177e1815d915c\Approved\Approved On: 24-Aug-2010 20:15

- Planned enrollment into another study (including, but not limited to intervention, laboratory, psychological, investigational drug, device, or biological studies).
- Treatment failure, defined as:
  - An increase to at least 3 times the baseline (mean of all 3 values before start of dosing) plasma HIV-1 RNA level at the Week 2 visit or thereafter (confirmed by a second measurement taken no more than 14 days after the first measurement);
  - HIV-1 RNA <0.5 $\log_{10}$ decrease from baseline (mean of all 3 values before start of dosing) on 2 consecutive measurements starting at Week 8 (second measurement taken no more than 14 days after the first measurement);
  - HIV-1 RNA <1.0 $\log_{10}$ decrease from baseline (mean of all 3 values before start of dosing) on 2 consecutive measurements starting at Week 8 (second measurement taken no more than 14 days after the first measurement), in a patient who had previously achieved a ≥2.0 $\log_{10}$ decrease from baseline; or;
  - An increase in HIV-1 RNA to ≥5,000 copies/mL on two consecutive measurements taken no more than 14 days apart, in subjects previously confirmed to have undetectable levels of <400 copies/mL on 2 consecutive visits;
  - In the Investigator's opinion, it is in the subject's best interest.

In addition, subjects may be formally discontinued from the study for any of the following reasons:

- Subject's decision not to participate any further (withdrawal of consent);
- In the Investigator's opinion, it is in the subject's best interest;
- The study is terminated by the Sponsor;
- Lack of compliance with administration of study medication or with protocol procedures such that reliable safety and efficacy assessments are compromised.

Efforts will be made to continue to follow up on those subjects who discontinue study drug, "in study, off-drug", until the last subject enrolled reaches Week 96 or enrolls in the observational phase. In addition, unless consent has been withdrawn or is refused, all subjects will be followed up for survival and selected endpoints regardless of the time at which they discontinue, or have discontinued, from the study: this post-study observational period will extend for a minimum of 5 years from each subject's first dose of double-blind study treatment.

7. ASSESSMENTS

7.1. Efficacy Assessments

All subjects will have antiviral activity assessed by HIV-1 PCR RNA levels and immunological status assessed by CD4 and CD8 counts at specified study visits. Blood samples for viral tropism and resistance-testing will drawn at specified study visits.

090177e1815d915c\Approved\Approved On: 24-Aug-2010 20:15

090177e1815d915c\Approved\Approved On: 24-Aug-2010 20:15

7.2. Safety Assessments

Safety will be assessed by spontaneous reports, physical examination and laboratory test results in all subjects who received at least one dose of study medication.

Safety assessments will be done at specified study visits and will include the following:

- Medical history;
- Complete or symptom directed physical examination;
- Vital signs;
- Laboratory tests (eg, hepatitis testing, pregnancy testing, hematology, chemistry, urinalysis and fasting metabolic assessment).

Other assessments will include recording of concomitant medications and adverse events.

If a subject develops a Grade 3 abnormality (with the exception of hypercholesterolemia, hypertriglyceridemia, asymptomatic CPK elevations, AST/ALT in the absence of a total bilirubin elevation >2.5 times ULN, anemia, glucose or asymptomatic amylase or lipase elevations) or a Grade 4 laboratory abnormality (with the exception of hypercholesterolemia and hypertriglyceridemia), the Investigator will immediately discuss the case with the medical monitor. Study medication must be discontinued immediately unless the medical monitor and Investigator agree that there is an adequate explanation for the abnormality and the subject can be safely continued. If the decision is made to interrupt therapy, subjects may remain in the study (and on study drug) if the abnormality has decreased to Grade 1 or 2 and rechallenge can occur within 14 days of stopping therapy. Longer treatment interruptions for the management of suspected drug toxicity or the recurrence of a Grade 3 or Grade 4 abnormality following rechallenge will require permanent discontinuation of study drug. These patients will remain "in study off drug" and will be followed with appropriate medical management until there is a return to baseline values or a clinical diagnosis of an intercurrent illness has been established.

Specific guidelines for the management of patients with abnormalities in hepatic enzymes are provided in Appendix 2.

8. ADVERSE EVENT REPORTING

Following study drug discontinuation, adverse events will be considered as potentially related to study drug (UK-427,857) until another medication is taken. Adverse events may then be ascribed as potentially related to this new medication.

8.1. Adverse Events

All observed or volunteered adverse events regardless of treatment group or suspected causal relationship to the investigational product(s) will be recorded on the adverse event page(s) of the case report form (CRF), except during the observational phase of the study. Adverse events that are on-going at the time of the subject enrolls in the observational phase will be recorded as continuing in the CRF.

For all adverse events, the investigator must pursue and obtain information adequate both to determine the outcome of the adverse event and to assess whether it meets the criteria for classification as a serious adverse event requiring immediate notification to Pfizer or its designated representative (See Section 8.4). For all adverse events, sufficient information should be obtained by the investigator to determine the causality of the adverse event. The investigator is required to assess causality and indicate that assessment on the CRF. Follow-up of the adverse event, after the date of therapy discontinuation, is required if the adverse event or its sequelae persist. Follow-up is required until the event or its sequelae resolve or stabilize at a level acceptable to the investigator and the Study medical monitor or designee.

8.2. Definition of an Adverse Event

An adverse event is any untoward medical occurrence in a clinical investigation subject administered a product or medical device; the event need not necessarily have a causal relationship with the treatment or usage. Examples of adverse events include but are not limited to:

- Abnormal test findings;
- Clinically significant symptoms and signs;
- Changes in physical examination findings;
- Hypersensitivity;
- Progression/worsening of underlying disease;
- Lack of effect.

Additionally, they may include the signs or symptoms resulting from:

- Drug overdose;
- Drug withdrawal;
- Drug abuse;
- Drug misuse;
- Drug interactions;
- Drug dependency;
- Extravasation;
- Exposure In Utero;
- Lack of or insufficient clinical response, benefit, efficacy or therapeutic effect should not be recorded as an adverse event.

For sentinel HIV-related infections, as well as AIDS-defining opportunistic infections and malignancies, detailed information will be collected. A list of these will be provided in Appendix 1.

090177e1815d915c\Approved\Approved On: 24-Aug-2010 20:15

8.3. Abnormal Laboratory Findings

A designated central laboratory will perform laboratory safety tests. Any screening laboratory result outside the inclusion or exclusion criteria may be repeated. If repeat screening results remain outside the eligibility criteria, the subject should not be enrolled unless there is prior written agreement between the sponsor and the Investigator to allow an exemption from the inclusion or exclusion criteria.

Changes of one or more grades in total bilirubin between screening and the randomization visit (including the Randomization visit results) should be reviewed with the Study medical monitor before the initiation of double-blind study medication. Laboratory safety tests will be performed at the following time points: (See Study Schedule/Flowchart).

- Screening;
- Randomization;
- Day 1 (Baseline) Pre-dose;
- Week 2;
- On Study Evaluations (Week 4, 8, 12, 16, 20, 32 and 40);
- Week 24 and 48 or Early Termination Visit.

8.3.1. Laboratory Tests will include:

1. **Hematology:** hemoglobin, RBC, hematocrit, WBC (including differentials) and platelet count.
2. **Clinical Chemistry:** total bilirubin (direct/indirect if elevated), total protein, albumin, uric acid, sodium, potassium, chloride, bicarbonate, BUN, creatinine, calcium, AST, ALT, lactate dehydrogenase, alkaline phosphatase, GGTP (if alkaline phosphatase elevated), creatine kinase, amylase and lipase (if amylase elevated). Fasting metabolic assessment, performed at baseline, Week 24 and Week 48 or Early Termination, will include glucose (at all other times glucose will be non-fasting), glycosylated hemoglobin, total cholesterol, high density lipoprotein cholesterol and triglycerides.
3. **Serum Pregnancy Test:** On women of childbearing potential (ie, females who have experienced menarche and who have not undergone successful surgical sterilization or are not post-menopausal). Serum pregnancy will be performed at the Screening visit and Urine test will be used at the following visits. A positive urine test will require a confirmatory serum pregnancy test.
4. **Hepatitis Testing:** At screening will include hepatitis B surface antigen (HBsAg), hepatitis B surface antibody (HBsAb), hepatitis B core antibody (HBcAb) and hepatitis C antibody (anti-HCV). If HCV positive at screening, HCV RNA should be determined at baseline and Weeks 12, 24 and 48 or Early Termination.

090177e1815d915c\Approved\Approved On: 24-Aug-2010 20:15

5. **Blood:** Will be collected for thyroid function tests (free T4 and TSH), CD4/CD8 cell counts, plasma HIV-1 RNA, viral tropism and drug resistance assays (See Section 6.5) and pharmacokinetic testing (See Section 6.3).

   Blood will be collected, processed, stored and shipped in accordance with the procedures outlined in a separate handling protocol. A total of 8.5 mL of blood will be taken for clinical chemistry and 4 mL for hematology measurements.

6. **Urine:** Urinalysis will include color, specific gravity, pH, blood, protein, glucose, ketones, bilirubin and a microscopic examination, which will include casts, crystals, WBC and RBC.

7. **CSF:** Cerebrospinal fluid (CSF) samples for measurement of UK-427,857 levels will be collected in patients who undergo diagnostic or therapeutic lumbar- or ventricular punctures during the study (See Appendix 2).

8.3.2. Follow Up of Laboratory Test Abnormalities

Abnormalities of any laboratory test considered to represent a significant danger to the patient will lead to immediate discontinuation of study drug. If a subject develops a Grade 3 abnormality (with the exception of hypercholesterolemia, hypertriglyceridemia, asymptomatic CPK elevations, AST/ALT in the absence of a total bilirubin elevation >2.5 times ULN, anemia, glucose or asymptomatic amylase or lipase elevations) or a Grade 4 laboratory abnormality (with the exception of hypercholesterolemia and hypertriglyceridemia), the Investigator will immediately discuss the case with the medical monitor. Study medication must be discontinued immediately unless the medical monitor and Investigator agree that there is an adequate explanation for the abnormality and the subject can be safely continued. If the decision is made to interrupt therapy, subjects may remain in the study (and on study drug) if the abnormality has decreased to Grade 1 or 2 and rechallenge can occur within 14 days of stopping therapy. Longer treatment interruptions for the management of suspected drug toxicity or the recurrence of a Grade 3 or Grade 4 abnormality following rechallenge will require permanent discontinuation of study drug. These patients will remain “in study off drug” and will be followed with appropriate medical management until there is a return to baseline values or a clinical diagnosis of an intercurrent illness has been established.

Specific guidelines for the management of patients with abnormalities in hepatic enzymes are provided in Appendix 2.

The results of all laboratory tests required by the protocol will be recorded in the subject’s case report form. All clinically important abnormal laboratory tests occurring during the study will be repeated at appropriate intervals until they return either to baseline or to a level deemed acceptable by the investigator and the Study clinical monitor (or his/her designated representative), or until a diagnosis that explains them is made.

The criteria for determining whether an abnormal objective test finding should be reported as an adverse event are as follows:

- Test result is associated with accompanying symptoms, and/or;

090177e1815d915c\Approved\Approved On: 24-Aug-2010 20:15

- Test result requires additional diagnostic testing or medical/surgical intervention, and/or;
- Test result leads to a change in trial dosing or discontinuation from the study, significant additional concomitant drug treatment, or other therapy, and/or;
- Test result is considered to be an adverse event by the investigator or sponsor.

Merely repeating an abnormal test, in the absence of any of the above conditions, does not constitute an adverse event. Any abnormal test result that is determined to be an error does not require reporting as an adverse event.

8.4. Serious Adverse Events

A serious adverse event or serious adverse drug reaction is any untoward medical occurrence at any dose that:

- Results in death;
- Is life-threatening;
- Requires inpatient hospitalization or prolongation of existing hospitalization;
- Results in persistent or significant disability/incapacity;
- Results in congenital anomaly/birth defect.

Medical and scientific judgment should be exercised in determining whether an event is an important medical event. An important medical event may not be immediately life-threatening and/or result in death or hospitalization. However, if it is determined that the event may jeopardize the subject and may require intervention to prevent one of the other outcomes listed in the definition above, the important medical event should be reported as serious.

Examples of such events are intensive treatment in an emergency room or at home for allergic bronchospasm; blood dyscrasias or convulsions that do not result in hospitalization; or development of drug dependency or drug abuse.[*]

8.5. Hospitalization

Adverse events reported from clinical trials associated with hospitalization or prolongation of hospitalization are considered serious. Any initial admission (even if less than 24 hours) to a healthcare facility meets these criteria. Admission also includes transfer within the hospital to an acute/intensive care unit (eg, from the psychiatric wing to a medical floor, medical floor to a coronary care unit, neurological floor to a tuberculosis unit).

Hospitalization does not include the following:

- Rehabilitation facilities;

[*] 21CFR 312.32

090177e1815d915c\Approved\Approved On: 24-Aug-2010 20:15

- Hospice facilities;
- Respite care (eg, caregiver relief);
- Skilled nursing facilities;
- Nursing homes;
- Routine emergency room admissions;
- Same day surgeries (as outpatient/same day/ambulatory procedures).

Hospitalization or prolongation of hospitalization in the absence of a precipitating, clinical adverse event is not in itself a Serious Adverse Event. Examples include:

- Admission for treatment of a preexisting condition not associated with the development of a new adverse event or with a worsening of the preexisting condition (eg, for work-up of persistent pre-treatment lab abnormality);
- Social admission (eg, subject has no place to sleep);
- Administrative admission (eg, for yearly physical exam);
- Protocol-specified admission during a clinical trial (eg, for a procedure required by the trial protocol);
- Optional admission not associated with a precipitating clinical adverse event (eg, for elective cosmetic surgery);
- Pre-planned treatments or surgical procedures should be noted in the baseline documentation for the entire protocol and/or for the individual subject.

Diagnostic and therapeutic non-invasive and invasive procedures, such as surgery, should not be reported as adverse events. However, the medical condition for which the procedure was performed should be reported if it meets the definition of an adverse event. For example, an acute appendicitis that begins during the adverse event reporting period should be reported as the adverse event, and the resulting appendectomy should be recorded as treatment of the adverse event.

8.6. Severity Assessment

If required on the adverse event case report forms, the investigator will use the adjectives MILD, MODERATE, SEVERE or VERY SEVERE to describe the maximum intensity of the adverse event. For purposes of consistency, these intensity grades are defined as follows:	
MILD	Events which are usually transient, requiring no special treatment, and do not interfere with the patient's daily activities.
MODERATE	Events which introduce a low level of inconvenience or concern to the patient and may interfere with daily activities, but are usually ameliorated by simple therapeutic measures.
SEVERE	Events that interrupt the patient's usual daily activity and traditionally require systemic drug therapy or other treatment.
VERY SEVERE	Events which are unacceptable and intolerable or which are irreversible or cause the patient to be in imminent danger of death.

090177e1815d915c\Approved\Approved On: 24-Aug-2010 20:15

Note the distinction between the severity and the seriousness of an adverse event. A severe event is not necessarily a serious event. For example, a headache may be severe (interferes significantly with subject's usual function) but would not be classified as serious unless it met one of the criteria for serious adverse events, listed above. See Appendix 6 for a listing of grading severities of adverse events.

8.7. Exposure In Utero

For investigational products within clinical trials and for marketed products, an Exposure In-Utero (EIU) occurs if:

1. A female becomes, or is found to be, pregnant either while receiving or having been directly exposed to (eg, environmental exposure) the medicinal product, or the female becomes, or is found to be, pregnant after discontinuing and/or being directly exposed to the medicinal product (maternal exposure).
2. A male has been exposed, either due to treatment or environmental, to the medicinal product prior to or around the time of conception and/or is exposed during the partner pregnancy (paternal exposure).

In each case meeting the definition of exposure *in utero* involving either a ViiV Healthcare medicinal product or a Pfizer medicinal product, whether investigational or marketed, the investigator must submit the required information to Pfizer on an Exposure in Utero Form. The investigator must submit information regarding study subjects or their partners and in the case of environmental exposure to ViiV Healthcare medicinal product or a Pfizer medicinal product in a pregnant woman (eg, a nurse reports that she is pregnant and has been exposed to a cytotoxic product by inhalation or spillage) using the Exposure in Utero Form. This must be done irrespective of whether an adverse event has occurred and within 24 hours of awareness of the pregnancy. The information submitted should include the anticipated date of delivery (See below for information related to induced termination of pregnancy).

Follow-up is conducted to obtain pregnancy outcome information on all Exposure in Utero reports with an unknown outcome. The investigator will follow the subject until completion of the pregnancy or until pregnancy termination (ie, induced abortion) and then notify the Study medical monitor or designee of the outcome. The investigator will provide this information as a follow up to the initial Exposure in Utero Form. The reason(s) for an induced abortion should be specified. An EIU report is not created when an ectopic pregnancy report is received since this pregnancy is not usually viable. Rather, an SAE case is created with the event of ectopic pregnancy.

If the outcome of the pregnancy meets the criteria for immediate classification as a serious adverse event (ie, spontaneous abortion, stillbirth, neonatal death, or congenital anomaly [including that in an aborted fetus, stillbirth or neonatal death]), the investigator should follow the procedures for reporting serious adverse events.

090177e1815d915c\Approved\Approved On: 24-Aug-2010 20:15

In the case of a live birth, the "normality" of the newborn can be assessed at the time of birth (ie, no minimum follow-up period of a presumably normal infant is required before an Exposure in Utero Form can be completed). The "normality" of an aborted fetus can be assessed by gross visual inspection, unless pre-abortion test findings are suggestive of a congenital anomaly.

Additional information about pregnancy outcomes that are classified as serious adverse events follows:

- "Spontaneous abortion" includes miscarriage and missed abortion.
- All neonatal deaths that occur within 1 month of birth should be reported, without regard to causality, as serious adverse events. In addition, any infant death after 1 month that the investigator assesses as possibly related to the in utero exposure to the investigational medication should be reported.

Additional information regarding the exposure in utero may be requested by the investigator. Further follow-up of birth outcomes will be handled on a case-by-case basis (eg, follow-up on preterm infants to identify developmental delays). In the case of paternal exposure, the investigator must obtain permission from the subject's partner in order to conduct any follow-up or collect any information.

8.8. Discontinuations (See Also Subject Withdrawal, Section 6.6)

The reason for a subject discontinuing from the trial will be recorded in the CRF. A discontinuation occurs when an enrolled subject ceases participation in the study, regardless of the circumstances, prior to completion of the protocol. The investigator must determine the primary reason for discontinuation. Withdrawal due to adverse event should be distinguished from withdrawal due to insufficient response, according to the definition of adverse event noted earlier, and recorded on the appropriate adverse event CRF page.

When a discontinuation is due to a serious adverse event, the serious adverse event must be reported in accordance with the reporting requirements defined below.

8.9. Eliciting Adverse Event Information

The investigator is to report all directly observed adverse events and all adverse events spontaneously reported by the trial subject. In addition, each trial subject will be questioned about adverse events at each clinic visit. The question asked will be "Since your last clinic visit have you had any health problems?"

8.10. Reporting Requirements (Serious and Nonserious)

Each adverse event is to be classified by the investigator as serious or nonserious. This classification determines the reporting procedures to be followed. If a serious adverse event occurs, expedited reporting will follow local and international regulations, as appropriate.

090177e1815d915c\Approved\Approved On: 24-Aug-2010 20:15

SAEs are reportable from the time that the subject provides informed consent, which is obtained prior to the subject's participation in the clinical trial, ie, prior to undergoing any trial-related procedure and/or receiving investigational product, through and including a minimum of 28 calendar days after the last administration of the investigational product. All SAE's and causality occurring while a subject is participating on the protocol including those patients who are "in study, off drug" must be reported, regardless of the time after the last administration of drug. Any serious adverse event occurring at any other time after completion of the study must be promptly reported if a causal relationship to study drug is suspected.

If a serious adverse event occurs, the Study medical monitor or designee is to be notified within 24 hours of awareness of the event by the investigator. In particular, if the serious adverse event is fatal or life-threatening, notification to the Study medical monitor or designee must be made immediately, irrespective of the extent of available adverse event information. This timeframe also applies to additional new information (follow-up) on previously forwarded serious adverse event reports.

In the rare event that the investigator does not become aware of the occurrence of a serious adverse event immediately (eg, if an outpatient trial subject initially seeks treatment elsewhere), the investigator is to report the event within 24 hours after learning of it and document the time of his/her first awareness of the adverse event.

For all serious adverse events, the investigator is obligated to pursue and provide information to the Study medical monitor or designee in accordance with the timeframes for reporting specified above. In addition, an investigator may be requested by the Study medical monitor or designee to obtain specific additional follow-up information in an expedited fashion. This information may be more detailed than that captured on the adverse event case report form. In general, this will include a description of the adverse event in sufficient detail to allow for a complete medical assessment of the case and independent determination of possible causality. Information on other possible causes of the event, such as concomitant medications and illnesses must be provided. In the case of a subject death, a summary of available autopsy findings must be submitted as soon as possible to Pfizer or its designated representative.

The investigator's assessment of causality must be provided. An investigator's causality assessment is the determination of whether there exists a reasonable possibility that the investigational product caused or contributed to an adverse event. If the investigator's final determination of causality is unknown and the investigator does not know whether or not study drug caused the event, then the event will be handled as "related to study drug" for reporting purposes. If the investigator's causality assessment is "unknown but not related to study drug", this should be clearly documented on study records. In addition, if the investigator determines the adverse event is associated with trial procedures, the investigator must record this causal relationship in the source documents and CRF, as appropriate, and report such an assessment in accordance with the serious adverse event reporting requirements, if applicable.

090177e1815d915c\Approved\Approved On: 24-Aug-2010 20:15

All adverse events will be reported on the adverse event page(s) of the CRF, except during the observation phase of the study. It should be noted that the form for collection of serious adverse event information is not the same as the adverse event CRF. Where the same data are collected, the forms must be completed in a consistent manner. For example, the same adverse event term should be used on both forms. Adverse events should be reported using concise medical terminology on the CRFs as well as on the form for collection of serious adverse event information.

Except during the observational phase of the study, nonserious adverse events are to be reported on the adverse event CRFs, which are to be submitted to Pfizer as specified in the adverse event report submission procedure for this protocol.

8.11. Additional Safety Information

No mechanistically specific antagonists for UK-427,857 are available and standard supportive measures should be used in the case of excessive pharmacological effects.

9. DATA ANALYSIS/STATISTICAL METHODS

Detailed methodology for summary and statistical analyses of the data collected in this trial will be documented in an Analysis Plan, which will be dated and maintained by the sponsor. This document may modify the plans outlined in the protocol; however, any major modifications of the primary endpoint definition and/or its analysis will also be reflected in a protocol amendment.

9.1. Sample Size Determination

A total of 600 subjects will be randomized in a 2:2:1 ratio (240 on UK-427,857 150 mg QD, 240 on UK-427,857 150 mg BID and 120 on placebo). Under the assumption of a standard deviation of 0.8, with a 2-sided significance level of 0.025 (Bonferroni adjustment for multiple comparisons) there will be >95% power to detect a difference of 0.5 in change from baseline in log-transformed HIV RNA levels between each UK-427,857 arm and placebo. This is the primary efficacy endpoint. The primary time point will be 48 weeks and it will also be analyzed at 24 weeks.

9.2. Efficacy Analysis

9.2.1. Analysis Populations

All tables and figures in this report will be based on one of three populations. Efficacy tables will be based on the "Full Analysis Set" and "Per protocol set". Safety tables will be based on the "Safety Population".

The Full Analysis Set will consist of all randomized patients who receive at least one dose of study medication. It will use the intention to treat (ITT) approach.

090177e1815d915c\Approved\Approved On: 24-Aug-2010 20:15

The per protocol set will consist of all randomized patients who meet the following criteria:

- Receive at least one dose of study medication,
- Treated for at least 14 days or discontinued before this time due to treatment failure,
- More than 80% compliant with randomized treatment,
- No violation of any inclusion or exclusion criteria, which would affect efficacy (such as tropism status).

It will use the intention to treat (ITT) approach.

The Safety Population will consist of all randomized patients who received at least one dose of study medication. Patients will be included in the dose group they actually receive.

9.2.2. Analysis of Primary Endpoint

9.2.2.1. Primary Endpoint

- The primary efficacy variable is the change from baseline in $\log_{10}$ transformed HIV-1 RNA levels.
- The primary efficacy time point is 48 weeks. This analysis will occur after all randomized subjects have reached the end of 48 weeks or discontinued prior to reaching 48 weeks. The primary efficacy variable will also be analyzed at 24 weeks.

9.2.2.2. Method of Analysis

Plasma HIV-1 RNA levels will be determined by the Roche Amplicor HIV–1 MONITOR Test, Standard assay. If the results of the standard method are <400 copies/mL the ultrasensitive assay will automatically be performed. For the analysis, HIV-1 RNA level for each subject at each time point will be defined based on results from the standard assay when the value is ≥400 copies/mL, and based on the results from the ultrasensitive assay when standard assay result is <400 copies/mL. Any HIV-1 RNA value <50 copies/mL (the LOQ from the ultrasensitive assay) will be treated as 49.

The primary endpoint is the change from baseline to 48 weeks in log-transformed HIV-1 RNA levels. Baseline will be calculated as the mean of all 3 values before start of dosing. These are collected at screening, randomization and immediately pre-dose. If one or two values are missing baseline is calculated from those present. Baseline is only missing if all 3 values are missing. All values are $\log_{10}$ transformed before the calculation. Only those data collected during the blinded treatment phase will be used for the primary analysis.

An ANCOVA model will be used with screening viral load level (randomization strata), enfuvirtide use, geographic region and treatment group as the main effects. The least squares mean treatment difference between each UK-427,857 dose group and placebo will be presented. A Bonferroni adjustment will be used to account for multiple comparisons.

090177e1815d915c\Approved\Approved On: 24-Aug-2010 20:15

Interactions of screening viral load level (randomization strata), enfuvirtide use and geographic region with treatment will be investigated. No main effect of treatment will be presented in the presence of an interaction term.

For the primary analysis subjects who discontinue the study before the timepoint of interest will have their final value imputed as baseline (ie, no change from baseline). Also, no change from baseline will be imputed for subjects with missing baseline or no viral load assessment on treatment.

To further assess the robustness of the primary analysis two sensitivity analyses will be performed:

1. the last observation carried forward approach will be used. This analysis will include only those subjects with an assessment of viral load at baseline and while on treatment.
2. subjects who discontinued before Week 24 (or 48) due to any reason, apart from protocol defined treatment failure (See Section 6.6), will have no change from baseline imputed. Treatment failures will use the last observation carried forward approach as in the sensitivity analysis above.

If the assumption of normality is seriously violated then the treatment effect will be assessed using non-parametric methods and non-parametric confidence intervals for the difference between treatments will be presented.

9.2.3. Analysis of Secondary Endpoints

9.2.3.1. Secondary Endpoints

The following secondary efficacy variables will be analyzed:

- Percentage of subjects with HIV-1 RNA levels <400 copies/mL;
- Percentage of subjects with HIV-1 RNA levels <400 copies/mL or having at least 0.5-log-transformed decrease from baseline;
- Percentage of subjects with HIV-1 RNA levels <400 copies/mL or having at least 1.0-log-transformed decrease from baseline;
- Percentage of subjects with HIV-1 RNA levels <50 copies/mL;
- Change from baseline in CD4 cell count;
- Change from baseline in CD8 cell count;
- Time to virological failure;

090177e1815d915c\Approved\Approved On: 24-Aug-2010 20:15

- Time-Averaged Difference (TAD) in $\log_{10}$ HIV-1 RNA;
- Genotype and phenotype at baseline and at the time of failure;
- Tropism at baseline and at the time of failure;
- Association between baseline resistance and virological response;
- All secondary variables will be analyzed at Week 24 and Week 48, apart from time to virological failure, which will only be analyzed as Week 48.

9.2.3.2. Method of Analysis

- The percentage of subjects with HIV-1 RNA levels <400 copies/mL, the percentage of subjects with HIV-1 RNA levels <400 copies/mL or having at least 0.5-log-transformed decrease from baseline, the percentage of subjects with HIV-1 RNA levels <400 copies/mL or having at least 1.0-log-transformed decrease from baseline and the percentage of subjects with HIV-1 RNA levels <50 copies/mL will be summarized for the ITT population using a last observation carried forward (LOCF) approach and at each visit. A Cochran-Mantel-Haenszel test will be performed and ninety-seven point five percent (97.5%) confidence intervals for the difference in percentages between each of the UK-427,857 dose groups and placebo will be provided using the normal approximation. These will be adjusted based on the randomization strata.
- These variables will also be analyzed using logistic regression including screening viral load level (<100,000 versus ≥100,000 copies/mL) (randomization strata), enfuvirtide use and geographic region (Northern Hemisphere/Southern Hemisphere) as factors.

TAD in $\log_{10}$ HIV-1 RNA, change from baseline in CD4 and CD8 cell count will be analyzed using an ANCOVA model with screening viral load level, CD4 or CD8 cell count respectively, enfuvirtide use, geographic region and treatment arm as the main effects.

Time to virological failure (loss-of-virological-response) will be based on following algorithm:

Day 1 for this analysis is defined as the first day of dosing. Time to virological failure is time from Day 1 until virological failure as defined below.

1. For 2. and 3. below, discard all visits with no data. In what follows, a visit means a visit with an observed viral load. Viral load data from all available visits, including off-schedule visits and post Week 48 visits, should be included for the calculation.
2. If a subject had never achieved a confirmed HIV-1 RNA level below 400 copies/mL (on 2 consecutive visits) before the following events, then this subject will be considered to have failed at Time 0:
   a. Death;

090177e1815d915c\Approved\Approved On: 24-Aug-2010 20:15

   b. Permanent discontinuation of the test drug or loss to follow-up;
   c. Introduction of a new anti-retroviral drug to the regimen, with the exception of changing a drug in the background therapy to a drug of the same class due to either toxicity or intolerance that is attributed to the background drugs, but not the test drug or its control;
   d. Last available visit;
   e. Entered open label due to early non-response or rebound (treatment failure criteria defined in Section 3 of the protocol).

3. For all subjects who had confirmed HIV-1 RNA levels below 400 copies/mL, ie, on 2 consecutive visits, the time of failure is the earliest time when a specific event had occurred. Those events are modifications in 4. and are listed below:
   a. Death;
   b. Permanent discontinuation of the test drug or loss to follow-up;
   c. The event as described in 2c;
   d. Entered open label due to early non-response or rebound (treatment failure criteria as described in protocol);
   e. Confirmed HIV-1 RNA levels above or equal to 400 copies/mL, defined as HIV-1 RNA levels from 2 consecutive visits are greater than or equal to 400 copies/mL or 1 visit ≥400 copies/mL followed by permanent discontinuation of the test drug or loss to follow-up.
4. If the time of virological failure defined above is immediately preceded by a single missing scheduled visit or multiple consecutive missing scheduled visits, then the time of virological failure is replaced by the first time of such missing visits.

A subject who has not failed during the study will be censored at the last available visit.

Time to virological failure will be summarized using Kaplan-Meier curves and the difference between each of the UK-427,857 arms and the control arm will be analyzed using a log-rank test, stratified by randomization strata.

Virus genotype and phenotype at baseline and at the time of failure will be summarized, as will tropism at baseline and at the time of failure.

Association between baseline resistance and virological response will be evaluated by summarizing the change from baseline in log-transformed HIV-1 RNA levels with respect to different baseline resistance factors. These are: NNRTI Resistance Subgroups, Selected Baseline NRTI-Resistance Mutation Groups, PI-Resistance Mutation, baseline enfuvirtide Resistance Mutation, Number of Baseline NNRTI-resistance Mutations, Individual Baseline NNRTI-resistance Mutations and V3 loop genotype. Baseline phenotypic and genotypic sensitivity

090177e1815d915c\Approved\Approved On: 24-Aug-2010 20:15

scores (PSS and GSS), defined as the number of agents in OBT to which the subject's virus showed phenotypic or, respectively, genotypic susceptibility will be assessed. The association between virological response and baseline PSS and GSS will be summarized.

Baseline NRTI Resistance, PI Resistance, NNRTI Resistance, enfuvirtide resistance, V3 loop genotype and tropism phenotype will be summarized.

9.3. Analysis of Other Endpoints

9.3.1. Pharmacokinetic Sampling and Population PK

A population analysis of time versus plasma concentration data of UK-427,857 will be performed using the nonlinear mixed effects modeling approach. The software NONMEM (UCSF, California, USA) will be used to derive the population mean (and variance) values for specific pharmacokinetic parameters. Additionally, a relationship between pharmacokinetic parameters (or dose) and efficacy, as well as adverse events, will be investigated.

In the pharmacokinetic (and pharmacodynamic) analysis a number of covariates will be tested and incorporated into the structural model if shown to significantly improve the model's ability to describe the data. The final PK/PD model for UK-427,857 will be obtained from this "full" model using only the covariate relationships that are thought to result in clinically significant alterations in drug pharmacokinetics and/ or pharmacodynamics.

A more detailed description of the methodology to be followed is given in the "Population Pharmacokinetic/Pharmacodynamic Analysis Plan for UK-427,857 Phase 2b/Phase 3 Data".

9.4. Safety Analysis

Safety will be assessed by spontaneous reports, physical examination, and laboratory test results in all subjects who received at least one dose of study medication.

9.5. Interim Analysis

No formal interim analysis will be performed. However, a DSMB will review the first 100 patients treated through 8 weeks to confirm whether this study should continue. Recruitment will continue during the DSMB review of the first 100 patients unless the overall treatment failure (ie, subjects who meet one of the prespecified stopping rules) rate at Week 8 exceeds 21% in studies A4001027 and A4001028 combined. This is based on the overall failure rates for TORO-1 and TORO-2 at week 8.[6, 7] However, recruitment may be allowed to continue during this time period, despite exceeding a 21% failure rate, following an ad-hoc review of the data by the DSMB and its recommendation to continue recruitment.

Subsequently, at approximately every 12-16 weeks, a DSMB will meet to discuss the results from this and the 3 other studies in the UK-427,857 program. For this study, summaries including demography, adverse events, discontinuations due to adverse events, viral load, CD4 cell count and tropism status will be provided by an independent Statistical Data Analysis

090177e1815d915c\Approved\Approved On: 24-Aug-2010 20:15

Center (SDAC) statistician to the DSMB. No formal statistical analysis will be performed and there will be no stopping rule based on virological response, although the DSMB may recommend terminating the study if they feel that continuing would compromise patient safety. Patients will be protected by individual patient stopping rules based on treatment failure. All Pfizer personnel responsible for the clinical trial conduct will remain blinded to the data provided to the DSMB. No statistical adjustment will be made in the final analysis for these interim looks.

9.6. Data Monitoring Committee

To help assure the safety of study participants, an independent Data Safety Monitoring Board (DSMB) will be formed. The DSMB will be responsible for evaluating the progress of the trial including periodic assessments of efficacy and safety data. In addition, the DSMB will make recommendations concerning continuation, termination or other modifications of the trial based on the observed beneficial or adverse effects of the treatment under study.

The DSMB will be made up of seven members with a Chairperson and statistician. A quorum will consist of 5 members. There will be both an open and closed portion of all DSMB meetings. The DSMB will report its recommendation to the Sponsor Management Committee. In reports prepared for the drug safety monitoring board, treatments will be labeled “D”, “E” and “F”. The assignment of actual assigned treatment to these labels will not be contained in the report; however, the convention will be consistent between reports. If the DSMB chooses to have the actual treatment assignments disclosed, a vote could be taken to authorize such disclosure. A full description of the DSMB process is detailed in the UK-427, 857 DSMB Charter.

10. QUALITY CONTROL AND QUALITY ASSURANCE

During trial conduct, Pfizer, or its agent, or the Sponsor will conduct periodic monitoring visits to ensure that the protocol and GCPs are being followed. The monitors may review source documents to confirm that the data recorded on CRFs is accurate. The investigator and institution will allow Pfizer monitors or its agents, or the sponsor, and appropriate regulatory authorities direct access to source documents to perform this verification.

The trial site may be subject to review by the institutional review board (IRB)/independent ethics committee (IEC), and/or to quality assurance audits performed by Pfizer or the Sponsor, and/or to inspection by appropriate regulatory authorities from the US or other countries.

It is important that the investigator(s) and their relevant personnel are available during the monitoring visits and possible audits or inspections and that sufficient time is devoted to the process.

11. DATA HANDLING AND RECORD KEEPING

11.1. Case Report Forms/Electronic Data Record

As used in this protocol, the term case report form (CRF) should be understood to refer to either a paper form or an electronic data record or both, depending on the data collection method used in this trial.

090177e1815d915c\Approved\Approved On: 24-Aug-2010 20:15

A CRF is required and should be completed for each included subject. The completed original CRFs are the sole property of the Sponsor and should not be made available in any form to third parties, except for authorized representatives of the Sponsor or appropriate regulatory authorities, without written permission from the Sponsor.

It is the investigator's responsibility to ensure completion and to review and approve all CRFs. CRFs must be signed by the investigator or by an authorized staff member. These signatures serve to attest that the information contained on the CRFs is true. At all times, the investigator has final personal responsibility for the accuracy and authenticity of all clinical and laboratory data entered on the CRFs. Subject source documents are the physician's subject records maintained at the trial site. In most cases, the source documents will be the hospital's or the physician's chart. In cases where the source documents are the hospital or the physician's chart, the information collected on the CRFs must match those charts.

In some cases, the CRF may also serve as the source document. In these cases, Pfizer and the investigator must prospectively document which items will be recorded in the source documents and for which items the CRF will stand as the source document.

11.2. Record Retention

To enable evaluations and/or audits from regulatory authorities or the Sponsor and its authorized representatives, the investigator agrees to keep records, including the identity of all participating subjects (sufficient information to link records, eg, CRFs and hospital records), all original signed informed consent forms, copies of all CRFs, source documents, and detailed records of treatment disposition. The records should be retained by the investigator according to ICH, local regulations, or as specified in the Clinical Study Agreement, whichever is longer.

If the investigator relocates, retires, or for any reason withdraws from the trial, Pfizer should be prospectively notified. The trial records must be transferred to an acceptable designee, such as another investigator, another institution, or to Pfizer. The investigator must obtain the Sponsor's written permission before disposing of any records, even if retention requirements have been met.

12. ETHICS

12.1. Institutional Review Board (IRB)/Independent Ethics Committee (IEC)

It is the responsibility of the investigator to obtain prospective approval of the trial protocol, protocol amendments, Clinical Pharmacogenomics Supplement (if applicable), informed consent forms, and other relevant documents, eg, advertisements, if applicable, from the IRB/IEC. All correspondence with the IRB/IEC should be retained in the Investigator File. Copies of IRB/IEC approvals should be forwarded to Pfizer.

The only circumstance in which an amendment may be initiated prior to IRB/IEC approval is where the change is necessary to eliminate apparent immediate hazards to the subjects. In that event, the investigator must notify the IRB/IEC and Pfizer in writing within 5 working days after the implementation.

090177e1815d915c\Approved\Approved On: 24-Aug-2010 20:15

12.2. Ethical Conduct of the Trial

The trial will be performed in accordance with the protocol, International Conference on Harmonization Good Clinical Practice guidelines, and applicable local regulatory requirements and laws.

12.3. Subject Information and Consent

The informed consent form must be agreed to by the Sponsor and the IRB/IEC and must be in compliance with ICH GCP, local regulatory requirements, and legal requirements.

The investigator must ensure that each trial subject, or his/her legally acceptable representative, is fully informed about the nature and objectives of the trial and possible risks associated with participation. The investigator will obtain written informed consent from each subject or the subject's legally acceptable representative before any study-specific activity is performed. The informed consent form used in this trial, and any changes made during the course of the trial, must be prospectively approved by both the IRB/IEC and the Sponsor before use. The investigator will retain a copy of each subject's signed consent form.

13. SPONSOR DISCONTINUATION CRITERIA

Premature termination of this clinical trial may occur because of a regulatory authority decision, change in opinion of the IRB/IEC, drug safety problems, or at the discretion of the Sponsor. In addition, the Sponsor retains the right to discontinue development of UK-427,857 at any time.

The Sponsor reserves the right to discontinue the trial prior to inclusion of the intended number of subjects, but intends only to exercise this right for valid scientific or administrative reasons. After such a decision, the investigator must contact all participating subjects within 24 hours. As directed by the Sponsor, all trial materials must be collected and all CRFs completed to the greatest extent possible.

14. PUBLICATION OF STUDY RESULTS

Publication of study results is discussed in the Clinical Study Agreement.

090177e1815d915c\Approved\Approved On: 24-Aug-2010 20:15

15. REFERENCES

1. UNAIDS Global Epidemic Report, December 2003.

2. Pozniak AI, Fatkenheuer G, Johnson M, et al. Effect of short-term monotherapy with UK-427,857 on viral load in HIV-infected patients. 43rd Annual Interscience Conference on Antimicrobial Agents and Chemotherapy, Sep 2003, Chicago, IL: Abstract 443.

3. Reynes J, Rouzier R, Kanouni T, et al. SCH-C: safety and antiviral effects of a CCR5 receptor antagonist in HIV-1 infected subjects. 9th Conference on Retroviruses and Opportunistic Infections, Feb 2002, Seattle, WA: Abstract 1.

4. Whitcomb JM, Huang W, Fransen S, et al. Analysis of baseline enfuvirtide (T20) susceptibility and co-receptor tropism in two Phase III study populations. 10th Conference on Retroviruses and Opportunistic Infections, Feb 2003, Boston, MA: Abstract 557.

5. Whitcomb JM, Huang W, Fransen S, et al. Analysis of baseline enfuvirtide (T20) susceptibility and co-receptor tropism in two Phase III study populations. 10th Conference on Retroviruses and Opportunistic Infections, Feb 2003, Boston, MA: Abstract 557.

6. Lalezari JP, Henry K, O'Hearn M et al. Enfuvirtide, an HIV-1 fusion inhibitor, for drug-resistant HIV infection in North and South America. N Engl J Med 2003; 348:2175-2185.

7. Lazzarin A, Clotet B, Cooper D, et al. Efficacy of enfuvirtide in patients infected with drug resistant HIV-1 in Europe and Australia. N Engl J Med 2003; 348:2186-2195.

090177e1815d915c\Approved\Approved On: 24-Aug-2010 20:15

Appendix 1. AIDS-DEFINING OPPORTUNISTIC ILLNESSES (OIS) Clinical Category C Events for Adolescents and Adults (CDC HIV Classification System)

I. PARASITIC INFECTIONS

A *PNEUMOCYSTIS CARINII (PCP)*

A101 Pulmonary PCP, histologically proven

A102 PCP, histologically proven at site other than lungs

B. *TOXOPLASMOSIS*

B101 Toxoplasmosis, clinical diagnosis (of brain only)

B102 Toxoplasmosis, proven by microscopy (of brain or other internal organ other than, liver, spleen, or lymph nodes)

C. *ISOSPORIASIS*

C101 Isosporiasis causing chronic diarrhea >1 month, proven by microscopy

D *CRYPTOSPORIDIOSIS*

D101 Cryptosporidiosis causing chronic diarrhea >1 month, proven by microscopy

II. FUNGAL INFECTIONS

A Candidiasis

A201 Candidiasis (esophageal), definitive diagnosis

A202 Candidiasis (esophageal), presumptive diagnosis

A203 Bronquial/pulmonary (lung, trachea), definitive diagnosis

B. *CRYPTOCOCCOSIS*

B201 Cryptococcosis, extrapulmonary, proven by microscopy

FUNGAL INFECTIONS (CONTINUED)

C. *HISTOPLASMOSIS*

C201 Histoplasmosis, extrapulmonary, disseminated, proven by microscopy

D Coccidioidomycosis

D201 Coccidioidomycosis, disseminated, at a site other than, or in addition to, lungs or cervical or hilar lymph nodes, proven by microscopy

III. BACTERIAL INFECTIONS

A Mycobacterium

A301 Mycobacterium tuberculosis, pulmonary

A302 Mycobacterium tuberculosis, extrapulmonary site regardless of pulmonary involvement

A303 Mycobacterium tuberculosis, disseminated, definitive diagnosis, proven by culture

B Non TB Mycobacterium Infections

B301 Mycobacterium Avium Intracellulare (MAI), extrapulmonary

B302 MAI in blood, proven by culture

B303 MAI colitis, proven by histology and culture

B304 MAI, disseminated, sites other than lungs or cervical or hilar lymph nodes, proven by culture

B305 M. kansasii, extrapulmonary only, proven by culture

B306 M. kansasii, in blood, proven by culture

B307 M. kansasii, colitis, proven by histology and culture

B308 M. kansasii, disseminated at site other than, or in addition to, lungs, or cervical, or hilar lymph nodes, proven by culture

BACTERIAL INFECTIONS (CONTINUED)

C *SALMONELLA*

C301 Salmonella (nontyphoid) bacteremia proven by culture, recurrent

IV. Viral Infections

A *CYTOMEGALOVIRUS (CMV)*

A401 CMV Retinitis

A402 CMV, other than liver, spleen, nodes

B *HERPES SIMPLEX VIRUS (HSV)*

B401 HSV Bronchitis

B402 HSV Pneumonitis

B403 HSV, mucocutaneous (lesions persisting for ≥1 month) proven by microscopy

B404 HSV, mucocutaneous, including HSV esophagitis

B405 HSV encephalitis

C *PROGRESSIVE MULTIFOCAL LEUKOENCEPHALOPATHY*

C401 Progressive Multifocal Leukoencephalopathy

V. NEOPLASTIC DISEASES

A *KAPOSI'S SARCOMA*

A501 Kaposi's sarcoma, mucocutaneous, proven by histology and cytology

A502 Kaposi's sarcoma, mucocutaneous, presumptive

A503 Kaposi's sarcoma, visceral

090177e1815d915c\Approved\Approved On: 24-Aug-2010 20:15

AIDS-Defining Opportunistic Illnesses (OIs) (continued)

Clinical Category C Events for Adolescents and Adults (CDC HIV Classification System)

NEOPLASTIC DISEASES (CONTINUED)

B *LYMPHOMA*

B501 Primary brain lymphoma proven by microscopy, Burkitt's, immunoblastic, sarcoma.

B502 Non-Hodgkin B-cell or unknown immunologic phenotype and histology showing small, noncleaved lymphoma

B503 Hodgkin

C *CERVICAL CARCINOMA*

C501 Histologically proven invasive carcinoma of the cervix

VI. OTHER CONDITIONS

A *HIV DEMENTIA/MOTOR DEFECTS*

A601 Loss of developmental milestones (pediatric)

A602 Progressive symmetrical motor defects (pediatric)

A603 Adult: Disabling cognitive and/or motor dysfunction interfering with activities.

B *HIV WASTING SYNDROME*

B601 HIV Wasting Syndrome: loss >10% of baseline + chronic diarrhea (>2 loose stools per day during >30 days) or chronic weakness and documented enigmatic fever >30 days.

HIV WASTING SYNDROME (CONTINUED)

B602 715

716

C 717

C601 Progressive symmetrical motor defects (pediatric) Adult: Disabling cognitive and/or motor

VII. PEDIATRICS

701 Persistent hepatomegaly

702 Persistent splenomegaly

703 Regression of development milestones (consistent regression over at least 3 months)

704 Recurrent or persistent upper respiratory infection, sinusitis or otitis media.

705 Bacterial meningitis, pneumonia, or sepsis (single episode)

706 Candidiasis, oropharyngis for >2 months

707 Cardiomyopathy

708 CMV infection

709 Diarrhea, recurrent or chronic

710 Hepatitis

711 Herpes zoster involving at least 2 distinct episodes or more than one dermatome.

712 Leiomyosarcoma

713 Lymphoid interstiticial pneumonia (LIP)

PEDIATRICS (CONTINUED)

714 Nocardiosis

715 Fever lasting >1 month

716 Varicella, disseminated (i.e., complicated chickenpox)

717 Toxoplasmosis of the brain with onset >1 month of age

VIII. SENTINEL INFECTIONS

1 Pneumococcus

2 Streptococcus

3 Staphylococcus

4 Enterococcus

5 Meningococcus

6 Mycoplasma

7 Chlamydia

8 Herpes virus

9 Cryptococcus

10 Candidiasis

11 Listeria

12 Haemophilus

13 Enteric bacteria

14 Pseudomonas

15 Legionella

16 Aspergillus

17 Protozoa

18 Helminth

19 Viral hepatitis

20 Other parasitic

21 Clostridia

22 Atypical mycobacteria

23 Mycobacteria

24 Spirochetal infections

25 Rickettsia
997 Other bacterial
998 Other viral
999 Other fungal

090177e1815d915c\Approved\Approved On: 24-Aug-2010 20:15

Appendix 2. Liver Enzyme Work-up

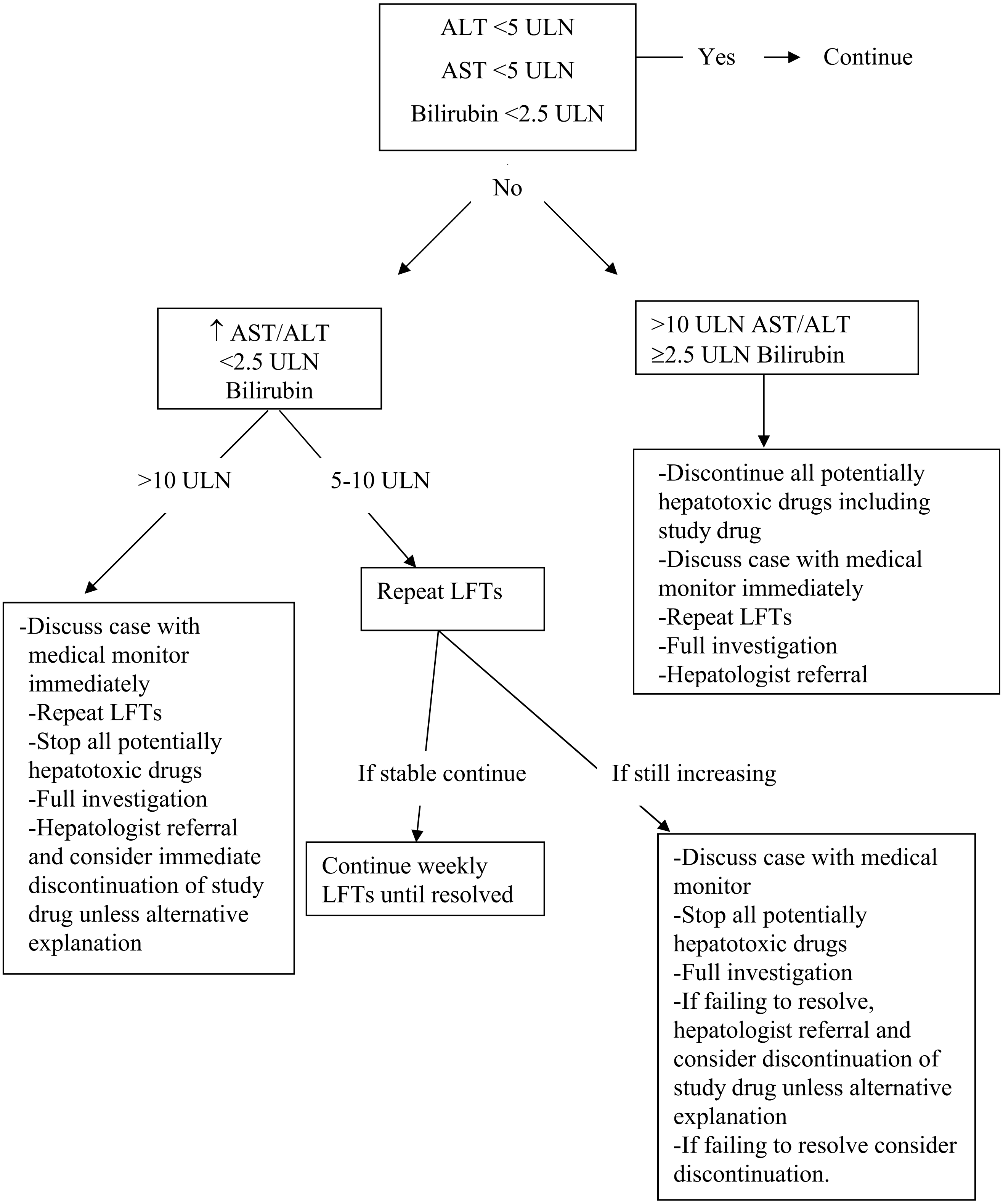

090177e1815d915c\Approved\Approved On: 24-Aug-2010 20:15

Appendix 3. COLLECTION OF CEREBROSPINAL FLUID

Cerebrospinal fluid (CSF) samples for measurement of UK-427,857 levels will be collected in patients who undergo diagnostic or therapeutic lumbar- or ventricular punctures during the study.

During the lumbar- or ventricular punctures CSF should be collected into two tubes. The first tube will be sent to the local laboratory for measurement of CSF protein, glucose concentrations and cell counts. The second tube will be centrifuged at 1500 g for 10 minutes and supernatant will be stored at –20°C until analyzed for UK-427,857 concentrations by the central laboratory.

On the same day in addition to CSF samples two blood samples will be collected at least 2 hours apart from each other. The detailed description of blood collection is given in Section 6.3.

The following data will be recorded in the CRF: method of the CSF collection, time of the last meal, time of the last two UK-427,857 doses, time of the blood and CSF sampling, concentrations of CSF protein, total WBC with differential and RBC.

Because of practical issues associated with lumbar puncture, it is likely that the majority of subjects will have just a single CSF sample collected during the study. Therefore population pharmacokinetic analysis will be used and the plasma concentrations of UK-427,857 will be calculated using the final model for the time of CSF sampling. The penetration of UK-427,857 into CSF will be described by the ratio of CSF and plasma concentrations. All visible bloody CSF samples will be excluded from the final analysis.

090177e1815d915c\Approved\Approved On: 24-Aug-2010 20:15

Appendix 4. Actg Grading Severity Of Adult Adverse Events

ABBREVIATIONS: Abbreviations utilized in the Table:

ULN = Upper Limit of Normal	LLN = Lower Limit of Normal
Rx = Therapy	Req = Required
Mod = Moderate	IV = Intravenous
ADL = Activities of Daily Living	Dec = Decreased

ESTIMATING SEVERITY GRADE

For abnormalities NOT found elsewhere on the Toxicity Table, use the scale below to estimate grade of severity:

**GRADE 1**	**Mild**	Transient or mild discomfort; no limitation in activity; no medical intervention/therapy required
**GRADE 2**	**Moderate**	Mild to moderate limitation in activity - some assistance may be needed; no or minimal medical intervention/therapy required
**GRADE 3**	**Severe**	Marked limitation in activity, some assistance usually required; medical intervention/therapy required, hospitalizations possible
**GRADE 4**	**Life-threatening**	Extreme limitation in activity, significant assistance required; significant medical intervention/therapy required, hospitalization or hospice care probable

SERIOUS OR LIFE-THREATENING AEs

ANY clinical event deemed by the clinician to be serious or life-threatening should be considered a Grade 4 adverse experience. Clinical events considered to be serious or life-threatening include, but are not limited to:

Seizures, coma, tetany, diabetic ketoacidosis, disseminated intravascular coagulation, diffuse petechiae, paralysis, acute psychosis

MISCELLANEOUS

a. When two values are used to define the criteria for each parameter, the lowest values will appear first.

b. Parameters are generally grouped by body system.

c. Some protocols may have additional protocol specific grading criteria.

090177e1815d915c\Approved\Approved On: 24-Aug-2010 20:15

090177e1815d915c\Approved\Approved On: 24-Aug-2010 20:15

ACTG GRADING SEVERITY OF ADULT ADVERSE EVENTS				
**PARAMETER**	**GRADE 1**	**GRADE 2**	**GRADE 3**	**GRADE 4**
	MILD	MODERATE	SEVERE	POTENTIALLY LIFE THREATENING
**HEMATOLOGY**				
Hemoglobin	8.0 g/dL - 9.4 g/dL	7.0 g/dL - 7.9 g/dL	6.5 g/dL - 6.9 g/dL	<6.5 g/dL
Absolute Neutrophil Count	1000 - 1500/mm^3	750 - 999/mm^3	500 - 749/mm^3	<500/mm^3
Platelets	75,000 - 99,000/mm^3	50,000 - 74,999/mm^3	20,000 - 49,999/mm^3	<20,000/mm^3
Prothrombin Time (PT)	>1.0 - 1.25 X ULN	>1.25 - 1.5 X ULN	>1.5 - 3.0 X ULN	>3 X ULN
PTT	>1.0 - 1.66 x ULN	>1.66 - 2.33 x ULN	>2.33 - 3.0 x ULN	>3.0 x ULN
Methemoglobin	5.0 - 10.0%	10.1 - 15.0%	15.1 - 20.0%	>20%
**CHEMISTRIES**				
SODIUM				
Hyponatremia	130 - 135 meq/L	123 - 129 meq/L	116 - 122 meq/L	<116 meq/L
Hypernatremia	146 - 150 meq/L	151 - 157 meq/L	158 - 165 meq/L	>165 meq/L
POTASSIUM				
Hypokalemia	3.0 - 3.4 meq/L	2.5 - 2.9 meq/L	2.0 - 2.4 meq/L	<2.0 meq/L
Hyperkalemia	5.6 - 6.0 meq/L	6.1 - 6.5 meq/L	6.6 - 7.0 meq/L	>7.0 meq/L
PHOSPHATE				
Hypophosphatemia	2.0 - 2.4 mg/dL	1.5 - 1.9 mg/dL	1.0 - 1.4 mg/dL	<1.0 mg/dL
CALCIUM - (corrected for albumin)				
Hypocalcemia	7.8 - 8.4 mg/dL	7.0 - 7.7 mg/dL	6.1 - 6.9 mg/dL	<6.1 mg/dL
Hypercalcemia	10.6 - 11.5 mg/dL	11.6 - 12.5 mg/dL	12.6 - 13.5 mg/dL	>13.5 mg/dL
MAGNESIUM				
Hypomagnesemia	1.2 - 1.4 meq/L	0.9 - 1.1 meq/L	0.6 - 0.8 meq/L	<0.6 meq/L
BILIRUBIN				
Hyperbilirubinemia	>1.0 - 1.5 x ULN	>1.5 - 2.5 x ULN	>2.5 - 5 x ULN	>5 x ULN

ACTG GRADING SEVERITY OF ADULT ADVERSE EVENTS				
**PARAMETER**	**GRADE 1**	**GRADE 2**	**GRADE 3**	**GRADE 4**
	MILD	MODERATE	SEVERE	POTENTIALLY LIFE THREATENING
GLUCOSE				
Hypoglycemia	55 - 64 mg/dL	40 - 54 mg/dL	30 - 39 mg/dL	<30 mg/
Hyperglycemia (nonfasting and no prior diabetes)	116 - 160 mg/dL	161 - 250 mg/dL	251 - 500 mg/dL	>500 mg/dL
Triglycerides	__________	400 - 750 mg/dL	751 - 1200 mg/dL	>1200 mg/dL
Creatinine	>1.0 - 1.5 x ULN	>1.5 - 3.0 x ULN	>3.0 - 6.0 x ULN	>6.0 x ULN
URIC ACID				
Hyperuricemia	7.5 - 10.0 mg/dL	10.1 - 12.0 mg/dL	12.1 - 15.0 mg/dL	>15.0 mg/dL
**LIVER TRANSAMINASE (LFTs)**				
AST (SGOT)	1.25 - 2.5 x ULN	>2.5 - 5.0 x ULN	>5.0 - 10.0 x ULN	>10.0 x ULN
ALT (SGPT)	1.25 - 2.5 x ULN	>2.5 - 5.0 x ULN	>5.0 - 10.0 x ULN	>10.0 x ULN
GGT	1.25 - 2.5 x ULN	>2.5 - 5.0 x ULN	>5.0 - 10.0 x ULN	>10.0 x ULN
Alk Phos	1.25 - 2.5 x ULN	>2.5 - 5.0 x ULN	>5.0 - 10.0 x ULN	>10.0 x ULN
**PANCREATIC ENZYMES**				
Amylase	>1.0 - 1.5 x ULN	>1.5 - 2.0 x ULN	>2.0 - 5.0 x ULN	>5.0 x ULN
Pancreatic amylase	>1.0 - 1.5 x ULN	>1.5 - 2.0 x ULN	>2.0 - 5.0 x ULN	>5.0 x ULN
Lipase	>1.0 - 1.5 x ULN	>1.5 - 2.0 x ULN	>2.0 - 5.0 x ULN	>5.0 x ULN
**CARDIOVASCULAR**				
Cardiac Arrhythmia	__________	Asymptomatic; transient dysrhythmia, no Rx req	Recurrent/persistent dysrhythmia; symptomatic, Rx req	Unstable Dysrhythmia, hospitalization, Rx req
Hypotension	Transient orthostatic hypotension, no Rx	Symptoms correctable with oral fluid Rx	IV fluid req, no hospitalization req	Hospitalization req
Hypertension	Transient, increase >20 mm/Hg; no Rx	Recurrent; chronic increase	Acute Rx req; outpatient	Hospitalization req

090177e1815d915c\Approved\Approved On: 24-Aug-2010 20:15

ACTG GRADING SEVERITY OF ADULT ADVERSE EVENTS				
**PARAMETER**	**GRADE 1**	**GRADE 2**	**GRADE 3**	**GRADE 4**
	MILD	MODERATE	SEVERE	POTENTIALLY LIFE THREATENING
		>20 mm/Hg, Rx req	hospitalization possible	
Pericarditis	Minimal effusion	Mild/mod asymptomatic effusion, no Rx	Symptomatic effusion, pain, EKG changes	Tamponade-Pericard iocentesis OR surgery req
Hemorrhage, blood loss	________	Mildly symptomatic, no Rx required	Gross blood loss OR 1-2 units transfused	Massive blood loss OR >2 units transfused
**GASTROINTESTINAL**				
Nausea	Mild OR transient; reasonable intake maintained	Mod discomfort OR intake decreased for <3 days	Severe discomfort OR minimal intake for ≥3 days	Hospitalization req
Vomiting	Mild OR transient; 2-3 episodes per day OR mild vomiting lasting <1 week	Mod OR persistent; 4-5 episodes per day; OR vomiting lasting ≥1 week	Severe vomiting of all food/fluids in 24 hrs or orthostatic hypotension or IV Rx req	Hypotensive shock OR hospitalization req; IV Rx req
Diarrhea	Mild OR transient; 3-4 loose stools per day OR mild diarrhea lasting <1 week	Mod OR persistent; 5-7 loose stools per day OR diarrhea lasting ≥1 week	Bloody diarrhea; OR orthostatic hypotension OR >7 loose stools/day OR IV Rx required	Hypotensive shock OR hospitalization req
Oral Discomfort/ Dysphagia	Mild discomfort, no difficulty swallowing	Difficulty swallowing but able to eat and drink	Unable to swallow solids	Unable to drink fluids; IV fluids req
Constipation	Mild	Moderate	Severe	Distention with vomiting
**RESPIRATORY**				
Cough (for aerosol studies)	Transient; no Rx	Treatment associated cough; inhaled bronchodilator	Uncontrolled cough; systemic Rx req	________
Bronchospasm Acute	Transient; no Rx; FEV1 70% - <80% (or peak flow)	Rx req; normalizes with bronchodilator; FEV1 50%-<70% (or peak flow)	No normalization with bronchodilator; FEV1 25% - <50% (or peak flow), retractions	Cyanosis; FEV1 <25% (or peak flow) OR intubated
Dyspnea	Dyspnea on exertion	Dyspnea with normal activity	Dyspnea at rest	Dyspnea requiring O_2 therapy
**NEUROLOGIC**				

090177e1815d915c\Approved\Approved On: 24-Aug-2010 20:15

ACTG GRADING SEVERITY OF ADULT ADVERSE EVENTS				
**PARAMETER**	**GRADE 1**	**GRADE 2**	**GRADE 3**	**GRADE 4**
	MILD	MODERATE	SEVERE	POTENTIALLY LIFE THREATENING
Neuro-cerebellar	Slight incoordination OR dysdiadochokinesia	Intention tremor OR dysmetria OR slurred speech OR nystagmus	Ataxia requiring assistance to walk or arm incoordination interfering with ADLs	Unable to stand
Neuro-psych/mood	___________	___________	Severe mood changes requiring medical intervention	Acute psychosis req hospitalization
Paresthesia (burning, tingling, etc)	Mild discomfort; no Rx req	Mod discomfort; non-narcotic analgesia req	Severe discomfort; OR narcotic analgesia req with symptomatic improvement	Incapacitating; OR not responsive to narcotic analgesia
Neuro-motor	Mild weakness in muscle of feet but able to walk and/or mild increase or decrease in reflexes	Mod weakness in feet (unable to walk on heels and/or toes), mild weakness in hands, still able to do most hand tasks and/or loss of previously present reflex or development of hyperreflexia and/or unable to do deep knee bends due to weakness	Marked distal weakness (unable to dorsiflex toes or foot drop), and mod proximal weakness eg, in hands interfering with ADLs and/or requiring assistance to walk and/or unable to rise from chair unassisted	Confined to bed or wheel chair because of muscle weakness
Neuro-sensory	Mild impairment (dec sensation, eg, vibratory, pinprick, hot/cold in great toes) in focal area or symmetrical distribution	Mod impairment (mod dec sensation, eg, vibratory, pinprick, hot/cold to ankles) and/or joint position or mild impairment that is not symmetrical	Severe impairment (dec or loss of sensation to knees or wrists) or loss of sensation of at least mod degree in multiple different body areas (ie, upper and lower extremities)	Sensory loss involves limbs and trunk
**URINALYSIS**				
Proteinuria<br>Spot urine	1+	2 - 3+	4+	Nephrotic syndrome
24 hour urine	200 mg-1 g loss/day OR <0.3% OR <3 g/l	>1 - 2 g loss/day OR 0.3 - 1.0% OR 3 - 10 g/l	>2 - 3.5 g loss/day OR >1.0% OR >10 g/l	Nephrotic syndrome OR >3.5 g loss/day
Gross Hematuria	Microscopic only	Gross, no clots	Gross plus clots	Obstructive OR transfusion req

090177e1815d915c\Approved\Approved On: 24-Aug-2010 20:15

**ACTG GRADING SEVERITY OF ADULT ADVERSE EVENTS**				
**PARAMETER**	**GRADE 1**	**GRADE 2**	**GRADE 3**	**GRADE 4**
	MILD	MODERATE	SEVERE	POTENTIALLY LIFE THREATENING
**MISCELLANEOUS**				
Fever<br>oral >12 hours	37.7 - 38.5C OR<br>100.0 - 101.5F	38.6 - 39.5C OR<br>101.6 - 102.9F	39.6 - 40.5C OR<br>103 - 105F	>40.5C OR<br>>105F
Headache	Mild; no Rx req	Mod; or non-narcotic analgesia Rx	Severe; OR responds to initial narcotic Rx	Intractable; or req repeated narcotic Rx
Allergic Reaction	Pruritus without rash	Localized urticaria	Generalized urticaria angioedema	Anaphylaxis
Cutaneous/Rash/ Dermatitis	Erythema, pruritus	Diffuse maculopapular rash OR dry desquamation	Vesiculation OR moist desquamation OR ulceration	ANY ONE: mucous membrane involvement, suspected Stevens-Johnson (TEN), erythema multiforme, necrosis req surgery, exfoliative dermatitis
Local Reaction<br>(2° parenteral Rx - not vaccination or phlebitis skin test)	Erythema	Induration<br><10 mm OR inflammation OR phlebitis	Induration >10 mm OR ulceration	Necrosis of skin
Fatigue	Normal activity reduced <25%	Normal activity reduced 25-50%	Normal activity reduced >50%; cannot work	Unable to care for self

090177e1815d915c\Approved\Approved On: 24-Aug-2010 20:15

Appendix 5. Information Pertaining to the Extension Phase of Protocol A4001027

This appendix outlines the various components of the program as patients in A4001027 reach Week 48 and are transitioned into the Post-week 48 period. The purpose of this appendix is to provide guidance on planning patient visits and procedures.

NOTE: Please refer to Section 5.6, regarding the patient visit requirements during the post 48 week phase of the trial. During this time patients will have the following "On Study Procedures" performed.

ON STUDY PROCEDURES: AS DEFINED BY PROTOCOL SECTION 6.2.4. INCLUDE (NOTE THAT THE PK SAMPLING AND ECG TESTING ARE NOT INCLUDED):

a. Targeted physical examination and vital signs;

b. Assessment of signs, symptoms and adverse events;

c. Review of concomitant medications;

d. Serum chemistry and hematology;

e. Hepatitis C virus RNA if hepatitis C virus antibody positive at screening;

f. CD4 and CD8 lymphocyte count determinations (absolute and percent);

g. Plasma HIV-1 RNA level as determined by the Roche Amplicor HIV-1 MONITOR test, standard method (if the results of the standard method are <400 copies/mL, the ultrasensitive method will automatically be performed);

h. Blood sample for preparation of two 1 mL plasma aliquots (frozen) stored for future testing;

i. Urine pregnancy testing for WOCBP (See Section 4.1). A positive urine test will require a confirmatory serum pregnancy test;

j. Plasma sample for HIV-1 co-receptor tropism phenotype as determined by the Monogram Biosciences recombinant virus entry assay upon treatment failure as defined in the protocol, (this sample is to be drawn when the confirmatory plasma HIV-1 RNA sample is collected) if this occurs and for patients with HIV-1 RNA >500 copies/mL;

k. Plasma sample for potential HIV-1 gp160 sequencing upon treatment failure only (as defined in the protocol);

l. Review dosing compliance; and

m. Dispense Study medication using Clinphone to obtain bottle numbers.

090177e1815d915c\Approved\Approved On: 24-Aug-2010 20:15

Drug Supplies/ClinPhone: Blinded therapy will continue to be assigned by ClinPhone for the Post-week 48 period. Sites will continue to phone into the system to obtain drug assignment at each scheduled visit. Once the last patient reaches Week 48, instructions will be given concerning drug supply.

Open Label: Open label Maraviroc will continue to be an option for patients as previously defined in the protocol. The process for obtaining open label medication will not change.

Labs/Covance: Sites will be supplied "Follow Up" kits that are to be used for all visits beyond Week 48. A supply of "Follow Up" kits will be issued to sites once they have a patient reach Week 40.

n. Kits contain the blood collection tubes, which are necessary for each visit.

o. Sites need to enter the corresponding week for the visit the patient is attending for, on the Covance Accession Form eg, Week 60, 72, 84, etc.

p. Samples should be shipped to Covance in the usual manner.

q. Sites can order additional kits if they require them through Covance but re-supply will be automatic.

090177e1815d915c\Approved\Approved On: 24-Aug-2010 20:15

Case Report Forms: Data collection for the Post-week 48 visits will be captured on paper CRFs. These will be distributed based on the schedule of patients reaching Week 48. If there are any questions relating to the receipt of paper CRF, please contact your CRA.

<u>The process for transitioning from I*Net to the paper CRF is as follows:</u>

Complete I*Net up to and including Week 48 for all patients.

a. Ensure that Data Entry is marked as 'Complete' for ALL Log pages:

If an event has resolved or treatment has completed, provide the 'Date Ended' otherwise mark as 'Continuing'. Change status to 'Complete'.

If a NEW event or therapy is reported at Week 48, record on the paper CRF.

Note: There is no need to rewrite data from log pages that are continuing into the paper CRF. As Events resolve or patient stops OBT or concomitant treatment the CRA will provide a Hand Written Query (HWQ) to Pfizer.

b. Dosing: Dispensed/Returned Logs:

Complete Week 40 Log as an I*Net Page, therefore 'Date Returned' to be provided at Week 48 Visit.

Open Week 48 Log on a paper CRF.

c. Do Not Complete the Patient Disposition Page if the patient is continuing post Week 48.

d. Print and request the Principal Investigator signs the Investigator Declaration Page (page 99999) to confirm sign off of patient's data in I*Net database.

Post Week 48: Complete all Visits on a paper CRF. The first full visit is Week 60.

Complete the Investigator Declaration Page (page 99998) post Week 48 when the patient completes all required visits in the extension phase or withdraws from the extension phase of the study.

090177e1815d915c\Approved\Approved On: 24-Aug-2010 20:15

Appendix 6. Clinical Protocol Amendment #1

Current Amendment: 1

Amendment No.	Date	Country(ies)	Site(s)
1	05 May 2005	All	All

SUMMARY

This protocol is being amended in order to:

- Clarify protocol required procedures;
- Update statistical sections;
- Update data for healthy volunteer and patient studies; and
- Correct previous errors in the protocol (i.e. administrative changes).

The most significant changes are:

- Exclusion of subjects who are taking medications that should not be coadministered with efavirenz (voriconazole, terfenadine, astemizole, midazolam, triazolam, cisapride, and ergot derivatives);
- Removal of the requirement to exclude subjects who will also be taking part in observational studies;
- Upon treatment failure, in addition to a confirmatory plasma HIV-1 RNA sample, the following shall also be collected:
    a. a plasma sample for HIV-1 co-receptor tropism phenotype as determined by the ViroLogic recombinant virus entry;
    b. a plasma sample for HIV-1 genotype and phenotype as determined by the ViroLogic PhenoSense GT assay;
- Clarification of requirements for host genotyping (specifically, samples are to be collected in all subjects unless prohibited by local regulations);
- Clarification of wording in treatment failure definition;
- Clarification of wording and addition of details pertaining to the statistical methodology in the statistical sections of the protocol.

REASON(S) FOR AMENDMENT

The protocol section(s) that have been amended and the details of the changes are summarized in the following sections.

090177e1815d915c\Approved\Approved On: 24-Aug-2010 20:15

090177e1815d915c\Approved\Approved On: 24-Aug-2010 20:15

PROTOCOL SECTION(S) AMENDED

The protocol sections that were amended are detailed below. The format is as follows:

> The "change from" section represents the current text in the protocol. Bolded text is used to indicate the addition of information to the current text, and strike-out of text (eg, ~~text~~) is used to show the deletion of information from the current text.
>
> The "change to" section represents the revised text, with the revisions shown in the "change from" section in normal text.

Summary, Statistical Methods

Change From

4th sentence

An ANCOVA model will be fitted with **screening** ~~baseline~~ viral load level (randomization strata), enfuvirtide use, geographic region and treatment arm as the main effects.

Change To

An ANCOVA model will be fitted with screening viral load level (randomization strata), enfuvirtide use, geographic region and treatment arm as the main effects.

Summary, Visit Schedule

Change From

Procedures	Screening (Day – 42 to -28)	Randomization (Day –7 to –4)	Baseline[a] Day 1	Week 2 [b]	Weeks 4, 8, 12, 16, 20, 32, 40[b]	Weeks 24 and 48 or Early Termination [b,c]
Viral Resistance (Phenotype, Genotype)[i]	X				X[j]	X[o]
Co-receptor tropism (Phenotype, Genotype[k])	X		X		X[j, l]	X[o]
Host Genotyping			X[p]			

j. Upon treatment failure as defined in the protocol **(this sample is to be drawn when the confirmatory plasma HIV-1 RNA sample is collected).**

. . .

o. Except at Early Termination if a treatment failure (sample should be drawn when confirmatory HIV-1 RNA is collected)

p. Unless prohibited by local regulations

Change To

Procedures	Screening (Day – 42 to -28)	Randomization (Day –7 to –4)	Baseline[a] Day 1	Week 2 [b]	Weeks 4, 8, 12, 16, 20, 32, 40[b]	Weeks 24 and 48 or Early Termination [b,c]
Viral Resistance (Phenotype, Genotype)[i]	X				X[j]	X[o]
Co-receptor tropism (Phenotype, Genotype[k])	X		X		X[j, l]	X[o]
Host Genotyping			X[p]			

j. Upon treatment failure as defined in the protocol (this sample is to be drawn when the confirmatory plasma HIV-1 RNA sample is collected).

. . .

o. Except at Early Termination if a treatment failure (sample should be drawn when confirmatory HIV-1 RNA is collected)

p. Unless prohibited by local regulations

Section 1.1.1, Data From Healthy Volunteer Studies

Change From

To date a total of 501 healthy volunteers have received at least one dose of UK-427,857. Single oral doses up to 1200 mg and multiple oral doses up to 300 mg BID for 28 days and up to 900 mg BID and 1200 mg QD for 7 days were well tolerated.

The most common all-causality Adverse Events (AEs) reported following single and multiple UK-427,857 dosing have been asthenia, headache, dizziness, nausea, rhinitis and postural hypotension. Postural hypotension is potentially the dose limiting adverse event. The adverse event profile seen in the single and multiple dose populations were similar and adverse events did not happen significantly more on UK-427,857 than on placebo at doses up to and including 300 mg BID. No Serious Adverse Events (SAEs) were reported in the Phase 1 program and most AEs were judged to be mild to moderate in severity.

090177e1815d915c\Approved\Approved On: 24-Aug-2010 20:15

~~To date more than 400 healthy volunteers have received UK-427,857. Single oral doses up to 900 mg and multiple oral doses up to 300 mg BID for 28 days were well tolerated.~~

~~The most common Adverse Events (AEs) reported following UK-427,857 dosing have been asthenia, headache, flatulence and nausea. Postural hypotension is potentially the dose limiting adverse event. No Serious Adverse Events (SAEs) have been reported and most AEs were judged to be mild to moderate in severity.~~

In the single dose study (A4001001) four out of nine subjects who were administered a single 1200 mg dose of UK-427,857 experienced postural hypotension. Clinically significant postural hypotension caused dosing to be halted for Cohort 3 of the multiple dose study (A4001002) with 2/9 subjects on 600 mg QD and 1/3 on placebo. Dosing was repeated at 600 mg QD in an additional group of subjects (Cohort 5) and was well tolerated, with one subject experiencing mild postural hypotension. Hence the maximum tolerated multiple dose remains undefined. **Cross-protocol analysis of adverse event data from the Phase 1 studies has indicated a dose relationship for episodes of postural hypotension and dizziness. A logistic regression analysis demonstrated that the incidence of postural hypotension increased above the placebo incidence at unit doses of above 600 mg**. Single and repeated dosing with UK-427,857 had no apparent effects on supine systolic or diastolic blood pressure, although there was evidence of a treatment-related increase in supine pulse rate after a single 1200 mg dose (mean increase from baseline of 9 bpm at 1 hour post- dose). There were dose-related reductions in standing systolic blood pressure at doses of 300 mg BID and above and for standing diastolic blood pressure at 600 mg QD.

Change To

To date a total of 501 healthy volunteers have received at least one dose of UK-427,857. Single oral doses up to 1200 mg and multiple oral doses up to 300 mg BID for 28 days and up to 900 mg BID and 1200 mg QD for 7 days were well tolerated.

The most common all-causality Adverse Events (AEs) reported following single and multiple UK-427,857 dosing have been asthenia, headache, dizziness, nausea, rhinitis and postural hypotension. Postural hypotension is potentially the dose limiting adverse event. The adverse event profile seen in the single and multiple dose populations were similar and adverse events did not happen significantly more on UK-427,857 than on placebo at doses up to and including 300 mg BID. No Serious Adverse Events (SAEs) were reported in the Phase 1 program and most AEs were judged to be mild to moderate in severity

In the single dose study (A4001001) four out of nine subjects who were administered a single 1200 mg dose of UK-427,857 experienced postural hypotension. Clinically significant postural hypotension caused dosing to be halted for Cohort 3 of the multiple dose study (A4001002) with 2/9 subjects on 600 mg QD and 1/3 on placebo. Dosing was repeated at 600 mg QD in an additional group of subjects (Cohort 5) and was well tolerated, with one subject experiencing mild postural hypotension. Hence the maximum tolerated multiple dose remains undefined. Cross-protocol analysis of adverse event data from the Phase 1 studies has indicated a dose

090177e1815d915c\Approved\Approved On: 24-Aug-2010 20:15

relationship for episodes of postural hypotension and dizziness. A logistic regression analysis demonstrated that the incidence of postural hypotension increased above the placebo incidence at unit doses of above 600 mg. Single and repeated dosing with UK-427,857 had no apparent effects on supine systolic or diastolic blood pressure, although there was evidence of a treatment-related increase in supine pulse rate after a single 1200 mg dose (mean increase from baseline of 9 bpm at 1 hour post- dose). There were dose-related reductions in standing systolic blood pressure at doses of 300 mg BID and above and for standing diastolic blood pressure at 600 mg QD.

Section 1.1.2, Data From Patient Studies

Change From

4th paragraph

Further follow-up samples taken from this patient at days 203, 251, 308, 373 and 433 demonstrated that the R5X4 virus was still present in the peripheral circulation, with emergence of X4 variants over the later sampling points. Over the >1 year period since the patient received UK-427,857, his viral load has been variable but remains within the range of his pre-study values (viral load at randomization visit was 80471 copies/mL). However, there has been a gradual decline in his CD4 count to a value of 219 cells/mm^3 on Day 433. This is a significant decline from his immediate pre-study CD4 count and is also significantly lower than his lowest count (421 copies/mm^3) recorded over the past >5 years of follow-up. The precise role of R5 and X4 viruses in HIV pathogenesis is not known, but there is an hypothesis that the X4 species is responsible for faster progression to late-stage AIDS. Therefore, in light of the evolving data, the possibility that UK-427,857 selected the X4 species over R5 and that this may be causally related to the decline in CD4 cell counts cannot be excluded. The patient remained clinically well and eventually started HAART on Day 433. ~~Further follow-up samples taken from this patient at days 203, 251 and 308 demonstrated that the R5X4 virus was still present in the peripheral circulation, with emergence of X4 variants over the later sampling points. Over the >9 months period since the patient received UK-427,857 his viral load has been variable but remains within the range of his pre-study values (viral load at randomization visit was 80471 copies/ml). However, there has been a gradual decline in his CD4 count (318 cells/mm^3 on Day 308). This is an approximately 50% decline from his immediate pre-study CD4 count and is also 25% lower than his lowest count (421 cells/mm^3) recorded over the past 6 years of follow-up. While the precise role of R5 and X4 viruses in HIV pathogenesis is not known, in light of the evolving data, the possibility that UK-427,857 selected the X4 species over R5 and that this may be causally related to the decline in CD4 cell counts cannot be excluded. The patient remains clinically well and has not initiated HAART at this time.~~

090177e1815d915c\Approved\Approved On: 24-Aug-2010 20:15

Change To

Further follow-up samples taken from this patient at days 203, 251, 308, 373 and 433 demonstrated that the R5X4 virus was still present in the peripheral circulation, with emergence of X4 variants over the later sampling points. Over the >1 year period since the patient received UK-427,857, his viral load has been variable but remains within the range of his pre-study values (viral load at randomization visit was 80471 copies/mL). However, there has been a gradual decline in his CD4 count to a value of 219 cells/mm^3 on Day 433. This is a significant decline from his immediate pre-study CD4 count and is also significantly lower than his lowest count (421 copies/mm^3) recorded over the past >5 years of follow-up. The precise role of R5 and X4 viruses in HIV pathogenesis is not known, but there is an hypothesis that the X4 species is responsible for faster progression to late-stage AIDS. Therefore, in light of the evolving data, the possibility that UK-427,857 selected the X4 species over R5 and that this may be causally related to the decline in CD4 cell counts cannot be excluded. The patient remained clinically well and eventually started HAART on Day 433.

Section 3, Trial Design

Change From

1st and 2nd paragraphs

. . . **Subjects not on any antiretroviral therapy for 4 weeks ending prior to the baseline visit should remain off therapy until the baseline visit.**

Investigators will optimize therapy, with 3-6 (excluding low-dose ritonavir) branded, open-label agents, on the basis of these resistance tests, treatment history and safety/adverse event considerations. No more than one non-nucleoside reverse transcriptase inhibitor may be included in the background regimen. Changes to background therapy may only be made after the baseline visit under the following circumstances and after consultation with the medical monitor: 1) toxicity attributed to one or more background agents - a drug of the same class may be substituted; 2) documented human error in interpretation of Screening resistance test results – during the 2 week period following the baseline visit but before the Week 2 visit, one agent may be substituted or added to the background regimen without requiring the patient to be discontinued from study drug; 3) dose adjustments to existing background agents due to reason 1) above or to the addition of a required concomitant agent that results in an anticipated new drug interaction. Experimental antiretroviral agents available through pre-approval access programs or by other means may be appropriate for use as part of background therapy following approval by the medical monitor on an individualized basis. **Subjects taking efavirenz as part of their OBT should consider the addition of a PI as part of their OBT.** Mean reduction in HIV-1 RNA will be assessed at 24 and 48 weeks or at the time of discontinuation from the study. All subjects who receive at least one dose of study medication will be assessed for safety. The study will enroll over approximately a 9-month period with 48 weeks of treatment. As part of this clinical study a blood sample will be taken for non-anonymized pharmacogenetic analysis**, unless prohibited by local regulations.**

090177e1815d915c\Approved\Approved On: 24-Aug-2010 20:15

Change To

. . . Subjects not on any antiretroviral therapy for 4 weeks ending prior to the baseline visit should remain off therapy until the baseline visit.

Investigators will optimize therapy, with 3-6 (excluding low-dose ritonavir) branded, open-label agents, on the basis of these resistance tests, treatment history and safety/adverse event considerations. No more than one non-nucleoside reverse transcriptase inhibitor may be included in the background regimen. Changes to background therapy may only be made after the baseline visit under the following circumstances and after consultation with the medical monitor: 1) toxicity attributed to one or more background agents - a drug of the same class may be substituted; 2) documented human error in interpretation of Screening resistance test results – during the 2 week period following the baseline visit but before the Week 2 visit, one agent may be substituted or added to the background regimen without requiring the patient to be discontinued from study drug; 3) dose adjustments to existing background agents due to reason 1) above or to the addition of a required concomitant agent that results in an anticipated new drug interaction. Experimental antiretroviral agents available through pre-approval access programs or by other means may be appropriate for use as part of background therapy following approval by the medical monitor on an individualized basis. Subjects taking efavirenz as part of their OBT should consider the addition of a PI as part of their OBT. Mean reduction in HIV-1 RNA will be assessed at 24 and 48 weeks or at the time of discontinuation from the study. All subjects who receive at least one dose of study medication will be assessed for safety. The study will enroll over approximately a 9-month period with 48 weeks of treatment. As part of this clinical study a blood sample will be taken for non-anonymized pharmacogenetic analysis, unless prohibited by local regulations.

Section 3, Trial Design, cont.

Change From

2nd set of bullets, last bullet

Added footnote to schema

- An increase in HIV-1 RNA to ≥5,000 copies/mL ~~on two consecutive measurements~~ **on two consecutive measurements taken no more than 14 days apart,** in subjects previously confirmed to have undetectable levels of <400 copies/mL **on 2 consecutive visits**. ~~(confirmed by a second measurement taken no more than 14 days after the first measurement).~~

090177e1815d915c\Approved\Approved On: 24-Aug-2010 20:15

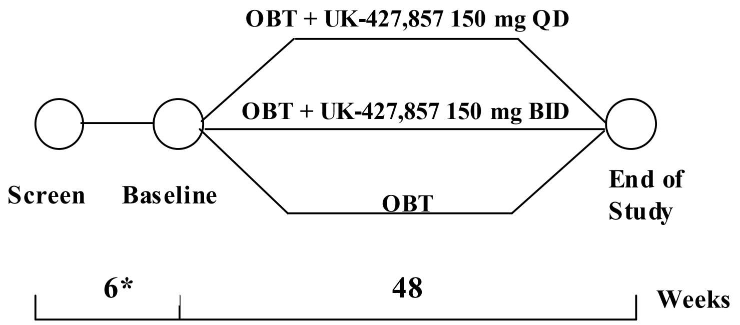

*** Subjects requiring enfuvirtide may require more time to obtain medication**

Change To

- An increase in HIV-1 RNA to ≥5,000 copies/mL on two consecutive measurements taken no more than 14 days apart, in subjects previously confirmed to have undetectable levels of <400 copies/mL on 2 consecutive visits.

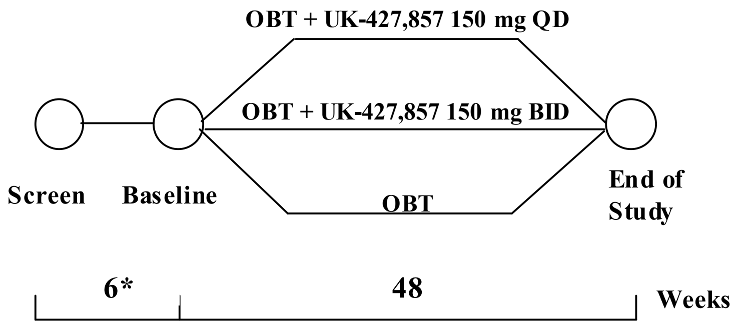

* Subjects requiring enfuvirtide may require more time to obtain medication

090177e1815d915c\Approved\Approved On: 24-Aug-2010 20:15

090177e1815d915c\Approved\Approved On: 24-Aug-2010 20:15

Section 4.2, Exclusion Criteria, Number 29

Change From

29. Any **safety, behavioral, clinical, or administrative reasons** ~~other clinical condition~~ that, in the Investigator's judgment, would potentially compromise study compliance or the ability to evaluate safety/efficacy.

Change To

29. Any safety, behavioral, clinical, or administrative reasons that, in the Investigator's judgment, would potentially compromise study compliance or the ability to evaluate safety/efficacy.

Section 5.1, Allocation to Treatment

Change From

2nd paragraph, 1st sentence

Randomization numbers will be assigned by a central web/telephone computer-based telerandomization system **(IVRS)**.

Change To

Randomization numbers will be assigned by a central web/telephone computer-based telerandomization system (IVRS).

Section 5.5, Concomitant Medications

Change From

3rd paragraph, 1st sentence in 4h paragraph, and 6th paragraph

Added sentence

Subjects taking efavirenz as part of their OBT should consider the addition of or a PI as part of their OBT.

. . .

Medications such as analgesics, anti-inflammatory agents, antibiotics and nutritional supplements **other than those listed as contraindicated below,** may be used as needed for treatment of adverse events and preexisting conditions.

. . .

As UK-427,857 is a substrate for CYP3A4 inhibition, the following agents should not be co-administered during the study period to patients receiving a 300 mg unit dose, due the risk of drug-drug interactions: ketoconazole, itraconazole, miconazole, clotrimazole, troleandomycin, nefazadone, clarithromycin, rifampin and rifabutin. The use of rifampin (rifabutin) for the treatment of a mycobacterial infection may be considered following consultation between the clinician and Pfizer medical monitor.

. . .

- **Voriconazole, terfenadine, astemizole, midazolam, triazolam, cisapride, and ergot derivatives as they should not be co-administered with efavirenz;**

Change To

Subjects taking efavirenz as part of their OBT should consider the addition of or a PI as part of their OBT.

. . .

Medications such as analgesics, anti-inflammatory agents, antibiotics and nutritional supplements other than those listed as contraindicated below**,** may be used as needed for treatment of adverse events and preexisting conditions.

. . .

As UK-427,857 is a substrate for CYP3A4 inhibition, the following agents should not be co-administered during the study period to patients receiving a 300 mg unit dose, due the risk of drug-drug interactions: ketoconazole, itraconazole, miconazole, clotrimazole, troleandomycin, nefazadone, clarithromycin, rifampin and rifabutin. The use of rifampin (rifabutin) for the treatment of a mycobacterial infection may be considered following consultation between the clinician and Pfizer medical monitor.

. . .

- Voriconazole, terfenadine, astemizole, midazolam, triazolam, cisapride, and ergot derivatives as they should not be co-administered with efavirenz;

Section 5.6, Rescue Therapy

Change From

3rd paragraph

The sponsor will provide UK-427,857, until it is commercially available, to subjects who complete 48 weeks of therapy and for whom it is medically appropriate to continue or begin therapy with UK-427,857. This protocol will be extended for a minimum of one year, following the last patient's last visit at Week 48. While the regulatory requirement in this patient

090177e1815d915c\Approved\Approved On: 24-Aug-2010 20:15

population will have been fulfilled, we intend to further evaluate the long-term safety and efficacy of UK-427,857 in antiretroviral-experienced patients. **During the extended period (minimum of one year), all subjects will be continued on double-blind therapy (via IVRS), until the last subject enrolled has completed their first 48-weeks of therapy. At which time the Sponsor will unblind the study and analyse the data.** During this time, patients will be monitored on an every 12-week schedule and the "On Study" procedures **(Section 6.2.4)** will be performed, apart from pharmacokinetic sampling.

Change To

The sponsor will provide UK-427,857, until it is commercially available, to subjects who complete 48 weeks of therapy and for whom it is medically appropriate to continue or begin therapy with UK-427,857. This protocol will be extended for a minimum of one year, following the last patient's last visit at Week 48. While the regulatory requirement in this patient population will have been fulfilled, we intend to further evaluate the long-term safety and efficacy of UK-427,857 in antiretroviral-experienced patients. During the extended period (minimum of one year), all subjects will be continued on double-blind therapy (via IVRS), until the last subject enrolled has completed their first 48-weeks of therapy. At which time the Sponsor will unblind the study and analyse the data. During this time, patients will be monitored on an every 12-week schedule and the "On Study" procedures (Section 6.2.4) will be performed, apart from pharmacokinetic sampling.

Section 6, Trial Procedures

Change From

1st bullet

- Screening: will occur within ~6 weeks prior to initial dosing with study medication. **Patients requiring additional time in obtaining their OBT may have an extension of the screening window. All deviations in the screening window will require discussion with the Pfizer medical monitor.**

Change To

- Screening: will occur within ~6 weeks prior to initial dosing with study medication. Patients requiring additional time in obtaining their OBT may have an extension of the screening window. All deviations in the screening window will require discussion with the Pfizer medical monitor.

Section 6.2.1, Randomization Visit (Day -7 to Day -4)

Change From

2nd bullet

090177e1815d915c\Approved\Approved On: 24-Aug-2010 20:15

- Selection of background regimen by the Investigator and agreement by the subject; confirmation (via FAX) to the medical monitor (sponsor)**. The OBT selection may be chosen when PSGT and gp41 drug resistance results are available, as such final selection could take place prior to the Randomization visit. Assurance** ~~and assurance~~ that the patient will have access to these medications at the Baseline/Day 1 visit (they must be brought by the patient to the investigative site at the Baseline Visit).

Change To

- Selection of background regimen by the Investigator and agreement by the subject; confirmation (via FAX) to the medical monitor (sponsor). The OBT selection may be chosen when PSGT and gp41 drug resistance results are available, as such final selection could take place prior to the Randomization visit. Assurance that the patient will have access to these medications at the Baseline/Day 1 visit (they must be brought by the patient to the investigative site at the Baseline Visit).

Section 6.2.2, Baseline (Day 1) Evaluation – *Prior to first dose*

Change From

16th bullet

- 9 mL blood sample for host genotyping **(unless prohibited by local regulations);**

Change To

- 9 mL blood sample for host genotyping (unless prohibited by local regulations);

Section 6.2.4, On Study Evaluations (Week 4, 8, 12, 16, 20, 32 and 40)

Change From

11th and 12th bullets

- Plasma sample for HIV-1 co-receptor tropism phenotype as determined by the ViroLogic recombinant virus entry assay upon treatment failure ~~(~~as defined in the protocol~~)~~**, (this sample is to be drawn when the confirmatory plasma HIV-1 RNA sample is collected)** if this occurs and for patients with HIV-1 RNA >500 copies/mL at Weeks 4, 8, 16, 32 and 40 only;
- Plasma sample for HIV-1 genotype and phenotype as determined by the ViroLogic PhenoSense GT assay upon treatment failure only **as defined in the protocol (this sample is to be drawn when the confirmatory plasma HIV-1 RNA sample is collected)** ~~(as defined in the protocol)~~;

090177e1815d915c\Approved\Approved On: 24-Aug-2010 20:15

Change To

- Plasma sample for HIV-1 co-receptor tropism phenotype as determined by the ViroLogic recombinant virus entry assay upon treatment failure as defined in the protocol, (this sample is to be drawn when the confirmatory plasma HIV-1 RNA sample is collected) if this occurs and for patients with HIV-1 RNA >500 copies/mL at Weeks 4, 8, 16, 32 and 40 only;
- Plasma sample for HIV-1 genotype and phenotype as determined by the ViroLogic PhenoSense GT assay upon treatment failure only as defined in the protocol (this sample is to be drawn when the confirmatory plasma HIV-1 RNA sample is collected);

Section 6.2.5, Week 24 and 48 or Early Termination Visit

Change From

14th and 15th bullets

- Plasma sample for HIV-1 co-receptor tropism phenotype as determined by the ViroLogic recombinant virus entry assay upon treatment failure as defined in the protocol (except at the Early Termination visit for those subjects who are treatment failures) ~~upon treatment failure (as defined in the protocol)~~ or for patients with HIV-1 RNA >500 copies/mL. Subjects that are treatment failures should have a co-receptor tropism sample drawn when the confirmatory plasma HIV-1 RNA sample is collected;
- Plasma sample for HIV-1 genotype and phenotype as determined by the ViroLogic PhenoSense GT assay upon treatment failure as defined in the protocol (except at the Early Termination visit for those subjects who are treatment failures or for patients with HIV-1 RNA >500 copies/mL. Subjects that are treatment failures) should have a PSGT sample drawn when the confirmatory plasma HIV-1 RNA sample is collected. ~~upon treatment failure (as defined in the protocol) or for patients with HIV-1 RNA >500 copies/mL~~;

Change To

- Plasma sample for HIV-1 co-receptor tropism phenotype as determined by the ViroLogic recombinant virus entry assay upon treatment failure as defined in the protocol (except at the Early Termination visit for those subjects who are treatment failures) or for patients with HIV-1 RNA >500 copies/mL. Subjects that are treatment failures should have a co-receptor tropism sample drawn when the confirmatory plasma HIV-1 RNA sample is collected;
- Plasma sample for HIV-1 genotype and phenotype as determined by the ViroLogic PhenoSense GT assay upon treatment failure as defined in the protocol (except at the Early Termination visit for those subjects who are treatment failures) or for patients

090177e1815d915c\Approved\Approved On: 24-Aug-2010 20:15

with HIV-1 RNA >500 copies/mL. Subjects that are treatment failures should have a PSGT sample drawn when the confirmatory plasma HIV-1 RNA sample is collected;

Section 6.5.2, Virus Tropism

Change From

4th and 5th bullets

- At the time of treatment failure **(to be drawn when the confirmatory plasma HIV-1 RNA sample is collected);**
- Early Termination Visit **(if not due to treatment failure)**

Change To

- At the time of treatment failure (to be drawn when the confirmatory plasma HIV-1 RNA sample is collected);
- Early Termination Visit (if not due to treatment failure)

Section 6.5.3, Viral Resistance

Change From

3rd and 4th bullets

- At the time of treatment failure **(to be drawn when the confirmatory plasma HIV-1 RNA sample is collected);**
- Early Termination Visit **(if not due to treatment failure).**

Change To

- At the time of treatment failure (to be drawn when the confirmatory plasma HIV-1 RNA sample is collected);
- Early Termination Visit (if not due to treatment failure).

Section 6.5.5, Blood Pressure, Heart Rate and ECG Monitoring

Added 4th paragraph and bullet

Supine BP and HR measurements will be recorded using a semi-automated sphygmomanometer at the following times:

- **Weeks 4, 8, 12, 16, 20, and 32.**

Section 6.5.6, Host Genotyping

Added 2nd paragraph, last sentence

090177e1815d915c\Approved\Approved On: 24-Aug-2010 20:15

Genotyping samples will be collected from all subjects unless prohibited by local regulations.

Section 6.6, Subject Withdrawal

Change From

3rd bullet and 4th sub-bullet

- Planned enrollment into another study (including, but not limited to intervention, laboratory, psychological, ~~observational, or~~ investigational drug, device, or biological studies);

. . .

An increase in HIV-1 RNA to ≥5,000 copies/mL, on two consecutive measurements taken no more than 14 days apart, in subjects previously confirmed to have undetectable levels of <400 copies/mL on 2 consecutive visits.~~An increase in HIV-1 RNA to ≥5,000 copies/mL, on two consecutive measurements, in subjects previously confirmed to have undetectable levels of <400 copies/mL (confirmed by a second measurement taken no more than 14 days after the first measurement).~~

Change To

- Planned enrollment into another study (including, but not limited to intervention, laboratory, psychological, investigational drug, device, or biological studies);

. . .

An increase in HIV-1 RNA to ≥5,000 copies/mL on two consecutive measurements taken no more than 14 days apart, in subjects previously confirmed to have undetectable levels of <400 copies/mL on 2 consecutive visits.

Section 9.2.2.2, Method of Analysis

Change From

Changes begin in 3rd paragraph

An ANCOVA model will be used with **screening** ~~baseline~~ viral load level (randomization strata), enfuvirtide use, geographic region and treatment group as the main effects. The least squares mean treatment difference between each UK-427,857 dose group and placebo will be presented. A Bonferroni adjustment will be used to account for multiple comparisons.

Interactions of **screening** ~~baseline~~ viral load level (randomization strata), enfuvirtide use and geographic region with treatment will be investigated. No main effect of treatment will be presented in the presence of an interaction term.

090177e1815d915c\Approved\Approved On: 24-Aug-2010 20:15

For the primary analysis subjects who discontinue the study before the timepoint of interest will have their final value imputed as baseline (i.e. no change from baseline). Also, no change from baseline will be imputed for subjects with missing baseline or no viral load assessment on treatment.

~~The primary analysis will include only those subjects with an assessment of viral load at baseline and while on treatment.~~ To further assess the robustness of the primary analysis ~~three~~ **two** sensitivity analyses will be performed:

> the last observation carried forward approach will be used. This analysis will include only those subjects with an assessment of viral load at baseline and while on treatment. ~~subjects with missing baseline or no viral load assessment on treatment will have no change from baseline imputed.~~
>
> subjects who discontinued before Week 24 (or 48) due to any reason, apart from protocol defined treatment failure (see Section 6.6), will have no change from baseline imputed. Treatment failures will use the last observation carried forward approach as in the ~~primary~~ **sensitivity** analysis **above**.

~~2. subjects who discontinued before Week 24 (or 48) due to any reason, including protocol defined treatment failure, will have no change from baseline imputed.~~

Change To

An ANCOVA model will be used with screening viral load level (randomization strata), enfuvirtide use, geographic region and treatment group as the main effects. The least squares mean treatment difference between each UK-427,857 dose group and placebo will be presented. A Bonferroni adjustment will be used to account for multiple comparisons.

Interactions of screening viral load level (randomization strata), enfuvirtide use and geographic region with treatment will be investigated. No main effect of treatment will be presented in the presence of an interaction term.

For the primary analysis subjects who discontinue the study before the timepoint of interest will have their final value imputed as baseline (ie, no change from baseline). Also, no change from baseline will be imputed for subjects with missing baseline or no viral load assessment on treatment.

To further assess the robustness of the primary analysis two sensitivity analyses will be performed:

> the last observation carried forward approach will be used. This analysis will include only those subjects with an assessment of viral load at baseline and while on treatment.
>
> subjects who discontinued before Week 24 (or 48) due to any reason, apart from protocol defined treatment failure (see Section 6.6), will have no change from baseline imputed.

090177e1815d915c\Approved\Approved On: 24-Aug-2010 20:15

Treatment failures will use the last observation carried forward approach as in the sensitivity analysis above.

Section 9.2.3.2, Method of Analysis

Change From

2nd bullet, 1st and 3rd paragraphs

- These variables will also be analyzed using logistic regression including **screening** ~~baseline~~ viral load level (<100,000 versus ≥100,000 copies/mL) (randomization strata), enfuvirtide use and geographic region (Northern Hemisphere/Southern Hemisphere) as factors.

TAD in $\log_{10}$ HIV-1 RNA, change from baseline in CD4 and CD8 cell count will be analyzed using an ANCOVA model with **screening** ~~baseline~~ viral load level, CD4 or CD8 cell count respectively, enfuvirtide use, geographic region and treatment arm as the main effects.

. . .

Day 1 for this analysis is defined as the first **day of dosing.** ~~occasion on which a subject had confirmed HIV-1 RNA levels below 400 copies/mL, i.e., on the first of 2 consecutive visits.~~ Time to virological failure is time from Day 1 until virological failure as defined below.

Change To

- These variables will also be analyzed using logistic regression including screening viral load level (<100,000 versus ≥100,000 copies/mL) (randomization strata), enfuvirtide use and geographic region (Northern Hemisphere/Southern Hemisphere) as factors.

TAD in $\log_{10}$ HIV-1 RNA, change from baseline in CD4 and CD8 cell count will be analyzed using an ANCOVA model with screening viral load level, CD4 or CD8 cell count respectively, enfuvirtide use, geographic region and treatment arm as the main effects.

. . .

Day 1 for this analysis is defined as the first day of dosing. Time to virological failure is time from Day 1 until virological failure as defined below.

090177e1815d915c\Approved\Approved On: 24-Aug-2010 20:15

Section 9.5, Interim Analysis

Change From

1st paragraph

No formal interim analysis will be performed. However, a DSMB will review the first 100 patients treated through 8 weeks to confirm whether this study should continue. Recruitment will continue during the DSMB review of the first 100 patients unless the overall treatment failure (ie, subjects who meet one of the prespecified stopping rules) rate at Week 8 exceeds 21% in studies A4001027 and A4001028 combined. This is based on the overall failure rates for TORO-1 and TORO-2 at week 8.[1,2] **However, recruitment may be allowed to continue during this time period, despite exceeding a 21% failure rate, following an ad-hoc review of the data by the DSMB and its recommendation to continue recruitment.**

Change To

No formal interim analysis will be performed. However, a DSMB will review the first 100 patients treated through 8 weeks to confirm whether this study should continue. Recruitment will continue during the DSMB review of the first 100 patients unless the overall treatment failure (ie, subjects who meet one of the prespecified stopping rules) rate at Week 8 exceeds 21% in studies A4001027 and A4001028 combined. This is based on the overall failure rates for TORO-1 and TORO-2 at week 8.[1,2] **However, recruitment may be allowed to continue during this time period, despite exceeding a 21% failure rate, following an ad-hoc review of the data by the DSMB and its recommendation to continue recruitment.**

090177e1815d915c\Approved\Approved On: 24-Aug-2010 20:15

Appendix 7. Clinical Protocol Amendment #2

Current Amendment: 2

Amendment No.	Date	Country(ies)	Site(s)
2	20 December 2005	All	All

Previous Amendments:

Amendment No.	Date	Country(ies)	Site(s)
1	05 May 2005	All	All

SUMMARY of significant changes

- Increase of study population size to 600 patients
- Addition of blood sample collection at the Randomization visit
- Clarification of algorithm in Appendix 2 (liver enzyme work-up)
- Exclusion of isoniazid use
- Exclusion of the initiation of potentially myelosuppressive, neurotoxic, hepatotoxic and/or cytotoxic agents within 60 days prior to randomization
- Removal of specific exclusion criteria pertaining to history of cardiovascular and cerebrovascular disease
- Addition of pharmacokinect study results with efavirenz
- Clarification of text in Section 5.6 (Rescue Therapy)
- Correct previous errors in the protocol (ie, concomitant medication, administrative edits)
- Addition of treatment failure rule for patients receiving open-label UK-427,857
- Addition of Appendix 5 to outline requirements for the extension phase of the study

REASON(S) FOR AMENDMENT

The protocol section(s) that have been amended and the details of the changes are summarized in the following sections.

PROTOCOL SECTION(S) AMENDED

The protocol sections that were amended are detailed below. The format is as follows:

- The "change from" section represents the current text in the protocol. Bolded text is used to indicate the addition of information to the current text, and strike-out of text (eg, text) is used to show the deletion of information from the current text.

090177e1815d915c\Approved\Approved On: 24-Aug-2010 20:15

- The "change to" section represents the revised text, with the revisions shown in the "change from" section in normal text.

Summary; Trial Design

Change From

1st paragraph, last sentence

. . . This study will involve approximately 90 centers to achieve a total randomized subject population of **600** ~~500~~ subjects.

Change To

. . . This study will involve approximately 90 centers to achieve a total randomized subject population of 600 subjects.

Summary; Visit Schedule

Change From

**Procedures**	**Screening (Day – 42 to -28)**	**Randomization (Day –7 to –4)**	**Baseline[a] Day 1**	**Week 2[b]**	**Weeks 4, 8, 12, 16, 20, 32, 40[b]**	**Weeks 24 and 48 or Early Termination[b,c]**
Chemistry, Hematology	X	**X[q]**	X	X	X	X

Footnote 'q' added

q. Chemistry only

Change To

**Procedures**	**Screening (Day – 42 to -28)**	**Randomization (Day –7 to –4)**	**Baseline[a] Day 1**	**Week 2[b]**	**Weeks 4, 8, 12, 16, 20, 32, 40[b]**	**Weeks 24 and 48 or Early Termination[b,c]**
Chemistry, Hematology	X	X[q]	X	X	X	X

Footnote

q.Chemistry only

Section 3; Trial Design

090177e1815d915c\Approved\Approved On: 24-Aug-2010 20:15

Change From

1st paragraph, 2nd sentence

. . . This study is planned to enroll **600** ~~500~~ subjects. . .

Change To

. . . This study is planned to enroll 600 subjects. . .

Section 4.1; Inclusion Criteria

Change From

Criterion #5

5. Documented genotypic (http://iasusa.org/resistance_mutations/index.html as determined by the ~~ViroLogic~~**Monogram Biosciences** GeneSeq HIV drug resistance assay) or phenotypic (fold change of patient's virus ≥ cutoff value based on ~~ViroLogic~~**Monogram Biosciences (formerly Virologic)** PhenoSense HIV drug resistance assay) resistance to three of the four antiretroviral drug classes [Nucleoside or nucleotide reverse transcriptase inhibitors (nRTIs), Non-nucleoside reverse transcriptase inhibitors (NNRTIs), Protease Inhibitors (PIs) or Entry Inhibitors (EIs)]

Change To

5. Documented genotypic (http://iasusa.org/resistance_mutations/index.html as determined by the Monogram Biosciences GeneSeq HIV drug resistance assay) or phenotypic (fold change of patient's virus ≥ cutoff value based on Monogram Biosciences (formerly Virologic) PhenoSense HIV drug resistance assay) resistance to three of the four antiretroviral drug classes [Nucleoside or nucleotide reverse transcriptase inhibitors (nRTIs), Non-nucleoside reverse transcriptase inhibitors (NNRTIs), Protease Inhibitors (PIs) or Entry Inhibitors (EIs)]

Section 4.2; Exclusion Criteria

Change From

Criterions #8, 11, 12, and 19-25

8. **Initiating** ~~Previous~~ therapy with a potentially myelosuppressive, neurotoxic, hepatotoxic and/or cytotoxic agent within **60** ~~30~~ days prior to randomization, or the expected need for such therapy during the study period. **NOTE: Trimethoprim-sulfamethoxazole may not be initiated 60 days prior to randomization but may be continued if the subject is on stable therapy. The use of isoniazid is prohibited. . .**

11. Total bilirubin, greater than **2**.5 times the upper limit of normal (unless unconjugated hyperbilirubinemia due to atazanavir or indinavir)**. Changes of one or more grades in total bilirubin between screening and the randomization visit should be reviewed with the Pfizer medical monitor before the initiation of double-blind study medication.**

090177e1815d915c\Approved\Approved On: 24-Aug-2010 20:15

12. AST ~~and/~~or ALT greater than 5 times the upper limit of normal. **Changes of one or more grades in AST or ALT between screening and the randomization visit should be reviewed with the Pfizer medical monitor before the initiation of double-blind study medication.**

. . .

~~19. Myocardial infarction, symptomatic angina or intermittent claudication within the previous 6 months.~~

~~20. Angiographically documented three-vessel coronary artery disease or left main stem disease.~~

~~21. Angiographically documented stenosis of >90% in the left anterior descending artery or a dominant right coronary or circumflex artery.~~

~~22. Congestive heart failure New York Heart Association Class III/IV.~~

~~23. History of a transient ischemic attack or cerebrovascular accident.~~

~~24. Documented carotid artery lesion of >70% or a carotid bruit (unless proven by ultrasound not to be due to carotid vascular disease).~~

~~25. Symptomatic postural hypotension (ie, systolic BP drop >20 mm Hg, diastolic BP drop >10 mm Hg and/or drop in systolic BP to <90 mm Hg) or postural hypotension (with or without symptoms) in a patient with hypertension being treated with one or more antihypertensive agents.~~

Change To

8. Initiating therapy with a potentially myelosuppressive, neurotoxic, hepatotoxic and/or cytotoxic agent within 60 days prior to randomization, or the expected need for such therapy during the study period. NOTE: Trimethoprim-sulfamethoxazole **may not** be initiated 60 days prior to randomization but may be continued if the subject is on stable therapy. The use of isoniazid is prohibited. . .

11. Total bilirubin, greater than 2.5 times the upper limit of normal (unless unconjugated hyperbilirubinemia due to atazanavir or indinavir). Changes of one or more grades in total bilirubin between screening and the randomization visit should be reviewed with the Pfizer medical monitor before the initiation of double-blind study medication.

12. AST or ALT greater than 5 times the upper limit of normal. Changes of one or more grades in AST or ALT between screening and the randomization visit should be reviewed with the Pfizer medical monitor before the initiation of double-blind study medication.

. . .

Criterion #19 thru 25 deleted; following criterions renumbered accordingly.

Section 5.5; Concomitant Medication(s)

090177e1815d915c\Approved\Approved On: 24-Aug-2010 20:15

Change From

4th paragraph

Subjects taking efavirenz as part of their OBT should consider the addition of a PI as part of their OBT. **In a pharmacokinetic study (A4001011), the mean reduction in UK-427,857 AUC_τ and C_{max} was approximately 50% in the presence of efavirenz. Increasing the UK-427,857 dose compensated for this reduction. As demonstrated in a follow-up pharmacokinetic study (A4001021), the effect of this induction due to efavirenz was similar in the presence and absence of a CYP3A4 inhibitor (Kaletra™, boosted saquinavir), resulting in an approximate 50% reduction in C_{max} and AUC_τ from that seen in the presence of the inhibitor alone. However, in this study, which evaluated the effects of combinations of metabolic inducers and inhibitors on the pharmacokinetics of UK-427,857, the net effect was that of inhibition.**

. . .

1st bullet

- ~~voriconazole, terfenadine, astemizole, midazolam, triazolam, cisapride, and ergot derivatives as they should not be co-administered with efavirenz;~~

Last 2 bullets added

- **isoniazid;**
- **use of tipranavir as part of OBT is not allowed at the time of this amendment (amendment 2) per the requirements of the DSMB. The investigators will be notified via a letter, if the DSMB removes this restriction. The recommended dose of maraviroc is 300 mg unit dose (either once or twice daily depending on the treatment arm to which the patient is randomized) when tipranavir/ritonavir is included as part of optimized background therapy.**

090177e1815d915c\Approved\Approved On: 24-Aug-2010 20:15

Change To

4th paragraph

Subjects taking efavirenz as part of their OBT should consider the addition of a PI as part of their OBT. In a pharmacokinetic study (A4001011), the mean reduction in UK-427,857 AUC_τ and C_{max} was approximately 50% in the presence of efavirenz. Increasing the UK-427,857 dose compensated for this reduction. As demonstrated in a follow-up pharmacokinetic study (A4001021), the effect of this induction due to efavirenz was similar in the presence and absence of a CYP3A4 inhibitor (Kaletra™, boosted saquinavir), resulting in an approximate 50% reduction in C_{max} and AUC_τ from that seen in the presence of the inhibitor alone. However, in this study, which evaluated the effects of combinations of metabolic inducers and inhibitors on the pharmacokinetics of UK-427,857, the net effect was that of inhibition.

. . .

1st bullet deleted

Last 2 bullets

- isoniazid;
- use of tipranavir as part of OBT is not allowed at the time of this amendment (amendment 2) per the requirements of the DSMB. The investigators will be notified via a letter, if the DSMB removes this restriction. The recommended dose of maraviroc is 300 mg unit dose (either once or twice daily depending on the treatment arm to which the patient is randomized) when tipranavir/ritonavir is included as part of optimized background therapy.

Section 5.6; Rescue Therapy

Change From

1st paragraph

If a patient meets the criteria for treatment failure or discontinues for another reason (eg, pregnancy, adverse event) and requires an alternative regimen, they will be followed until the Week 48 visit according to protocol guidelines. The new regimen, selected by the Investigator based on the results of resistance testing at the time of failure, must be recorded in the CRF. For patients whose virus still remains CCR5-tropic and potentially sensitive to UK-427,857, **open-label** study drug may be continued during this period. The Investigator, in consultation with the medical monitor, will evaluate the appropriateness of continued therapy with **open-label** UK-427,857 based on ongoing review of clinical and laboratory parameters. **Patients that receive open-label UK-427,857 will follow the protocol designed visit schedule and procedures. If a patients viral load increases beyond three times the value at the early termination visit, upon confirmation of such value, the patient will be considered a**

090177e1815d915c\Approved\Approved On: 24-Aug-2010 20:15

treatment failure during the open-label dosing phase. The primary investigator will discontinue open label drug following consultation with the Pfizer medical monitor.

Last paragraph; last sentence

. . . During this time, patients will be monitored on an every 12-week schedule and the "On Study" procedures (Section 6.2.4) will be performed, apart from pharmacokinetic sampling **(see** Appendix 5**)**.

Change To

1st paragraph

If a patient meets the criteria for treatment failure or discontinues for another reason (eg, pregnancy, adverse event) and requires an alternative regimen, they will be followed until the Week 48 visit according to protocol guidelines. The new regimen, selected by the Investigator based on the results of resistance testing at the time of failure, must be recorded in the CRF. For patients whose virus still remains CCR5-tropic and potentially sensitive to UK-427,857, open-label study drug may be continued during this period. The Investigator, in consultation with the medical monitor, will evaluate the appropriateness of continued therapy with open-label UK-427,857 based on ongoing review of clinical and laboratory parameters. Patients that receive open-label UK-427,857 will follow the protocol designed visit schedule and procedures. If a patients viral load increases beyond three times the value at the early termination visit, upon confirmation of such value, the patient will be considered a treatment failure during the open-label dosing phase. The primary investigator will discontinue open label drug following consultation with the Pfizer medical monitor.

Last paragraph; last sentence

. . . During this time, patients will be monitored on an every 12-week schedule and the "On Study" procedures (Section 6.2.4) will be performed, apart from pharmacokinetic sampling (see Appendix 5).

Section 6.1; Screening Visit (Day –42 to Day -28)

Change From

8th and 9th bullets

- Plasma sample for HIV-1 co-receptor tropism phenotype as determined by the ~~ViroLogic~~**Monogram Biosciences** recombinant virus entry assay;
- Plasma sample for HIV-1 genotype and phenotype as determined by the ViroLogicMonogram Biosciences PhenoSense™ GT assay; and

090177e1815d915c\Approved\Approved On: 24-Aug-2010 20:15

Change To

- Plasma sample for HIV-1 co-receptor tropism phenotype as determined by the Monogram Biosciences recombinant virus entry assay;
- Plasma sample for HIV-1 genotype and phenotype as determined by the Monogram Biosciences PhenoSense™ GT assay; and

Section 6.2.1; Randomization Visit (Day -7 to Day -4)

Change From

1st bullet added

Change To

- Serum chemistry (review of Inclusion/Exclusion criteria)

Section 6.2.2; Baseline (Day 1) Evaluation – *Prior to first dose*

Change From

12th bullet

- Plasma sample for HIV-1 co-receptor tropism phenotype as determined by the ~~ViroLogic~~**Monogram Biosciences** recombinant virus entry assay;

Change To

- Plasma sample for HIV-1 co-receptor tropism phenotype as determined by the Monogram Biosciences recombinant virus entry assay;

Section 6.2.4; On Study Evaluations (Week 4, 8, 12, 16, 20, 32 and 40)

Change From

11th and 12th bullets

- Plasma sample for HIV-1 co-receptor tropism phenotype as determined by the ~~ViroLogic~~**Monogram Biosciences** recombinant virus entry assay upon treatment failure as defined in the protocol, (this sample is to be drawn when the confirmatory plasma HIV-1 RNA sample is collected) if this occurs and for patients with HIV-1 RNA >500 copies/mL at Weeks 4, 8, 16, 32 and 40 only;
- Plasma sample for HIV-1 genotype and phenotype as determined by the ~~ViroLogic~~**Monogram Biosciences** PhenoSense GT assay upon treatment failure only as defined in the protocol (this sample is to be drawn when the confirmatory plasma HIV-1 RNA sample is collected);

Change To

090177e1815d915c\Approved\Approved On: 24-Aug-2010 20:15

- Plasma sample for HIV-1 co-receptor tropism phenotype as determined by the Monogram Biosciences recombinant virus entry assay upon treatment failure as defined in the protocol, (this sample is to be drawn when the confirmatory plasma HIV-1 RNA sample is collected) if this occurs and for patients with HIV-1 RNA >500 copies/mL at Weeks 4, 8, 16, 32 and 40 only;
- Plasma sample for HIV-1 genotype and phenotype as determined by the Monogram Biosciences PhenoSense GT assay upon treatment failure only as defined in the protocol (this sample is to be drawn when the confirmatory plasma HIV-1 RNA sample is collected);

Section 6.2.5; Week 24 and 48 or Early Termination Visit

Change From

14th and 15 bullets

- Plasma sample for HIV-1 co-receptor tropism phenotype as determined by the ~~ViroLogic~~**Monogram Biosciences** recombinant virus entry assay upon treatment failure as defined in the protocol (except at the Early Termination visit for those subjects who are treatment failures) or for patients with HIV-1 RNA >500 copies/mL. Subjects that are treatment failures should have a co-receptor tropism sample drawn when the confirmatory plasma HIV-1 RNA sample is collected;
- Plasma sample for HIV-1 genotype and phenotype as determined by the ~~ViroLogic~~**Monogram Biosciences** PhenoSense GT assay upon treatment failure as defined in the protocol (except at the Early Termination visit for those subjects who are treatment failures) or for patients with HIV-1 RNA >500 copies/mL. Subjects that are treatment failures should have a PSGT sample drawn when the confirmatory plasma HIV-1 RNA sample is collected;

Change To

- Plasma sample for HIV-1 co-receptor tropism phenotype as determined by the Monogram Biosciences recombinant virus entry assay upon treatment failure as defined in the protocol (except at the Early Termination visit for those subjects who are treatment failures) or for patients with HIV-1 RNA >500 copies/mL. Subjects that are treatment failures should have a co-receptor tropism sample drawn when the confirmatory plasma HIV-1 RNA sample is collected;
- Plasma sample for HIV-1 genotype and phenotype as determined by the Monogram Biosciences PhenoSense GT assay upon treatment failure as defined in the protocol (except at the Early Termination visit for those subjects who are treatment failures) or for patients with HIV-1 RNA >500 copies/mL. Subjects that are treatment failures should have a PSGT sample drawn when the confirmatory plasma HIV-1 RNA sample is collected;

Section 6.3; Population Pharmacokinetics

090177e1815d915c\Approved\Approved On: 24-Aug-2010 20:15

Change From

Last paragraph added

Change To

For subjects who have been discontinued from the study and remain in-study, off drug, samples for pharmacokinetic testing are not required after the early termination visit is completed.

Section 6.5.2; Virus Tropism

Change From

1st paragraph, 1st sentence

Virus tropism will be determined using the ~~ViroLogic~~**Monogram Biosciences** PhenoSense™ Entry Assay. . .

Change To

Virus tropism will be determined using the Monogram Biosciences PhenoSense™ Entry Assay. .

Section 6.5.3; Viral Resistance

Change From

1st paragraph, 1st sentence

Phenotypic and genotypic resistance to PIs, NRTIs and NNRTIs will be evaluated using the ~~ViroLogic~~**Monogram Biosciences** PhenoSense™ GT assay. . .

Change To

Phenotypic and genotypic resistance to PIs, NRTIs and NNRTIs will be evaluated using the Monogram Biosciences PhenoSense™ GT assay. . .

Section 6.5.5; Blood Pressure, Heart Rate and ECG Monitoring

Change From

3rd paragraph

Patients with ~~symptomatic~~ postural hypotension at the ~~screening or~~ baseline visits will be ~~excluded until such time as this has been appropriately managed. Patients with asymptomatic postural hypotension at the baseline visit will be~~ monitored for 4 hours following the first dose of study drug and any adverse events recorded. Any adverse events felt potentially related to

090177e1815d915c\Approved\Approved On: 24-Aug-2010 20:15

postural hypotension at this visit should be discussed immediately with the medial monitor or designee.

Change To

Patients with postural hypotension at the baseline visit will be monitored for 4 hours following the first dose of study drug and any adverse events recorded. Any adverse events felt potentially related to postural hypotension at this visit should be discussed immediately with the medial monitor or designee.

Section 7.2; Safety Assessments

Change From

4th paragraph, 1st sentence

If a subject develops a Grade 3 abnormality (with the exception of hypercholesterolemia, hypertriglyceridemia, asymptomatic CPK elevations, AST/ALT in the absence of a total bilirubin elevation >**2.**5 times ULN, anemia, glucose or asymptomatic amylase or lipase elevations) or a Grade 4 laboratory abnormality (with the exception of hypercholesterolemia and hypertriglyceridemia), the Investigator will immediately discuss the case with the medical monitor. . .

Change To

If a subject develops a Grade 3 abnormality (with the exception of hypercholesterolemia, hypertriglyceridemia, asymptomatic CPK elevations, AST/ALT in the absence of a total bilirubin elevation >2.5 times ULN, anemia, glucose or asymptomatic amylase or lipase elevations) or a Grade 4 laboratory abnormality (with the exception of hypercholesterolemia and hypertriglyceridemia), the Investigator will immediately discuss the case with the medical monitor. . .

Section 8.3; Abnormal Laboratory Findings

Change From

1st paragraph split

A designated central laboratory will perform laboratory safety tests. Any screening laboratory result outside the inclusion or exclusion criteria may be repeated. If repeat screening results remain outside the eligibility criteria, the subject should not be enrolled unless there is prior written agreement between the sponsor and the Investigator to allow an exemption from the inclusion or exclusion criteria.

Changes of one or more grades in total bilirubin between screening and the randomization visit (including the Randomization visit results) should be reviewed with the Pfizer medical monitor

090177e1815d915c\Approved\Approved On: 24-Aug-2010 20:15

before the initiation of double-blind study medication. Laboratory safety tests will be performed at the following time points: *(See Study Schedule/Flowchart).*

3rd bullet

- Day 1 **(Baseline)** Pre-dose

Change To

1st paragraph

A designated central laboratory will perform laboratory safety tests. Any screening laboratory result outside the inclusion or exclusion criteria may be repeated. If repeat screening results remain outside the eligibility criteria, the subject should not be enrolled unless there is prior written agreement between the sponsor and the Investigator to allow an exemption from the inclusion or exclusion criteria.

Changes of one or more grades in total bilirubin between screening and the randomization visit (including the Randomization visit results) should be reviewed with the Pfizer medical monitor before the initiation of double-blind study medication. Laboratory safety tests will be performed at the following time points: *(See Study Schedule/Flowchart.)*

3rd bullet

- Day 1 (Baseline) Pre-dose

Section 8.3.2; Follow Up of Laboratory Test Abnormalities

Change From

1st paragraph, 2nd sentence

. . . If a subject develops a Grade 3 abnormality (with the exception of hypercholesterolemia, hypertriglyceridemia, asymptomatic CPK elevations, AST/ALT in the absence of a total bilirubin elevation >~~5~~ **2.5** times ULN, anemia, glucose or asymptomatic amylase or lipase elevations) or a Grade 4 laboratory abnormality (with the exception of hypercholesterolemia and hypertriglyceridemia), the Investigator will immediately discuss the case with the medical monitor. . .

090177e1815d915c\Approved\Approved On: 24-Aug-2010 20:15

Change To

. . . If a subject develops a Grade 3 abnormality (with the exception of hypercholesterolemia, hypertriglyceridemia, asymptomatic CPK elevations, AST/ALT in the absence of a total bilirubin elevation >2.5 times ULN, anemia, glucose or asymptomatic amylase or lipase elevations) or a Grade 4 laboratory abnormality (with the exception of hypercholesterolemia and hypertriglyceridemia), the Investigator will immediately discuss the case with the medical monitor. . .

Section 9.1; Sample Size Determination

Change From

1st paragraph, 1st sentence

A total of **600** ~~500~~ subjects will be randomized in a 2:2:1 ratio (**240** ~~200~~ on UK-427,857 150 mg QD, **240** ~~200~~ on UK-427,857 150 mg BID and **120** ~~100~~ on placebo). . .

Change To

A total of 600 subjects will be randomized in a 2:2:1 ratio (240 on UK-427,857 150 mg QD, 240 on UK-427,857 150 mg BID and 120 on placebo). . .

Appendix 1.; AIDS-Defining Opportunistic Illnesses (OIS)

Change From

V. Neoplastic Diseases

A Kaposi's Sarcoma

A501 Kaposi's sarcoma,
mucocutaneous, proven by
histology or cytology

A502 Kaposi's sarcoma,
mucocutaneous, presumptive

A503 Kaposi's sarcoma, visceral

. . .

VIII. Sentinel infections

1 Pneumococcus

2 Streptococcus

090177e1815d915c\Approved\Approved On: 24-Aug-2010 20:15

3 Staphylococcus

4 Enterococcus

5 Meningococcus

6 Mycoplasma

7 Chlamydia

8 Herpes virus

9 Cryptococcus

10 Candidiasis

11 Listeria

12 Haemophilus

13 Enteric bacteria

14 Pseudomonas

15 Legionella

16 Aspergillus

17 Protozoa

18 Helminth

19 Viral hepatitis

20 Other parasitic

21 Clostridia

22 Atypical mycobacteria

23 Mycobacteria

24 Spirochetal infections

25 Rickettsia

997 Other bacterial

090177e1815d915c\Approved\Approved On: 24-Aug-2010 20:15

998 Other viral

999 Other fungal

Change To

V. Neoplastic Diseases

A Kaposi's Sarcoma

A501 Kaposi's sarcoma, mucocutaneous, proven by histology or cytology

A502 Kaposi's sarcoma, mucocutaneous, presumptive

A503 Kaposi's sarcoma, visceral

. . .

VIII. Sentinel infections

1 Pneumococcus

2 Streptococcus

3 Staphylococcus

4 Enterococcus

5 Meningococcus

6 Mycoplasma

7 Chlamydia

8 Herpes virus

9 Cryptococcus

10 Candidiasis

11 Listeria

12 Haemophilus

13 Enteric bacteria

090177e1815d915c\Approved\Approved On: 24-Aug-2010 20:15

14 Pseudomonas

15 Legionella

16 Aspergillus

17 Protozoa

18 Helminth

19 Viral hepatitis

20 Other parasitic

21 Clostridia

22 Atypical mycobacteria

23 Mycobacteria

24 Spirochetal infections

25 Rickettsia

997 Other bacterial

998 Other viral

999 Other fungal

090177e1815d915c\Approved\Approved On: 24-Aug-2010 20:15

Appendix 2; Liver Enzyme Work-up

Change From

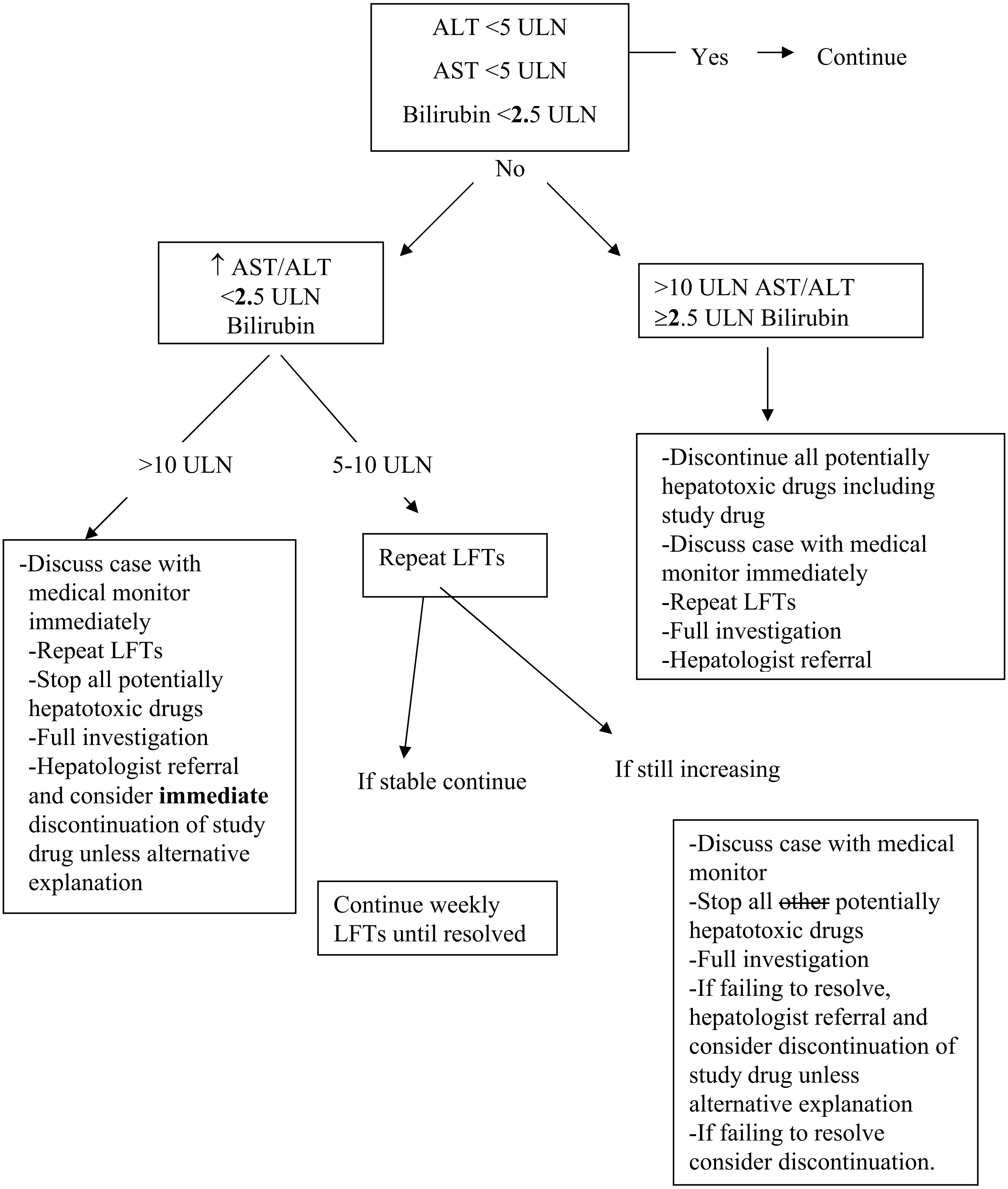

090177e1815d915c\Approved\Approved On: 24-Aug-2010 20:15

Change To

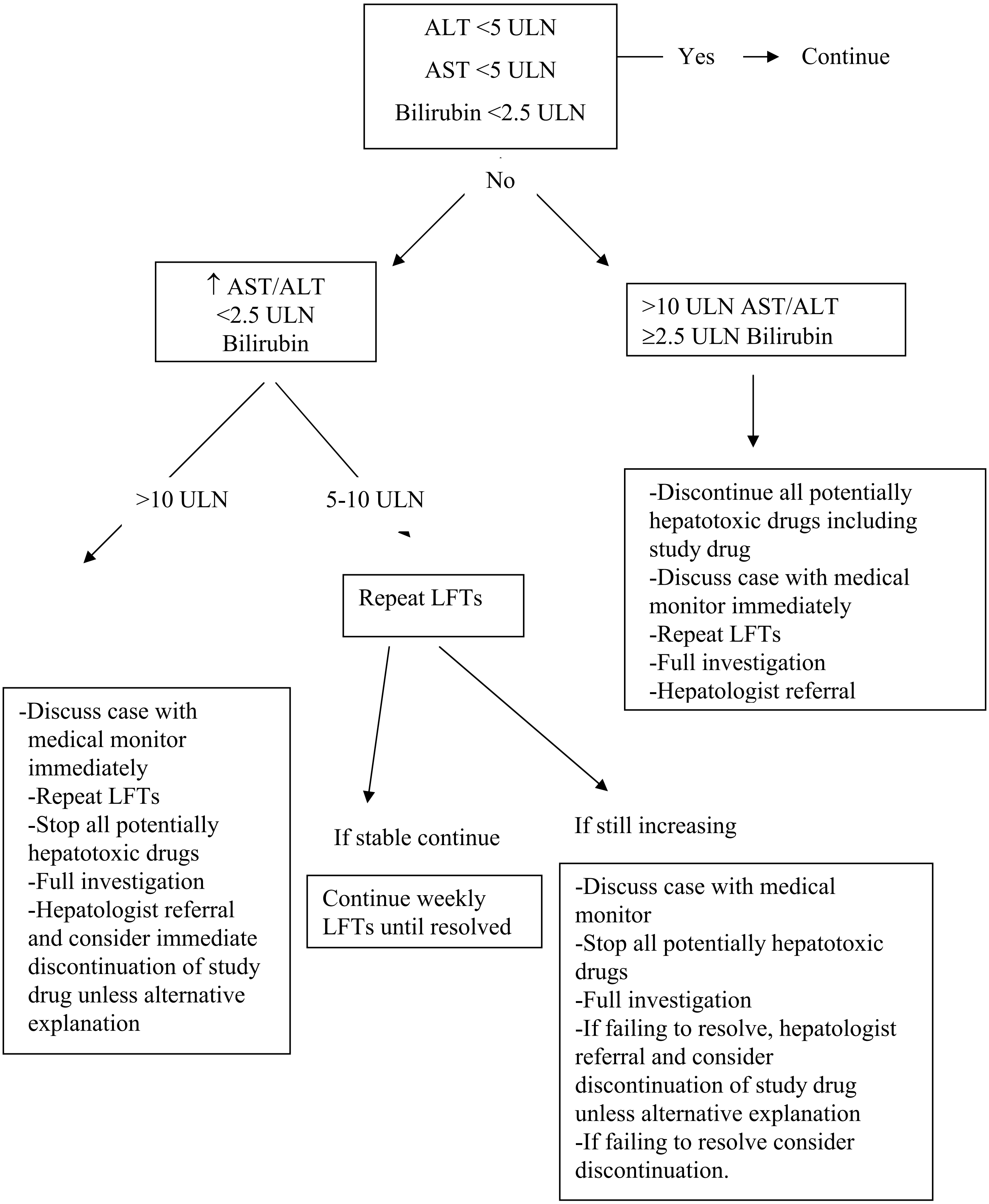

090177e1815d915c\Approved\Approved On: 24-Aug-2010 20:15

Appendix 5; Information pertaining to the Extension Phase of Protocol A4001027

Change From

Entire Appendix 5 added

Change To

This appendix outlines the various components of the program as patients in A4001027 reach Week 48 and are transitioned into the Post-week 48 period. The purpose of this appendix is to provide guidance on planning patient visits and procedures.

NOTE: Please refer to Section 5.6, regarding the patient visit requirements during the post 48 week phase of the trial. During this time patients will have the following "On Study Procedures" performed.

ON STUDY PROCEDURES: AS DEFINED BY PROTOCOL SECTION 6.2.4. INCLUDE (NOTE THAT THE PK SAMPLING AND ECG TESTING ARE NOT INCLUDED):

- Targeted physical examination and vital signs;
- Assessment of signs, symptoms and adverse events;
- Review of concomitant medications;
- Serum chemistry and hematology;
- Hepatitis C virus RNA if hepatitis C virus antibody positive at screening;
- CD4 and CD8 lymphocyte count determinations (absolute and percent);
- Plasma HIV-1 RNA level as determined by the Roche Amplicor HIV-1 MONITOR test, standard method (if the results of the standard method are <400 copies/mL, the ultrasensitive method will automatically be performed);
- Blood sample for preparation of two 1 mL plasma aliquots (frozen) stored for future testing;
- Urine pregnancy testing for WOCBP (see Section 4.1). A positive urine test will require a confirmatory a serum pregnancy test;
- Plasma sample for HIV-1 co-receptor tropism phenotype as determined by the Monogram Biosciences recombinant virus entry assay upon treatment failure as defined in the protocol, (this sample is to be drawn when the confirmatory plasma HIV-1 RNA sample is collected) if this occurs and for patients with HIV-1 RNA >500 copies/mL;
- Plasma sample for potential HIV-1 gp160 sequencing upon treatment failure only (as defined in the protocol);
- Review dosing compliance; and
- Dispense Study medication using Clinphone to obtain bottle numbers.

090177e1815d915c\Approved\Approved On: 24-Aug-2010 20:15

Drug Supplies/ClinPhone: Blinded therapy will continue to be assigned by ClinPhone for the Post-week 48 period. Sites will continue to phone into the system to obtain drug assignment at each scheduled visit. Once the last patient reaches Week 48, instructions will be given concerning drug supply.

Open Label: Open label Maraviroc will continue to be an option for patients as previously defined in the protocol. The process for obtaining open label medication will not change.

Labs/Covance: Sites will be supplied "Follow Up" kits that are to be used for all visits beyond Week 48. A supply of "Follow Up" kits will be issued to sites once they have a patient reach Week 40.

- Kits contain the blood collection tubes, which are necessary for each visit.
- Sites need to enter the corresponding week for the visit the patient is attending for, on the Covance Accession Form eg, Week 60, 72, 84, etc.
- Samples should be shipped to Covance in the usual manner.
- Sites can order additional kits if they require them through Covance but re-supply will be automatic.

Case Report Forms: Data collection for the Post-week 48 visits will be captured on paper CRFs. These will be distributed based on the schedule of patients reaching Week 48. If there are any questions relating to the receipt of paper CRF, please contact your CRA.

<u>The process for transitioning from I*Net to the paper CRF is as follows:</u>

Complete I*Net up to and including Week 48 for all patients.

- Ensure that Data Entry is marked as 'Complete' for ALL Log pages:

If an event has resolved or treatment has completed, provide the 'Date Ended' otherwise mark as 'Continuing'. Change status to 'Complete'.

If a NEW event or therapy is reported at Week 48, record on the paper CRF.

Note: There is no need to rewrite data from log pages that are continuing into the paper CRF. As Events resolve or patient stops OBT or concomitant treatment the CRA will provide a Hand Written Query (HWQ) to Pfizer.

- Dosing: Dispensed/Returned Logs:

Complete Week 40 Log as an I*Net Page, therefore 'Date Returned' to be provided at Week 48 Visit.

Open Week 48 Log on a paper CRF.

- Do Not Complete the Patient Disposition Page if the patient is continuing post Week 48.

090177e1815d915c\Approved\Approved On: 24-Aug-2010 20:15

- Print and request the Principal Investigator signs the Investigator Declaration Page (page 99999) to confirm sign off of patient's data in I*Net database.

Post Week 48: Complete all Visits on a paper CRF. The first full visit is Week 60.

Complete the Investigator Declaration Page (page 99998) post Week 48 when the patient completes all required visits in the extension phase or withdraws from the extension phase of the study.

090177e1815d915c\Approved\Approved On: 24-Aug-2010 20:15

Appendix 8. Clinical Protocol Amendment #3

Current Amendment: 3

Amendment No.	Date	Country(ies)	Site(s)
3	02 March 2006	All	All

Previous Amendments:

Amendment No.	Date	Country(ies)	Site(s)
2	20 December 2005	All	All

Amendment No.	Date	Country(ies)	Site(s)
1	05 May 2005	All	All

SUMMARY of significant changes

- Addition of measurement of Height and Weight;
- Correction of the Bilirubin value in Appendix 2.

REASON(S) FOR AMENDMENT

The protocol section(s) that have been amended and the details of the changes are summarized in the following sections.

PROTOCOL SECTION(S) AMENDED

The protocol sections that were amended are detailed below. The format is as follows:

- The "change from" section represents the current text in the protocol. Bolded text is used to indicate the addition of information to the current text, and strike-out of text (~~eg, text~~) is used to show the deletion of information from the current text.
- The "change to" section represents the revised text, with the revisions shown in the "change from" section in normal text.

090177e1815d915c\Approved\Approved On: 24-Aug-2010 20:15

Summary; Visit Schedule

Change From

Procedures	Screening (Day – 42 to -28)	Randomization (Day –7 to –4)	Baseline[a] Day 1	Week 2[b]	Weeks 4, 8, 12, 16, 20, 32, 40[b]	Weeks 24 and 48 or Early Termination[b,c]
Targeted Physical Exam/Vital Signs					X	
**Body Weight/Height[r]**			**X**	**X**	**X**	**X**
Selection/confirmation of OBT Regimen		X				

Footnote 'r' added

r. **Height recorded once. Weight through Week 24 only.**

Change To

Procedures	Screening (Day – 42 to -28)	Randomization (Day –7 to –4)	Baseline[a] Day 1	Week 2[b]	Weeks 4, 8, 12, 16, 20, 32, 40[b]	Weeks 24 and 48 or Early Termination[b,c]
Targeted Physical Exam/Vital Signs					X	
Body Weight/Height[r]			X	X	X	X
Selection/confirmation of OBT Regimen		X				

Footnote

r. Height recorded once. Weight through Week 24 only.

Section 6.2.2; Baseline (Day 1) Evaluation – *Prior to first dose*

Change From

3rd bullet added

Change To

- Body weight (record height at baseline or at a visit through Week 24 only);

090177e1815d915c\Approved\Approved On: 24-Aug-2010 20:15

Section 6.2.3; Week 2 Visit

Change From

2nd bullet added

Change To

- Body weight;

Section 6.2.4; On Study Evaluations (Week 4, 8, 12, 16, 20, 32 and 40)

Change From

2nd bullet added

Change To

- Body weight at all visits through Week 20;

Section 6.2.5; Week 24 and 48 or Early Termination Visit

Change From

2nd bullet added

Change To

- Body weight (at Week 24 only);

Appendix 2; Liver Enzyme Work-up

Change From

>10 ULN AST/ALT
≥**2.**5 ULN Bilirubin

Change To

>10 ULN AST/ALT
≥2**.**5 ULN Bilirubin

090177e1815d915c\Approved\Approved On: 24-Aug-2010 20:15

Appendix 9. Clinical Protocol Amendment #4

Current Amendment: 4

Amendment No. 4	Date<br>24 October 2007	Country (ies)	Site(s)

Previous Amendments:

Amendment No. 3	Date<br>02 March 2006	Country (ies)	Site(s)
Amendment No. 2	Date<br>20 December 2005	Country (ies)	Site(s)
Amendment No. 1	Date 05 May 2005	Country (ies)	Site(s)
Original	Date 24 August 2004	Country (ies)	Site(s)

SUMMARY

Reason(s) for Amendment

The protocol section(s) that have been amended and the details of the changes are summarized in the following sections.

Protocol Section(s) Amended

The protocol sections that were amended are detailed below. The format is as follows:

The “change from” section represents the current text in the protocol. Bolded text is used to indicate the addition of information to the current text, and strike-out of text (eg, ~~text~~) is used to show the deletion of information from the current text.

The “change to” section represents the revised text, with the revisions shown in the “change from” section in normal text.

Section

Change From

Change To

090177e1815d915c\Approved\Approved On: 24-Aug-2010 20:15

090177e1815d915c\Approved\Approved On: 24-Aug-2010 20:15

1. Section SUMMARY, Observational Phase

Addition

Observational Phase

After a patient has reached their Week 48 visit, they will continue to visit the investigator site for every 12 weeks and on-study evaluations completed, until they reach their Week 96 visit, as stated in section 5.6 of the protocol.

When the last subject enrolled in the study reaches their week 48 visit the study will be unblinded and following completion of the formal Week 48 analysis by the sponsor, open label drug will be provided to subjects for whom it is medically appropriate to continue, or begin, therapy with UK-427,857. Open label drug will be supplied to these subjects, until each subject reaches 5 years from their first dose of double-blind study treatment .

Because the study population has a diverse visit schedule, subjects entering the observational phase will be divided based on whether they have or not had a Week 96 visit at the time.

Subjects that will reach their Week 96 visit

Subject's reaching their Week 96 visit, (which will also be classified as their End of Study visit) this visit will mark the completion of the active phase of the study. All subjects, upon discontinuation from the active phase of the study, will begin the follow-up phase of the study. This phase of the study will include subjects being reconsented and collection of long term survival and selected endpoint (LTS/SE) data including liver failure, myocardial infarction and ischemia, malignancies, Category C events, infections reported as serious adverse events, and rhabdomyolysis. This data will be collected in an electronic data collection system every six months, for a minimum of 5 years timed from first dose of double-blind study treatment. If subjects are receiving maraviroc, it will continue to be provided until the end of the observational portion of the study.

Subjects that have passed their Week 96 visit

Subject's who have passed their Week 96 visit will have their next scheduled visit as the completion of the active phase of the study. All subjects, upon discontinuation from the active phase of the study, will begin the follow-up phase of the study. This phase of the study will include subjects being reconsented and collection of LTS/SE data including liver failure, myocardial infarction and ischemia, malignancies, Category C events, infections reported as serious adverse events, and rhabdomyolysis every six months, for a minimum of 5 years timed from first dose of double-blind study treatment. If subjects are receiving maraviroc, it will continue to be provided until the end of the observational portion of the trial.

Previously Discontinued Subjects

In compliance with regulatory recommendations, an attempt (two documented telephone calls and one certified letter) will be made to follow up on subjects who have previously permanently discontinued study treatment but who are no longer returning for visits under the "*In study Off Drug*" phase, for LTS /SE data. If the subject has died any information concerning the clinical aspects and circumstances of death, death certificate, and autopsy should be obtained. This will be initiated by the investigator.

2. Section, Schedule of Activities

Change From

VISIT SCHEDULE:

Procedures	Screening (Day –42 to -28)	Randomization (Day –7 to –4)	Baseline[a] Day 1	Week 2 [b]	Weeks 4, 8, 12, 16, 20, 32, 40[b,s]	Weeks 24 and 48 or Early Termination [b,c]	**Observational follow-up (Every 6 months)**
Informed Consent and Eligibility Check	X						**X[t]**
Medical History			X				
Physical Exam/Vital Signs			X			X	
Targeted Physical Exam/Vital Signs					X		
Body Weight/Height [r]			X	X	X	X	
Selection/confirmation of OBT Regimen		X					**X**
Adverse Events			X	X	X	X	
Concomitant Medications			X	X	X	X	
Chemistry, Hematology	X	X[q]	X	X	X	X	
Fasting Metabolic Assessment (total cholesterol, HDL/LDL, triglycerides, glucose, glycosylated hemoglobin)			X			X	

090177e1815d915c\Approved\Approved On: 24-Aug-2010 20:15

12-lead Electrocardiogram			X			X[u]	
Orthostatic BP Monitoring	X		X[d]	X		X	
PK Sampling[e]				X	X	X	
Urinalysis			X			X	
Hepatitis screen (B core Ab, sAg, sAb, C Ab)	X						
Hepatitis C Virus RNA[f]			X		X	X	
CD4/CD8	X		X	X	X	X	
Plasma HIV-1 RNA	X	X	X	X	X	X	
Pregnancy Test[g]	X		X		X	X	
Plasma/PBMC /Proviral DNA Storage[h]			X		X	X	
Viral Resistance (Phenotype, Genotype)[i]	X				X[j]	X[o]	
Co-receptor tropism (Phenotype, Genotype[k])	X		X		X[j, l]	X[o]	
Host Genotyping			X[p]				
Free T4, TSH			X			X	
Dispense Study Medication			X	X[m]	X	X[n]	**X**
Assess Dosing Compliance				X	X	X	
**Long term survival and selected endpoint assessment (LTS/SE)**							**X**

090177e1815d915c\Approved\Approved On: 24-Aug-2010 20:15

a. Day 1, prior to dosing.
b. All visits must occur within +/- 4 days.
c. Subjects who discontinue study drug due to treatment failure or for other reasons must be followed per protocol until Week 48.
d. Patients with asymptomatic postural hypotension at the Baseline visit will be monitored for 4 hours following the first dose of study drug.
e. Two 5 ml PK samples are required at Weeks 2 and 24 and must be at least 30 minutes apart. One 5 ml PK sample required at other visits. Through Week 24 only.
f. If Hepatitis C antibody is positive at Screening Visit, to be performed at Baseline, Weeks 12, 24 and 48 or Early Termination.
g. For Women of Child Bearing Potential. Serum pregnancy at Screening and Urine Tests at the following visits. A positive Urine test must be confirmed with a serum test.
h. Plasma aliquots (2 of 1 mL each) at all timepoints. PBMCs, and proviral DNA will be stored at Baseline and at Weeks 24 and 48 or upon treatment failure only.
i. Reverse transcriptase, protease and fusion inhibitor resistance testing at screening to determine background regimen and at Week 24/48 if viral load >500 copies/mL or upon treatment failure.
j. Upon treatment failure as defined in the protocol (this sample is to be drawn when the confirmatory plasma HIV-1 RNA sample is collected).
k. Genotype (V3 loop alone or as part of gp 160 sequencing) at Baseline, Weeks 24 and 48 and at treatment failure only.
l. Weeks 4, 8, 16, 32 and 40 only.
m. Container from previous visit.
n. At Week 48 or Early Termination, medication will be dispensed to subjects who have completed 48 weeks of therapy and for whom it is medically appropriate to continue or begin therapy with UK-427,857. (See Section 5.6.).
o. Except at Early Termination if a treatment failure (sample should be drawn when confirmatory HIV-1 RNA is collected).
p. Unless prohibited by local regulations.
q. Chemistry only.
r. Height recorded once. Weight through Week 24 only.
s. Also applicable for visits every 12 weeks post-Week 48 (ie. Week 60, 72, 84, 96, 108, 120, etc.) until the last enrolled subject has reached the Week 96 visit (with the exception of PK sampling and ECGs)
t. Informed consent for the observational phase will be sought prior to enrolment from subjects who have permanently discontinued or were lost to follow-up and from subjects who are still enrolled in the study whether in open-label or in the "in-study off drug".
u. ECGs are not required at scheduled or early termination visits post Week 48. Any ECGs done after week 48 will be done at the clinical discretion of the investigator and will not be sent to eRT .

090177e1815d915c\Approved\Approved On: 24-Aug-2010 20:15

Change To
VISIT SCHEDULE:

Procedures	Screening (Day –42 to -28)	Randomization (Day –7 to –4)	Baseline[a] Day 1	Week 2 [b]	Weeks 4, 8, 12, 16, 20, 32, 40[b,s]	Weeks 24 and 48 or Early Termination [b,c]	Observational follow-up (Every 6 months)
Informed Consent and Eligibility Check	X						X[t]
Medical History			X				
Physical Exam/Vital Signs			X			X	
Targeted Physical Exam/Vital Signs					X		
Body Weight/Height[r]			X	X	X	X	
Selection/confirmation of OBT Regimen		X					X
Adverse Events			X	X	X	X	
Concomitant Medications			X	X	X	X	
Chemistry, Hematology	X	X[q]	X	X	X	X	
Fasting Metabolic Assessment (total cholesterol, HDL/LDL, triglycerides, glucose, glycosylated hemoglobin)			X			X	
12-lead Electrocardiogram			X			X[u]	
Orthostatic BP Monitoring	X		X[d]	X		X	
PK Sampling[e]				X	X	X	
Urinalysis			X			X	
Hepatitis screen (B core Ab, sAg, sAb, C Ab)	X						
Hepatitis C Virus RNA[f]			X		X	X	
CD4/CD8	X		X	X	X	X	
Plasma HIV-1 RNA	X	X	X	X	X	X	
Pregnancy Test[g]	X		X		X	X	
Plasma/PBMC/Proviral DNA Storage[h]			X		X	X	
Viral Resistance (Phenotype, Genotype)[i]	X				X[j]	X[o]	
Co-receptor tropism (Phenotype, Genotype[k])	X		X		X[j, l]	X[o]	
Host Genotyping			X[p]				
Free T4, TSH			X			X	

090177e1815d915c\Approved\Approved On: 24-Aug-2010 20:15

Dispense Study Medication			X	X[m]	X	X[n]	X
Assess Dosing Compliance				X	X	X	
Long term survival and selected endpoint assessment (LTS/SE)							X

a. Day 1, prior to dosing.
b. All visits must occur within +/- 4 days.
c. Subjects who discontinue study drug due to treatment failure or for other reasons must be followed per protocol until Week 48.
d. Patients with asymptomatic postural hypotension at the Baseline visit will be monitored for 4 hours following the first dose of study drug.
e. Two 5 ml PK samples are required at Weeks 2 and 24 and must be at least 30 minutes apart. One 5 ml PK sample required at other visits. Through Week 24 only.
f. If Hepatitis C antibody is positive at Screening Visit, to be performed at Baseline, Weeks 12, 24 and 48 or Early Termination.
g. For Women of Child Bearing Potential. Serum pregnancy at Screening and Urine Tests at the following visits. A positive Urine test must be confirmed with a serum test.
h. Plasma aliquots (2 of 1 mL each) at all timepoints. PBMCs, and proviral DNA will be stored at Baseline and at Weeks 24 and 48 or upon treatment failure only.
i. Reverse transcriptase, protease and fusion inhibitor resistance testing at screening to determine background regimen and at Week 24/48 if viral load >500 copies/mL or upon treatment failure.
j. Upon treatment failure as defined in the protocol (this sample is to be drawn when the confirmatory plasma HIV-1 RNA sample is collected).
k. Genotype (V3 loop alone or as part of gp 160 sequencing) at Baseline, Weeks 24 and 48 and at treatment failure only.
l. Weeks 4, 8, 16, 32 and 40 only.
m. Container from previous visit.
n. At Week 48 or Early Termination, medication will be dispensed to subjects who have completed 48 weeks of therapy and for whom it is medically appropriate to continue or begin therapy with UK-427,857. (See Section 5.6).
o. Except at Early Termination if a treatment failure (sample should be drawn when confirmatory HIV-1 RNA is collected).
p. Unless prohibited by local regulations.
q. Chemistry only.
r. Height recorded once. Weight through Week 24 only.
s. Also applicable for visits every 12 weeks post-Week 48 (ie. Week 60, 72, 84, 96, 108, 120, etc.) until the last enrolled subject has reached the Week 96 visit (with the exception of PK sampling and ECGs)
t. Informed consent for the observational phase will be sought prior to enrolment from subjects who have permanently discontinued or were lost to follow-up and from subjects who are still enrolled in the study whether in open-label or in the “in-study off drug”.
u. ECGs are not required at scheduled or early termination visits post Week 48. Any ECGs done after week 48 will be done at the clinical discretion of the investigator and will not be sent to eRT .

090177e1815d915c\Approved\Approved On: 24-Aug-2010 20:15

3. Section 5, TRIAL TREATMENTS, 5.3. Drug Supplies, Bullet 1

Change From

- Pfizer Global Research and Development will supply UK-427,857 and matching placebo tablets. For the observational portion of study, the Sponsor will supply open label UK-427, 857 for subjects for whom it is medically appropriate.

Change To

- Pfizer Global Research and Development will supply UK-427,857 and matching placebo tablets. For the observational portion of study, the Sponsor will supply open label UK-427, 857 for subjects for whom it is medically appropriate.

4. Section 5, TRIAL TREATMENTS, 5.3.1. Formulation and Packaging

Change From

UK-427,857 tablets will be supplied as 150 mg dosage units. Matching placebo for UK-427,857 will also be supplied. **For the observational portion of study, the Sponsor will also supply open label UK-427, 857 in 300 mg dosage units.**

Change To

UK-427,857 tablets will be supplied as 150 mg dosage units. Matching placebo for UK-427,857 will also be supplied. For the observational portion of study, the Sponsor will also supply open label UK-427, 857 in 300 mg dosage units.

5. Section 5, TRIAL TREATMENTS, 5.3.4. Compliance, Paragraph 1

Change From

Subjects will bring unused UK-427,857/placebo tablets to each study visit. The number of tablets will be counted and, if more than expected, subjects will be asked to account for missed doses. The Investigator and medical monitor will also evaluate subject compliance with the study regimen based on information such as plasma HIV-1 RNA levels. Potential reasons for non-compliance with UK-427,857 dosing (ie, AEs, lost medication) will be followed up by the study site personnel and strategies to improve dosing compliance will be explored. Compliance with optimized background therapy will be assessed through the use of a patient adherence diary. **Compliance will not be assessed during the observational phase of the study.**

Change To

Subjects will bring unused UK-427,857/placebo tablets to each study visit. The number of tablets will be counted and, if more than expected, subjects will be asked to account for missed doses. The Investigator and medical monitor will also evaluate subject compliance with the study regimen based on information such as plasma HIV-1 RNA levels. Potential reasons for

090177e1815d915c\Approved\Approved On: 24-Aug-2010 20:15

non-compliance with UK-427,857 dosing (ie, AEs, lost medication) will be followed up by the study site personnel and strategies to improve dosing compliance will be explored. Compliance with optimized background therapy will be assessed through the use of a patient adherence diary. Compliance will not be assessed during the observational phase of the study.

6. Section 5, TRIAL TREATMENTS, 5.4. Drug Storage and Drug Accountability, Paragraph 2

Change From

To ensure adequate records, all study drug will be accounted for in the case report form and drug accountability inventory forms as instructed by Pfizer. **During the observational phase of the study, study drug will be accounted for only in the drug accountability inventory form.** Unless otherwise authorized by Pfizer, all unallocated or unused drug supplies must be returned to Pfizer or its appointed agent (eg, CRO) at the end of the clinical trial. Subjects must return all bottles to the Investigator, who will then return the bottles to Pfizer or an appointed agent (eg, CRO) for review of dosing records and destruction.

Change To

To ensure adequate records, all study drug will be accounted for in the case report form and drug accountability inventory forms as instructed by Pfizer. During the observational phase of the study, study drug will be accounted for only in the drug accountability inventory form. Unless otherwise authorized by Pfizer, all unallocated or unused drug supplies must be returned to Pfizer or its appointed agent (eg, CRO) at the end of the clinical trial. Subjects must return all bottles to the Investigator, who will then return the bottles to Pfizer or an appointed agent (eg, CRO) for review of dosing records and destruction.

7. **Section 5, TRIAL TREATMENTS, 5.5. Concomitant Medication(s), Table**

Change From

**Concomitant Antiretrovirals**	**Recommended UK-427,857 Dose**
≥1 PI and/or delavirdine	150 mg
All other regimens	300 mg
**Efavirenz or etravirine when not in the presence of a PI or other strong CYP3A4 inhibitor***	**600 mg**

*** The use of efavirenz or etravirine with tipranavir is not recommended.**

090177e1815d915c\Approved\Approved On: 24-Aug-2010 20:15

Change To

**Concomitant Antiretrovirals**	**Recommended UK-427,857 Dose**
≥1 PI and/or delavirdine	150 mg
All other regimens	300 mg
Efavirenz or etravirine when not in the presence of a PI or other strong CYP3A4 inhibitor*	600 mg

* The use of efavirenz or etravirine with tipranavir is not recommended.

8. Section 5, TRIAL TREATMENTS, 5.6 Rescue Therapy

Change From

If a patient meets the criteria for treatment failure or discontinues for another reason (eg, pregnancy, adverse event) and requires an alternative regimen, they will be followed **where possible** until ~~the Week 48 visit~~ **they reach their Week 96 visit** according to protocol **visit and assessment** guidelines **(ie. In-study off drug). Each subject will be included in the observational follow-up phase for long-term survival and selected endpoints (LTS/SE) data collection**.

The new regimen, selected by the Investigator based on the results of resistance testing at the time of failure, must be recorded in the CRF. For patients whose virus still remains CCR5-tropic and potentially sensitive to UK-427,857, open-label study drug may be continued during this period. The Investigator, in consultation with the medical monitor, will evaluate the appropriateness of continued therapy with open-label UK-427,857 based on ongoing review of clinical and laboratory parameters. Patients that receive open-label UK-427,857 will follow the protocol designed visit schedule and procedures. If a patients viral load increases beyond three times the **mean baseline** value ~~at the early termination visit~~, upon confirmation of such value, the patient will be considered a treatment failure during the open-label dosing phase. The primary investigator will discontinue open label drug following consultation with the Pfizer medical monitor.

Women who become pregnant during the study period must be unblinded to study drug following end of study assessments. The Investigator, in consultation with the medical monitor, should decide on the optimal antiretroviral regimen on an individualized basis. Protocol-required procedures for follow-up must be performed unless contraindicated by pregnancy. Other appropriate pregnancy follow-up procedures related to perinatal care and neonatal outcome should be performed and ~~noted in the CRF~~ **data provided via an updated Serious Adverse Event Monitoring (AEM) form**. A detailed fetal anomalies ultrasound scan should be performed between Weeks 12 and 16 of gestation and recorded **via submission of an updated**

090177e1815d915c\Approved\Approved On: 24-Aug-2010 20:15

AEM form~~ in the CRF~~. In addition, the Investigator must complete a Pfizer **Exposure *in utero*** ~~pregnancy surveillance~~ form. Infants will be ~~enrolled into an in utero exposed infant follow-on study~~**followed up until 18 months of age with data provided to the Sponsor via an updated AEM form**. ~~This study will have regular assessments and require a development assessment at 18 months.~~

All subjects will continue on double-blind therapy (via IVRS), until the last subject enrolled has reached Week 48 and the study data have undergone formal analysis. Following unblinding of the study, the Sponsor will provide open label UK-427,857 for study subjects for whom it is medically appropriate (see below) to continue or begin therapy with UK-427,857 until each subject reaches 5 years from their first dose of double-blind study treatment. The protocol assessment period will continue until each enrolled subject reaches their Week 96 visit or has been enrolled in the LTS/SE portion of the study; during this time subjects will continue to be monitored in accordance with protocol-specific investigations on an every 12 week visit schedule, but the "On study" procedures (Section 6.2.4) will exclude pharmacokinetic sampling and the collection of ECGs (see Appendix 5). ECGs are not required after the subject's Week 48 visit. While the study endpoints will have been reached, regulatory agencies require further long-term safety data of UK-427,857 in antiretroviral-experienced patients.

As each subject reaches Week 96, they will enter the LTS/SE with their follow up period extending 5 years from their first dose of double-blind study treatment. For subjects that have already passed their Week 96 visit, they will roll into the LTS/SE at their next scheduled regular visit.

Change To

If a patient meets the criteria for treatment failure or discontinues for another reason (eg, pregnancy, adverse event) and requires an alternative regimen, they will be followed where possible until they reach their Week 96 visit according to protocol visit and assessment guidelines (ie, In-study off drug). Each subject will be included in the observational follow-up phase for long-term survival and selected endpoints (LTS/SE) data collection.

The new regimen, selected by the Investigator based on the results of resistance testing at the time of failure, must be recorded in the CRF. For patients whose virus still remains CCR5-tropic and potentially sensitive to UK-427,857, open-label study drug may be continued during this period. The Investigator, in consultation with the medical monitor, will evaluate the appropriateness of continued therapy with open-label UK-427,857 based on ongoing review of clinical and laboratory parameters. Patients that receive open-label UK-427,857 will follow the protocol designed visit schedule and procedures. If a patients viral load increases beyond three times the mean baseline value, upon confirmation of such value, the patient will be considered a treatment failure during the open-label dosing phase. The primary investigator will discontinue open label drug following consultation with the Pfizer medical monitor.

090177e1815d915c\Approved\Approved On: 24-Aug-2010 20:15

Women who become pregnant during the study period must be unblinded to study drug following end of study assessments. The Investigator, in consultation with the medical monitor, should decide on the optimal antiretroviral regimen on an individualized basis. Protocol-required procedures for follow-up must be performed unless contraindicated by pregnancy. Other appropriate pregnancy follow-up procedures related to perinatal care and neonatal outcome should be performed and data provided via an updated Serious Adverse Event Monitoring (AEM) form. A detailed fetal anomalies ultrasound scan should be performed between Weeks 12 and 16 of gestation and recorded via submission of an updated AEM form. In addition, the Investigator must complete a Pfizer Exposure in utero form. Infants will be followed up until 18 months of age with data provided to the Sponsor via an updated AEM form.

All subjects will continue on double-blind therapy (via IVRS), until the last subject enrolled has reached Week 48 and the study data have undergone formal analysis. Following unblinding of the study, the Sponsor will provide open label UK-427,857 for study subjects for whom it is medically appropriate (see below) to continue or begin therapy with UK-427,857 until each subject reaches 5 years from their first dose of double-blind study treatment. The protocol assessment period will continue until each enrolled subject reaches their Week 96 visit or has been enrolled in the LTS/SE portion of the study; during this time subjects will continue to be monitored in accordance with protocol-specific investigations on an every 12 week visit schedule, but the “On study” procedures (Section 6.2.4) will exclude pharmacokinetic sampling and the collection of ECGs (see Appendix 5). ECGs are not required after the subject’s Week 48 visit. While the study endpoints will have been reached, regulatory agencies require further long-term safety data of UK-427,857 in antiretroviral-experienced patients.

As each subject reaches Week 96, they will enter the LTS/SE with their follow up period extending 5 years from their first dose of double-blind study treatment. For subjects that have already passed their Week 96 visit, they will roll into the LTS/SE at their next scheduled regular visit.

9. Section 5, TRIAL TREATMENTS 5.6.1 Subject Eligibility for Open Label Maraviroc After Unblinding of Subjects at Week 48.

Change From

After subjects have reached week 48 and have been unblinded, they may be eligible for open label maraviroc BID. Eligibility will be determined as follows:

- **Subjects who were on maraviroc and are ≤50 copies/ml may continue on maraviroc. All subjects receiving maraviroc QD will be changed to maraviroc BID. Consultation with the medical monitor is not required.**
- Subjects who were on maraviroc and are ≥50 copies/ml and are pending or have met a protocol viral load stopping rule may be eligible for maraviroc BID. Consultation with the medical monitor is required.

090177e1815d915c\Approved\Approved On: 24-Aug-2010 20:15

Subjects who were on placebo and are ≤50 copies/ml will not be eligible for maraviroc. However, if the subject is experiencing toxicity due to their OBT and the investigator feels it would be in the best interest of the subject to change, maraviroc may be considered Consultation with the medical monitor is required.

Subjects who were on placebo and are pending or have met a protocol viral load stopping rule may be eligible for maraviroc. If subjects are enrolled in the LTS/SE and fail on their OBT and have never received maraviroc, may be eligible for open label maraviroc BID. Consultation with the medical monitor is required.

Since the primary endpoint of the study was week 48 and in the interest of the retention of subjects to week 96 and into the LTS/SE, changes in OBT will be allowed. As new anti-retrovirals become available, guidelines for dosing with maraviroc will change. Any changes in the OBT may require dose adjustment for open label maraviroc: please consult the Pfizer Medical Monitor, if you are planning any OBT changes. It is important to continue recording OBT, even when the subject is enrolled in the LTS/SE.

~~The sponsor will provide UK-427,857, until it is commercially available, to subjects who complete 48 weeks of therapy and for whom it is medically appropriate to continue or begin therapy with UK-427,857. This protocol will be extended for a minimum of 1 year, following the last patient's last visit at Week 48. While the regulatory requirement in this patient population will have been fulfilled, we intend to further evaluate the long-term safety and efficacy of UK-427,857 in antiretroviral-experienced patients. During the extended period (minimum of one year), all subjects will be continued on double-blind therapy (via IVRS), until the last subject enrolled has completed their first 48-weeks of therapy. At which time the Sponsor will unblind the study and analyse the data. During this time, patients will be monitored on an every 12-week schedule and the "On Study" procedures (Section) will be performed, apart from pharmacokinetic sampling (see Appendix 5).~~

Change To

After subjects have reached week 48 and have been unblinded, they may be eligible for open label maraviroc BID. Eligibility will be determined as follows:

- Subjects who were on maraviroc and are ≤50 copies/ml may continue on maraviroc. All subjects receiving maraviroc QD will be changed to maraviroc BID. Consultation with the medical monitor is not required.
- Subjects who were on maraviroc and are ≥50 copies/ml and are pending or have met a protocol viral load stopping rule may be eligible for maraviroc BID. Consultation with the medical monitor is required.
- Subjects who were on placebo and are ≤50 copies/ml will not be eligible for maraviroc. However, if the subject is experiencing toxicity due to their OBT and the investigator feels it would be in the best interest of the subject to change, maraviroc may be considered Consultation with the medical monitor is required.

090177e1815d915c\Approved\Approved On: 24-Aug-2010 20:15

- Subjects who were on placebo and are pending or have met a protocol viral load stopping rule may be eligible for maraviroc. If subjects are enrolled in the LTS/SE and fail on their OBT and have never received maraviroc, may be eligible for open label maraviroc BID. Consultation with the medical monitor is required.

Since the primary endpoint of the study was week 48 and in the interest of the retention of subjects to week 96 and into the LTS/SE, changes in OBT will be allowed. As new anti-retrovirals become available, guidelines for dosing with maraviroc will change. Any changes in the OBT may require dose adjustment for open label maraviroc: please consult the Pfizer Medical Monitor, if you are planning any OBT changes. It is important to continue recording OBT, even when the subject is enrolled in the LTS/SE.

10. Section 6. TRIAL PROCEDURES, Bullet 6 and 7 and Paragraph 2

Change From

- Every 12 weeks after Week 48 until each subject reaches Week 96 or beyond but prior to the enactment of the LTS/SE
- Every 6 months after the LTS/SE observational study begins.

Every attempt should be made to keep subjects to visit schedules ±4 days. It is anticipated that subjects may require additional unscheduled clinic visits. If a subject discontinues study therapy **(either blinded or open label)** prior to **reaching** the Week ~~48~~**96** visit, they will complete the End of Study procedures but will remain "in study off drug", keeping to the scheduled visits and procedures ~~at~~**as** outlined. The schedule of evaluations is listed below.

Change To

- Every 12 weeks after Week 48 until each subject reaches Week 96 or beyond but prior to the enactment of the LTS/SE
- Every 6 months after the LTS/SE observational study begins.

Every attempt should be made to keep subjects to visit schedules ±4 days. It is anticipated that subjects may require additional unscheduled clinic visits. If a subject discontinues study therapy (either blinded or open label) prior to reaching the Week 96 visit, they will complete the End of Study procedures but will remain "in study off drug", keeping to the scheduled visits and procedures as outlined. The schedule of evaluations is listed below.

090177e1815d915c\Approved\Approved On: 24-Aug-2010 20:15

11. Section 6. TRIAL PROCEDURES, 6.2.4. Title

Change From

6.2.4 On Study Evaluations (Week 4, 8, 12, 16, 20, 32 and 40). **Post Week 48, every 12 weeks thereafter: Week 60, 72, 84, 96, etc.**

Change To

6.2.4. On Study Evaluations (Week 4, 8, 12, 16, 20, 32 and 40). Post Week 48, every 12 weeks thereafter: Week 60, 72, 84, 96, etc.

12. Section 6. TRIAL PROCEDURES, 6.2. Trial Period, Week 24 and 48 or Early Termination Visit, Bullet 7

Change From

12-lead electrocardiogram and orthostatic BP monitoring (to be performed immediately prior to the first pharmacokinetic sample). **This is NOT required at scheduled or Early Termination visits occurring after Week 48;**

Change To

12-lead electrocardiogram and orthostatic BP monitoring (to be performed immediately prior to the first pharmacokinetic sample). This is NOT required at scheduled or Early Termination visits occurring after Week 48;

13. Section 6. TRIAL PROCEDURES, 6.2.7. Observation Follow-up Phase: Long term survival and selected endpoint (LTS/SE)

Addition

6.2.7 Observation Follow-up Phase: Long term survival and selected endpoint (LTS/SE)

Ongoing study subjects will be asked for their consent prior to entering the LTS/SE observational follow up phase. Subjects will be asked for their consent:

- **to be evaluated for LTS/SE every 6 months; the follow-up phase will continue for the duration of 5 years after first dose of double-blind study treatment;**
- **to provide at least one named secondary contact/next-of-kin who may be contacted by the primary investigator in order to collect LTS/SE data;**
- **to permit the investigator to contact the subject's primary/other treating physician in order to collect LTS/SE data;**

090177e1815d915c\Approved\Approved On: 24-Aug-2010 20:15

- **to provide their national identification or insurance number (where applicable and allowed by local law), so publicly available death registries can be consulted by the investigator site for LTS/SE data;**
- **to allow the investigator site to collect any information concerning the clinical aspects and circumstances of death, death certificate, and autopsy in the event of the subject's death.**

Only anonymous survival outcomes will be made available to the sponsor and regulatory authorities. Personal information will remain confidential with the investigator.

- **For ongoing subjects, survival status will be collected every 6 months from their final scheduled visit. Every 6 months after week 96 (or final scheduled visit), subjects will be questioned about the occurrence of the following events:**
- **liver failure**
- **myocardial infarction and ischemia**
- **malignancies**
- **Category C events**
- **infections reported as serious adverse events**
- **rhabdomyolysis**
- **In subjects who are ISOD or previously discontinued, the follow up may be accomplished by a telephone interview. However, it is the Sponsor's preference to conduct the assessment face to face.**
- **Subjects who previously discontinued**

Subjects who have already discontinued from the study or were lost to follow-up, but did not explicitly withdraw consent, will be contacted (two documented telephone calls and one certified letter) to consent for the observational follow-up phase (LTS/SE). Information concerning mortality will be recorded and each subject will be followed for LTS/SE for 5 years from their first dose of double-blind study treatment.

If the subject consents for LTS/SE, they will be followed for mortality and selected endpoints as above. If the subject consents for follow up of survival status, it will be collected at the time of consent and every 6 months thereafter. If the subject has died any information concerning the clinical aspects and circumstances of death, death certificate, and autopsy should be obtained . . In particular, deaths that are selected endpoints of interest should be recorded as such in the CRF. The status of each subject should be recorded even though the investigator has had no contact or knowledge of the subject. If the subject's status is unknown, the last date the subject was known to be alive will be recorded. In the course of clinical practice, information may become available about the mortality status of a subject by public sources, other patients, or relatives, the report of a death should be recorded.

090177e1815d915c\Approved\Approved On: 24-Aug-2010 20:15

14. Section 6. TRIAL PROCEDURES, 6.5.5. Virology Testing, Blood Pressure, Heart Rate and ECG Monitoring, Bullet 4

Change From

- Early Termination Visit **(ECG is not required after week 48*).***

Change To

- Early Termination Visit (ECG is not required after week 48*).*

15. Section 6. TRIAL PROCEDURES, 6.6. Subject Withdrawal, Paragraphs 5 and 7

Change From

A subject may be withdrawn from study therapy at any time for any of the following reasons; however, they will remain "in study, off drug" and will be monitored according to the protocol specified guidelines until **each subject enrolled reaches** Week **496 or enrolls in the observational phase of the study:**~~8:~~

Efforts will be made to continue to follow up on those subjects who discontinue study drug, "in study, off-drug", until the last subject enrolled reaches Week 96 or enrolls in the observational phase. In addition, unless consent has been withdrawn or is refused, all subjects will be followed up for survival and selected endpoints regardless of the time at which they discontinue, or have discontinued, from the study: this post-study observational period will extend for a minimum of 5 years from each subject's first dose of double-blind study treatment.

Change To

A subject may be withdrawn from study therapy at any time for any of the following reasons; however, they will remain "in study, off drug" and will be monitored according to the protocol specified guidelines until each subject enrolled reaches Week 96 or enrolls in the observational phase of the study:

Efforts will be made to continue to follow up on those subjects who discontinue study drug, "in study, off-drug", until the last subject enrolled reaches Week 96 or enrolls in the observational phase. In addition, unless consent has been withdrawn or is refused, all subjects will be followed up for survival and selected endpoints regardless of the time at which they discontinue, or have discontinued, from the study: this post-study observational period will extend for a minimum of 5 years from each subject's first dose of double-blind study treatment.

090177e1815d915c\Approved\Approved On: 24-Aug-2010 20:15

16. Section 8. ADVERSE EVENT REPORTING, 8.1. Adverse Events, Paragraph 1

Change From

All observed or volunteered adverse events regardless of treatment group or suspected causal relationship to the investigational product(s) will be recorded on the adverse event page(s) of the case report form (CRF)**, except during the observational phase of the study. Adverse events that are on-going at the time of the subject enrolls in the observational phase will be recorded as continuing in the CRF.**

Change To

All observed or volunteered adverse events regardless of treatment group or suspected causal relationship to the investigational product(s) will be recorded on the adverse event page(s) of the case report form (CRF), except during the observational phase of the study. Adverse events that are on-going at the time of the subject enrolls in the observational phase will be recorded as continuing in the CRF.

17. Section 8. ADVERSE EVENT REPORTING, 8.7. Exposure In Utero

Change From

1. **A female becomes, or is found to be, pregnant either while receiving or having been directly exposed to (eg, environmental exposure) the investigational product, or the female becomes, or is found to be, pregnant after discontinuing and/or being directly exposed to the investigational product (maternal exposure).**
2. **A male has been exposed, either due to treatment or environmental, to the investigational product prior to or around the time of conception and/or is exposed during the partner pregnancy (paternal exposure).**

~~a female becomes, or is found to be, pregnant either while receiving or having been exposed to (eg, environmental) an investigational medication or product, or the female becomes, or is found to be, pregnant after discontinuing and/or being exposed to the investigational medication or product.~~

If any trial subject becomes or is found to be pregnant while receiving an investigational medication/product, the investigator must submit this information to the Pfizer medical monitor or designee on an Exposure in Utero Form. This must be done irrespective of whether an adverse event has occurred and within 24 hours of awareness of the pregnancy. The information submitted should include the anticipated date of delivery (see below for information related to induced termination of pregnancy).

090177e1815d915c\Approved\Approved On: 24-Aug-2010 20:15

Change To

1. A female becomes, or is found to be, pregnant either while receiving or having been directly exposed to (eg, environmental exposure) the investigational product, or the female becomes, or is found to be, pregnant after discontinuing and/or being directly exposed to the investigational product (maternal exposure).

2. A male has been exposed, either due to treatment or environmental, to the investigational product prior to or around the time of conception and/or is exposed during the partner pregnancy (paternal exposure).

If any trial subject becomes or is found to be pregnant while receiving an investigational medication/product, the investigator must submit this information to the Pfizer medical monitor or designee on an Exposure in Utero Form. This must be done irrespective of whether an adverse event has occurred and within 24 hours of awareness of the pregnancy. The information submitted should include the anticipated date of delivery (see below for information related to induced termination of pregnancy).

18. Section 8. ADVERSE EVENT REPORTING, 8.10, Reporting Requirements (Serious and Nonserious), Paragraphs 1, 6, and 7

Change From

Each adverse event is to be classified by the investigator as serious or nonserious. This classification determines the reporting procedures to be followed. If a serious adverse event occurs, expedited reporting will follow local and international regulations, as appropriate. SAEs are reportable from the time that the subject provides informed consent, which is obtained prior to the subject's participation in the clinical trial, i.e., prior to undergoing any trial-related procedure and/or receiving investigational product, through and including **a minimum of** 28 calendar days after the last administration of the investigational product. **All SAE's and causality occurring while a subject is participating on the protocol including those patients who are "in study, off drug" must be reported, regardless of the time after the last administration of drug.** Any serious adverse event occurring at any other time after completion of the study must be promptly reported if a causal relationship to study drug is suspected.

All adverse events will be reported on the adverse event page(s) of the CRF**, except during the observation phase of the study**. It should be noted that the form for collection of serious adverse event information is not the same as the adverse event CRF. Where the same data are collected, the forms must be completed in a consistent manner. For example, the same adverse event term should be used on both forms. Adverse events should be reported using concise medical terminology on the CRFs as well as on the form for collection of serious adverse event information.

090177e1815d915c\Approved\Approved On: 24-Aug-2010 20:15

Except during the observational phase of the study, ~~N~~**n**onserious adverse events are to be reported on the adverse event CRFs, which are to be submitted to Pfizer as specified in the adverse event report submission procedure for this protocol.

Change To

Each adverse event is to be classified by the investigator as serious or nonserious. This classification determines the reporting procedures to be followed. If a serious adverse event occurs, expedited reporting will follow local and international regulations, as appropriate. SAEs are reportable from the time that the subject provides informed consent, which is obtained prior to the subject's participation in the clinical trial, ie, prior to undergoing any trial-related procedure and/or receiving investigational product, through and including a minimum of 28 calendar days after the last administration of the investigational product. All SAE's and causality occurring while a subject is participating on the protocol including those patients who are "in study, off drug" must be reported, regardless of the time after the last administration of drug. Any serious adverse event occurring at any other time after completion of the study must be promptly reported if a causal relationship to study drug is suspected.

All adverse events will be reported on the adverse event page(s) of the CRF, except during the observation phase of the study. It should be noted that the form for collection of serious adverse event information is not the same as the adverse event CRF. Where the same data are collected, the forms must be completed in a consistent manner. For example, the same adverse event term should be used on both forms. Adverse events should be reported using concise medical terminology on the CRFs as well as on the form for collection of serious adverse event information.

Except during the observational phase of the study, nonserious adverse events are to be reported on the adverse event CRFs, which are to be submitted to Pfizer as specified in the adverse event report submission procedure for this protocol.

090177e1815d915c\Approved\Approved On: 24-Aug-2010 20:15

Appendix 10. Clinical Protocol Amendment 5

Current Amendment: 5

Amendment No. 5	Date<br>17 June 2008	Country (ies)	Site(s)

Previous Amendments:

Amendment No. 4	Date	Country (ies)	Site(s)
Amendment No. 3	Date<br>02 March 2006	Country (ies)	Site(s)
Amendment No. 2	Date<br>20 December 2005	Country (ies)	Site(s)
Amendment No. 1	Date<br>05 May 2005	Country (ies)	Site(s)

SUMMARY

Reason(s) for Amendment

The protocol section(s) that have been amended and the details of the changes are summarized in the following sections.

Protocol Section(s) Amended

The protocol sections that were amended are detailed below. The format is as follows:

- The “change from” section represents the current text in the protocol. Bolded text is used to indicate the addition of information to the current text, and strike-out of text (eg, ~~text~~) is used to show the deletion of information from the current text.
- The “change to” section represents the revised text, with the revisions shown in the “change from” section in normal text.

Section <Insert section number> , <Insert section title> , Page
<Insert page number as appropriate>

Change From

Change To

090177e1815d915c\Approved\Approved On: 24-Aug-2010 20:15

1. Section, SUMMARY, Observational Phase, Paragraph 3

Change From

Because the study population has a diverse visit schedule, subjects entering the observational phase will be divided based on whether they have or not had a Week 96 visit at the time. **Subjects may transition to the observational phase at an unplanned visit.**

Change To

Because the study population has a diverse visit schedule, subjects entering the observational phase will be divided based on whether they have or not had a Week 96 visit at the time. Subjects may transition to the observational phase at an unplanned visit.

2. Section, SUMMARY, Subjects that will reach their Week 96 visit, Paragraph 1, Sentence 4

Change From

. . . . This data will be collected ~~in an electronic data collection system~~ every six months, for a minimum of 5 years timed from first dose of double-blind study treatment.

Change To

. . . . This data will be collected every six months, for a minimum of 5 years timed from first dose of double-blind study treatment.

3. **Section, SUMMARY, Subjects that have passed their Week 96 visit, Paragraph 1, Sentence 4**

Change From

. . . . ~~Data will be collected in an electronic data collection system.~~ If subjects are receiving maraviroc, it will continue to be provided until the end of the observational portion of the trial.

Change To

. . . . If subjects are receiving maraviroc, it will continue to be provided until the end of the observational portion of the trial.

090177e1815d915c\Approved\Approved On: 24-Aug-2010 20:15

4. Section 5. TRIAL TREATMENTS, 5.5. Concomitant Medication(s), Table, Footnotes and Bullet 6

Change From

Concomitant Antiretrovirals	Recommended UK-427,857 Dose
≥1 PI **(except tipranavir/ritonavir)**** ** and/or delavirdine	150 mg
All other regimens	300 mg
Efavirenz or etravirine when not in the presence of a PI-or other strong CYP3A4 inhibitor*	600 mg

PI = protease inhibitor
* The use of efavirenz or etravirine with tipranavir is not recommended.

****The recommended dose of maraviroc is 300 mg unit dose twice daily when tipranavir/ritonavir is included as part of optimized background therapy.**

- ~~Use of tipranavir as part of OBT is not allowed at the time of this amendment (amendment 2) per the requirements of the DSMB. The investigators will be notified via a letter, if the DSMB removes this restriction. The recommended dose of maraviroc is 300 mg unit dose (either once or twice daily depending on the treatment arm to which the patient is randomized) when tipranavir/ritonavir is included as part of optimized background therapy.~~

Change To

Concomitant Antiretrovirals	Recommended UK-427,857 Dose
≥1 PI (except tipranavir/ritonavir)** and/or delavirdine	150 mg
All other regimens	300 mg
Efavirenz or etravirine when not in the presence of a PI-or other strong CYP3A4 inhibitor*	600 mg

PI = protease inhibitor
* The use of efavirenz or etravirine with tipranavir is not recommended.

**The recommended dose of maraviroc is 300 mg unit dose twice daily when tipranavir/ritonavir is included as part of optimized background therapy.

6. Section 5. TRIAL TREATMENTS, 5.6. Rescue Therapy, Paragraph 3

Change From

Women who become pregnant during the study period must be unblinded to study drug following end of study assessments. The Investigator, in consultation with the medical monitor, should decide on the optimal antiretroviral regimen on an individualized basis. Protocol-required procedures for follow-up must be performed unless contraindicated by pregnancy. Other appropriate pregnancy follow-up procedures related to perinatal care and neonatal outcome

090177e1815d915c\Approved\Approved On: 24-Aug-2010 20:15

should be performed and data provided via an updated Serious Adverse Event Monitoring (AEM) form. A detailed fetal anomalies ultrasound scan should be performed between Weeks 12 and 16 of gestation and recorded via submission of an updated AEM form. In addition, the Investigator must complete a Pfizer Exposure in utero form. **Infants inadvertently exposed to maraviroc in utero during Pfizer clinical trials will be referred, if geographical access permits, to select US and European sites that are already conducting follow-up studies of ARV-exposed infants.**
~~Infants will be followed up until 18 months of age with data provided to the Sponsor via an updated AEM form.~~

Change To

Women who become pregnant during the study period must be unblinded to study drug following end of study assessments. The Investigator, in consultation with the medical monitor, should decide on the optimal antiretroviral regimen on an individualized basis. Protocol-required procedures for follow-up must be performed unless contraindicated by pregnancy. Other appropriate pregnancy follow-up procedures related to perinatal care and neonatal outcome should be performed and data provided via an updated Serious Adverse Event Monitoring (AEM) form. A detailed fetal anomalies ultrasound scan should be performed between Weeks 12 and 16 of gestation and recorded via submission of an updated AEM form. In addition, the Investigator must complete a Pfizer Exposure in utero form. Infants inadvertently exposed to maraviroc in utero during Pfizer clinical trials will be referred, if geographical access permits, to select US and European sites that are already conducting follow-up studies of ARV-exposed infants.

7. Section 6. TRIAL PROCEDURES, 6.2.2. Baseline (Day 1) Evaluation – *Prior to first dose,* Bullet 16

Change From

- Urine pregnancy testing for WOCBP (See Section 4.1). A positive urine test will require a confirmatory ~~a~~ serum pregnancy test;

Change To

- Urine pregnancy testing for WOCBP (See Section 4.1). A positive urine test will require a confirmatory serum pregnancy test;

8. Section 6. TRIAL PROCEDURES, 6.2.4. On Study Evaluations (Week 4, 8, 12, 16, 20, 32 and 40). Post Week 48, every 12 weeks thereafter: Week 60, 72, 84, 96, etc. Bullet 11

Change From

- Urine pregnancy testing for WOCBP (See Section 4.1). A positive urine test will require a confirmatory ~~a~~ serum pregnancy test;

Change To

090177e1815d915c\Approved\Approved On: 24-Aug-2010 20:15

- Urine pregnancy testing for WOCBP (See Section 4.1). A positive urine test will require a confirmatory serum pregnancy test;

9. Section 6. TRIAL PROCEDURES, 6.2.5. Week 24 and 48 or Early Termination Visit, Bullet 14

Change From

- Urine pregnancy testing for WOCBP (See Section 4.1). A positive urine test will require a confirmatory ~~a~~ serum pregnancy test; ~~A positive urine test will require a confirmatory a serum pregnancy test;~~

Change To

- Urine pregnancy testing for WOCBP (See Section 4.1). A positive urine test will require a confirmatory serum pregnancy test;

10. Section 8. ADVERSE EVENT REPORTING, 8.7. Exposure In Utero, Paragraphs 2, 3, and 6

Change From

If any study subject or study subject's partner becomes or is found to be pregnant during the study subject's treatment with the investigational product, the investigator must submit this information to Pfizer on an Exposure in Utero Form. ~~If any trial subject becomes or is found to be pregnant while receiving an investigational medication/product, the investigator must submit this information to the Pfizer medical monitor or designee on an Exposure in Utero Form.~~**In addition, the investigator must submit information regarding environmental exposure to a Pfizer product in a pregnant woman (eg, a nurse reports that she is pregnant and has been exposed to a cytotoxic product by inhalation or spillage) using the Exposure in Utero Form.** This must be done irrespective of whether an adverse event has occurred and within 24 hours of awareness of the pregnancy. The information submitted should include the anticipated date of delivery (See below for information related to induced termination of pregnancy).

Follow-up is conducted to obtain pregnancy outcome information on all Exposure in Utero reports with an unknown outcome. The investigator will follow the subject until completion of the pregnancy or until pregnancy termination (ie, induced abortion) and then notify the Pfizer medical monitor or designee of the outcome. The investigator will provide this information as a follow up to the initial Exposure in Utero Form. The reason(s) for an induced abortion should be specified. An EIU report is not created when an ectopic pregnancy report is received since this pregnancy is not usually viable. Rather, an SAE case is created with the event of ectopic pregnancy.

Additional information regarding the exposure in utero may be requested by the investigator. Further follow-up of birth outcomes will be handled on a case-by-case basis (eg,. follow-up on preterm infants to identify developmental delays).In the case of paternal

090177e1815d915c\Approved\Approved On: 24-Aug-2010 20:15

exposure, the investigator must obtain permission from the subject's partner in order to conduct any follow-up or collect any information.

Change To

If any study subject or study subject's partner becomes or is found to be pregnant during the study subject's treatment with the investigational product, the investigator must submit this information to Pfizer on an Exposure in Utero Form. In addition, the investigator must submit information regarding environmental exposure to a Pfizer product in a pregnant woman (eg, a nurse reports that she is pregnant and has been exposed to a cytotoxic product by inhalation or spillage) using the Exposure in Utero Form. This must be done irrespective of whether an adverse event has occurred and within 24 hours of awareness of the pregnancy. The information submitted should include the anticipated date of delivery (See below for information related to induced termination of pregnancy).

Follow-up is conducted to obtain pregnancy outcome information on all Exposure in Utero reports with an unknown outcome. The investigator will follow the subject until completion of the pregnancy or until pregnancy termination (ie, induced abortion) and then notify the Pfizer medical monitor or designee of the outcome. The investigator will provide this information as a follow up to the initial Exposure in Utero Form. The reason(s) for an induced abortion should be specified. An EIU report is not created when an ectopic pregnancy report is received since this pregnancy is not usually viable. Rather, an SAE case is created with the event of ectopic pregnancy.

Additional information regarding the exposure in utero may be requested by the investigator. Further follow-up of birth outcomes will be handled on a case-by-case basis (eg, follow-up on preterm infants to identify developmental delays).In the case of paternal exposure, the investigator must obtain permission from the subject's partner in order to conduct any follow-up or collect any information.

11. Section Appendix 5, Letter i.

Change From

i. Urine pregnancy testing for WOCBP (see Section 4.1). A positive urine test will require a confirmatory ~~a~~ serum pregnancy test;

Change To

i. Urine pregnancy testing for WOCBP (see Section 4.1). A positive urine test will require a confirmatory serum pregnancy test;

090177e1815d915c\Approved\Approved On: 24-Aug-2010 20:15

Appendix 11. Clinical Protocol Amendment 6

Current Amendment: 6

Amendment No. 6	Date<br>10 August 2010	Country (ies)	Site(s)

Previous Amendments:

Amendment No. 5	Date<br>17 June 2008	Country (ies)	Site(s)
Amendment No. 4	Date<br>24 October 2007	Country (ies)	Site(s)
Amendment No. 3	Date<br>02 March 2006	Country (ies)	Site(s)
Amendment No. 2	Date<br>20 December 2005	Country (ies)	Site(s)
Amendment No. 1	Date<br>05 May 2005	Country (ies)	Site(s)

SUMMARY

Reason(s) for Amendment

The 6th Amendment to this protocol reflects the change of sposor for this study from Pfizer Inc. to ViiV Healthcare. ViiV Healthcare as the sponsor (“ViiV Healthcare” or “Sponsor”) will continue to use Pfizer and Pfizer’s designated agents (“Pfizer”) to conduct the study.

The protocol section(s) that have been amended and the details of the changes are summarized in the following sections.

Protocol Section(s) Amended

The protocol sections that were amended are detailed below. The format is as follows:

- The “change from” section represents the current text in the protocol. Bolded text is used to indicate the addition of information to the current text, and strike-out of text (eg, ~~text~~) is used to show the deletion of information from the current text.
- The “change to” section represents the revised text, with the revisions shown in the “change from” section in normal text.

Section <Insert section number> , <Insert section title> , Page <Insert page number as appropriate>

090177e1815d915c\Approved\Approved On: 24-Aug-2010 20:15

Change From

Change To

1. Section Title Page, Logo and Footer

Change From

This document contains confidential information belonging to ~~Pfizer~~**ViiV Healthcare.** Except as otherwise agreed to in writing, by accepting or reviewing this document, you agree to hold this information in confidence and not to disclose it to others (except where required by applicable law) or to use it for unauthorized purposes. In the event of any actual or suspected breach of this obligation, Pfizer **and/or ViiV Healthcare** should be promptly notified.

~~Pfizer~~ ~~Company~~ Confidential

Change To

This document contains confidential information belonging to ViiV Healthcare. Except as otherwise agreed to in writing, by accepting or reviewing this document, you agree to hold this information in confidence and not to disclose it to others (except where required by applicable law) or to use it for unauthorized purposes. In the event of any actual or suspected breach of this obligation, Pfizer and/or ViiV Healthcare should be promptly notified.

Confidential

2. Section 3. TRIAL DESIGN, paragraph 3

Change From

A Data Safety Monitoring Board (DSMB) will review the results following treatment of 100 patients for at least 8 weeks. No formal statistical tests will be performed. If the DSMB feels that either of the doses of UK-427,857 show substantial evidence of harm to patients, then the DSMB will recommend ending recruitment to this treatment arm. In addition, ~~Pfizer Worldwide Safety and Risk Management~~**The Sponsor or its designee** will conduct regular

090177e1815d915c\Approved\Approved On: 24-Aug-2010 20:15

blinded reviews of serious adverse events (weekly) and adverse clinical and laboratory events (monthly) with particular focus on new infections, liver enzyme abnormalities and postural hypotension.

Change To

A Data Safety Monitoring Board (DSMB) will review the results following treatment of 100 patients for at least 8 weeks. No formal statistical tests will be performed. If the DSMB feels that either of the doses of UK-427,857 show substantial evidence of harm to patients, then the DSMB will recommend ending recruitment to this treatment arm. In addition, The Sponsor or its designee will conduct regular blinded reviews of serious adverse events (weekly) and adverse clinical and laboratory events (monthly) with particular focus on new infections, liver enzyme abnormalities and postural hypotension.

3. Section 4.2. Exclusion Criteria, list numbers 11 and 12:

Change From

11. Total bilirubin, greater than 2.5 times the upper limit of normal (unless unconjugated hyperbilirubinemia due to atazanavir or indinavir). Changes of one or more grades in total bilirubin between screening and the randomization visit should be reviewed with the ~~Pfizer~~ **Study** medical monitor before the initiation of double-blind study medication;

12. AST or ALT greater than 5 times the upper limit of normal. Changes of one or more grades in AST or ALT between screening and the randomization visit should be reviewed with the ~~Pfizer~~ **Study** medical monitor before the initiation of double-blind study medication;

Change To

11. Total bilirubin, greater than 2.5 times the upper limit of normal (unless unconjugated hyperbilirubinemia due to atazanavir or indinavir). Changes of one or more grades in total bilirubin between screening and the randomization visit should be reviewed with the Study medical monitor before the initiation of double-blind study medication;

12. AST or ALT greater than 5 times the upper limit of normal. Changes of one or more grades in AST or ALT between screening and the randomization visit should be reviewed with the Study medical monitor before the initiation of double-blind study medication;

4. Section 5.3. Drug Supplies, list bullet 1:

Change From

- **ViiV Healthcare or it designee** will supply UK-427,857 and matching placebo tablets. For the observational portion of study, the Sponsor will supply open label UK-427, 857 for subjects for whom it is medically appropriate.

090177e1815d915c\Approved\Approved On: 24-Aug-2010 20:15

Change To

- ViiV Healthcare or it designee will supply UK-427,857 and matching placebo tablets. For the observational portion of study, the Sponsor will supply open label UK-427, 857 for subjects for whom it is medically appropriate

5. Section 5.4. Drug Storage and Drug Accountability, paragraph 2:

Change From

To ensure adequate records, all study drug will be accounted for in the case report form and drug accountability inventory forms as instructed by Pfizer. During the observational phase of the study, study drug will be accounted for only in the drug accountability inventory form. Unless otherwise authorized by Pfizer**,** all unallocated or unused drug supplies must be returned to ~~Pfizer~~ **the Sponsor** or its appointed agent (eg, CRO) at the end of the clinical trial. Subjects must return all bottles to the Investigator, who will then return the bottles to ~~Pfizer~~ **the Sponsor** or an appointed agent (eg, CRO) for review of dosing records and destruction.

Change To

To ensure adequate records, all study drug will be accounted for in the case report form and drug accountability inventory forms as instructed by Pfizer. During the observational phase of the study, study drug will be accounted for only in the drug accountability inventory form. Unless otherwise authorized by Pfizer, all unallocated or unused drug supplies must be returned to the Sponsor or its appointed agent (eg, CRO) at the end of the clinical trial. Subjects must return all bottles to the Investigator, who will then return the bottles to the Sponsor or an appointed agent (eg, CRO) for review of dosing records and destruction.

6. Section 5.6. Rescue Therapy, paragraphs 2 and 3:

Change From

The new regimen, selected by the Investigator based on the results of resistance testing at the time of failure, must be recorded in the CRF. For patients whose virus still remains CCR5-tropic and potentially sensitive to UK-427,857, open-label study drug may be continued during this period. The Investigator, in consultation with the medical monitor, will evaluate the appropriateness of continued therapy with open-label UK-427,857 based on ongoing review of clinical and laboratory parameters. Patients that receive open-label UK-427,857 will follow the protocol designed visit schedule and procedures. If a patients viral load increases beyond three times the mean baseline value upon confirmation of such value, the patient will be considered a treatment failure during the open-label dosing phase. The primary investigator will discontinue open label drug following consultation with the ~~Pfizer~~ **Study** medical monitor.

090177e1815d915c\Approved\Approved On: 24-Aug-2010 20:15

Women who become pregnant during the study period must be unblinded to study drug following end of study assessments. The Investigator, in consultation with the medical monitor, should decide on the optimal antiretroviral regimen on an individualized basis. Protocol-required procedures for follow-up must be performed unless contraindicated by pregnancy. Other appropriate pregnancy follow-up procedures related to perinatal care and neonatal outcome should be performed and data provided via an updated Serious Adverse Event Monitoring (AEM) form. A detailed fetal anomalies ultrasound scan should be performed between Weeks 12 and 16 of gestation and recorded via submission of an updated AEM form. In addition, the Investigator must complete a ~~Pfizer~~**n** Exposure in utero form. Infants inadvertently exposed to maraviroc in utero during ~~Pfizer~~ **this** clinical trial~~s~~ will be referred, if geographical access permits, to select US and European sites that are already conducting follow-up studies of ARV-exposed infants.

Change To

The new regimen, selected by the Investigator based on the results of resistance testing at the time of failure, must be recorded in the CRF. For patients whose virus still remains CCR5-tropic and potentially sensitive to UK-427,857, open-label study drug may be continued during this period. The Investigator, in consultation with the medical monitor, will evaluate the appropriateness of continued therapy with open-label UK-427,857 based on ongoing review of clinical and laboratory parameters. Patients that receive open-label UK-427,857 will follow the protocol designed visit schedule and procedures. If a patients viral load increases beyond three times the mean baseline value upon confirmation of such value, the patient will be considered a treatment failure during the open-label dosing phase. The primary investigator will discontinue open label drug following consultation with the Study medical monitor.

Women who become pregnant during the study period must be unblinded to study drug following end of study assessments. The Investigator, in consultation with the medical monitor, should decide on the optimal antiretroviral regimen on an individualized basis. Protocol-required procedures for follow-up must be performed unless contraindicated by pregnancy. Other appropriate pregnancy follow-up procedures related to perinatal care and neonatal outcome should be performed and data provided via an updated Serious Adverse Event Monitoring (AEM) form. A detailed fetal anomalies ultrasound scan should be performed between Weeks 12 and 16 of gestation and recorded via submission of an updated AEM form. In addition, the Investigator must complete an Exposure in utero form. Infants inadvertently exposed to maraviroc in utero during this clinical trial will be referred, if geographical access permits, to select US and European sites that are already conducting follow-up studies of ARV-exposed infants.

090177e1815d915c\Approved\Approved On: 24-Aug-2010 20:15

7. Section 5.6.1. Subject Eligibility for Open Lable Maraviroc After Unblinding of Subject at Week 48, paragraph 2:

Change From

Since the primary endpoint of the study was week 48 and in the interest of the retention of subjects to week 96 and into the LTS/SE, changes in OBT will be allowed. As new anti-retrovirals become available, guidelines for dosing with maraviroc will change. **Any changes in the OBT may require dose adjustment for open label maraviroc: please consult the ~~Pfizer~~** Study **Medical Monitor, if you are planning any OBT changes. It is important to continue recording OBT, even when the subject is enrolled in the LTS/SE.**

Change To

Since the primary endpoint of the study was week 48 and in the interest of the retention of subjects to week 96 and into the LTS/SE, changes in OBT will be allowed. As new anti-retrovirals become available, guidelines for dosing with maraviroc will change. **Any changes in the OBT may require dose adjustment for open label maraviroc: please consult the Study Medical Monitor, if you are planning any OBT changes. It is important to continue recording OBT, even when the subject is enrolled in the LTS/SE.**

8. Section 6.4. Drug Assay:

Change From

At a time determined by the study monitor, the samples will be transported in dry ice to Covance Central Laboratory and stored until being shipped to Tandem Analytics where they will be assayed for UK-427,857 using a previously validated method. Final UK-427,857 plasma concentration data will be transferred to the ~~Pfizer Clinical Data System~~**Sponsor**. All samples must be stored and shipped according to the procedures detailed in the Central Laboratory Manual.

Change To

At a time determined by the study monitor, the samples will be transported in dry ice to Covance Central Laboratory and stored until being shipped to Tandem Analytics where they will be assayed for UK-427,857 using a previously validated method. Final UK-427,857 plasma concentration data will be transferred to the Sponsor. All samples must be stored and shipped according to the procedures detailed in the Central Laboratory Manual.

090177e1815d915c\Approved\Approved On: 24-Aug-2010 20:15

9. Section 6.6. Subject Withdrawal, paragraph 3 and list bullet 12:

Change From

The primary reason for a patient discontinuing from study drug or the clinical study will be recorded in the CRF. The investigator must determine the primary reason for discontinuation. Withdrawal due to adverse events must be distinguished from withdrawal due to insufficient efficacy. A discontinuation must be reported immediately to ~~Pfizer~~ **the Study** medical monitor or his/her designated representative if it is due to a serious adverse event. If a subject is discontinued from the study or study treatment due to a drug-related adverse event, the subject must be followed until the adverse event has resolved or until the event is determined to be chronic or stable in nature. The End of Study procedures must be performed at the time of discontinuation (and prior to unblinding) from either study drug or from the clinical study.

- The study is terminated by ~~Pfizer~~**the Sponsor**;

Change To

The primary reason for a patient discontinuing from study drug or the clinical study will be recorded in the CRF. The investigator must determine the primary reason for discontinuation. Withdrawal due to adverse events must be distinguished from withdrawal due to insufficient efficacy. A discontinuation must be reported immediately to the Study medical monitor or his/her designated representative if it is due to a serious adverse event. If a subject is discontinued from the study or study treatment due to a drug-related adverse event, the subject must be followed until the adverse event has resolved or until the event is determined to be chronic or stable in nature. The End of Study procedures must be performed at the time of discontinuation (and prior to unblinding) from either study drug or from the clinical study.

- The study is terminated by the Sponsor;

10. Section 8.1. Adverse Events, paragraph 2:

Change From

For all adverse events, the investigator must pursue and obtain information adequate both to determine the outcome of the adverse event and to assess whether it meets the criteria for classification as a serious adverse event requiring immediate notification to Pfizer or its designated representative (See Section 8.4). For all adverse events, sufficient information should be obtained by the investigator to determine the causality of the adverse event. The investigator is required to assess causality and indicate that assessment on the CRF. Follow-up of the adverse event, after the date of therapy discontinuation, is required if the adverse event or its sequelae persist. Follow-up is required until the event or its sequelae resolve or stabilize at a level acceptable to the investigator and the Pfizer **Study** medical monitor or designee.

090177e1815d915c\Approved\Approved On: 24-Aug-2010 20:15

Change To

For all adverse events, the investigator must pursue and obtain information adequate both to determine the outcome of the adverse event and to assess whether it meets the criteria for classification as a serious adverse event requiring immediate notification to Pfizer or its designated representative (See Section 8.4). For all adverse events, sufficient information should be obtained by the investigator to determine the causality of the adverse event. The investigator is required to assess causality and indicate that assessment on the CRF. Follow-up of the adverse event, after the date of therapy discontinuation, is required if the adverse event or its sequelae persist. Follow-up is required until the event or its sequelae resolve or stabilize at a level acceptable to the investigator and the Study medical monitor or designee.

11. Section 8.3. Abnormal Laboratory Findings, paragraph 2:

Change From

Changes of one or more grades in total bilirubin between screening and the randomization visit (including the Randomization visit results) should be reviewed with the ~~Pfizer~~ **Study** medical monitor before the initiation of double-blind study medication. Laboratory safety tests will be performed at the following time points: (See Study Schedule/Flowchart).

Change To

Changes of one or more grades in total bilirubin between screening and the randomization visit (including the Randomization visit results) should be reviewed with the Study medical monitor before the initiation of double-blind study medication. Laboratory safety tests will be performed at the following time points: (See Study Schedule/Flowchart).

12. Section 8.3.2. Follow of Laboratory Test Abnormalities, paragraph 3:

Change From

The results of all laboratory tests required by the protocol will be recorded in the subject's case report form. All clinically important abnormal laboratory tests occurring during the study will be repeated at appropriate intervals until they return either to baseline or to a level deemed acceptable by the investigator and the ~~Pfizer~~ **Study** clinical monitor (or his/her designated representative), or until a diagnosis that explains them is made.

Change To

The results of all laboratory tests required by the protocol will be recorded in the subject's case report form. All clinically important abnormal laboratory tests occurring during the study will be repeated at appropriate intervals until they return either to baseline or to a level deemed acceptable by the investigator and the Study clinical monitor (or his/her designated representative), or until a diagnosis that explains them is made.

090177e1815d915c\Approved\Approved On: 24-Aug-2010 20:15

090177e1815d915c\Approved\Approved On: 24-Aug-2010 20:15

13. Section 8.7. Exposure In Utero, section:

Change From

For investigational products within clinical trials and for marketed products, an Exposure In-Utero (EIU) occurs if:

1. A female becomes, or is found to be, pregnant either while receiving or having been directly exposed to (eg, environmental exposure) the investigational product, or the female becomes, or is found to be, pregnant after discontinuing and/or being directly exposed to the investigational product (maternal exposure).
2. A male has been exposed, either due to treatment or environmental, to the investigational product prior to or around the time of conception and/or is exposed during the partner pregnancy (paternal exposure).

If any study subject or study subject's partner becomes or is found to be pregnant during the study subject's treatment with the investigational product, the investigator must submit this information to Pfizer on an Exposure in Utero Form. In addition, the investigator must submit information regarding environmental exposure to a Pfizer product in a pregnant woman (eg, a nurse reports that she is pregnant and has been exposed to a cytotoxic product by inhalation or spillage) using the Exposure in Utero Form. This must be done irrespective of whether an adverse event has occurred and within 24 hours of awareness of the pregnancy. The information submitted should include the anticipated date of delivery (See below for information related to induced termination of pregnancy).

Follow-up is conducted to obtain pregnancy outcome information on all Exposure in Utero reports with an unknown outcome. The investigator will follow the subject until completion of the pregnancy or until pregnancy termination (ie, induced abortion) and then notify the Pfizer medical monitor or designee of the outcome. The investigator will provide this information as a follow up to the initial Exposure in Utero Form. The reason(s) for an induced abortion should be specified. An EIU report is not created when an ectopic pregnancy report is received since this pregnancy is not usually viable. Rather, an SAE case is created with the event of ectopic pregnancy.

If the outcome of the pregnancy meets the criteria for immediate classification as a serious adverse event (ie, spontaneous abortion, stillbirth, neonatal death, or congenital anomaly [including that in an aborted fetus, stillbirth or neonatal death]), the investigator should follow the procedures for reporting serious adverse events.

In the case of a live birth, the "normality" of the newborn can be assessed at the time of birth (ie, no minimum follow-up period of a presumably normal infant is required before an Exposure in Utero Form can be completed). The "normality" of an aborted fetus can be assessed by gross visual inspection, unless pre-abortion test findings are suggestive of a congenital anomaly.

Additional information about pregnancy outcomes that are classified as serious adverse events follows:

- ~~"Spontaneous abortion" includes miscarriage and missed abortion.~~
- ~~All neonatal deaths that occur within 1 month of birth should be reported, without regard to causality, as serious adverse events. In addition, any infant death after 1 month that the investigator assesses as possibly related to the in utero exposure to the investigational medication should be reported.~~

~~Additional information regarding the exposure in utero may be requested by the investigator. Further follow-up of birth outcomes will be handled on a case-by-case basis (eg, follow-up on preterm infants to identify developmental delays). In the case of paternal exposure, the investigator must obtain permission from the subject's partner in order to conduct any follow-up or collect any information.~~

For investigational products within clinical trials and for marketed products, an Exposure In-Utero (EIU) occurs if:

1. **A female becomes, or is found to be, pregnant either while receiving or having been directly exposed to (eg, environmental exposure) the medicinal product, or the female becomes, or is found to be, pregnant after discontinuing and/or being directly exposed to the medicinal product (maternal exposure).**
2. **A male has been exposed, either due to treatment or environmental, to the medicinal product prior to or around the time of conception and/or is exposed during the partner pregnancy (paternal exposure).**

In each case meeting the definition of exposure *in utero* involving either a ViiV Healthcare medicinal product or a Pfizer medicinal product, whether investigational or marketed, the investigator must submit the required information to Pfizer on an Exposure in Utero Form. The investigator must submit information regarding study subjects or their partners and in the case of environmental exposure to ViiV Healthcare medicinal product or a Pfizer medicinal product in a pregnant woman (eg, a nurse reports that she is pregnant and has been exposed to a cytotoxic product by inhalation or spillage) using the Exposure in Utero Form. This must be done irrespective of whether an adverse event has occurred and within 24 hours of awareness of the pregnancy. The information submitted should include the anticipated date of delivery (See below for information related to induced termination of pregnancy).

Follow-up is conducted to obtain pregnancy outcome information on all Exposure in Utero reports with an unknown outcome. The investigator will follow the subject until completion of the pregnancy or until pregnancy termination (ie, induced abortion) and then notify the Study medical monitor or designee of the outcome. The investigator will provide this information as a follow up to the initial Exposure in Utero Form. The reason(s) for an induced abortion should be specified. An EIU report is not created when an ectopic pregnancy report is received since this pregnancy is not usually viable. Rather, an SAE case is created with the event of ectopic pregnancy.

090177e1815d915c\Approved\Approved On: 24-Aug-2010 20:15

If the outcome of the pregnancy meets the criteria for immediate classification as a serious adverse event (ie, spontaneous abortion, stillbirth, neonatal death, or congenital anomaly [including that in an aborted fetus, stillbirth or neonatal death]), the investigator should follow the procedures for reporting serious adverse events.

In the case of a live birth, the "normality" of the newborn can be assessed at the time of birth (ie, no minimum follow-up period of a presumably normal infant is required before an Exposure in Utero Form can be completed). The "normality" of an aborted fetus can be assessed by gross visual inspection, unless pre-abortion test findings are suggestive of a congenital anomaly.

Additional information about pregnancy outcomes that are classified as serious adverse events follows:

- **"Spontaneous abortion" includes miscarriage and missed abortion.**
- **All neonatal deaths that occur within 1 month of birth should be reported, without regard to causality, as serious adverse events. In addition, any infant death after 1 month that the investigator assesses as possibly related to the in utero exposure to the investigational medication should be reported.**

Additional information regarding the exposure in utero may be requested by the investigator. Further follow-up of birth outcomes will be handled on a case-by-case basis (eg, follow-up on preterm infants to identify developmental delays). In the case of paternal exposure, the investigator must obtain permission from the subject's partner in order to conduct any follow-up or collect any information.

Change To

For investigational products within clinical trials and for marketed products, an Exposure In-Utero (EIU) occurs if:

1. A female becomes, or is found to be, pregnant either while receiving or having been directly exposed to (eg, environmental exposure) the medicinal product, or the female becomes, or is found to be, pregnant after discontinuing and/or being directly exposed to the medicinal product (maternal exposure).
2. A male has been exposed, either due to treatment or environmental, to the medicinal product prior to or around the time of conception and/or is exposed during the partner pregnancy (paternal exposure).

In each case meeting the definition of exposure *in utero* involving either a ViiV Healthcare medicinal product or a Pfizer medicinal product, whether investigational or marketed, the investigator must submit the required information to Pfizer on an Exposure in Utero Form. The investigator must submit information regarding study subjects or their partners and in the case of environmental exposure to ViiV Healthcare medicinal product or a Pfizer medicinal product in a pregnant woman (eg, a nurse reports that she is pregnant and has been exposed to a cytotoxic product by inhalation or spillage) using the Exposure in Utero Form. This must be done

090177e1815d915c\Approved\Approved On: 24-Aug-2010 20:15

irrespective of whether an adverse event has occurred and within 24 hours of awareness of the pregnancy. The information submitted should include the anticipated date of delivery (See below for information related to induced termination of pregnancy).

Follow-up is conducted to obtain pregnancy outcome information on all Exposure in Utero reports with an unknown outcome. The investigator will follow the subject until completion of the pregnancy or until pregnancy termination (ie, induced abortion) and then notify the Study medical monitor or designee of the outcome. The investigator will provide this information as a follow up to the initial Exposure in Utero Form. The reason(s) for an induced abortion should be specified. An EIU report is not created when an ectopic pregnancy report is received since this pregnancy is not usually viable. Rather, an SAE case is created with the event of ectopic pregnancy.

If the outcome of the pregnancy meets the criteria for immediate classification as a serious adverse event (ie, spontaneous abortion, stillbirth, neonatal death, or congenital anomaly [including that in an aborted fetus, stillbirth or neonatal death]), the investigator should follow the procedures for reporting serious adverse events.

In the case of a live birth, the “normality” of the newborn can be assessed at the time of birth (ie, no minimum follow-up period of a presumably normal infant is required before an Exposure in Utero Form can be completed). The “normality” of an aborted fetus can be assessed by gross visual inspection, unless pre-abortion test findings are suggestive of a congenital anomaly.

Additional information about pregnancy outcomes that are classified as serious adverse events follows:

- “Spontaneous abortion” includes miscarriage and missed abortion.
- All neonatal deaths that occur within 1 month of birth should be reported, without regard to causality, as serious adverse events. In addition, any infant death after 1 month that the investigator assesses as possibly related to the in utero exposure to the investigational medication should be reported.

Additional information regarding the exposure in utero may be requested by the investigator. Further follow-up of birth outcomes will be handled on a case-by-case basis (eg, follow-up on preterm infants to identify developmental delays). In the case of paternal exposure, the investigator must obtain permission from the subject’s partner in order to conduct any follow-up or collect any information.

090177e1815d915c\Approved\Approved On: 24-Aug-2010 20:15

3. Section 8.10. Reporting Requirements (Serious and Nonserious), paragraphs 3 and 5:

Change From

If a serious adverse event occurs, the ~~Pfizer~~ **Study** medical monitor or designee is to be notified within 24 hours of awareness of the event by the investigator. In particular, if the serious adverse event is fatal or life-threatening, notification to the ~~Pfizer~~ **Study** medical monitor or designee must be made immediately, irrespective of the extent of available adverse event information. This timeframe also applies to additional new information (follow-up) on previously forwarded serious adverse event reports.

For all serious adverse events, the investigator is obligated to pursue and provide information to the ~~Pfizer~~ **Study** medical monitor or designee in accordance with the timeframes for reporting specified above. In addition, an investigator may be requested by the ~~Pfizer~~ **Study** medical monitor or designee to obtain specific additional follow-up information in an expedited fashion. This information may be more detailed than that captured on the adverse event case report form. In general, this will include a description of the adverse event in sufficient detail to allow for a complete medical assessment of the case and independent determination of possible causality. Information on other possible causes of the event, such as concomitant medications and illnesses must be provided. In the case of a subject death, a summary of available autopsy findings must be submitted as soon as possible to Pfizer or its designated representative.

Change To

If a serious adverse event occurs, the Study medical monitor or designee is to be notified within 24 hours of awareness of the event by the investigator. In particular, if the serious adverse event is fatal or life-threatening, notification to the Study medical monitor or designee must be made immediately, irrespective of the extent of available adverse event information. This timeframe also applies to additional new information (follow-up) on previously forwarded serious adverse event reports.

For all serious adverse events, the investigator is obligated to pursue and provide information to the Study medical monitor or designee in accordance with the timeframes for reporting specified above. In addition, an investigator may be requested by the Study medical monitor or designee to obtain specific additional follow-up information in an expedited fashion. This information may be more detailed than that captured on the adverse event case report form. In general, this will include a description of the adverse event in sufficient detail to allow for a complete medical assessment of the case and independent determination of possible causality. Information on other possible causes of the event, such as concomitant medications and illnesses must be provided. In the case of a subject death, a summary of available autopsy findings must be submitted as soon as possible to Pfizer or its designated representative.

090177e1815d915c\Approved\Approved On: 24-Aug-2010 20:15

4. Section 10. QUALITY CONTROL AND QUALITY ASSURANCE, paragraphs 1 and 2:

Change From

During trial conduct, Pfizer, **or its agent, or the Sponsor** ~~or its agent~~ will conduct periodic monitoring visits to ensure that the protocol and GCPs are being followed. The monitors may review source documents to confirm that the data recorded on CRFs is accurate. The investigator and institution will allow Pfizer monitors or its agents, **or the sponsor,** and appropriate regulatory authorities direct access to source documents to perform this verification.

The trial site may be subject to review by the institutional review board (IRB)/independent ethics committee (IEC), and/or to quality assurance audits performed by Pfizer **or the Sponsor**, and/or to inspection by appropriate regulatory authorities from the US or other countries.

Change To

During trial conduct, Pfizer, or its agent, or the Sponsor or its agent will conduct periodic monitoring visits to ensure that the protocol and GCPs are being followed. The monitors may review source documents to confirm that the data recorded on CRFs is accurate. The investigator and institution will allow the Pifzer monitors or its agents, or the sponsor, and appropriate regulatory authorities direct access to source documents to perform this verification.

The trial site may be subject to review by the institutional review board (IRB)/independent ethics committee (IEC), and/or to quality assurance audits performed by the Pfizer or the Sponsor, and/or to inspection by appropriate regulatory authorities from the US or other countries.

5. Section 11.1. Case Report Forms/Electronic Data Record, paragraph 2:

Change From

A CRF is required and should be completed for each included subject. The completed original CRFs are the sole property of ~~Pfizer~~ **the Sponsor** and should not be made available in any form to third parties, except for authorized representatives of ~~Pfizer~~ **the Sponsor** or appropriate regulatory authorities, without written permission from ~~Pfizer~~**the Sponsor**.

Change To

A CRF is required and should be completed for each included subject. The completed original CRFs are the sole property of the Sponsor and should not be made available in any form to third parties, except for authorized representatives of the Sponsor or appropriate regulatory authorities, without written permission from the Sponsor.

090177e1815d915c\Approved\Approved On: 24-Aug-2010 20:15

6. Section 11. Record Retention:

Change From

To enable evaluations and/or audits from regulatory authorities or ~~Pfizer~~**the Sponsor and its authorized representatives**, the investigator agrees to keep records, including the identity of all participating subjects (sufficient information to link records, eg, CRFs and hospital records), all original signed informed consent forms, copies of all CRFs, source documents, and detailed records of treatment disposition. The records should be retained by the investigator according to ICH, local regulations, or as specified in the Clinical Study Agreement, whichever is longer.

If the investigator relocates, retires, or for any reason withdraws from the trial, Pfizer should be prospectively notified. The trial records must be transferred to an acceptable designee, such as another investigator, another institution, or to Pfizer. The investigator must obtain ~~Pfizer's~~ **the Sponsor's** written permission before disposing of any records, even if retention requirements have been met.

Change To

To enable evaluations and/or audits from regulatory authorities or the Sponsor **and its authorized representatives**, the investigator agrees to keep records, including the identity of all participating subjects (sufficient information to link records, eg, CRFs and hospital records), all original signed informed consent forms, copies of all CRFs, source documents, and detailed records of treatment disposition. The records should be retained by the investigator according to ICH, local regulations, or as specified in the Clinical Study Agreement, whichever is longer.

If the investigator relocates, retires, or for any reason withdraws from the trial, Pfizer should be prospectively notified. The trial records must be transferred to an acceptable designee, such as another investigator, another institution, or to Pfizer. The investigator must obtain the Sponsor's written permission before disposing of any records, even if retention requirements have been met.

7. Section 12.3. Subject Information and Consent:

Change From

The informed consent form must be agreed to by ~~Pfizer~~ **the Sponsor** and the IRB/IEC and must be in compliance with ICH GCP, local regulatory requirements, and legal requirements.

The investigator must ensure that each trial subject, or his/her legally acceptable representative, is fully informed about the nature and objectives of the trial and possible risks associated with participation. The investigator will obtain written informed consent from each subject or the subject's legally acceptable representative before any study-specific activity is performed. The informed consent form used in this trial, and any changes made during the course of the trial, must be prospectively approved by both the IRB/IEC and ~~Pfizer~~ **the Sponsor** before use. The investigator will retain a copy of each subject's signed consent form.

090177e1815d915c\Approved\Approved On: 24-Aug-2010 20:15

Change To

The informed consent form must be agreed to by the Sponsor and the IRB/IEC and must be in compliance with ICH GCP, local regulatory requirements, and legal requirements.

The investigator must ensure that each trial subject, or his/her legally acceptable representative, is fully informed about the nature and objectives of the trial and possible risks associated with participation. The investigator will obtain written informed consent from each subject or the subject's legally acceptable representative before any study-specific activity is performed. The informed consent form used in this trial, and any changes made during the course of the trial, must be prospectively approved by both the IRB/IEC and the Sponsor before use. The investigator will retain a copy of each subject's signed consent form.

8. Section 13. SPONSOR DISCONTINUATION CRITERIA:

Change From

Premature termination of this clinical trial may occur because of a regulatory authority decision, change in opinion of the IRB/IEC, drug safety problems, or at the discretion of ~~**Pfizer**~~**the Sponsor**. In addition, ~~Pfizer~~ **the Sponsor** retains the right to discontinue development of UK-427,857 at any time.

Pfizer **The Sponsor** reserves the right to discontinue the trial prior to inclusion of the intended number of subjects, but intends only to exercise this right for valid scientific or administrative reasons. After such a decision, the investigator must contact all participating subjects within 24 hours. As directed by **the Sponsor**~~Pfizer~~, all trial materials must be collected and all CRFs completed to the greatest extent possible.

Change To

Premature termination of this clinical trial may occur because of a regulatory authority decision, change in opinion of the IRB/IEC, drug safety problems, or at the discretion of the Sponsor. In addition, the Sponsor retains the right to discontinue development of UK-427,857 at any time.

The Sponsor reserves the right to discontinue the trial prior to inclusion of the intended number of subjects, but intends only to exercise this right for valid scientific or administrative reasons. After such a decision, the investigator must contact all participating subjects within 24 hours. As directed by the Sponsor, all trial materials must be collected and all CRFs completed to the greatest extent possible.

090177e1815d915c\Approved\Approved On: 24-Aug-2010 20:15
